# Peptide ligand isomerism drives divergent stability and guest binding in Pd_3_L_4_ metal-peptidic cages

**DOI:** 10.1039/d5sc06441d

**Published:** 2025-12-09

**Authors:** Ben E. Barber, Ellen M. G. Jamieson, Leah E. M. White, Charlie T. McTernan

**Affiliations:** a Artificial Molecular Machinery Laboratory, The Francis Crick Institute 1 Midland Road London NW1 1AT UK charlie.mcternan@crick.ac.uk; b Department of Chemistry, King's College London Britannia House, 7 Trinity Street London SE1 1DB UK charlie.mcternan@kcl.ac.uk

## Abstract

The self-assembly of metal–organic cages enables the rapid creation of atomically defined, three-dimensional, nanoscale architectures from easily accessible building blocks. Rigid and flat aromatic panels are typically used as ligands, but limit the diversity and aqueous solubility of cages thus formed. Building on our recent success using oligoprolines to create defined metal-peptidic Pd_2_L_4_ cages with emergent head-to-tail isomer control, we now show that installation of an additional metal-binding motif enables formation of a new family of Pd_3_L_4_ dual-cavity anisotropic ‘peanut’ cages. Using automated solid-phase peptide synthesis enables generation of a ligand series by varying sequence isomer and/or the stereochemistry of the 4*R*/*S*-hydroxyproline. Small differences in ligand isomerism generate four distinct self-assembly outcomes, forming: the Pd_3_L_4_*cis CCNN* cage isomer, the Pd_3_L_4_ ‘All Up’ *CCCC* cage isomer, a mixture of all possible isomers of Pd_3_L_4_ cages, or an interpenetrated Pd_6_L_8_ cage. Finally, these subtle alterations in cage structure led to differing host–guest interactions and strikingly divergent stability profiles for the metal-peptidic cages when exposed to a range of stimuli. Certain isomers remain stable to base for more than six days, while others fully degrade within an hour. This work underscores the advantages of using biological building blocks in supramolecular chemistry to create systems with tuneable properties.

## Abstract 2

We show that using oligoprolines enables us to create defined metal-peptidic cages with three metal-binding motifs, forming a new family of Pd_3_L_4_ dual-cavity anisotropic ‘peanut’ cages.

## Graphical abstract

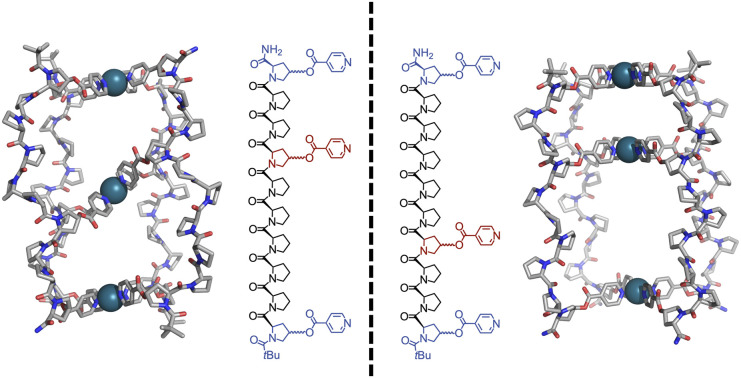

## Introduction

Metal–organic cages are discrete, three-dimensional species formed from the self-assembly of metal ions with rigid, organic ligands.^1–3^ A variety of polyhedra with defined internal cavities can be readily formed, and the function of these systems often stems from the dramatically different properties of the internal cavity from the surrounding solution.^4,5^ Metal–organic cages have been shown to perform challenging separations,^6^ catalyse reactions at rates comparable to enzymes,^7–9^ act as contrast agents or transport cargoes *in vivo*,^10–12^ and sequester contaminants.^13,14^ Flat, aromatic panels are often used to provide the structural rigidity required to favour the self-assembly of defined, discrete species.^15^ This leads to two key problems; firstly, water solubility can be challenging and stability limited, due to the fundamental propensity of building blocks to precipitate from solution.^16^ Secondly, functionalisation of the internal cavity, where the most interesting properties of the cages lie, is challenging and few examples have been reported.^17–19^

Creating anisotropic cavities has been an area of increasing interest, as researchers seek to move away from the pseudospherical cavities of the current generation of metal–organic cages, towards systems better able to mimic the selectivity and potency of biological systems.^20^ The use of less symmetric ligands, and heteroleptic systems, have provided routes to lower symmetry cages with augmented properties.^21^ However, they are still bounded by the limitations of aromatic and conjugated building blocks. Creating functionalised, and particularly chiral, cage cavities remains challenging.^22,23^

One way to functionalise the interior of metal–organic cages is to use tritopic, linear, ligands to create differentiated cavities in a single assembly in ‘peanut’ cages.^24,25^ However, the assembly of ‘peanut’ cages from low symmetry ligands is rare, limiting their anisotropy. Lewis and co-workers have reported a pseudo-heteroleptic Pd_3_L_4_ cage formed from a single asymmetric ligand, with geometric complementarity leading to self-assembly of a single cage isomer.^26^ The anisotropy of such cages is limited, however, by the planarity and lack of chirality of the planar building blocks.

To address these challenges with solubility, biocompatibility, and anisotropy, we recently reported a new class of metal–organic cages with a defined and open structure – metal-peptidic cages – formed from oligoproline ligands, whose defined folding in solution provides the requisite rigidity for the formation of discrete species.^27^ Oligoprolines reliably form a left-handed polyproline II (PPII) helix in aqueous solutions, which contains all *trans*-amide bonds.^28,29^ This secondary structure has a rigid helical structure with a repeat length of 9 Å, with every third residue aligned on the same face of the helix, and tolerates substitution.^30–33^ This creates a platform for the design of intrinsically water-soluble cages whose interior can be functionalised.^34^ We have previously demonstrated that a family of Pd_2_L_4_ cages of different sizes could be readily formed, and that the richly chiral and helical surfaces of these cages led to unusual host–guest behaviour. More recently, Palma and co-workers have reported a modified system using alternative pyridine linkers – highlighting the robustness of the oligoproline platform.^35^ Others have investigated the use of metallo-peptidic systems in discrete multi-nuclear complexes and knots, and extended frameworks, achieving high levels of stereocontrol.^36–44^

**Fig. 1:**
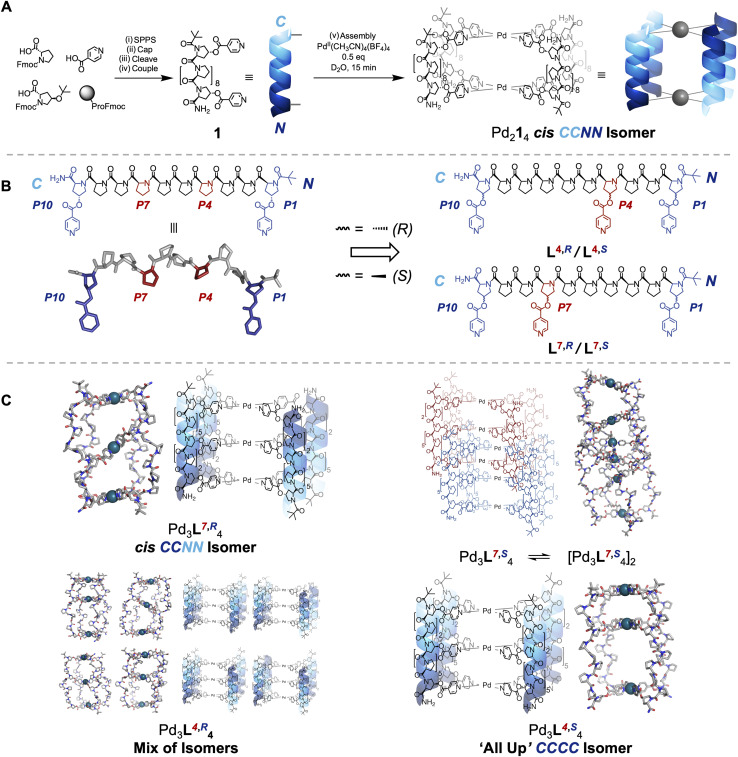
(A) Synthesis of 10mer oligoproline ligand 1 and its self-assembly into Pd_2_1_4_ metal-peptide cage in our previous work.^27^ (i) Solid-phase peptide synthesis (see SI for protocol). (ii) Pivalic anhydride : CH_2_Cl_2_ : DMF 1 : 4.5 : 4.5, r.t., 45 min. (iii) Trifluoroacetic acid : triisopropyl silane : H_2_O 38 : 1:1, r.t, 2 h (iv) EDCI (6 equiv.), DMAP (3 equiv.), isonicotinic acid (6 equiv.), CH_2_Cl_2_, r.t., 16 h. (v) Pd(CH_3_CN)_4_(BF_4_)_2_ (0.5 equiv)., D_2_O, r.t., 15 min. (B) Chemical structure and molecular model of 1, with internally-aligned proline sidechains highlighted in red (left). Additional metal coordinating residues were installed at these positions to give oligoproline ligands L^7,^*^R^*, L^4,^*^R^*, L^7,^*^S^* and L^4,^*^S^* used in this paper (right, see SI for synthesis). (C) Self-assembly outcomes. (i) Pd(CH_3_CN)_4_(BF_4_)_2_ (0.75 equiv.), D_2_O, r.t., 4 days.

Oligoproline ligands are intrinsically directional (the C-terminus is distinct from the N-terminus), and so there are four different head-to-tail cage isomers that can form, even though our cages are homoleptic (Fig. 1B).^45–47^ The C-termini can all be aligned at one end and bind one palladium(ii) ion of the cage (the ‘All Up’ *CCCC*); three C-termini and one N-terminus can lie at one end (the *CCCN*); or there are two cases where two C-termini and two N-termini are at each end, either with C termini *cis* or *trans* to each other across the palladium(ii) centre (*cis CCNN* and *trans CNCN*, respectively). Our previous research found the use of complex, chiral, and helical building blocks led to the unexpected emergence of isomer control, with the *cis CCNN* head-to-tail isomer of cage formed exclusively (Fig. 1A).^27^

Herein, the helical nature and modular synthesis of oligoprolines enables the design of cages with multiple internal cavities, and controlled head-to-tail isomerism, by installing additional metal coordinating residues. Using intrinsically tuneable subcomponents, and automated peptide synthesis, provides a unique platform for the synthesis of tritopic building blocks for Pd_3_L_4_ cages.^48^ Fine adjustments to the structure can be made by changing the peptide sequence and/or the point chirality of amino acid building blocks. This allows us to precisely adjust the relative location and geometry of metal-binding motifs along the peptidic backbone. We hypothesised that such adjustments would enable us to control the outcome, and head-to-tail isomerism, of metal-peptidic cage assembly. Differentiated, highly anisotropic, cavities form and highly complex behaviour can be generated from simple changes to peptide sequence isomers and/or point stereochemistry (Fig. 1). Four isomers of a single ligand deliver four unique outcomes – a *cis CCNN* Pd_3_L_4_ cage, an ‘All Up’ *CCCC* Pd_3_L_4_ cage, a mixture of all possible Pd_3_L_4_ cage isomers, and an interpenetrated Pd_6_L_8_ cage – demonstrating the flexibility and power of using complex chiral building blocks in supramolecular chemistry. Furthermore, each outcome of cage self-assembly shows significant differences in stability to a range of stimuli, and differentiated host–guest chemistry, informing future applications.

## Results and discussion

**Fig. 2:**
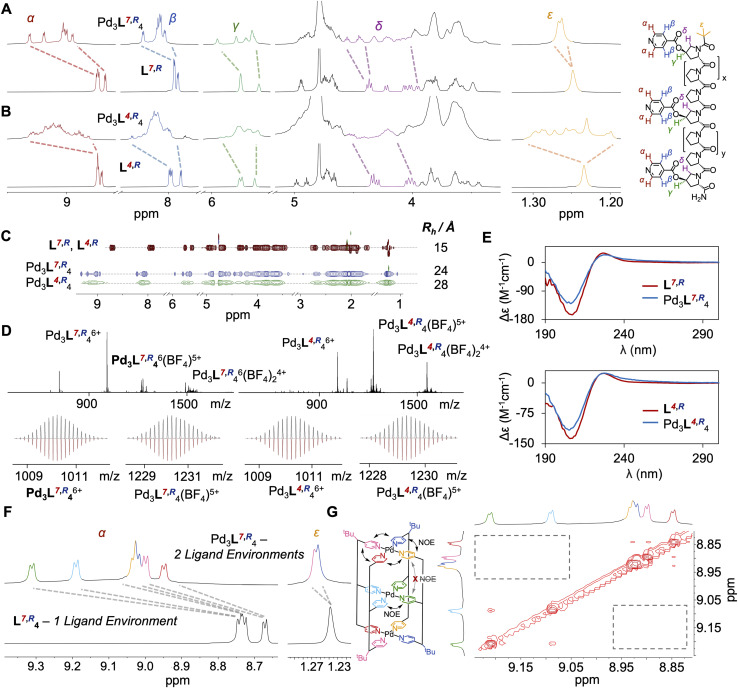
(A) ^1^H NMR (600 MHz, D_2_O, 298 K) of cage Pd_3_L^7,^*^R^*_4_ (top) and ligand L^7,^*^R^* (bottom). (B) ^1^H NMR (600 MHz, D_2_O, 298 K) of cage Pd_3_L^4,^*^R^*_4_ (top) and ligand L^4,^*^R^* (bottom). (C) ^1^H DOSY NMR (600 MHz, D_2_O, 298 K) of ligands L^7,^*^R^*, L^4,^*^R^* (red) and cages Pd_3_L^7,^*^R^*_4_ (blue) and Pd_3_L^4,^*^R^*_4_ (green) with hydrodynamic radii shown. (D) ESI-HRMS data of cages Pd_3_L^7,^*^R^*_4_ (left) and Pd_3_L^4,^*^R^*_4_ (right) and their isotopic distributions (recorded top, simulated bottom). (E) Circular dichroism of ligands L^7,^*^R^*, L^4,^*^R^* (red) and cages Pd_3_L^7,^*^R^*_4_ and Pd_3_L^4,^*^R^*_4_ (blue). (F) ^1^H NMR (600 MHz, D_2_O, 298 K) of cage Pd_3_L^7,^*^R^*_4_ (top) and ligand L^7,^*^R^* (bottom), highlighting the doubling of ^1^H environments upon cage formation, consistent with *cis CCNN* isomer formation. (G) Partial NOESY NMR (800 MHz, D_2_O, 298 K) of Pd_3_L^7,^*^R^*_4_. H_α_ pyridine environments corresponding to the internal co-ordinating motifs are isolated from the N- and C- terminal H_α_ pyridine environments, and the lack of NOEs is highlighted, confirming formation of *cis CCNN* cage isomer.

**Fig. 3:**
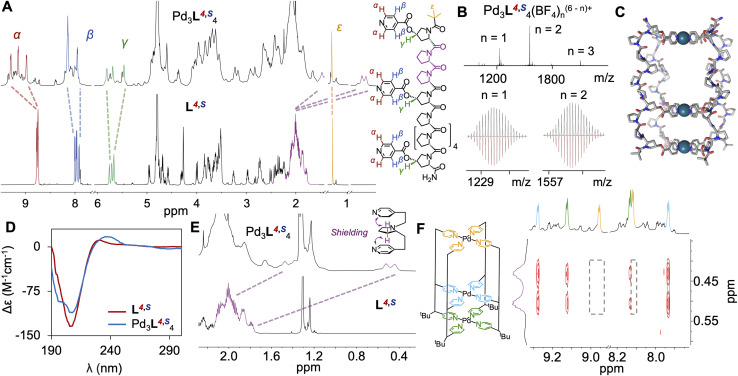
(A) ^1^H NMR (600 MHz, D_2_O, 298 K) of cage Pd_3_L^4,^*^S^*_4_ (top) and ligand L^4,^*^S^* (bottom). (B) ESI-HRMS data of Pd_3_L^4,^*^S^*_4_ with isotopic distributions (recorded top, simulated bottom). (C) Circular dichroism of ligand L^4,^*^S^* (red) and cage Pd_3_L^4,^*^S^*_4_ (blue) showing retention of general PPII structure, but with deviations at c. 240 nm. (D) Molecular model of Pd_3_L^4,^*^S^*_4_ ‘All Up’ *CCCC* isomer. (E) ^1^H NMR (600 MHz, D_2_O, 298 K) showing downfield shifted proline backbone peaks. (F) ^1^H NOESY NMR (950 MHz, D_2_O, 298 K) showing correlations between downfield shifted proline backbone peaks and two of three H_α_ and H_β_ environments, confirming ‘All Up’ *CCCC* isomer assembly.

We initially targeted the installation of an additional metal binding site within the cavity in an attempt to override the intrinsic preference of our Pd_2_L_4_ systems to form *cis CCNN* cages (Fig. 1A),^27^ reasoning that an asymmetrically aligned additional binding site would favour the ‘All Up’ *CCCC* isomer, as the only way to achieve coordinative saturation. The alignment of every third residue on the same face of the helix in an idealised PPII structure provides a direct route to Pd_3_L_4_ cages (Fig. 1B).^28^ Ligands L^7,^*^R^*, L^4,^*^R^*, L^7,^*^S^* and L^4,^*^S^* were synthesised, consisting of seven proline and three hydroxyproline residues, with isonicotinic acids coupled to the Hyp positions, by solid phase peptide synthesis (SPPS), and purified by high performance liquid chromatography (HPLC). Due to the directionality of the PPII helix, in a 10mer helix the additional metal binding site can be closer to the N-terminus (L^4,^*^R^*, L^4,^*^S^*) or the C-terminus (L^7,^*^R^*, L^7,^*^S^*). The stereochemistry of the C–O bond was also varied, as this might further perturb isomer preference. L^4,^*^R^* and L^7,^*^R^* contained 4*R* stereocentres on Hyp sidechains, which occur naturally in biology, whilst L^4,^*^S^* and L^7,^*^S^* contained the unnatural 4*S* stereocentre. A *tert*-butyl carbonyl group was installed to provide additional PPII stability and a distinct NMR handle for cage assembly and assignment of isomer formation.^49^ Folding to a PPII conformation in all ligands (L^7,^*^R^*, L^4,^*^R^*, L^4,^*^S^* and L^7,^*^S^*) in H_2_O was confirmed by CD spectroscopy, showing characteristic minima and maxima at c. 205 and 225 nm (Fig. 2E and 3D, SI Section 9).^50^

With the ligands in hand, L^7,^*^R^* and L^4,^*^R^*, both containing natural 4*R* Hyp stereocentres, were assembled to cages by addition of Pd(CH_3_CN)_4_(BF_4_)_2_ in a precisely 4 : 3 ligand : metal ratio. Discrete species Pd_3_L^7,^*^R^*_4_ and Pd_3_L^4,^*^R^*_4_ were formed in each case (Fig. 2A and B), with downfield shifts in the pyridyl protons indicative of palladium(ii) co-ordination, and desymmetrisation of ligand signals along the oligoproline backbone, indicative of cage assembly. The ^1^H NMR of both assemblies showed changes over time, with initially broad signals sharpening gradually over 24 hours (Fig. S107–S110). This indicates that the self-assembly process faces a higher energetic barrier to equilibration than our Pd_2_L_4_ cages, which were equilibrated within five minutes. We attribute this to the costs of breaking additional coordination bonds (*vide infra*).

^1^H NMR, ^13^C NMR, Correlated Spectroscopy (COSY), Heteronuclear Single Quantum Coherence Spectroscopy (HSQC), Heteronuclear Multiple Bond Correlation Spectroscopy (HMBC), Diffusion-Ordered Spectroscopy (DOSY), High Resolution Electrospray Mass Spectroscopy (ESI-HRMS), Ion Mobility Mass Spectrometry (IMMS) and Circular Dichroism (CD) data were all consistent with the formation of metal-peptidic cages (Fig. 2, S41–S63, S163 and S164). DOSY spectra showed a single diffusion band for Pd_3_L^7,^*^R^*_4_ and Pd_3_L^4,^*^R^*_4_, with a hydrodynamic radius approximately twice that of the free ligand (Fig. 2C), consistent with cage assembly.^51^ ESI-HRMS showed clean formation of cage, and isotopic distributions matching simulations (Fig. 2D). IMMS showed peaks at a collision cross-section of 1046 Å^2^ and 1076 Å^2^ for Pd_3_L^7,^*^R^*_4_ and Pd_3_L^4,^*^R^*_4_, consistent with previous results for the Pd_2_1_4_ species (Fig. S163 and S164).^27,52^ CD confirmed retention of the PPII structure of ligands L^7,^*^R^* and L^4,^*^R^* on cage assembly (Fig. 2E, S166 and S167).

Interestingly, whilst HRMS, CD and DOSY suggested formation of identical species, interrogation of the ^1^H NMR told a different story. Ligand L^7,^*^R^*, where the additional binding site is close to the C-terminus, assembled into a sharply resolved single species Pd_3_L^7,^*^R^*_4_, with two-fold desymmetrisation throughout the ligand strand. Further analysis showed unexpected formation of *cis CCNN*, rather than the expected ‘All Up’.^27^ The assignment of a single isomer, rather than an equal mixture of ‘All Up’ *CCCC* and *trans CNCN* isomers, is further supported by NOESY analysis, with H_α_ pyridine environments corresponding to the internal co-ordinating motifs isolated from the N- and C-terminal H_α_ pyridine environments (Fig. 2G, S182 and S183, see SI Section 10 for detailed reasoning).

The ^1^H NMR spectrum for Pd_3_L^4,^*^R^*_4_ is more complex (Fig. 2B), but clearly resolves eight major *tert*-butyl signals, indicative of eight different ligand environments (Fig. S184). This is further supported by analysis of the aromatic pyridine H_α_ and H_β_ protons (Fig. S181) showing a similar increase in identifiable environments. As no other major species were observed by ESI-HRMS (Fig. S60), and a single species was shown by DOSY, these ligand environments likely correspond to multiple cage isomers being present in solution. To observe eight different environments, all four cage isomers must be present, thus demonstrating a total lack of isomer selectivity, which was consistent across assembly temperatures (Fig. S64–S66). Our original Pd_2_L_4_ cages display an inherent energetic preference for the *cis CCNN* isomer, and our additional modifications are fighting this intrinsic bias (SI Section 12 for further discussion).

We sought to obtain crystallographic data to better understand the difference in cage isomer selectivity between Pd_3_L^7,^*^R^*_4_ and Pd_3_L^4,^*^R^*_4_. However, despite extensive attempts (>200 per sample), no crystals suitable for X-ray diffraction formed, consistent with previously reported difficulties in crystallising PPII structures.^27,28^ Molecular modelling studies were therefore undertaken. Our previous studies of Pd_2_L_4_ systems indicated that the *cis CCNN* cage isomer adopted a tilt, where the helical axis of the oligoproline rods was not perpendicular to the pyridine co-ordination vector (Fig. S197), which significantly decreased the energy of the *cis CCNN* relative to the other isomers.

Modelling of Pd_3_L^7,^*^R^*_4_ shows that the lowest energy isomer of the cage is likewise the tilted *cis CCNN*, with a distortion of the internal palladium co-ordination plane resulting in the formation of two symmetric cavities (Fig. 1C, S229 and S230). Computational models of Pd_3_L^4,^*^R^*_4_ gave the *cis CCNN* isomer as the highest energy structure (SI Section 12), suggesting it is strongly disfavoured. This supports the difference in behaviour between the two systems seen by ^1^H NMR.

Having noted the striking effect of moving the internal binding site between N- and C-terminal proximity, we next investigated the effect of epimerising the C–O bond in Hyp, hypothesising that this would enable further control. Ligands L^7,^*^S^*_4_ and L^4,^*^S^*_4_ (Fig. 1) are diastereoisomers of L^7,^*^R^*_4_ and L^4,^*^R^*_4_ respectively, with 4*S*-hydroxyprolines in place of 4*R*. Additional peaks were observed in the ^1^H NMR spectra of both ligands (Fig. S185 and S186). As both L^7,^*^S^*_4_ and L^4,^*^S^*_4_ were >98% pure by liquid chromatography mass spectrometry (Fig. S28 and S38), these peaks likely correspond to a subpopulation of ligand containing *cis* amide bonds, estimated by NMR to be <10%. This was confirmed by CD spectroscopy, with a reduction in the characteristic PPII peaks for both L^7,^*^S^*_4_ and L^4,^*^S^*_4_ relative to L^7,^*^R^*_4_ and L^4,^*^R^*_4_ (Fig. 3D, S171, S173 and S175). 4*S*-Hyp electron withdrawing substituents are known to destabilise PPII helices,^53–55^ inducing an endo-ring pucker that destabilises *trans* amide bonds relative to 4*R*-Hyp explaining this reduction.^30^

Ligands L^7,^*^S^*_4_ and L^4,^*^S^*_4_ were assembled with Pd(CH_3_CN)_4_(BF_4_)_2_ in a precisely 4 : 3 ligand : metal ratio, and fully characterised (Fig. 3, S67–S92, S165 and S166). Results were consistent with metal-peptidic cage assembly. For L^4,^*^S^*_4_ the ^1^H NMR yielded a sharp and discrete major species Pd_3_L^4,^*^S^*_4_ (c. 70% of the ligand converts to major isomer) after 48 h, in contrast to the mixture of cage isomers observed for Pd_3_L^4,^*^R^*_4_ (Fig. 3A and S113). DOSY showed a single diffusion band, indicating formation of a single species with a hydrodynamic radius of 28 Å (Fig. S85). ESI-HRMS confirmed successful self-assembly of cage Pd_3_L^4,^*^S^*_4_, and isotopic distributions matching simulations (Fig. 3B and S90–S92). IMMS showed a peak for Pd_3_L^4,^*^S^*_4_ at a collision cross-section of 1023 Å^2^, consistent with previous results (Fig. S166).

The major species of Pd_3_L^4,^*^S^*_4_ has a single ligand environment, which is not consistent with a *cis CCNN* metal-peptidic cage. The absence of desymmetrisation indicates formation of either an ‘All Up’ *CCCC* or *trans CNCN* arrangement as the major isomer (see SI Section 10). Geometric considerations, and molecular modelling, suggest the formation of the *trans CNCN* isomer would be unfavourable (see SI Section 12) without complete loss of PPII folding, which is not observed by CD. We therefore assign Pd_3_L^4,^*^S^*_4_ as the ‘All Up’ *CCCC* isomer.

Analysis of the NOESY correlations further supports this assignment (Fig. 3E and F). The NOESY spectrum of Pd_3_L^4,^*^S^*_4_ shows strong correlations and shielding between protons of two of the three pyridines on each ligand and proline sidechains (Fig. 3F and S190), which we have not observed in other systems. Significant upfield shifts of the α, β and γ protons of Pro2/Pro3 are consistent with close proximity to the pyridine's aromatic ring current (Fig. 3E and S191). The most likely mechanism involves compression of the smaller cavity of the ‘All Up’ *CCCC*Pd_3_L^4,^*^S^*_4_ cage. This hypothesis is supported by CD changes of Pd_3_L^4,^*^S^*_4_ with respect to free ligand L^4,^*^S^* (Fig. 3D and S170), and the appearance of new absorbances (the characteristic PPII peak at 230 nm shifts and broadens to 238 nm) which do not correlate with PPII, PPI, or unstructured peptide,^53,56^ and could be due to a twisting and compression of the smaller cavity of Pd_3_L^4,^*^S^*_4_. Given the decrease in PPII helical character (^1^H NMR + CD; Fig. S175 and S186) in ligand L^4,^*^S^* compared to ligand L^4,^*^R^*, this process is likely mediated by *trans* to *cis* isomerism of some amide bonds in the smaller cavity of the cage, which contains a higher local concentration of PPII-destabilising 4*S*-Hyp. Minor species in the ^1^H NMR likely correspond to small amounts of the three other cage isomers, as no other major species were observed by ESI-HRMS (Fig. S90).

Whilst the change in isomer selectivity surprised us, flipping the 4*R*-Hyp stereocentres of L^4,^*^R^* to 4*S*-Hyp does indeed lead to self-assembly of a single Pd_3_L_4_ cage isomer. This novel ‘All Up’ *CCCC* isomer has two separate and highly distinctive cavities, achieving one the initial aims of this project.

Self-assembly of L^7,^*^S^* with Pd(CH_3_CN)_4_(BF_4_)_2_ gave a broad species in the ^1^H NMR spectra which did not resolve over seven days (Fig. S111). ESI-HRMS showed the unexpected formation of both Pd_3_L_4_ and Pd_6_L_8_ species (Fig. S75–S81 and S187). DOSY NMR showed a single diffusion band with a hydrodynamic radius of 33.3 Å (Fig. S187), larger than the hydrodynamic radii seen for the three Pd_3_L_4_ cages Pd_3_L^7,^*^R^*_4_, Pd_3_L^4,^*^R^*_4_ and Pd_3_L^4,^*^S^*_4_ (24.0–28.3 Å), consistent with the presence of a larger metal-peptidic structure.

We assigned this to an interpenetrated Pd_6_L_8_ cage (Pd_3_L^7,*S*^_4_)_2_ formed in equilibrium by the interlocking of two Pd_3_L^7,*S*^_4_ cage complexes. Such structures have been reported in the literature, often driven by exclusion of solvent from cavities.^57–59^ The broadness in the ^1^H NMR is due to increased molecular weight leading to decreased tumbling, but also to the presence of isomers, along with the equilibrium between Pd_3_L^7,*S*^_4_ and (Pd_3_L^7,*S*^_4_)_2_. Dilution of the Pd_6_L_8_ cage (Pd_3_L^7,*S*^_4_)_2_ led to the disappearance of peaks corresponding to the interpenetrated species, leaving the Pd_3_L_4_^6+^ peaks unchanged, supporting this assignment (Fig. S75, S76 and S187).^59,60^ This was supported by DOSY studies, in which a decrease in hydrodynamic radii was observed upon dilution (Fig. S121). Assembly studies (see SI Section 5) showed that an intermediate Pd_2_L_4_ species first forms, before assembly of (Pd_3_L^7,*S*^_4_)_2_. CD showed significant loss of PPII character, consistent with distortion on formation of the assembled cage. As such, the cavity compression seen in Pd_3_L^4,^*^S^*_4_ and the partial catenation seen in (Pd_3_L^7,*S*^_4_)_2_ are different responses to the same stressor – increasing disruption of PPII by incorporation of 4*S*-Hyp.

**Fig. 4:**
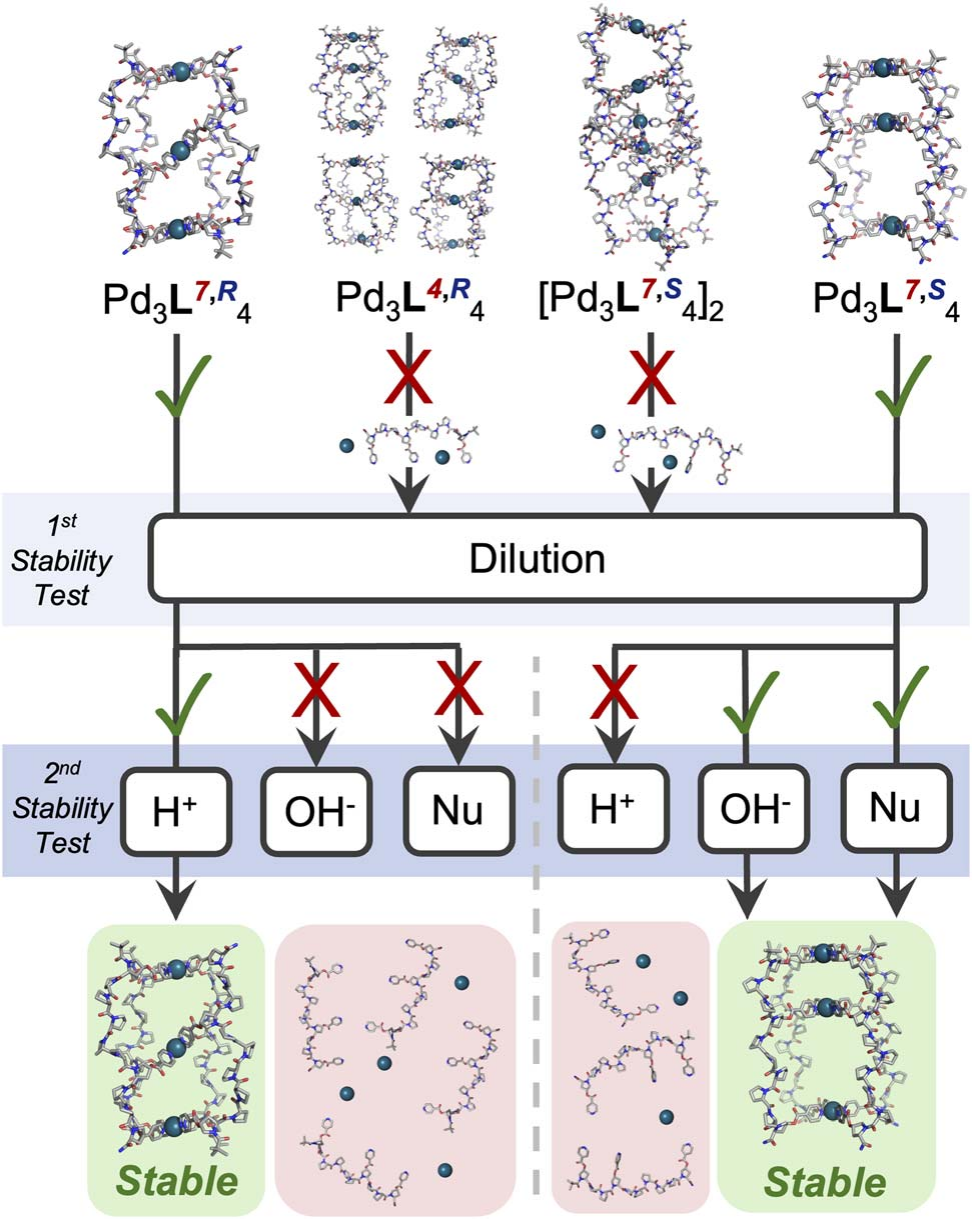
Summary of the results of stability studies showing differential stabilities to dilution; then to acid, base, competitive glutathione/pyridine (Nu) coordination. In each case, the more stable cage is highlighted – Pd_3_L^7,^*^R^*_4_ for acid stability and Pd_3_L^4,^*^S^*_4_ for base and nucleophile stability.

Finally, we sought to probe whether the different cage structures formed had distinct chemical properties. In the longer term, these cages hold promise for applications in biological systems, which requires tolerance to different stimuli. Firstly, the assemblies were diluted, and monitored by ^1^H NMR (Fig. 4, SI Section 7). Cages Pd_3_L^7,^*^R^*_4_ and Pd_3_L^4,^*^S^*_4_, the single isomer cages, were stable to a concentration of 50 µM in D_2_O (Fig. S115, S116, S122 and S123). This represents a fourfold increase from the stability of previously reported Pd_2_1_4_, likely due to the additional co-ordination bonds within Pd_3_L^7,^*^R^*_4_ and Pd_3_L^4,^*^S^*_4_.^27^ In contrast, cages Pd_3_L^4,^*^R^*_4_ and (Pd_3_L^7,^*^S^*_4_)_2_ only showed stability to concentrations of 500 µM in D_2_O (Fig. S117–S120). The most stable cages were then challenged with base, acid, and competitive metal binding ligands – glutathione and pyridine (Fig. 4). Pyridine was chosen due to the ubiquity of nitrogen heterocycles in drug molecules, and glutathione due to its high concentrations in cells and plasma.

Cages Pd_3_L^7,^*^R^*_4_ and Pd_3_L^4,^*^S^*_4_ were first tested for base stability by addition of 4 eq. NaOD, and showed distinct differences in behaviour. Whilst cage Pd_3_L^7,^*^R^*_4_ was almost immediately (<1 h) disassembled and its ester bonds hydrolysed (Fig. S133 and S134), cage Pd_3_L^4,^*^S^*_4_ was only partially disassembled after 120 h (<25% by ^1^H NMR; Fig. S140 and S141).

A similar trend in resilience was observed when Pd_3_L^7,^*^R^*_4_ and Pd_3_L^4,^*^S^*_4_ were challenged with competing ligands – glutathione and pyridine. Addition of 4 eq. of glutathione caused complete disassembly of Pd_3_L^7,^*^R^*_4_ within 5 min, but Pd_3_L^4,^*^S^*_4_ was not completely disassembled until 48 h post addition (Fig. S155, S156, S161 and S162). Both Pd_3_L^7,^*^R^*_4_ and Pd_3_L^4,^*^S^*_4_ showed significantly enhanced stability to pyridine addition, with 4 eq. failing to cause full disassembly even after 48 h (Fig. S143, S144, S151 and S152). However, after addition of 12 eq. pyridine, Pd_3_L^4,^*^S^*_4_ remained detectable in solution for c. 1 h, whilst Pd_3_L^7,^*^R^*_4_ was almost immediately (<10 min) disassembled (Fig. S145, S146, S153 and S154). The partially collapsed smaller cavity of the ‘All Up’ *CCCC*Pd_3_L^4,^*^S^*_4_ cage may provide a more condensed cage structure, in which Pd metal centres and ester bonds are sterically protected from external attack, explaining this difference. The *cis CCNN* cage Pd_3_L^7,^*^R^*_4_ has two symmetric cavities with a more open structure, and could be rapidly attacked by ^−^OD, glutathione, and pyridine. This is supported by the observation of complete hydrolysis of ligand esters upon treatment of cage Pd_3_L^7,^*^R^*_4_ with ^−^OD, whilst Pd_3_L^4,^*^S^*_4_ showed significant residual cage (Fig. S135 and S142).

Remarkably, when cages Pd_3_L^7,^*^R^*_4_ and Pd_3_L^4,^*^S^*_4_ were challenged by addition of 4 eq. DCl, a reversal in the stability trend was seen. The ‘All Up’ Pd_3_L^4,^*^S^*_4_ cage was >80% disassembled after 48 h, whereas only c. 35% of the *cis CCNN*Pd_3_L^7,^*^R^*_4_ cage had been disassembled (Fig. S124, S125, S131 and S132). The difference observed between acid and base addition is likely due to the differing mechanisms of cage destruction, with accessibility of the ester (base hydrolysis) and the pyridine nitrogen (protonation) varying between cages.

**Fig. 5:**
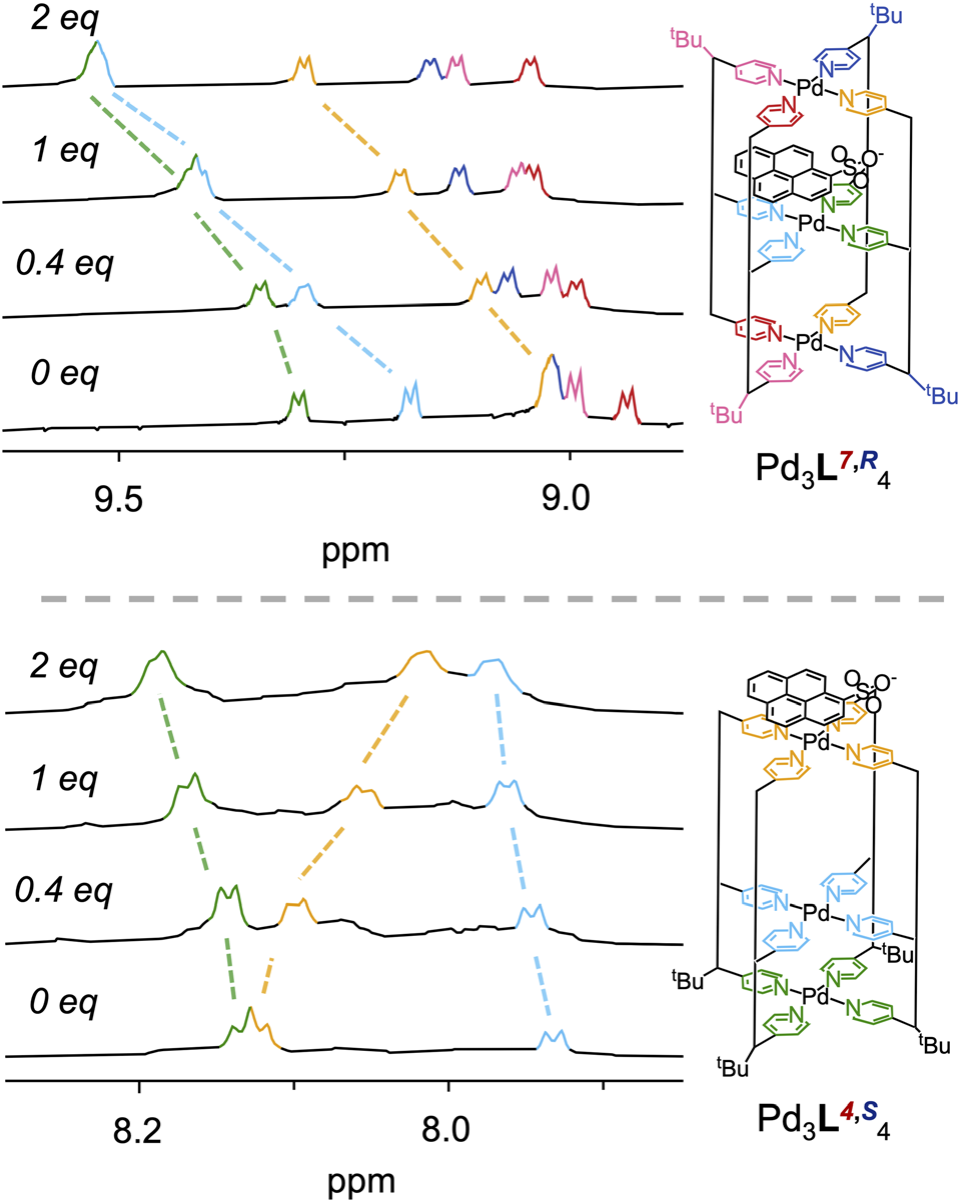
^1^H NMR indicating differential binding modes of cages Pd_3_L^7,^*^R^*_4_, and Pd_3_L^4,^*^S^*_4_ with sodium pyrene-1-sulfonate. Pd_3_L^7,^*^R^*_4_ (top) shows H_α_ Δppm shifts in all (external and internal) pyridines residues, suggesting a cavity bound species whereas Pd_3_L^4,^*^S^*_4_ (bottom) shows significant shifts in only external pyridine residues suggesting guest binding on the external aromatic Pd-pyridine panels of the cage.

Having shown differential stability, varying host–guest properties between the differentially sized cavities were then explored (SI Section 11). Exemplar ^1^H NMR titrations of the *cis CCNN*Pd_3_L^7,^*^R^*_4_ and ‘All Up’ *CCCC*Pd_3_L^4,^*^S^*_4_ cages with negatively charged aromatic anions benzenesulfonate and pyrene-1-sulfonate were performed. Pleasingly, substantial differences in binding constants for sodium benzenesulfonate with Pd_3_L^7,^*^R^*_4_ and Pd_3_L^4,^*^S^*_4_ were found, with the *cis CCNN*Pd_3_L^7,^*^R^*_4_ binding almost an order of magnitude more strongly than the ‘All Up’ *CCCC*Pd_3_L^4,^*^S^*_4_ cage (*K*_a_ = 911 M^−1^ ± 6% for Pd_3_L^7,^*^R^*_4_*vs. K*_a_ = 120 M^−1^ ± 4% for Pd_3_L^4,*S*^_4_, Fig. S192–S204). A similar, but less pronounced, trend of interaction was found with sodium pyrene-1-sulfonate between the two cages (*K*_a_ = 1557 M^−1^ ± 7% for Pd_3_L^7,^*^R^*_4_*vs. K*_a_ = 2192 M^−1^ ± 6% for Pd_3_L^4,*S*^_4_, Fig. S205–S210). Further, evidence of differing binding modes between the two cages was seen by ^1^H NMR (Fig. 5). For Pd_3_L^7,^*^R^*_4_, ^1^H resonances of external and internal pyridine H_α_ residues (Fig. S206) showed significant Δppm shifts (up to c. 0.25 ppm), suggesting a cavity bound species interacting with the central pyridines. In Pd_3_L^4,^*^S^*_4_, only the external Hyp H_γ_ and pyridine residues showed significant Δppm shifts (up to 0.35 ppm for Hyp, Fig. S209), suggesting guest binding on the external aromatic Pd-pyridine panels of the cage, and so evidencing differential binding modes in cages with different cavities (Fig. 5).

Finally, guest binding with Irinotecan, an anticancer agent, was investigated. As Irinotecan is administered clinically as the HCl salt, this provides a bridge between the differential acid stabilities seen and guest binding. Binding constants were similar (*K*_a_ = 2188 M^−1^ ± 6% for Pd_3_L^7,^*^R^*_4_*vs. K*_a_ = 2629 M^−1^ ± 9% for Pd_3_L^4,^*^S^*_4_, Fig. S211–S216), but Pd_3_L^4,^*^S^*_4_, and not Pd_3_L^7,^*^R^*_4_, disassembled at higher Irinotecan. HCl equivalencies (>2 eq.) (Fig. S212 and S215). The interplay of differential guest binding and cage stability, therefore, provides a novel route to control guest binding.

## Conclusions

Herein, we have shown that a series of four isomeric oligoproline ligands can be used to synthesise Pd_3_L_4_ metal-peptidic ‘peanut’ cages. Small changes in ligands, varying structural isomers and point chiral centres, can lead to dramatic changes in self-assembly and stability. Each of the four tritopic ligands produced a different cage upon addition of Pd(ii): L^7,^*^R^* formed the *cis CCNN* Pd_3_L_4_ isomer, L^4,^*^R^* formed all possible Pd_3_L_4_ isomers simultaneously, L^7,^*^S^* formed an interpenetrated Pd_6_L_8_ cage, and L^4,^*^S^* formed the ‘All Up’ *CCCC* Pd_3_L_4_ isomer, generating a diverse range of anisotropic cavities. The *cis* and ‘All Up’ cages were significantly more stable, and showed contrasting differences in stability to various stimuli. They showed differences in host–guest behaviour, and binding site. Future work will explore how the asymmetric cavities generated can be leveraged for drug delivery and release, catalysis and sensing.

## Author contributions

Authorship is alphabetical between B. E. B., E. M. G. J., and L. E. M. W. Conceptualisation, B. E. B., E. M. G. J., L. E. M. W., and C. T. M.; formal analysis, B. E. B., E. M. G. J., and L. E. M. W.; investigation, B. E. B., E. M. G. J., and L. E. M. W.; resources, C. T. M.; writing – original draft, B. E. B., E. M. G. J., L. E. M. W.; writing – review & editing; B. E. B., E. M. G. J., L. E. M. W., and C. T. M.; visualisation, B. E. B., E. M. G. J., L. E. M. W., and C. T. M; supervision; C. T. M.; project administration, C. T. M.; funding acquisition, C. T. M.

## Conflicts of interest

There are no conflicts to declare.

## Supplementary Material

SC-OLF-D5SC06441D-s001

## Data Availability

The data supporting this article have been included as part of the supplementary information (SI). Supplementary information is available. See DOI: https://doi.org/10.1039/d5sc06441d.
