# Supplementary material for: Peptide ligand isomerism drives divergent stability and guest binding in Pd3L4 metal-peptidic cages
Source: Chem Sci. 2025 Dec 9;17(6):3148–56. doi: 10.1039/d5sc06441d (PMC12705214; doi:10.1039/d5sc06441d)
Supplement: SC-017-D5SC06441D-s001 [file SC-017-D5SC06441D-s001.pdf]

Supplementary Information (SI) for Chemical Science.
This journal is © The Royal Society of Chemistry 2025

Supplementary Information

Peptide Ligand Isomerism Drives Divergent Stability and Guest Binding in Pd_3L_4 Metal-Peptidic Cages

Ben E. Barber,[1,2,3] Ellen M. G. Jamieson,[1,2,3] Leah E. M. White,[1,2,3] and Charlie T. McTernan[1,2,*]

[1]Artificial Molecular Machinery Laboratory, The Francis Crick Institute, 1 Midland Road, London NW1 1AT, UK.
[2]Department of Chemistry, King's College London, Britannia House, 7 Trinity Street, London SE1 1DB, UK.
[3]Authorship is alphabetical.
[*]Corresponding Author

Contents

1. General Experimental **3**

2. General Procedures **5**
General SPPS Procedure *5*
General Ester Coupling *6*
General Cage Synthesis *6*

3. Synthesis of Peptide Rods & Precursors **7**
Pyr-Peptide Rod $\mathbf{L}^{7,R}$ *8*
Pyr-Peptide Rod $\mathbf{L}^{4,R}$ *14*
Pyr-Peptide Rod $\mathbf{L}^{7,S}$ *20*
Pyr-Peptide Rod $\mathbf{L}^{4,S}$ *26*

4. Synthesis of Metal Peptidic Cages **32**
Metal-Peptidic Cage $\mathbf{Pd_3L^{7,R}_4}(BF_4)_6$ *33*
Metal-Peptidic Cage $\mathbf{Pd_3L^{4,R}_4}(BF_4)_6$ *40*
Metal-Peptidic Cage $[\mathbf{Pd_3L^{7,S}_4}(BF_4)_6]_2$ *50*
Metal-Peptidic Cage $\mathbf{Pd_3L^{4,S}_4}(BF_4)_6$ *59*

5. Cage Titrations **66**

6. DOSY Analysis **72**

7. Cage Stabilities **78**
Dilution Study *83*
Acid Stability Studies *93*
Base Stability Studies *101*
Pyridine Stability Studies *109*
Glutathione Stability Studies *121*

8. Ion Mobility Mass Spectrometry **129**

9. CD Spectra Comparison **131**

10. Isomerisation Discussion **138**
Cage $\mathbf{Pd_3L^{7,R}_4}(BF_4)_6$ *140*
Cage $\mathbf{Pd_3L^{4,R}_4}(BF_4)_6$ *142*
Cage $[\mathbf{Pd_3L^{7,S}_4}(BF_4)_6]_2$ *145*
Cage $\mathbf{Pd_3L^{4,S}_4}(BF_4)_6$ *147*
Assembly in d_3*-MeCN* *153*

11. Host-Guest Studies **157**

12. Molecular Models **181**

13. References **190**

1. General Experimental

Synthesis: Unless otherwise stated, all reagents, including anhydrous solvents, were purchased from commercial sources and used without further purification. Reactions were carried out under ambient conditions unless otherwise stated. Solid phase peptide synthesis was carried out on a CEM Liberty Blue Microwave Peptide Synthesiser; an Eppendorf Centrifuge 5910 Ri was used for centrifugation; a SP Scientific Bench Top Pro Lyophiliser was used for drying. Preparative HPLC was performed on a Waters LC Prep AutoPurification System. Chromatographic separation was performed on an XSelect CSH C18 OBD Prep Column (130 Å, 5 μm, 19 x 150 mm). The instrument was operated at a flow rate 25 mL min^{-1} with an injection volume of 750 µL.

Analysis: NMR spectra were acquired on a Bruker Ascend 400 equipped with a BBO Smart Probe, an Avance III 600 equipped with a $^{1}H/^{13}C/^{15}N$ triple-resonance PFG cryoprobe, a Bruker Avance NEO 600 MHz equipped with a TCI Cryoprobe Prodigy $^{1}H/^{13}C/^{15}N$ a Bruker Avance III 700 MHz equipped with a QCI Cryoprobe $^{1}H/^{13}C/^{15}N/^{31}P$ or an Avance III HD 950 equipped with a $^{1}H/^{13}C/^{15}N$ triple-resonance PFG cryoprobe at a constant temperature of 298 K unless stated otherwise. Chemical shifts are reported in parts per million from low field to high field, and referenced to residual solvent or internal standards. Coupling constants (*J*) are reported in Hertz (Hz), and standard abbreviations indicating multiplicity were used as follows: m = multiplet, sext = sextet, quint = quintet, q = quartet, t = triplet, d = doublet, s = singlet, app = apparent and br = broad. Signal assignment was carried out using 2D NMR methods (HSQC, HMBC, COSY, NOESY, ROESY) where necessary. J-Modulated Spin-Acho ^{13}C NMR spectra were acquired an Avance III 600 using the standard Bruker "jmod" pulse sequence. ^{1}H DOSY NMR spectra were acquired on an Avance III 600 using the pulse sequence "ledbpgp2s". Data were collected using 32 gradient strengths with a relaxation delay of 2 s. Data were processed in MestReNova using Bayesian method, resolution factor 100 and repetitions 8, to obtain diffusion coefficients. Diffusion coefficients were subsequently used to calculate hydrodynamic radii using the Stokes-Einstein equation:

Equation S1.

$$r = \frac{k_B T}{6\pi\eta D}$$

Where r = hydrodynamic radius, k_B = Boltzmann constant, T = temperature, η = medium viscosity and D = diffusion coefficient.

Low resolution mass spectrometry and UPLC analysis was performed on a Waters Acquity H-class UPLC coupled with a Waters SQD2 mass spectrometer. Chromatographic separation was performed on an ACQUITY UPLC BEH C18 column (130 Å, 1.7 μm, 2.1 x 50 mm) with the following conditions: Solvent A: H_2O:MeCN (95:5) + 2 mM ammonium acetate; Solvent B: MeCN; gradient of 0 – 100% B over 2 min. The instrument was operated at a flow rate of 0.8 mL min^{-1} with a column temperature of 45 °C and an injection volume of 5 µL.

ESI-HRMS were recorded on an Orbitrap Exploris 120 mass spectrometer. The ESI source was operated in positive-negative switching mode at spray voltages of 3500 and -2500 V in the positive and negative modes, respectively. Sheath gas was set to 35, auxiliary gas to 7 and sweep gas to 1. The ion transfer tube temperature was set to 280 °C and the vaporiser temperature was set to 250 °C. A mass range of 400 – 3000 Da was scanned at a resolution of 120,000. Unless otherwise stated, all solutions used for ESI-HRMS analysis were diluted with H_2O to ~ 200 μM.

Circular dichroism (CD) spectra were recorded on a JASCO J-815 CD Spectropolarimeter equipped with a JASCO CDF-426S/15 Peltier temperature controller using quartz cells (Hellma) of 0.1, 0.2 and 1 mm pathlength in H_2O at 273 K unless otherwise stated. The scans were recorded from 190 – 400 nm with the following parameters: 0.2 nm data pitch, 2 nm bandwidth, 200 nm min^{-1} scanning speed and are the sum of 25 accumulations. The background corresponding to the solvent and cuvette's absorption was recorded and subtracted from sample measurements and all spectra were smoothed using the Savitzky-Golay method with convolution width of 15.

Ion mobility studies were performed by Warwick AMS RTP on a commercial basis. Ionisation was by direct infusion in positive ionisation mode using a custom nanospray ion source, using pulled a glass capillary. NanoESI capillary voltage applied: 1500 V. Dry gas flow rate: 4.0 L/min. Dry temperature: 200 °C. Carrier gas: N_2. TIMS temperature: 305 K. Entrance pressure: 2.67 mbar. Exit pressure: 0.97 mbar. IMS Ramp Start: 0.3 V·s/cm^2. IMS Ramp End: 1.5 V·s/cm^2. Standard MS spectra and Ion Mobility measurements were acquired using timsTOF Pro (mass range of m/z 400 – 3000). All mass spectra were calibrated by a quadratic calibration function, and all ion mobility spectra were calibrated by a linear function, using commercially available low concentration tuning mix from Agilent. Reduced mobilities were converted into collision cross-sections (CCS) using the Mason-Schamp equation.[S1]

2. General Procedures

All solid phase peptide synthesiser (SPPS) reactions were run on a 0.167 mmol scale, and general procedures are written at this scale. All steps were performed with no isolation or purification of intermediates unless specifically stated.

Resin loading measurement was performed according to literature procedures, and the average loading found to be 0.017 mmol g^{-1}.[S2]

a. *General SPPS Procedure*

Stock solutions were prepared to the following specifications: Fmoc-Pro-OH (0.2 M), Fmoc-(2*S*,4*R*)-Hyp(^{t}Bu)-OH (0.2 M), Fmoc-(2*S*,4*S*)-Hyp(^{t}Bu)-OH (0.1 M), ethyl cyano(hydroxyimino)acetate (1.0 M), and *N*,*N′*-diisopropylcarbodiimide (1.0 M) in DMF. Fmoc deprotection was carried out by addition of 10 mL of 20% (v/v) piperidine in DMF for 50 s, temperature 90 °C and microwave power 30 W, and the resin washed with DMF (4 x 7 mL) and drained. 5.0 mL of amino acid solution was added to the reaction vessel, followed by 2.0 mL 1.0 M *N*,*N′*-diisopropylcarbodiimide and 1.0 mL 1.0 M ethyl cyano(hydroxyimino)acetate. For each amino acid, the following coupling conditions were used:

	Temperature (°C)	Microwave Power (W)	Hold Time (s)
Fmoc-Pro-OH	90	30	120
Fmoc-(2S,4R)-Hyp(^{t}Bu)-OH	50	60	480
Fmoc-(2S,4S)-Hyp(^{t}Bu)-OH	90	40	1800

Table S1. SPPS coupling conditions for different amino acids.

The reaction vessel was drained and washed with DMF (4 x 7 mL).

Capping Protocol

Following SPPS, the resin was transferred to a falcon tube and a solution of pivalic anhydride in 1:1 CH_2Cl_2:DMF (10 mL, 10% (v/v)) was added. The mixture was shaken for 45 mins, filtered, and the resin washed with CH_2Cl_2 (10 x 5 mL).

Resin Cleavage and Global Deprotection

The resin was transferred to a falcon tube and treated with a cleavage mixture (10 mL, v/v, TFA:Triisopropyl silane:H_2O 38:1:1). The slurry was shaken for 2 hours, filtered and the resin washed with further cleavage mixture (5 mL). The filtrate was reduced *in vacuo* to a volume of ~2 – 5 mL. Diethyl ether (40 mL) was added to yield a white precipitate which was separated *via* centrifugation (3000 rpm, 10 mins), followed by decanting the solvent from the pellet. The pellet was then resuspended in diethyl ether (10 mL), sonicated, and then re-centrifuged (3000 rpm, 10 mins) twice. After the final

trituration, the solvent was decanted and the pellet allowed to air dry. Desired peptide formation was confirmed by LCMS.

b. General Ester Coupling

Crude peptide residue was used after resin cleavage with no purification.

Crude peptide residue (assumed 0.167 mmol, 100% yield, based on SPPS synthesis), DMAP (61.1 mg, 0.5 mmol), isonicotinic acid (123.1 mg, 1.00 mmol) and EDC.HCl (191.7 mg, 1.00 mmol) were dissolved in CH_2Cl_2 (12.5 mL) in a sealed microwave vial under an inert atmosphere. The reaction mixture was then stirred for 16 hours, turning to an orange/brown solution. The mixture was diluted in $CHCl_3$:isopropyl alcohol (3:1 v/v, 30 mL) and washed with H_2O (3 x 30 mL). The aqueous phase was re-extracted with $CHCl_3$:isopropyl alcohol (3:1 v/v, 3 x 30 mL), and the combined organic phases were then washed with brine (30 mL), dried ($MgSO_4$) and concentrated *in vacuo* to yield a brown oily crude residue.

c. General Cage Synthesis

As previously reported,[S3] cage formation was found to be highly sensitive to equivalencies of metal salt.

To synthesise a sample of cage at concentration of *n*, a solution of [Pyr-Proline Rod] in D_2O was prepared to approximately 8*n* mM the desired cage concentration. The exact concentration was then determined by comparison with a known standard of DSS, then a solution of the desired palladium salt, [PdX], in D_2O would be prepared at 4n mM. Approximately 2.2 eq of [PdX] solution was added and the mixture checked by NMR. Provided the relative Cage:Ligand ratio was as expected, the subsequent 0.8 eq PdX solution would be added, yielding a solution of Cage at *n* mM.

3. Synthesis of Peptide Rods & Precursors

Pyridine functionalised proline rods undergo ester hydrolysis in D_2O at room temperature, and so while care was taken to limit time in solution prior to obtaining full characterisation, there is unavoidably a small amount of hydrolysis present that is not visible by LCMS or in samples run immediately on dissolution. Internal proline residues along the oligoproline rods resolve as overlapping multiplets so they have been given one label for the NMR assignments to reduce confusion. The five membered ring systems showed diastereotopic splitting of many of the protons, often leading to their resolving separately. Where this has occurred, the first proton will be labelled as H_{α} and the second as $H_{\alpha'}$, assigned arbitrarily based on which signal is at the higher ppm.

As discussed in the manuscript, PPII folding is destabilised when using *4S*-hydroxyproline, instead of the naturally occurring 4*R*-hydroxyproline, and they adopt some degree of PPI folding by *cis-trans* isomerism of the amide backbone bonds.[S4] In systems which show greater amounts of PPI this can be observed by NMR. We verify the purify of these samples by their LCMS-HPLC traces.

Pyr-Peptide Rod **L**^{7,R}

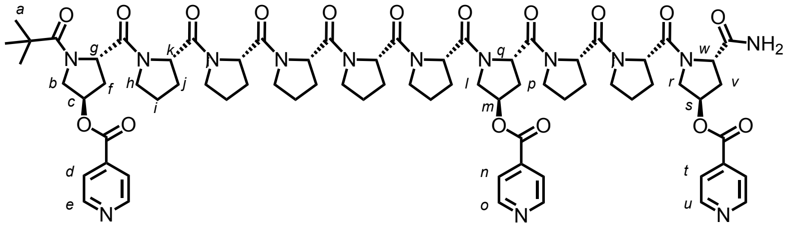

L^{7,R} was prepared by following the **General SPPS Procedure**, then the crude residue was submitted to the **General Coupling Procedure** without purification. Preparative HPLC, using a gradient of 10 – 40% MeCN in H_2O, which was then dried by lyophilisation, yielded **L**^{7,R} as a white powder (60.2 mg, 51% overall yield).

^{1}H NMR (600 MHz, D_2O) δ 8.74 (app. dd, 4H, 4 of H_e, H_o or H_u), 8.67 (d, *J* = 6.3 Hz, 2H, 2 of H_e, H_o or H_u), 7.95 (app. d, 4H, 4 of H_d, H_n or H_t), 7.92 (d, *J* = 6.3 Hz, 2H, 2 of H_d, H_n or H_t), 5.75 (br s, 2H, H_c and H_s), 5.60 (br s, 1H, H_m), 4.95 (app. q, 2H, H_g and H_q), 4.79 – 4.67 (m, 8H H_k and H_w), 4.37 (app. t, 2H, H_b and H_r) 4.22 (d, *J* = 12.6 Hz, 1H, H_l), 4.07 (dd, *J* = 12.4, 4.0 Hz, 1H, $H_{r'}$), 4.02 (dd, *J* = 12.8, 3.7 Hz, 1H, $H_{b'}$), 3.99 – 3.93 (m, 1H, $H_{l'}$), 3.90 – 3.78 (m, 7H, 7 of H_h), 3.70 – 3.58 (m, 5H, 5 of H_h), 3.51 – 3.46 (m, 2H, 2 of H_h), 2.71 (app. quin, 2H, Hf and H_v), 2.54 (dd, *J* = 13.9, 8.9 Hz, 1H, H_p), 2.38 – 2.29 (m, 7H, 6 of H_j, and $H_{v'}$), 2.23 – 2.19 (m, 1H, 1 of H_j), 2.17 – 2.00 (m, 14H, $H_{f'}$, H_p, 12 of H_i and H_j) 1.97 – 1.86 (m, 8H, 8 of H_i and H_j), 1.72 (app. h, 1H, 1 of H_j), and 1.25 (s, 9H, H_a). ^{13}C NMR (151 MHz, D_2O) δ 180.2, 175.7, 172.9, 172.1, 171.8, 171.6, 171.5, 171.5, 171.5, 171.4, 170.6, 165.7, 165.7, 149.7, 149.7, 149.6, 137.8, 137.6, 123.5, 123.3, 76.3, 75.3, 74.9, 59.0, 58.8, 58.7, 58.7, 58.5, 58.5, 58.4, 58.3, 57.1, 54.0, 53.1, 52.4, 47.7, 47.7, 47.6, 47.5, 47.4, 38.3, 34.8, 33.1, 32.3, 28.1, 27.9, 27.9, 27.8, 27.8, 26.2, 24.7, 24.5, 24.5, and 24.4. ESI-HRMS: m/z observed 718.3337 [**L**^{7,R} + 2H]$^{2+}$, calc. 718.3377, (Figure S10).

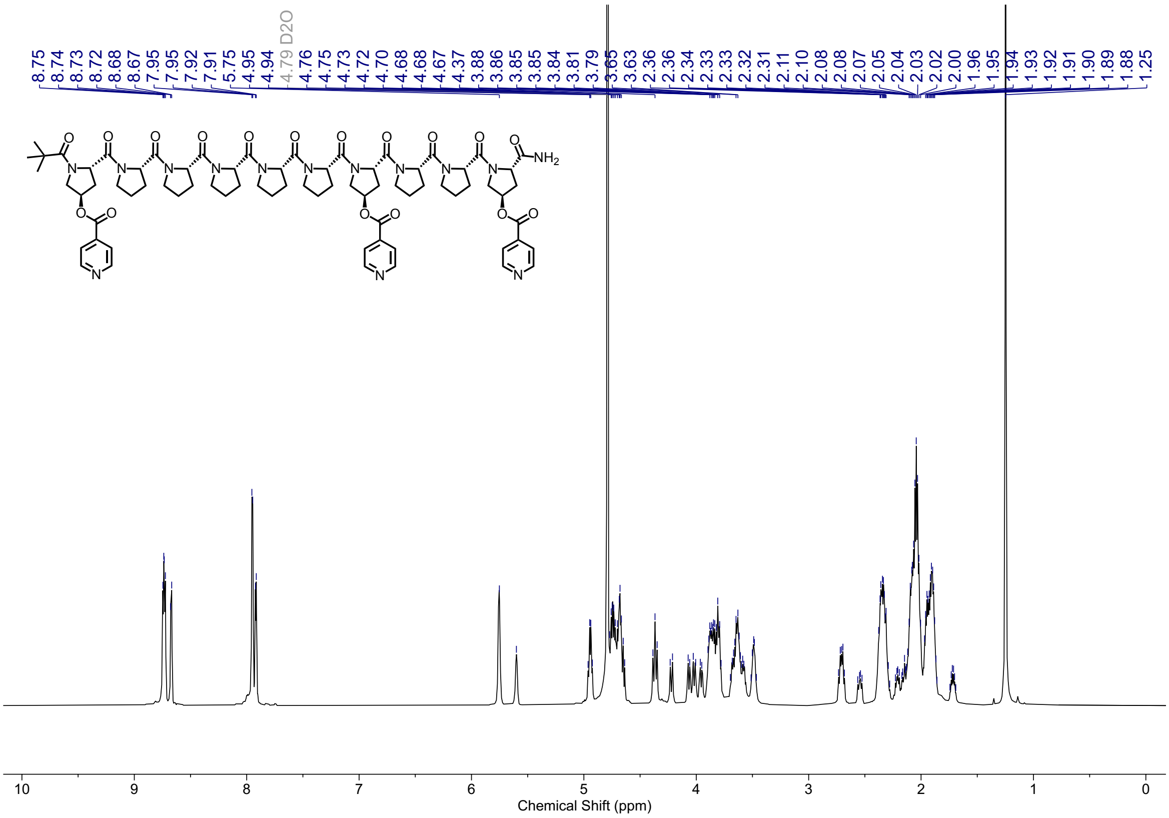

Figure S1. ^{1}H NMR (D_2O, 600 MHz, 298 K) of $\mathbf{L}^{7,R}$.

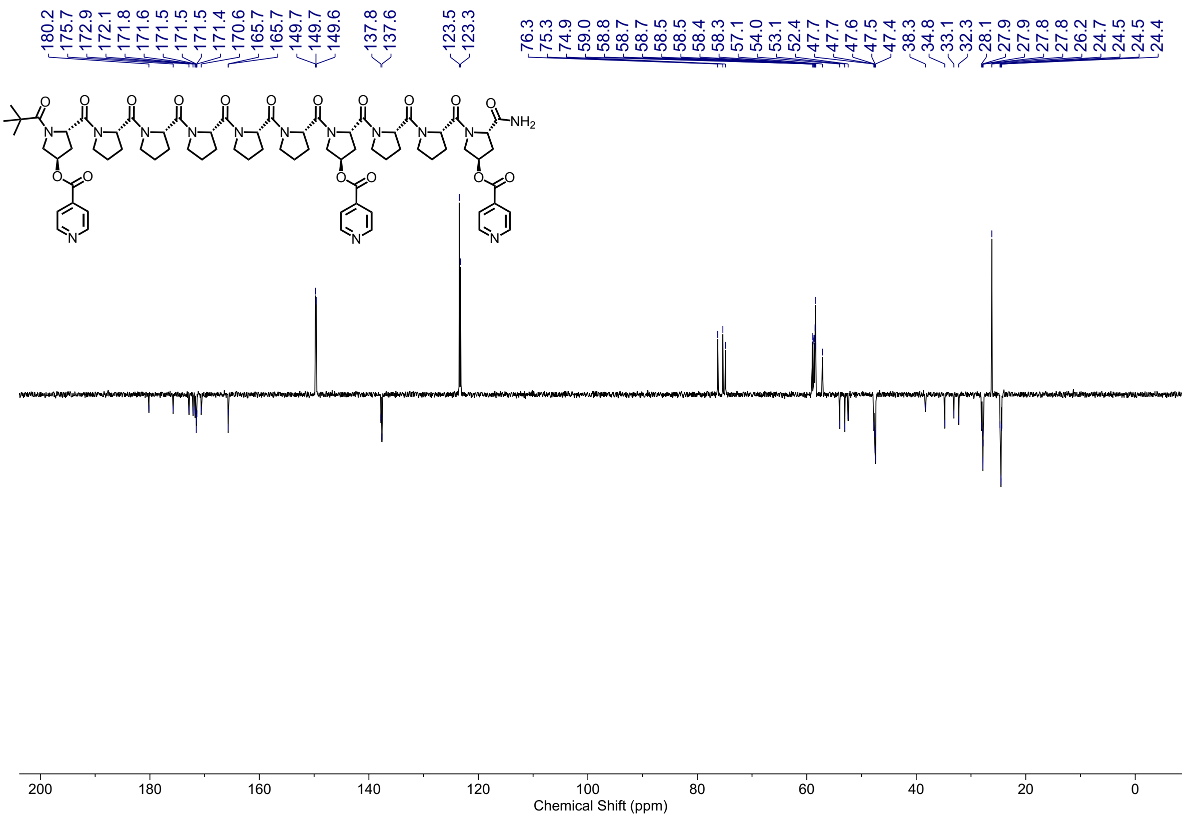

Figure S2. ^{13}C JMOD (D_2O, 151 MHz, 298 K) of $\mathbf{L}^{7,R}$.

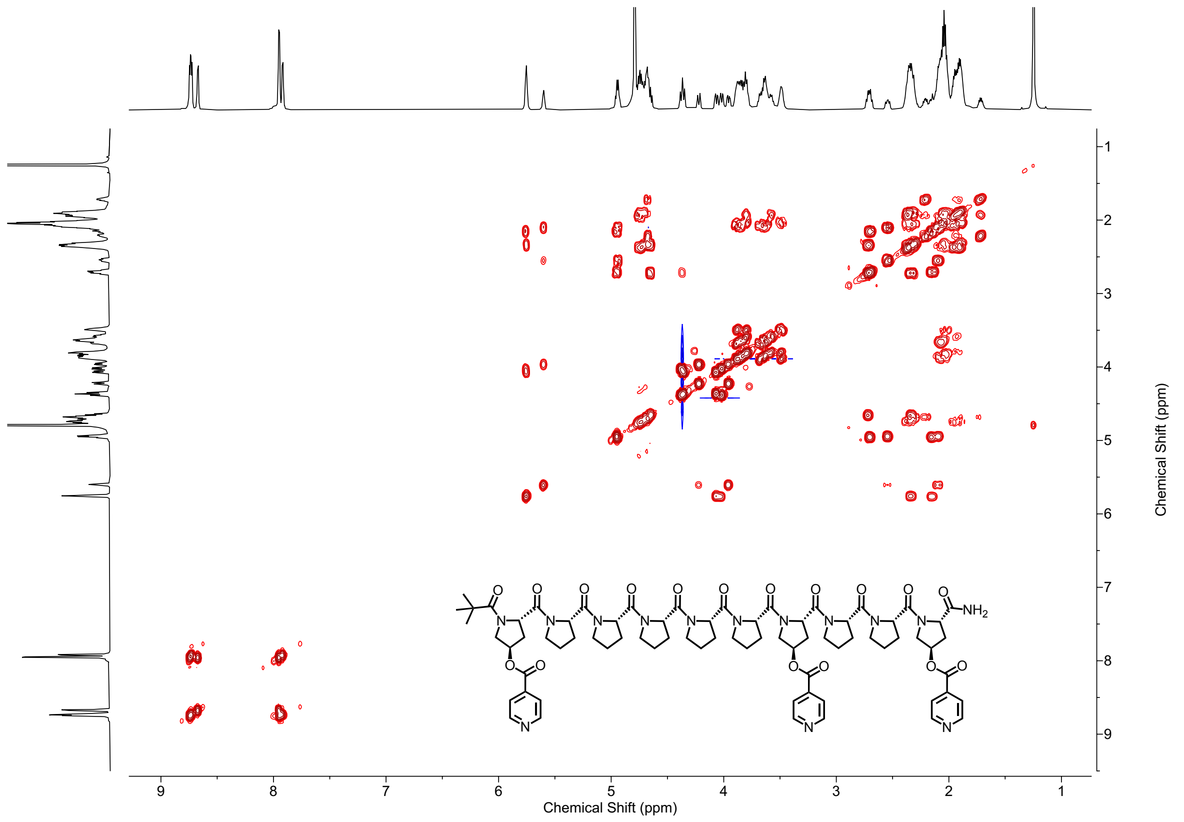

Figure S3. ^{1}H-^{1}H COSY (D_2O, 600 MHz, 298 K) of **L**^{7,R}.

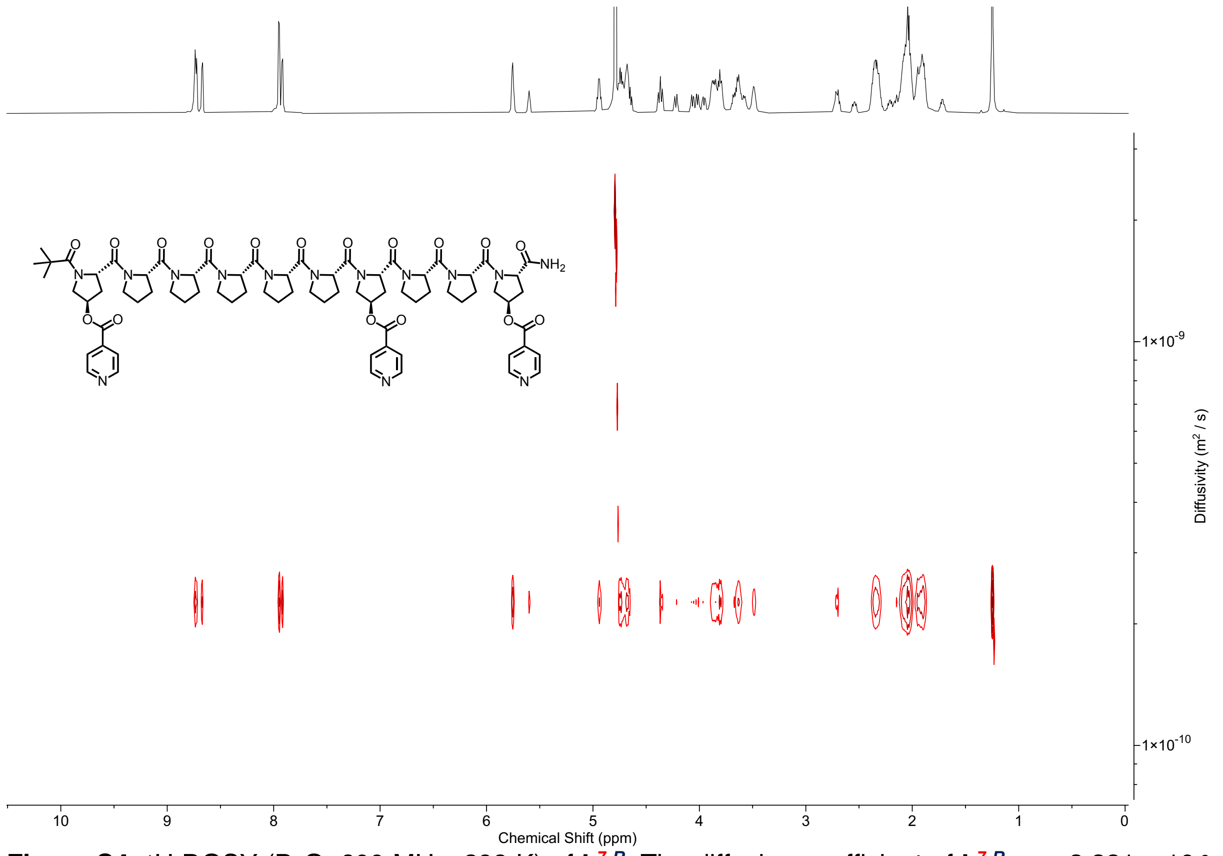

Figure S4. ^{1}H DOSY (D_2O, 600 MHz, 298 K) of **L**^{7,R}. The diffusion coefficient of **L**^{7,R} was 2.221 x 10^{-9} m^2 s^{-1}, giving a hydrodynamic radius of 14.7 Å.

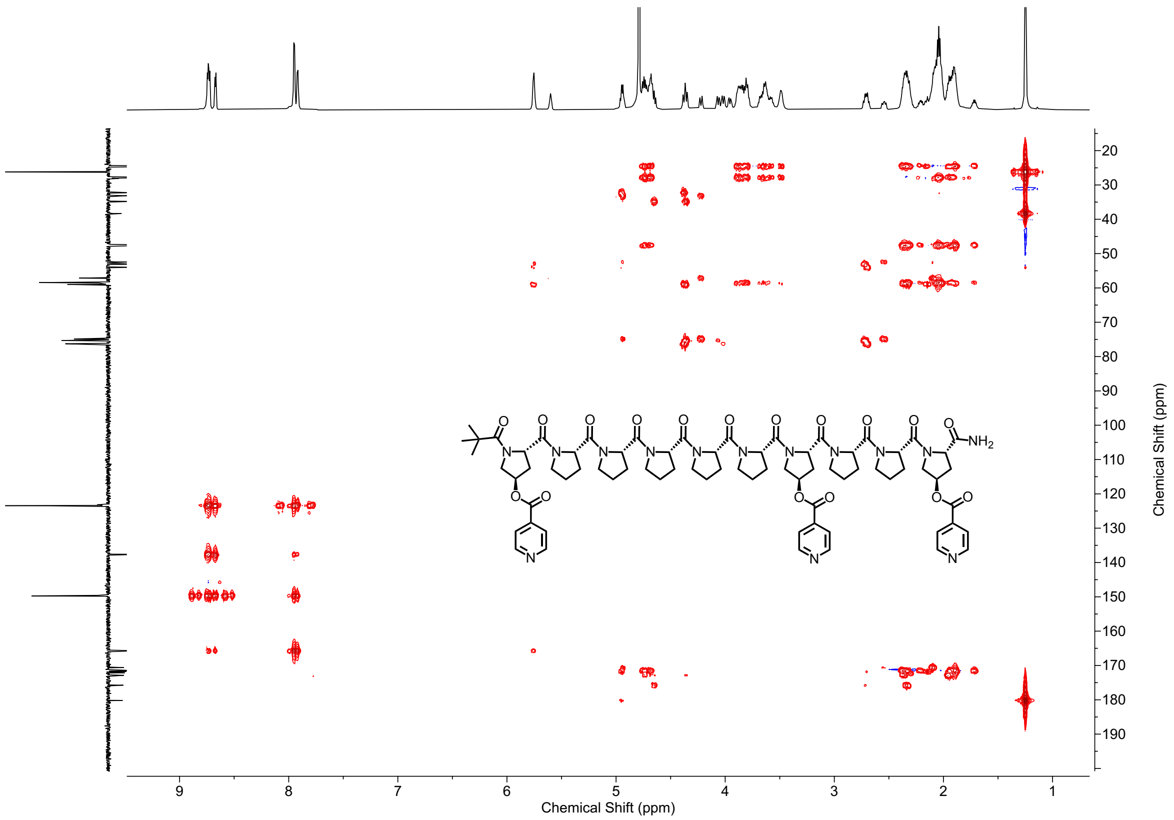

Figure S5. ^{1}H-^{13}C HMBC (D_2O, 600 MHz, 298 K) of $\mathbf{L}^{7,R}$.

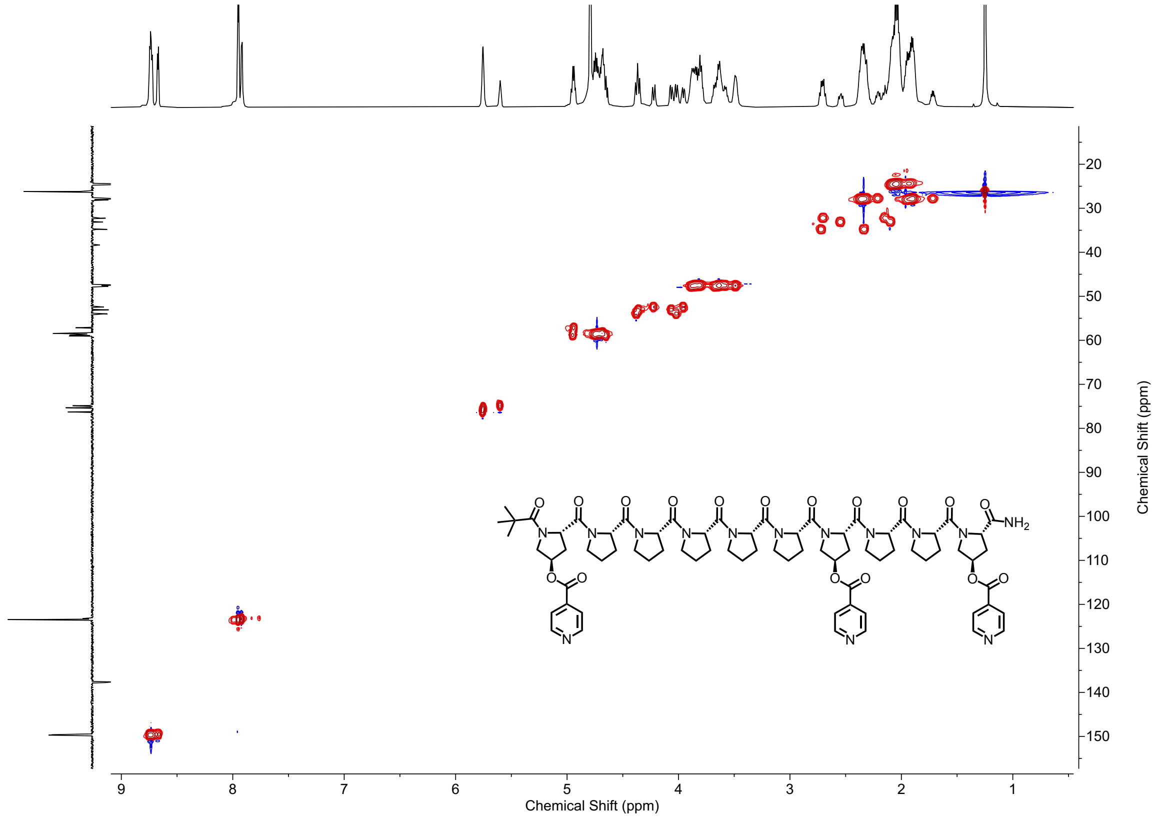

Figure S6. ^{1}H-^{13}C HSQC (D_2O, 600 MHz, 298 K) of $\mathbf{L}^{7,R}$.

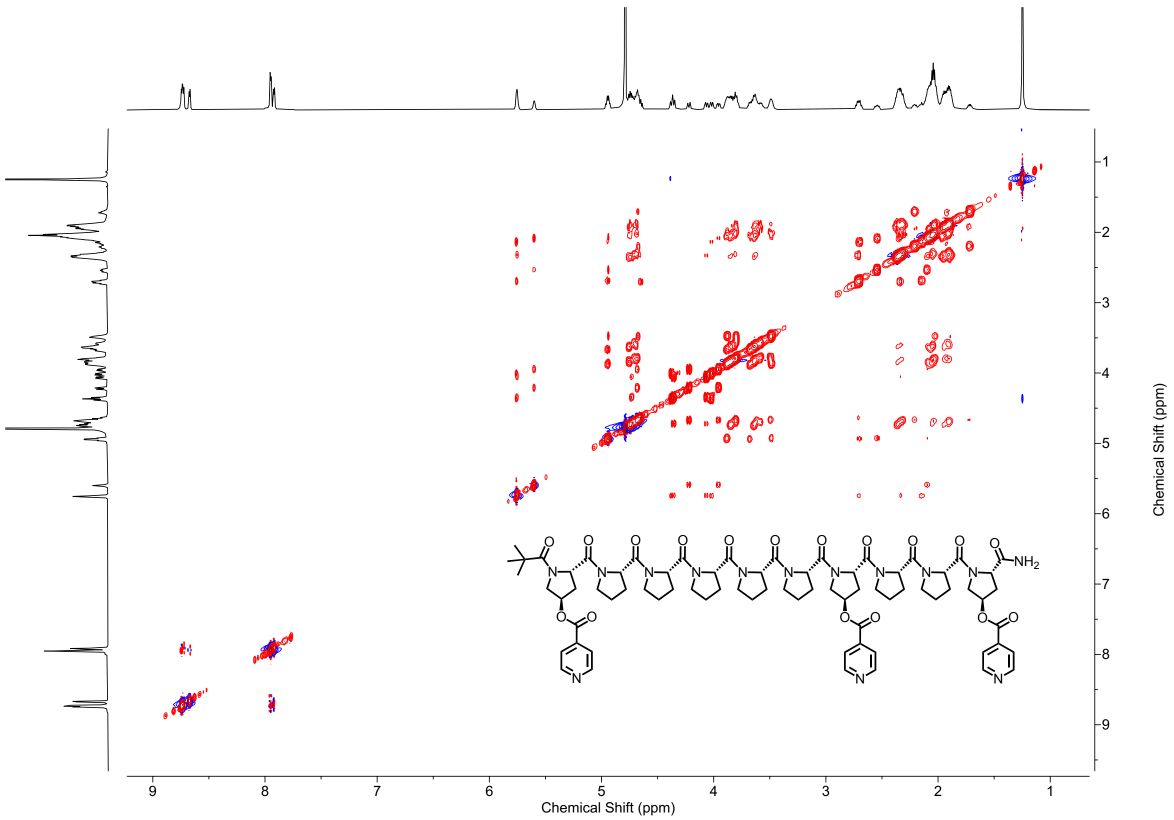

Figure S7. ^{1}H-^{1}H NOESY (D_2O, 600 MHz, 298 K, Mixing time = 0.300 s) of $\mathbf{L}^{7,R}$.

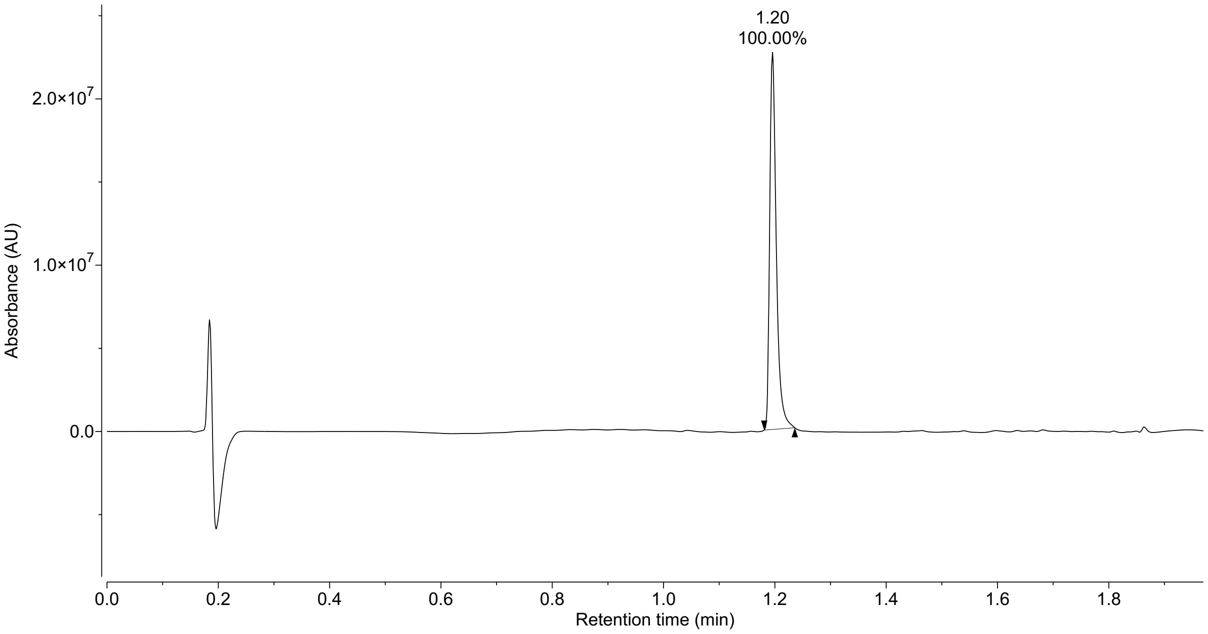

Figure S8. UPLC chromatogram of $\mathbf{L}^{7,R}$ using general solvent system.

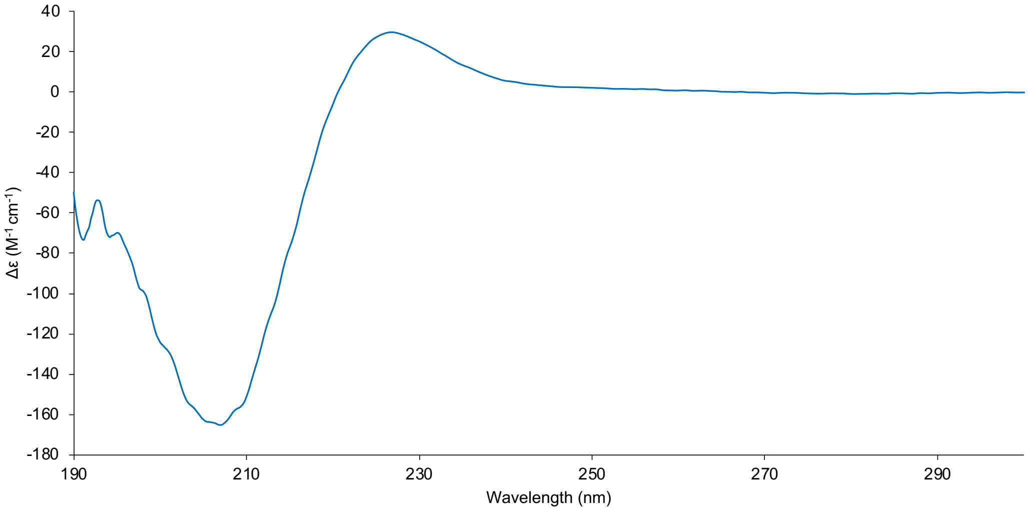

Figure S9. CD spectrum of **L**^{7,R} in H_2O (293 K, 0.15 mM, 1 mm). Minima and maxima observed at 207 nm and 227 nm consistent with folding to the PPII helix.

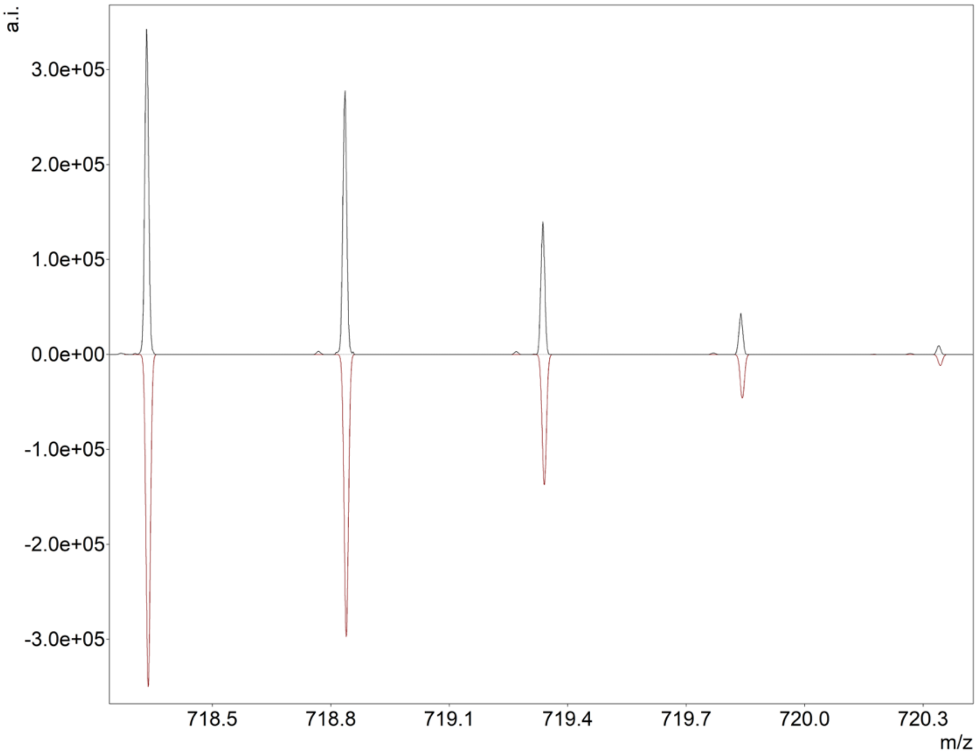

Figure S10. HRMS experimental (top) and calculated (bottom) isotope patterns of [**L**^{7,R} + 2H]$^{2+}$.

Pyr-Peptide Rod **L**^{4,R}

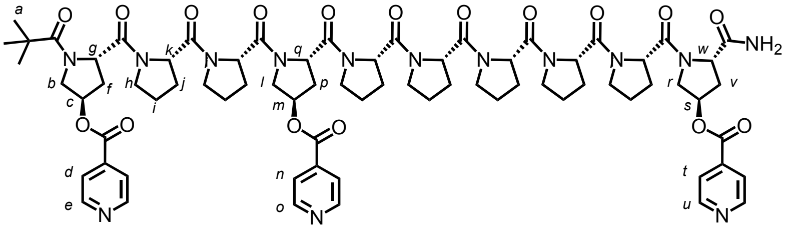

L^{4,R} was prepared by following the **General SPPS Procedure**, then the crude residue was submitted to the **General Coupling Procedure**. Preparative HPLC, using a gradient of 10 – 40% MeCN in H_2O, which was then concentrated by lyophilisation, yielded **L**^{4,R} as a white powder (56.0 mg, 47% overall yield).

^{1}H NMR (600 MHz, D_2O) δ 8.74 – 8.73 (app. t, 4H, H_e and H_u), 8.70 (d, J = 6.2 Hz, 2H, H_o), 7.99 (d, J = 5.4 Hz, 2H, H_t), 7.97 (d, J = 6.2 Hz, 2H, H_n), 7.89 (d, J = 5.3 Hz, 2H, 2 of H_d), 5.75 (app. d, 2H, H_m and H_s), 5.64 (br s, 1H, H_m), 4.98 (t, J = 8.5 Hz, 1H, H_q), 4.90 (t, J = 8.5 Hz, 1H, H_g) 4.77 – 4.65 (m, 8H, H_k and H_w), 4.35 – 4.33 (app. d, 2H, H_l, H_r), 4.30 (d, J = 12.6 Hz, 1H, H_b), 4.06 – 3.98 (m, 3H, $H_{b'}$, $H_{l'}$ and $H_{r'}$), 3.94 – 3.77 (m, 7H, H_h), 3.67 – 3.42 (m, 7H, $H_{h'}$), 2.80 – 2.71 (m, 2H, H_p and H_v), 2.48 (dd, J = 14.2, 8.1 Hz, 1H, H_f), 2.39 – 2.24 (m, 8H, $H_{p'}$, $H_{v'}$, 6 of H_j), 2.21 – 1.85 (m, 22H, Hf', 21 of H_i and H_j), 1.67 (h, J = 6.5 Hz, 1H, 1 of H_j), and 1.23 (s, 9H, H_a). ^{13}C NMR (151 MHz, D_2O) δ 180.1, 175.8, 172.8, 172.2, 171.6, 171.6, 171.5, 171.5, 171.5, 171.4, 170.7, 165.8, 165.6, 149.7, 149.6, 137.8, 137.5, 123.5, 123.5, 123.2, 75.8, 75.5, 75.3, 59.0, 58.8, 58.7, 58.6, 58.5, 58.5, 58.4, 58.4, 57.3, 53.7, 53.0, 52.9, 47.8, 47.7, 47.6, 47.5, 47.4, 38.3, 34.7, 33.5, 32.1, 28.1, 27.9, 27.9, 27.9, 27.8, 27.8, 26.2, 24.6, 24.6, 24.5, 24.5, and 24.4. ESI-HRMS: m/z observed 718.3371 [**L**^{4,R} + 2H]$^{2+}$, calc. 718.3377, (Figure S20).

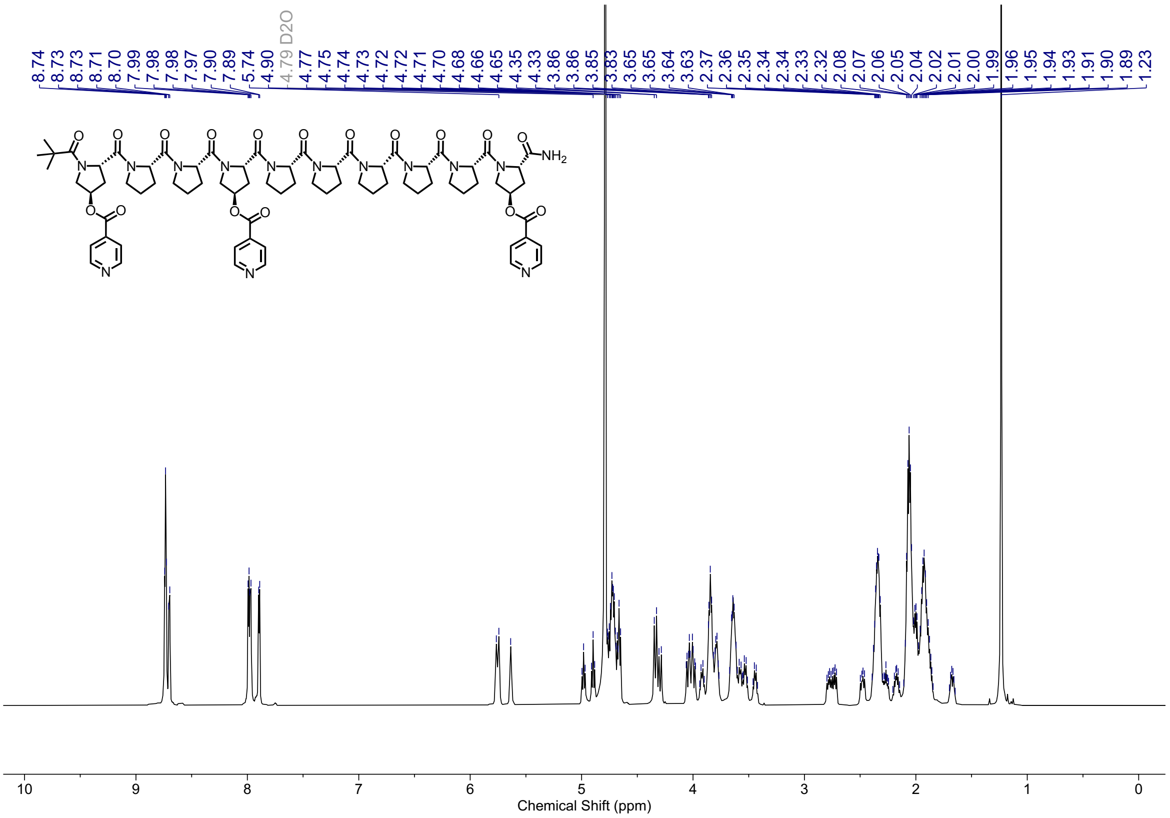

Figure S11. ^{1}H NMR (D_2O, 600 MHz, 298 K) of $\mathbf{L}^{4,R}$.

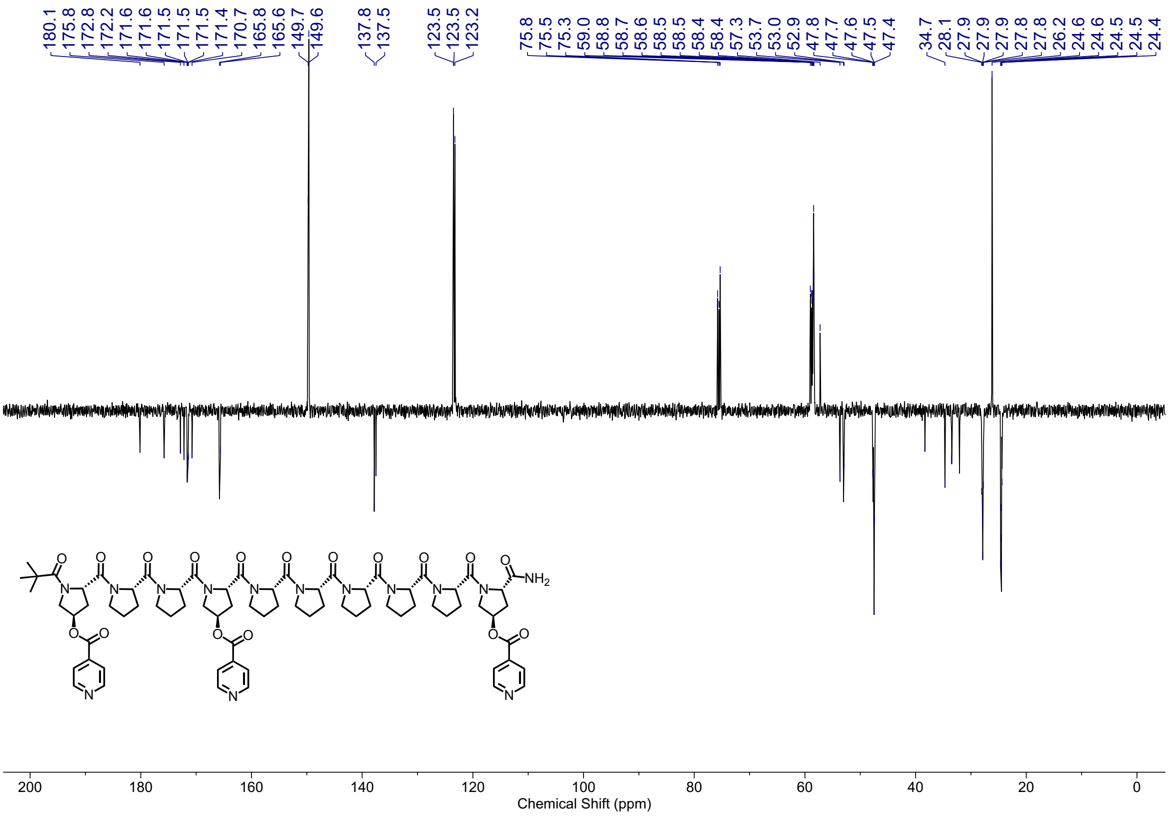

Figure S12. ^{13}C JMOD (D_2O, 151 MHz, 298 K) of $\mathbf{L}^{4,R}$.

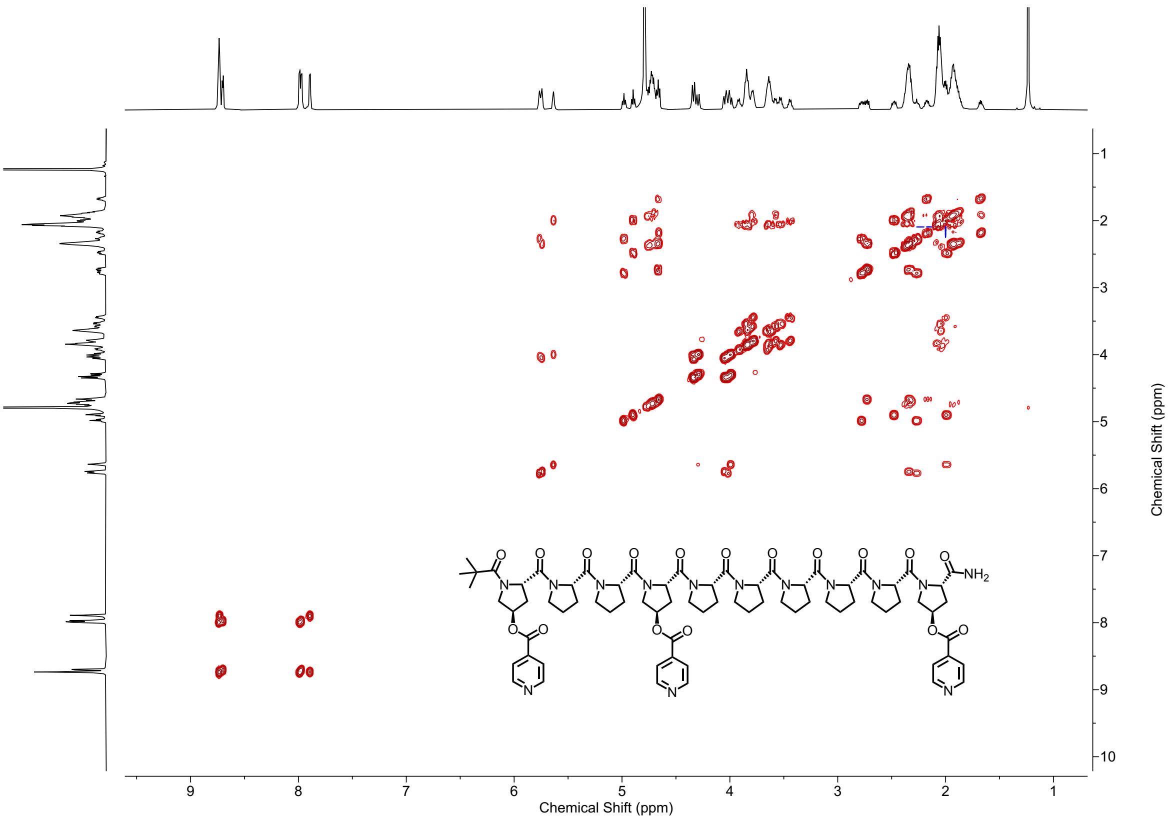

Figure S13. ^{1}H-^{1}H COSY (D_2O, 600 MHz, 298 K) of **L**^{4,R}.

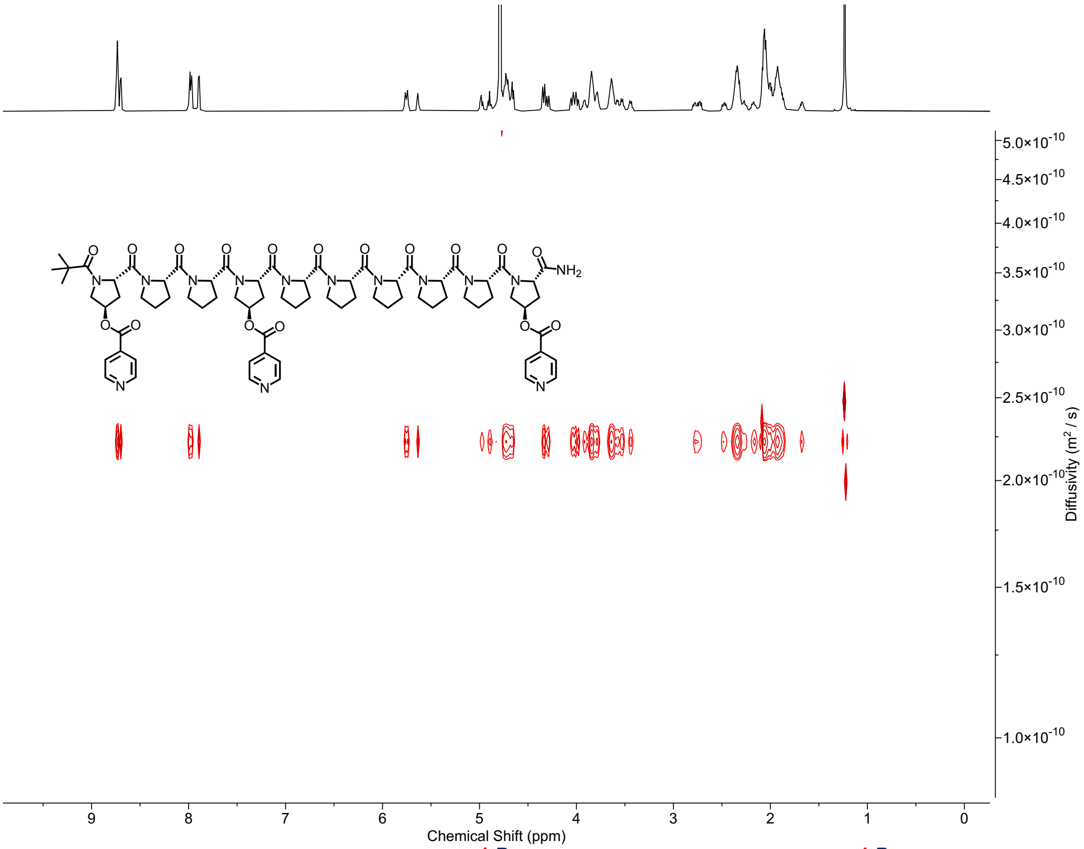

Figure S14. ^{1}H DOSY (D_2O, 600 MHz, 298 K) of **L**^{4,R}. The diffusion coefficient of **L**^{4,R} was 2.221 x 10^{-9} m^2 s^{-1}, giving a hydrodynamic radius of 14.7 Å.

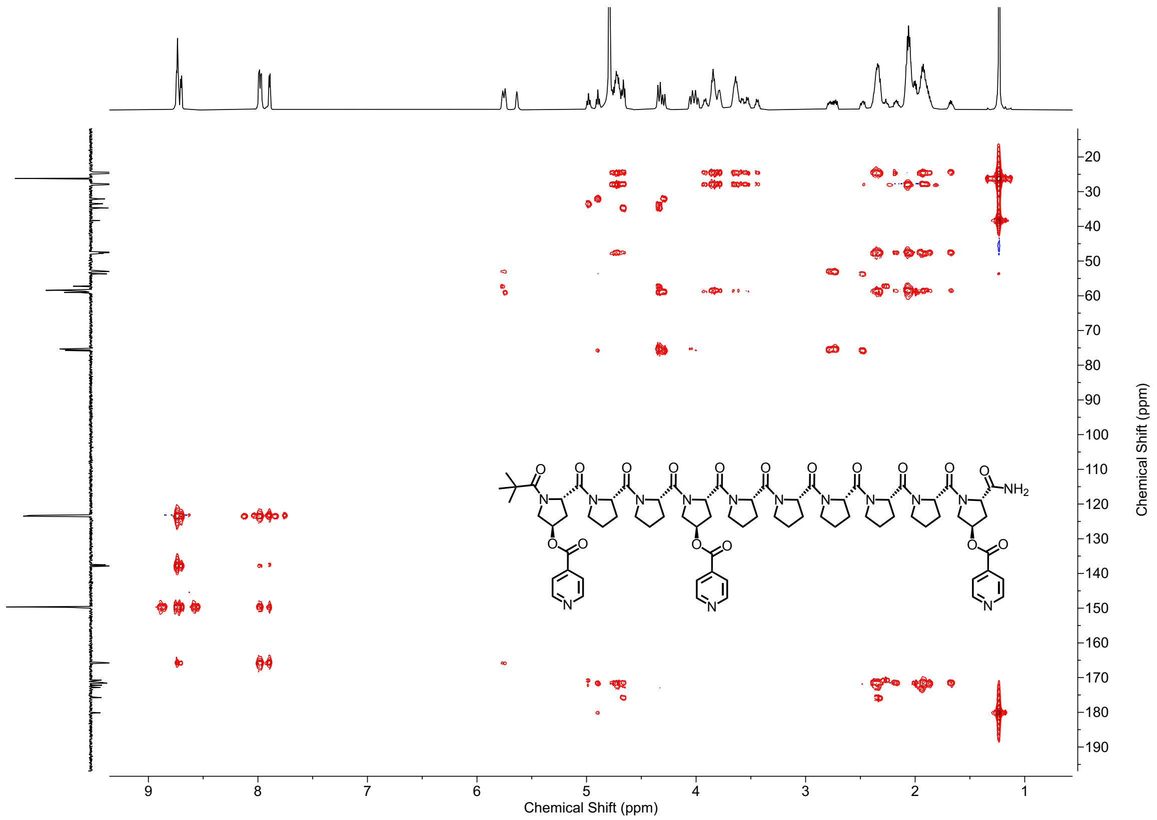

Figure S15. ^{1}H-^{13}C HMBC (D_2O, 600 MHz, 298 K) of **L**^{4,R}.

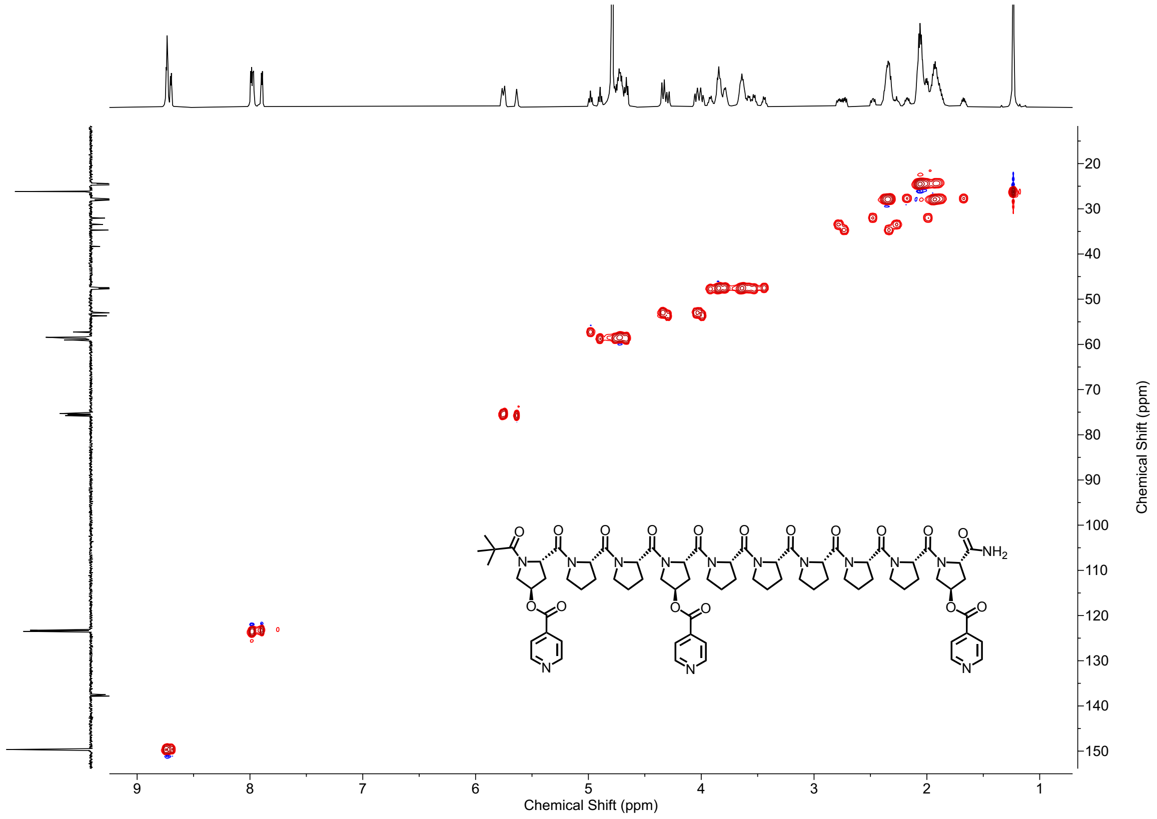

Figure S16. ^{1}H-^{13}C HSQC (D_2O, 600 MHz, 298 K) of **3**.

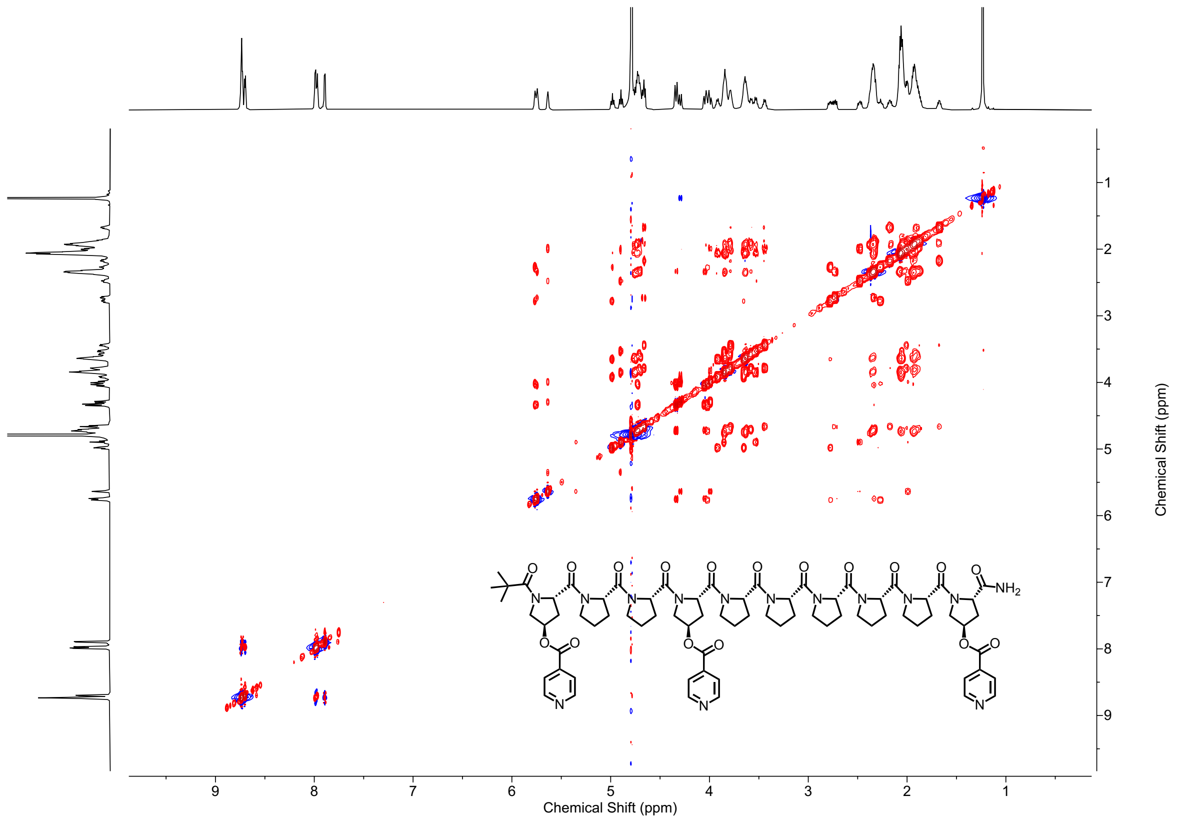

Figure S17. ^1H-^1H NOESY (D_2O, 600 MHz, 298 K, Mixing time = 0.300 s) of $\mathbf{L}^{4,R}$.

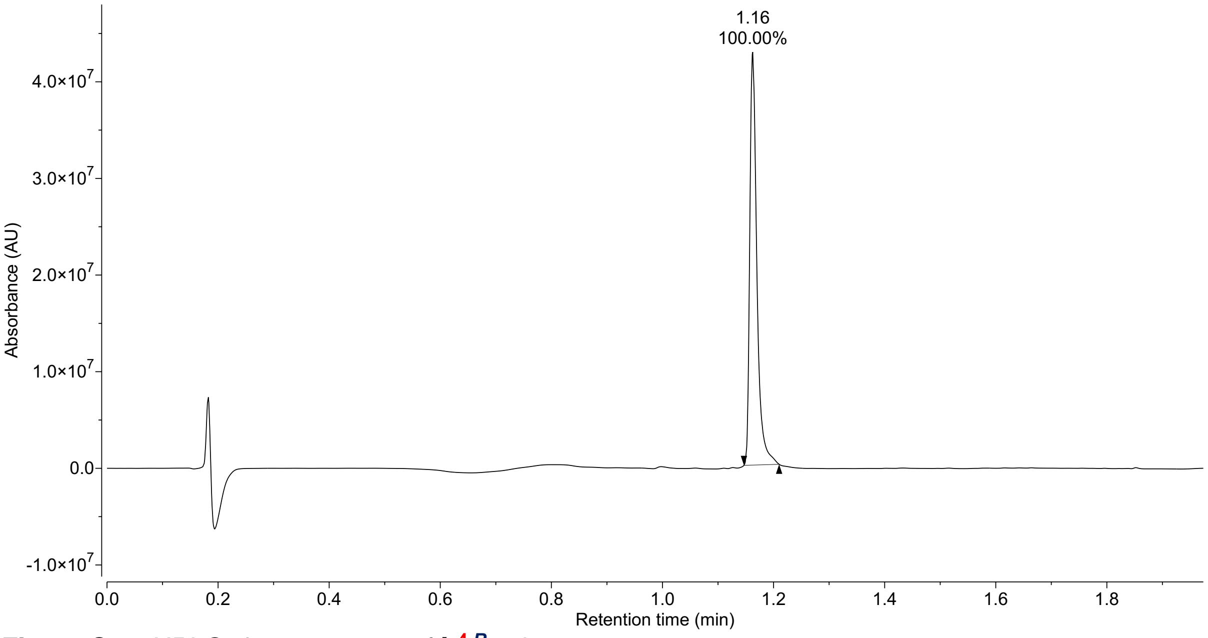

Figure S18. UPLC chromatogram of $\mathbf{L}^{4,R}$ using general solvent system.

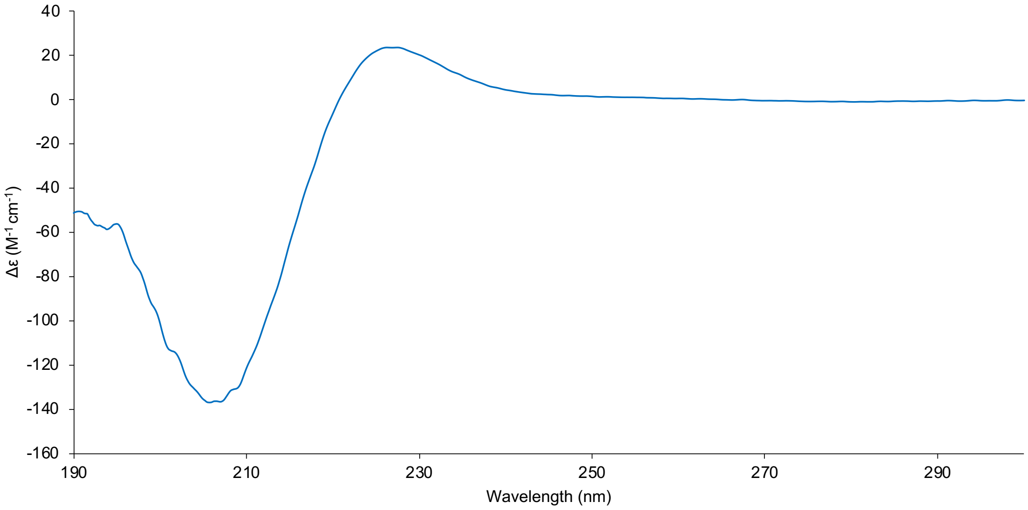

Figure S19. CD spectrum of **L**^{4,R} in H_2O (293 K, 0.15 mM, 1 mm). Minima and maxima observed at 206 nm and 226 nm consistent with folding to the PPII helix.

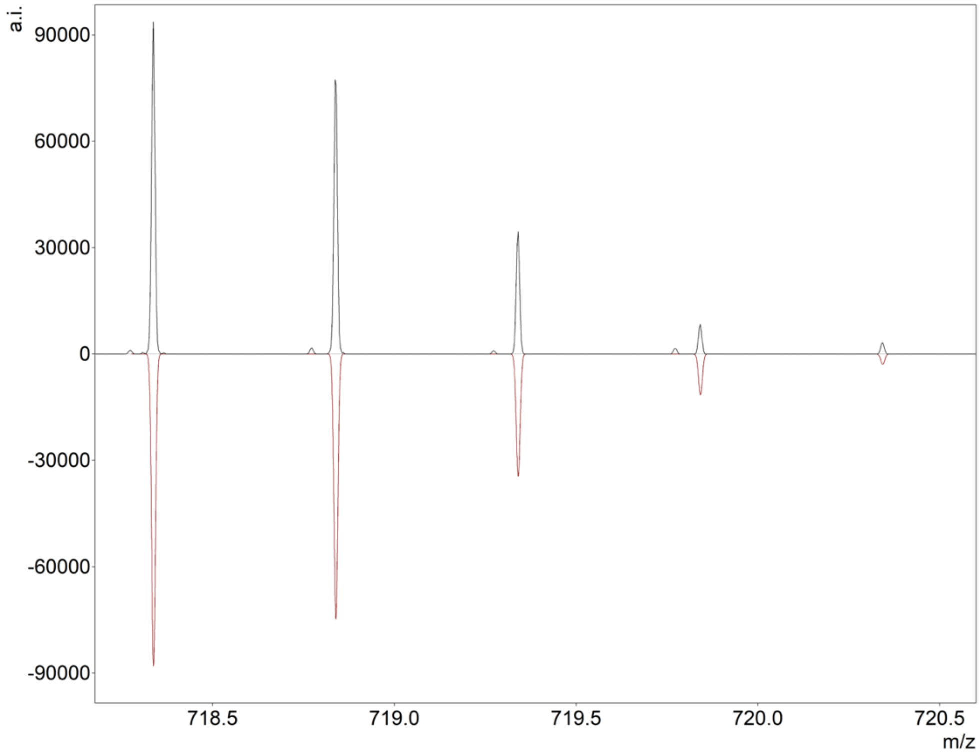

Figure S20. HRMS experimental (top) and calculated (bottom) isotope patterns of [**L**^{4,R} + 2H]$^{2+}$.

Pyr-Peptide Rod **L**^{7,S}

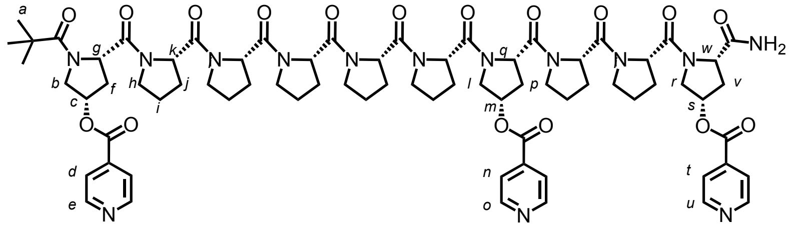

L^{7,S} was prepared by following the **General SPPS Procedure**, then the crude residue was submitted to the **General Coupling Procedure**. Preparative HPLC, using a gradient of 10 – 40% MeCN in H_2O, which was then concentrated by lyophilisation, yielded **L**^{7,S} as a white powder (18.9 mg, 16% overall yield).

^{1}H NMR (600 MHz, D_2O) δ: 8.79 (d, *J* = 6.0 Hz, 2H, H_o), 8.76 (d, *J* = 6.0 Hz, 4H, H_e and H_u), 8.01 – 7.98 (app. t, 4H, H_n and H_d), 7.93 (d, *J* = 6.0 Hz, 2H, H_t), 5.78 (m, 1H, H_m), 5.70 (m, 1H, H_c), 5.63 – 5.61 (br t, 1H, H_s), 4.97 – 4.94 (app t, 2H, H_g and H_m), 4.71 – 4.69 (m, 3H, 3 of H_k), 4.61 – 4.57 (m, 1H, 2 of H_w), 4.27 (app. d, 2H, H_b and H_l), 4.08 (d, 1H, $H_{l'}$), 3.85 – 3.62 (m, 11 H, 10 of H_h, $H_{b'}$), 3.47 (d, *J* = 11.9 Hz, 1H, H_r), 3.41 – 3.37 (m, 1H, 1 of H_h), 3.36 (dd, *J* = 5.5 Hz, 11.9 Hz) 1H, $H_{r'}$), 3.33 – 3.25 (m, 1H, 1 of H_h), 3.07 – 3.03 (app. q, 1H, 1 of H_h), 2.86 – 2.78 (m, 1H, H_p), 2.75 – 2.69 (m, 1H, H_f), 2.65 – 2.60 (m, 1H, H_v), 2.50 (d, *J* = 14.9 Hz, 1H, $H_{p'}$), 2.44 (d, *J* = 14.9 Hz, 1H, $H_{v'}$), 2.35 (d, *J* = 14.9 Hz, 1H, $H_{p'}$), 2.29 – 2.24 (m, 2H, H_j), 2.15 – 1.78 (m, 26H, 12 of H_j, 14 of H_i), 1.30 (s, 9H, H_a). ^{13}C NMR (151 MHz, D_2O) δ 180.3, 179.9, 175.4, 174.5, 173.4, 171.6, 171.2, 169.7, 165.6, 165.4, 149.8, 149.5, 149.4, 149.1, 138.6, 138.5, 138.5, 138.3, 137.5, 124.1, 124.0, 123.9, 123.3, 123.1, 123.0, 76.0, 75.5, 75.4, 75.1, 75.1, 73.7, 72.3, 59.9, 59.8, 59.0, 58.9, 58.9, 58.6, 58.5, 58.4, 58.1, 58.1, 57.8, 57.8, 57.7, 53.5, 52.9, 52.8, 47.6, 47.5, 47.1, 47.0, 36.3, 36.2, 34.5, 34.5, 34.2, 34.2, 32.6, 32.5, 27.8, 27.7, 27.7, 27.7, 27.6, 27.6, 27.5, 27.5, 26.4, 24.5, 24.5, 24.3, 24.2.* ESI-HRMS: m/z observed 718.3342 [**L**^{7,S} + 2H]$^{2+}$, calc. 718.3377 (Figure S30).

*Some peaks have been assigned from 2D NMR spectroscopy.

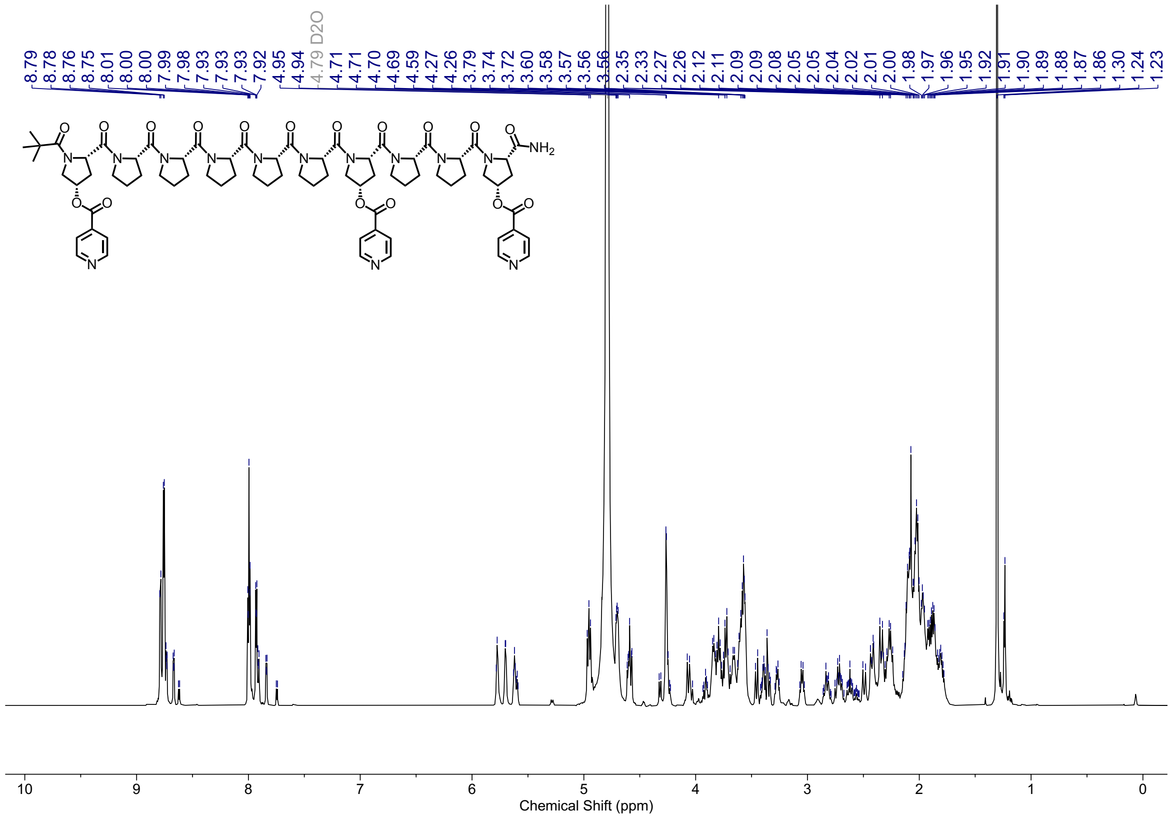

Figure S21. ^{1}H NMR (D_2O, 600 MHz, 298 K) of **L**^{7,S}.

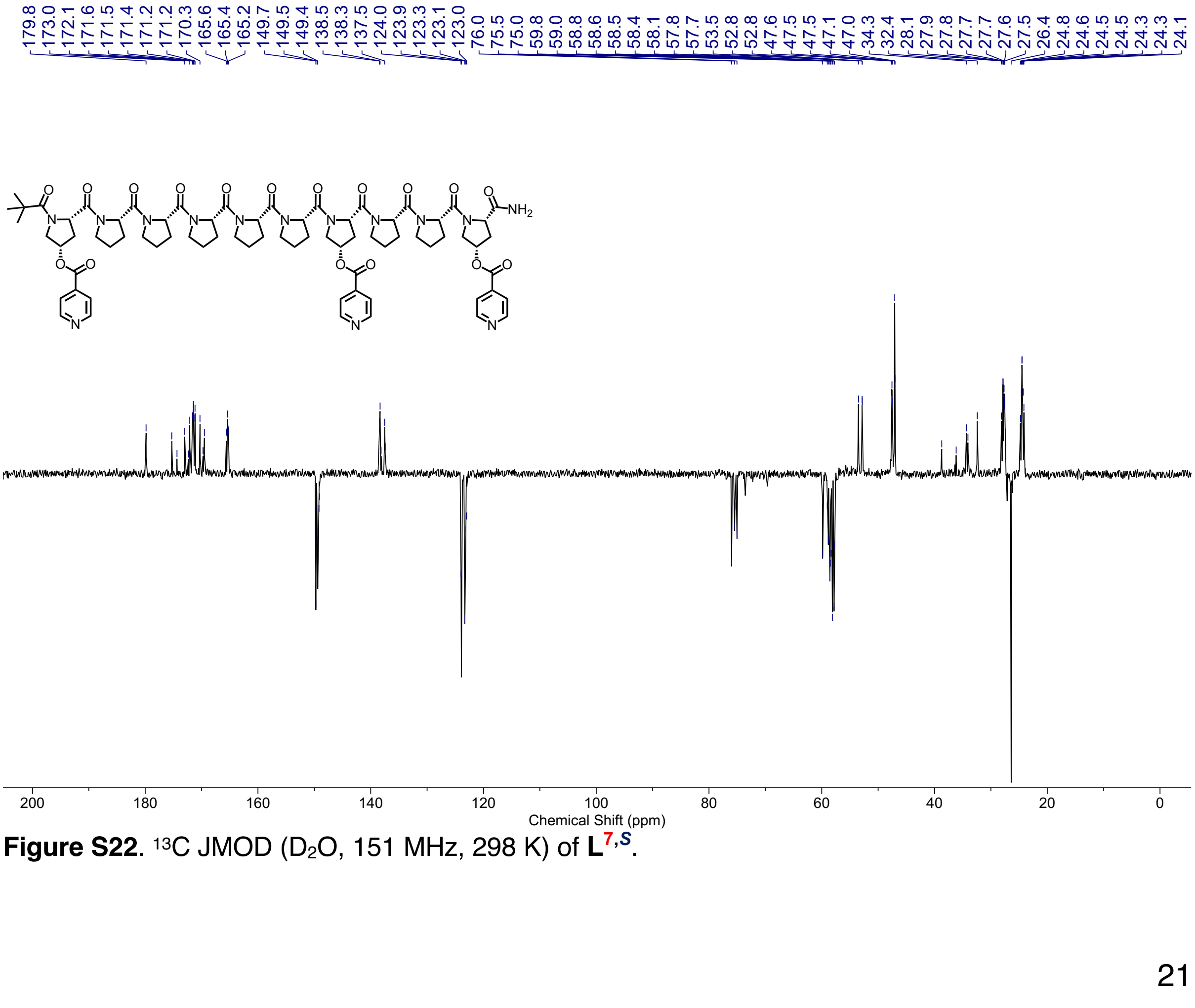

Figure S22. ^{13}C JMOD (D_2O, 151 MHz, 298 K) of **L**^{7,S}.

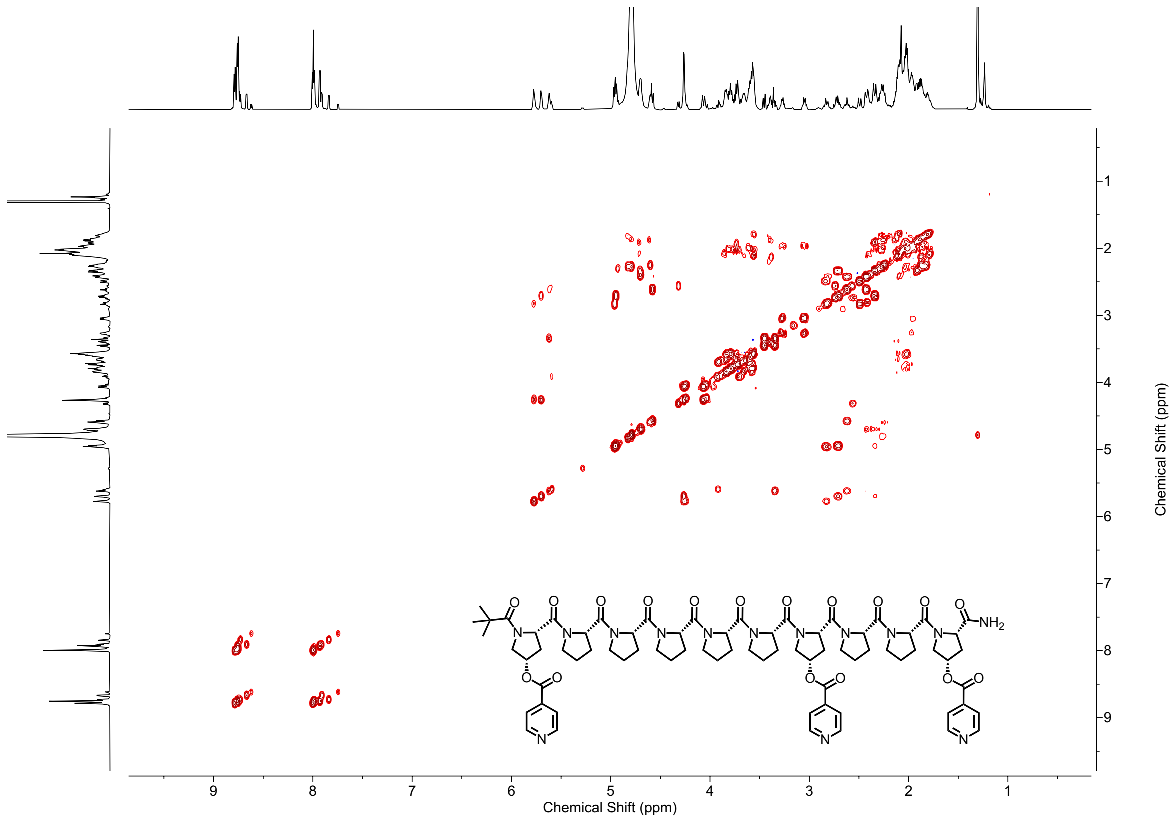

Figure S23. ^1H-^1H COSY (D_2O, 600 MHz, 298 K) of **L**^{7,S}.

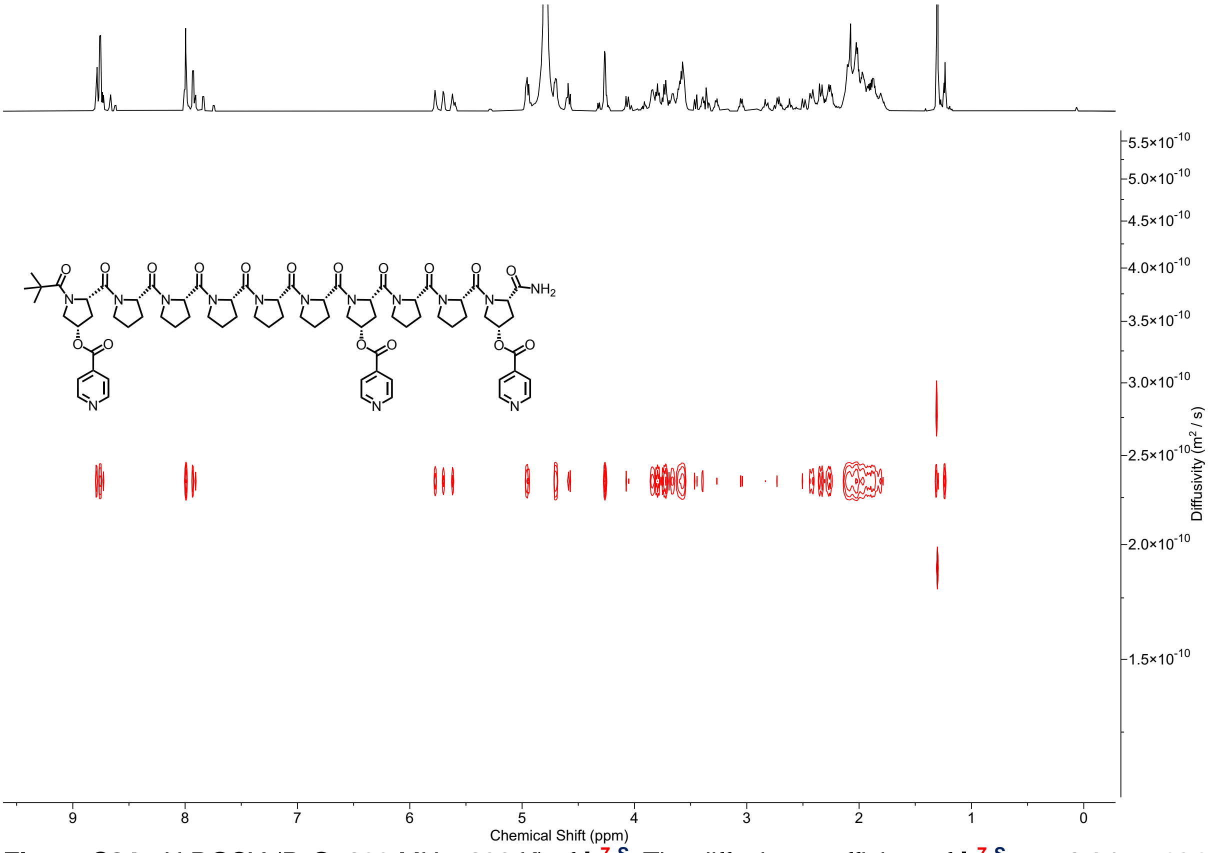

Figure S24. ^1H DOSY (D_2O, 600 MHz, 298 K) of **L**^{7,S}. The diffusion coefficient of **L**^{7,S} was 2.345 x 10^{-9} m^2 s^{-1}, giving a hydrodynamic radius of 13.9 Å.

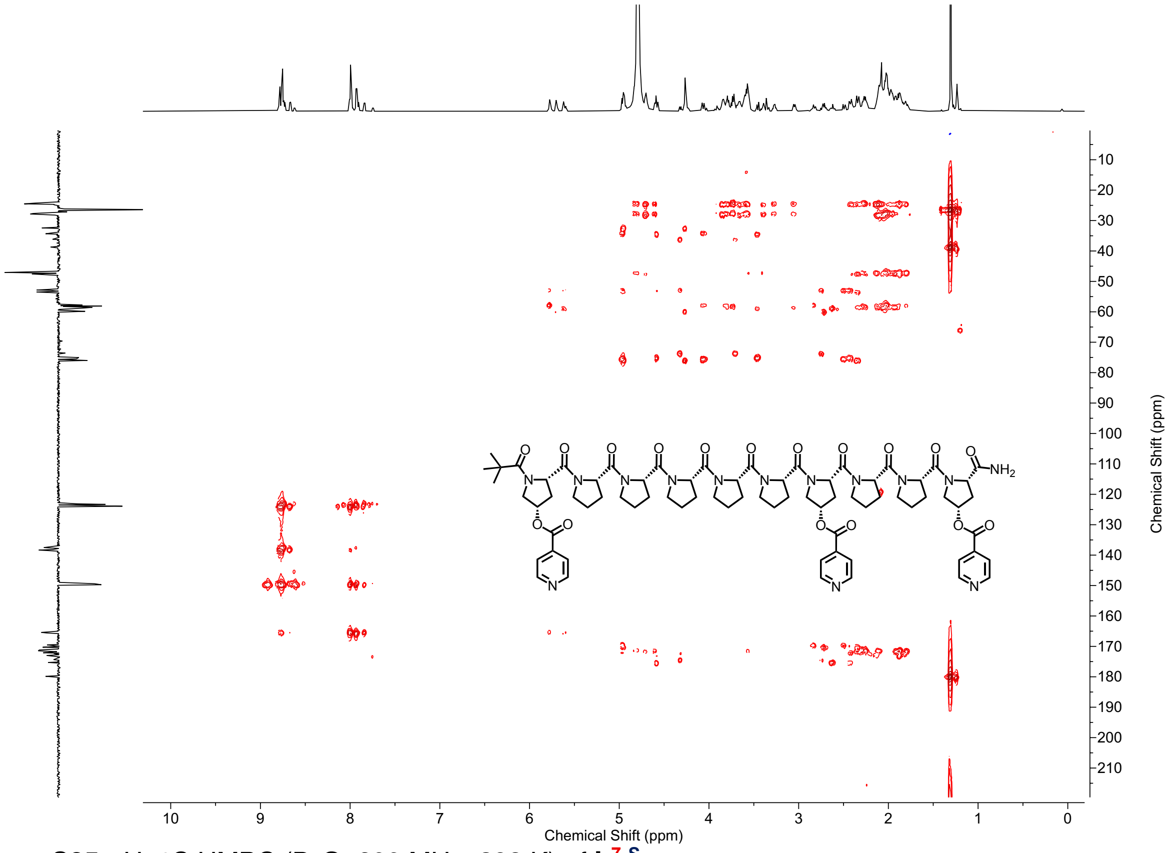

Figure S25. ^{1}H-^{13}C HMBC (D_2O, 600 MHz, 298 K) of **L**^{7,S}.

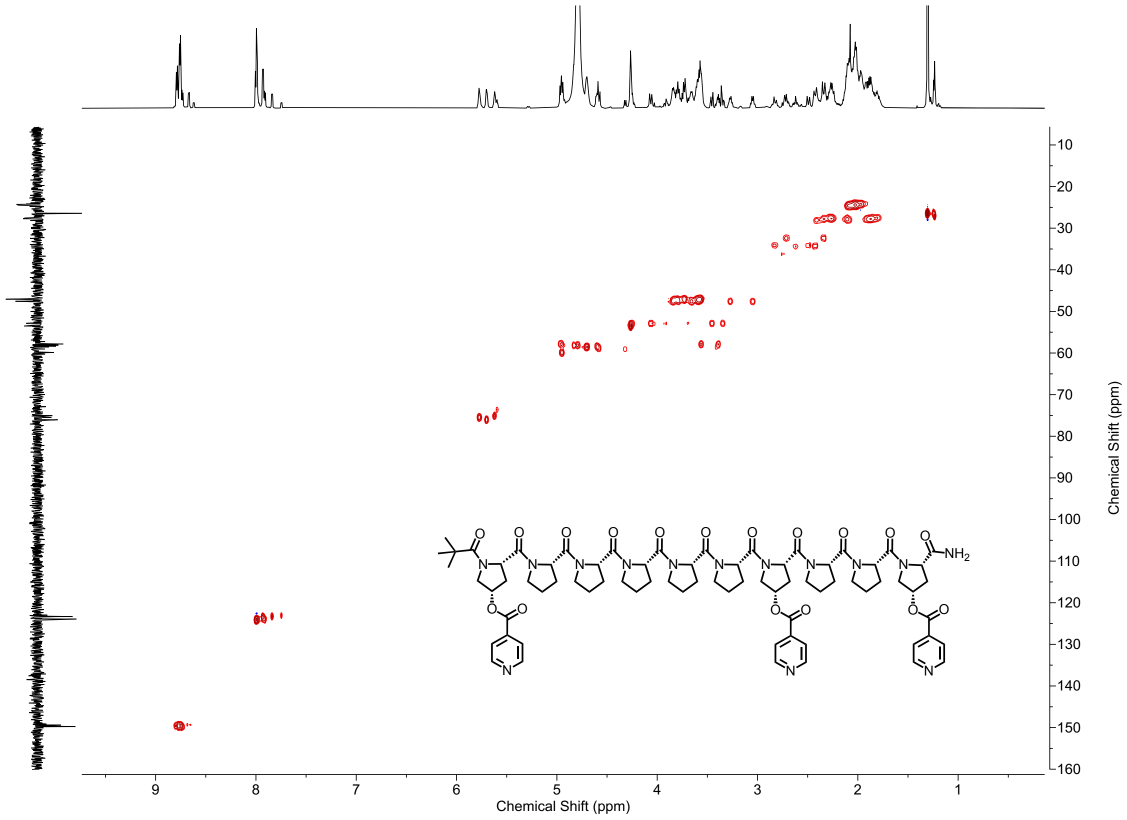

Figure S26. ^{1}H-^{13}C HSQC (D_2O, 600 MHz, 298 K) of **L**^{7,S}.

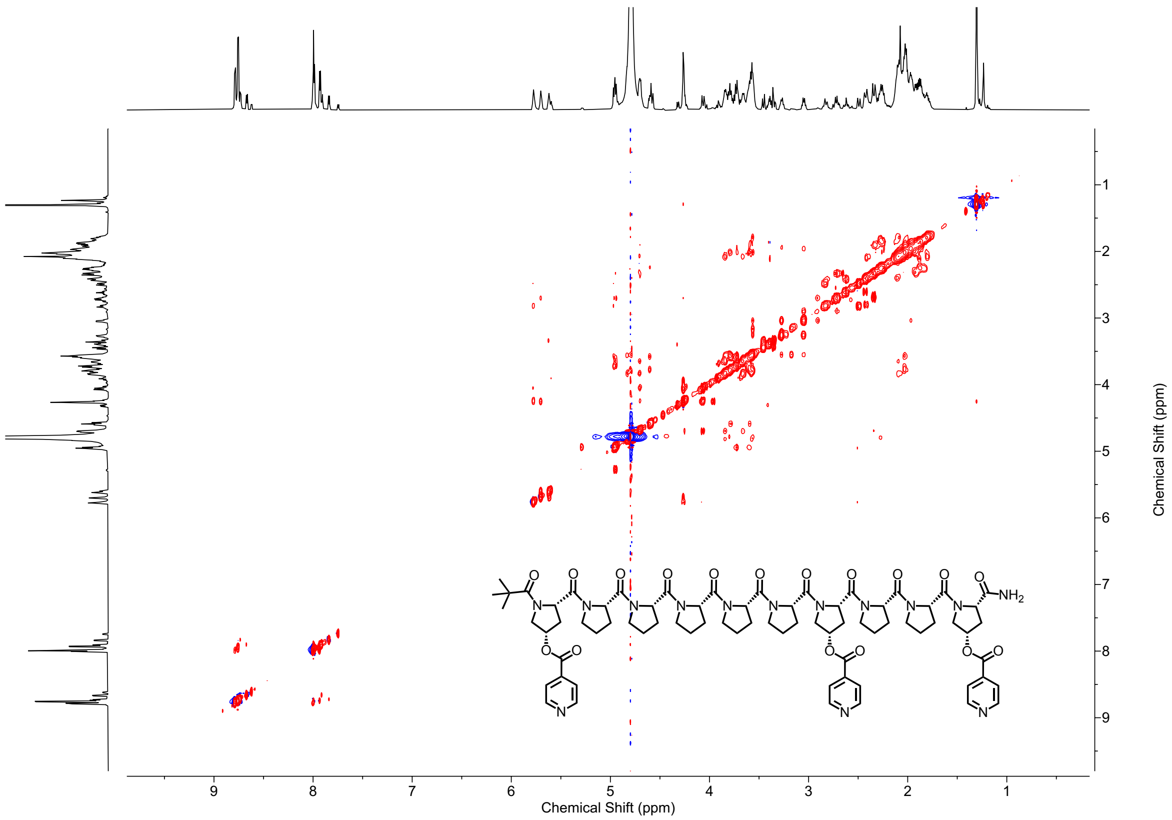

Figure S27. ^{1}H-^{1}H NOESY (D_2O, 600 MHz, 298 K, Mixing time = 0.300 s) of $\mathbf{L}^{7,S}$.

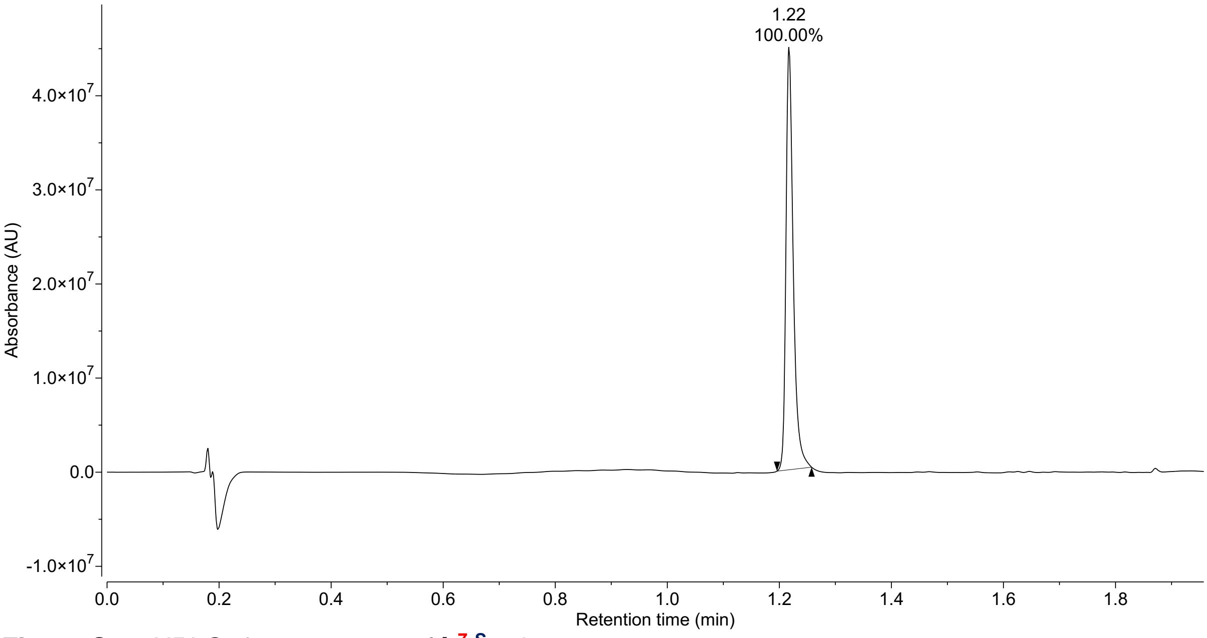

Figure S28. UPLC chromatogram of $\mathbf{L}^{7,S}$ using general solvent system.

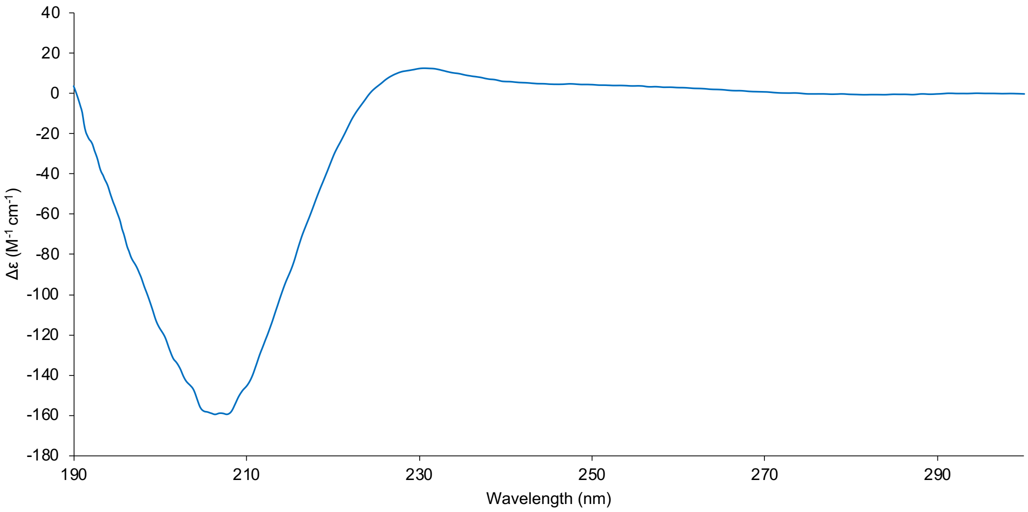

Figure S29. CD spectrum of **L**^{7,S} in H_2O (293 K, 0.15 mM, 1 mm). Minima and maxima observed at 206 nm and 231 nm. Reduction in intensity of maximum consistent with decrease in PPII character.

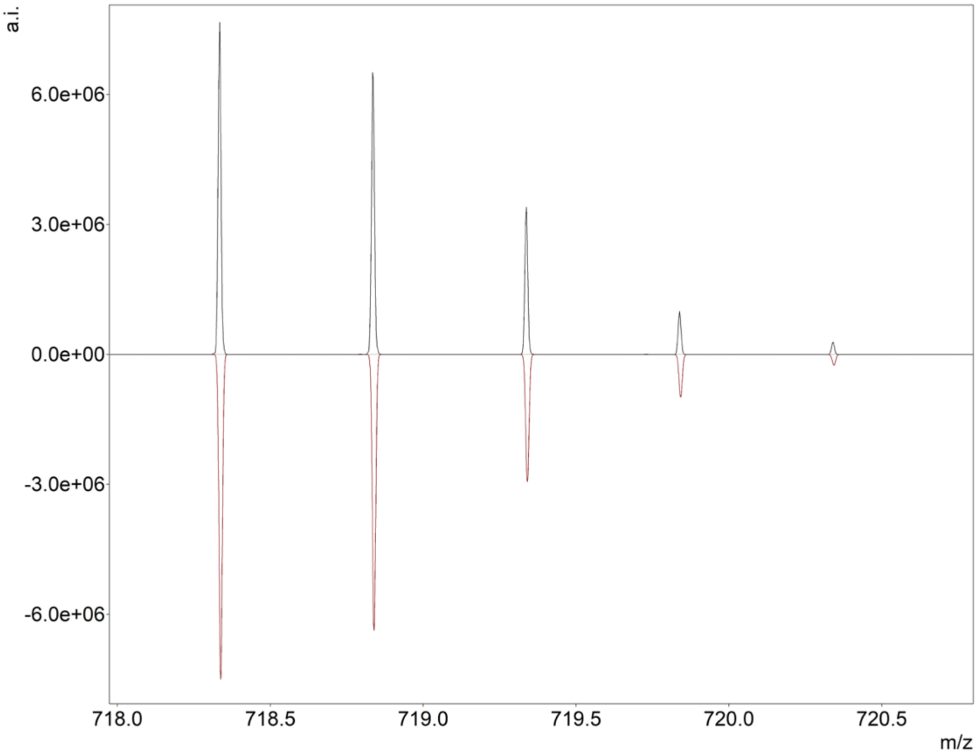

Figure S30. HRMS experimental (top) and calculated (bottom) isotope patterns of [**L**^{7,S} + 2H]$^{2+}$.

Pyr-Peptide Rod **L**^{4,S}

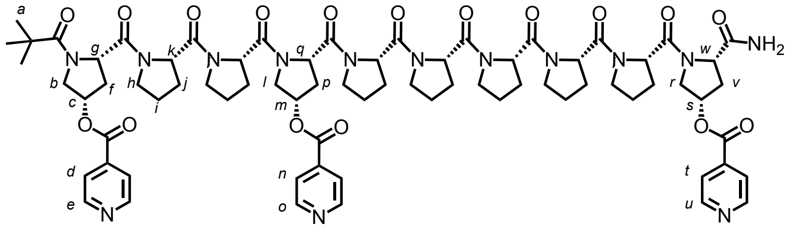

L^{4,S} was prepared by following the **General SPPS Procedure**, then the crude residue was submitted to the **General Coupling Procedure**. Preparative HPLC, using a gradient of 10 – 40% MeCN in H_2O, which was then concentrated by lyophilisation, yielded **L**^{4,S} as a white powder (47.3 mg, 40% overall yield).

^{1}H NMR (600 MHz, D_2O) δ: 8.77 (d, J = 6.0 Hz, 2H, H_o), 8.76 – 8.73 (m, 4H, H_e and H_u), 8.00 (d, J = 6.1 Hz, 2H, H_d), 7.97 (d, J = 6.0 Hz, 2H, H_n), 7.92 (d, J = 6.2, 2H, H_t), 5.78 – 5.74 (app t, 1H, H_s) 5.74 – 5.68 (app q, 1H, H_c), 5.69 – 5.65 (app t, 1H, H_m), 4.96 (d, J = 10.1 Hz, 1H, H_g), 4.85 – 4.73 (m, 3H, 1 of H_k, H_q and H_w), 4.72 – 4.61 (m, 2H, 2 of H_k), 4.61 – 4.54 (m, 1H, 1 of H_k), 4.30 (dd, J = 12.6, 5.0 Hz, 1H, H_r), 4.27 (d, J = 2.8 Hz, 2H, H_b), 3.99 (d, J = 13.0 Hz, 2H, H_l and $H_{r'}$), 3.90 – 3.47 (m, 16H, 12 of H_h, 3 of H_k, $H_{l'}$), 3.29 – 3.21 (m, 1H, 1 of H_h), 3.01 – 2.93 (m, 1H, 1 of H_h), 2.78 – 2.62 (m, 3H, H_f, H_p and H_v), 2.51 (d, J = 14.5 Hz, 1H, $H_{v'}$), 2.45 – 1.75 (m, 30H, $H_{f'}$, 14 of H_i, 14 of H_j, $H_{p'}$), 1.31 (s, 9H, H_a). ^{13}C NMR (151 MHz, D_2O) δ: 179.9, 175.4, 173.1, 172.0, 171.7, 171.5, 171.4, 171.4, 170.9, 170.4, 169.4, 165.7, 165.5, 165.2, 149.7, 149.5, 149.4, 138.4, 138.3, 137.6, 124.0, 123.3, 123.2, 76.0, 75.5, 75.1, 59.9, 59.5, 59.3, 59.0, 58.5, 58.5, 58.3, 58.2, 58.2, 57.9, 57.8, 57.7, 53.5, 52.9, 52.8, 52.7, 47.6, 47.5, 47.2, 47.1, 47.0, 38.7, 34.6, 33.8, 32.4, 28.2, 27.8, 27.8, 27.7, 27.7, 27.6, 27.5, 26.4, 24.7, 24.7, 24.5, 24.5, 24.3, 24.3, 24.1. ESI-HRMS: m/z observed 718.3340 [**L**^{4,S} + 2H]$^{2+}$, calc. 718.3377 (Figure S40).

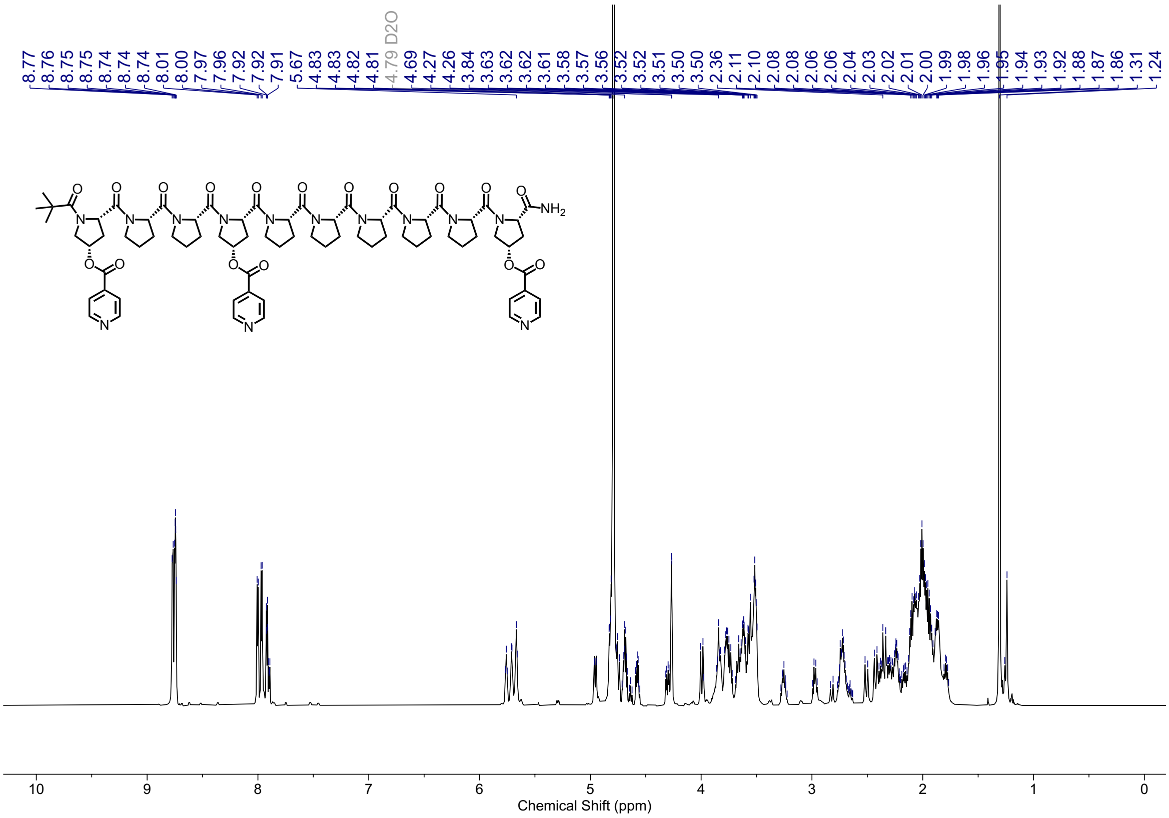

Figure S31. ^{1}H NMR (D_2O, 600 MHz, 298 K) of **L**^{4,S}.

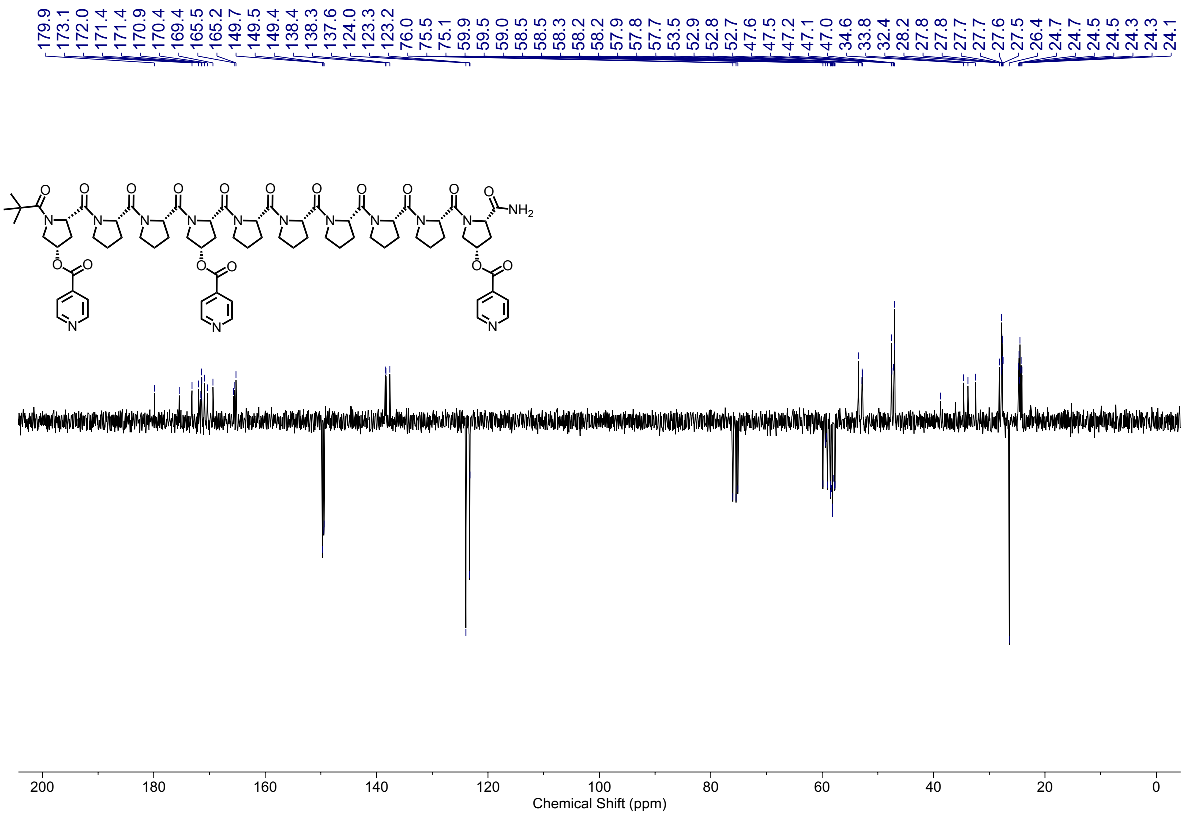

Figure S32. ^{13}C JMOD (D_2O, 151 MHz, 298 K) of **L**^{4,S}.

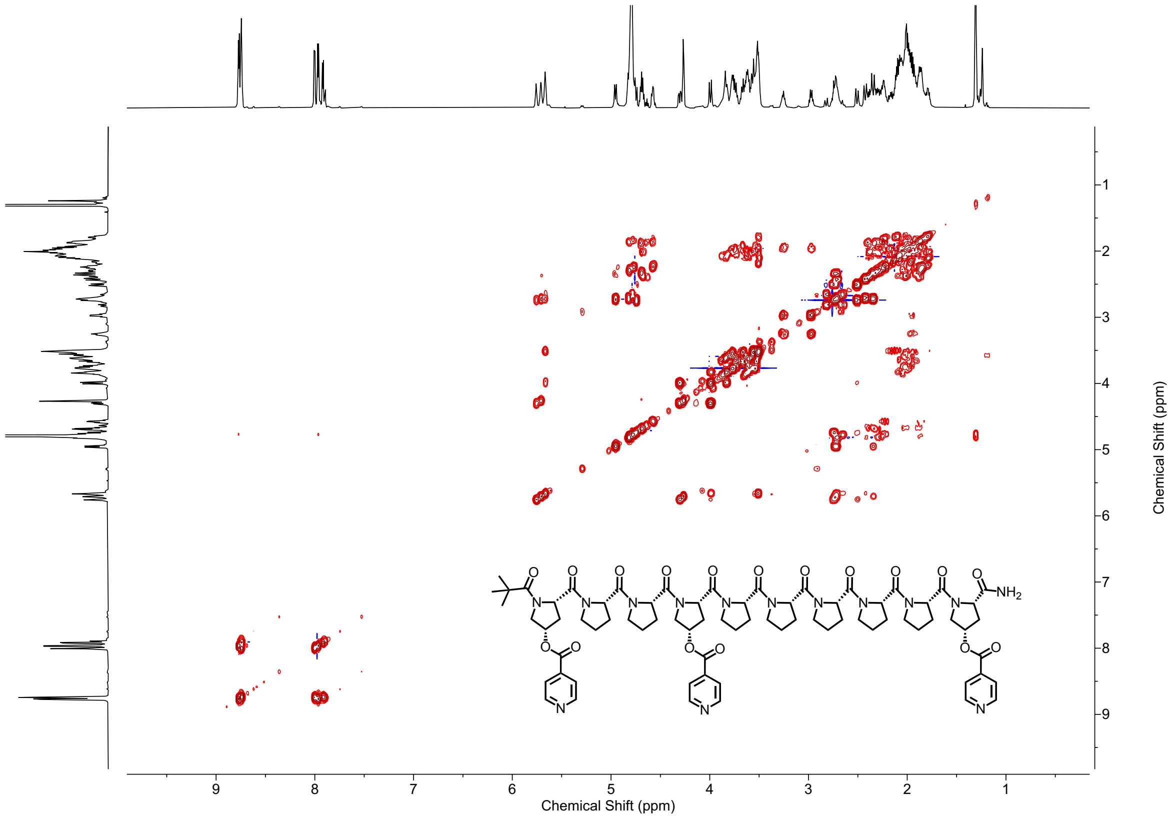

Figure S33. ^{1}H-^{1}H COSY (D_2O, 600 MHz, 298 K) of $\mathbf{L}^{4,S}$.

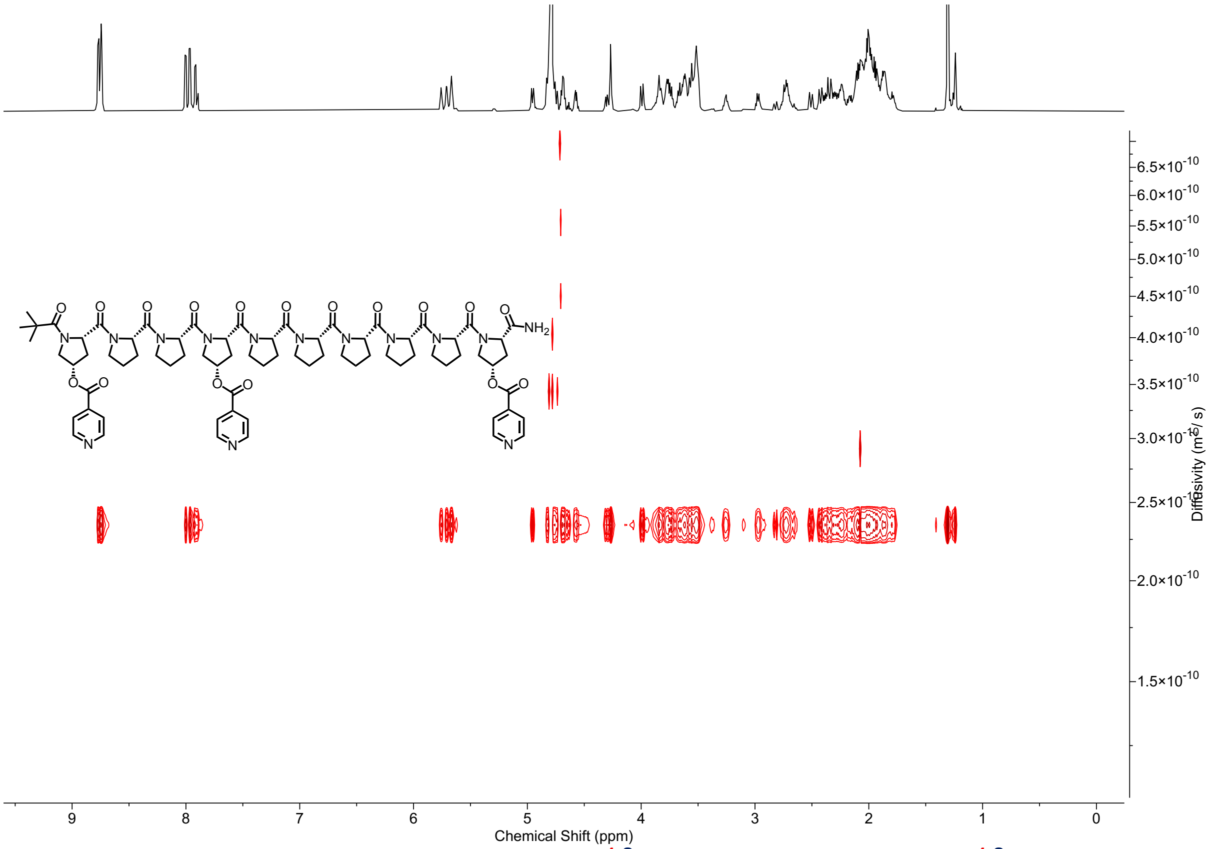

Figure S34. ^{1}H DOSY (D_2O, 600 MHz, 298 K) of $\mathbf{L}^{4,S}$.The diffusion coefficient of $\mathbf{L}^{4,S}$ was 2.345 x 10^{-9} m^2 s^{-1}, giving a hydrodynamic radius of 13.9 Å.

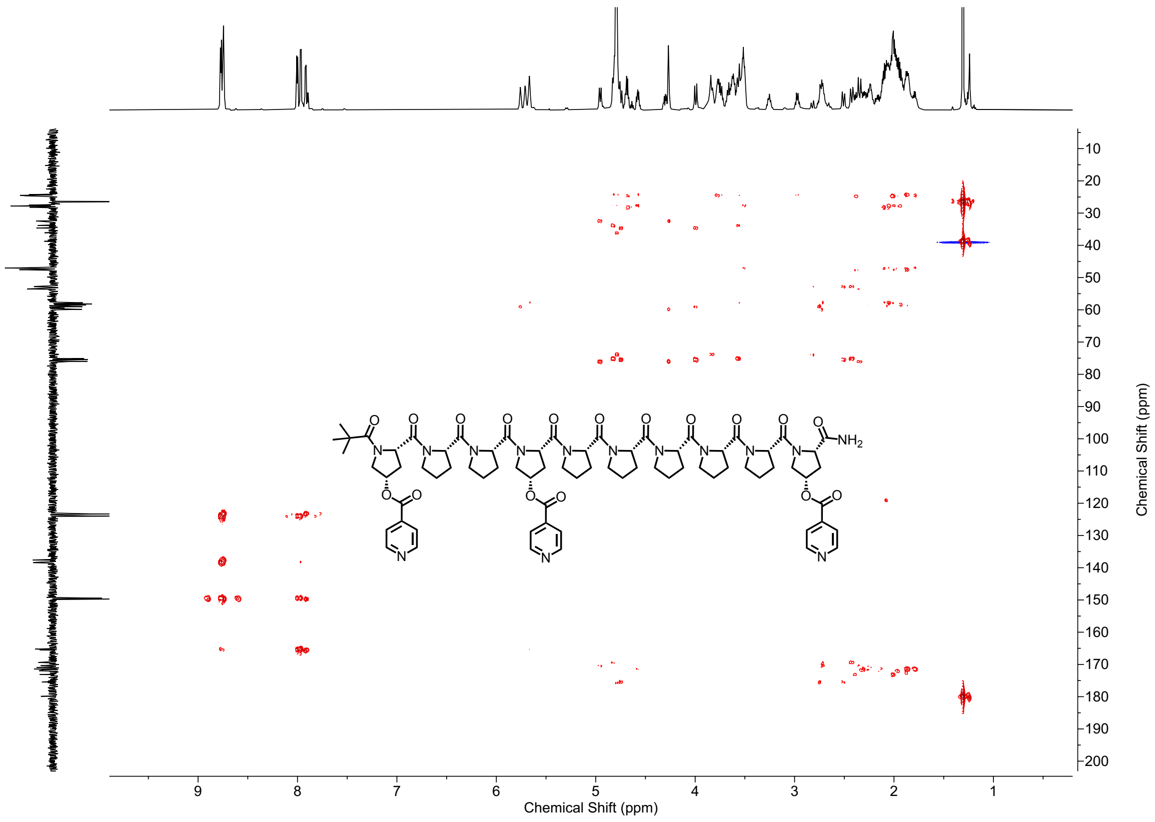

Figure S35. ¹H-¹³C HMBC (D_2O, 600 MHz, 298 K) of **L**[4,*S*].

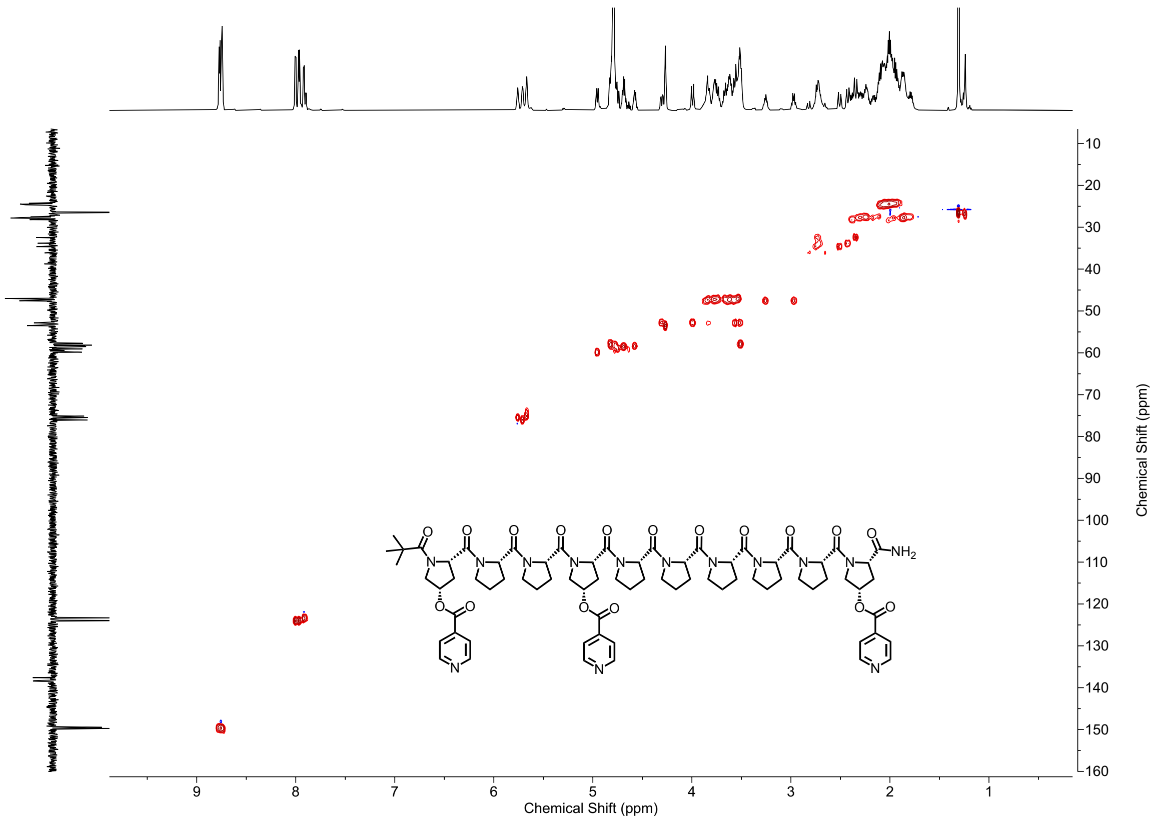

Figure S36. ¹H-¹³C HSQC (D_2O, 600 MHz, 298 K) of **L**[4,*S*].

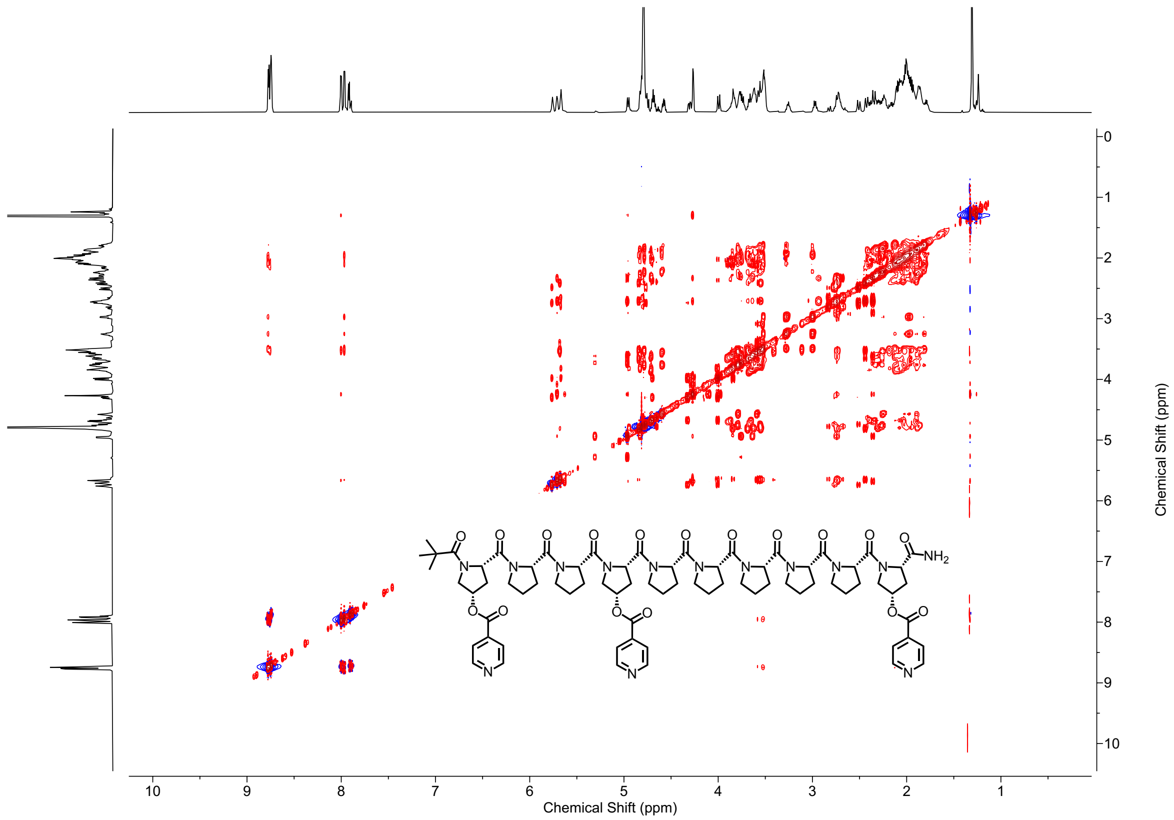

Figure S37. ^1H-^1H NOESY (D_2O, 600 MHz, 298 K, Mixing time = 0.300 s) of $\mathbf{L}^{4,S}$.

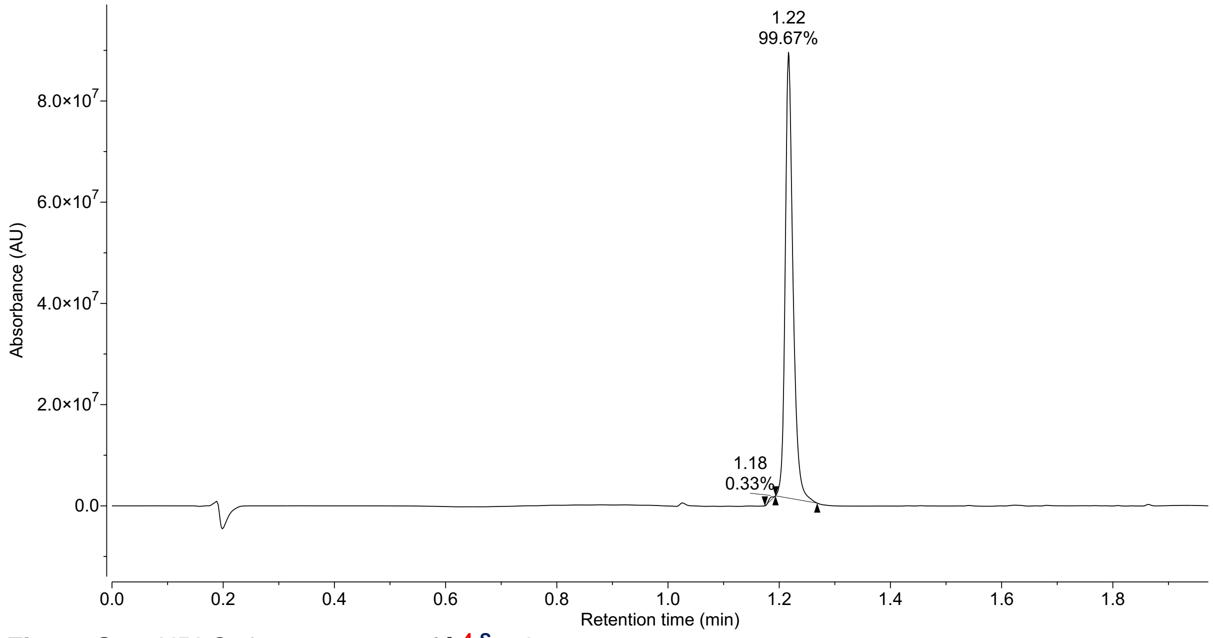

Figure S38. UPLC chromatogram of $\mathbf{L}^{4,S}$ using general solvent system.

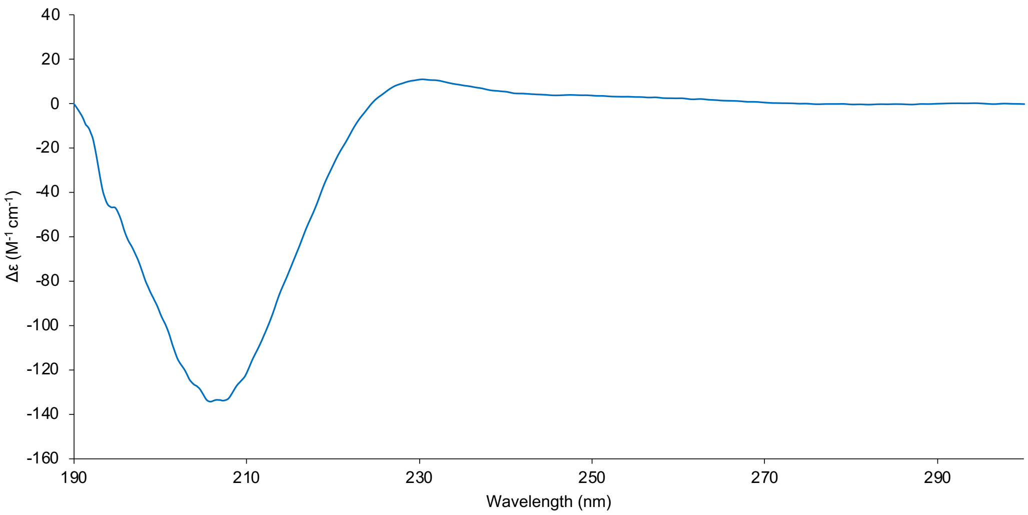

Figure S39. CD spectrum of **L**^{4,S} in H_2O (293 K, 0.15 mM, 1 mm). Minima and maxima observed at 206 nm and 230 nm. Reduction in intensity of maximum consistent with decrease in PPII character.

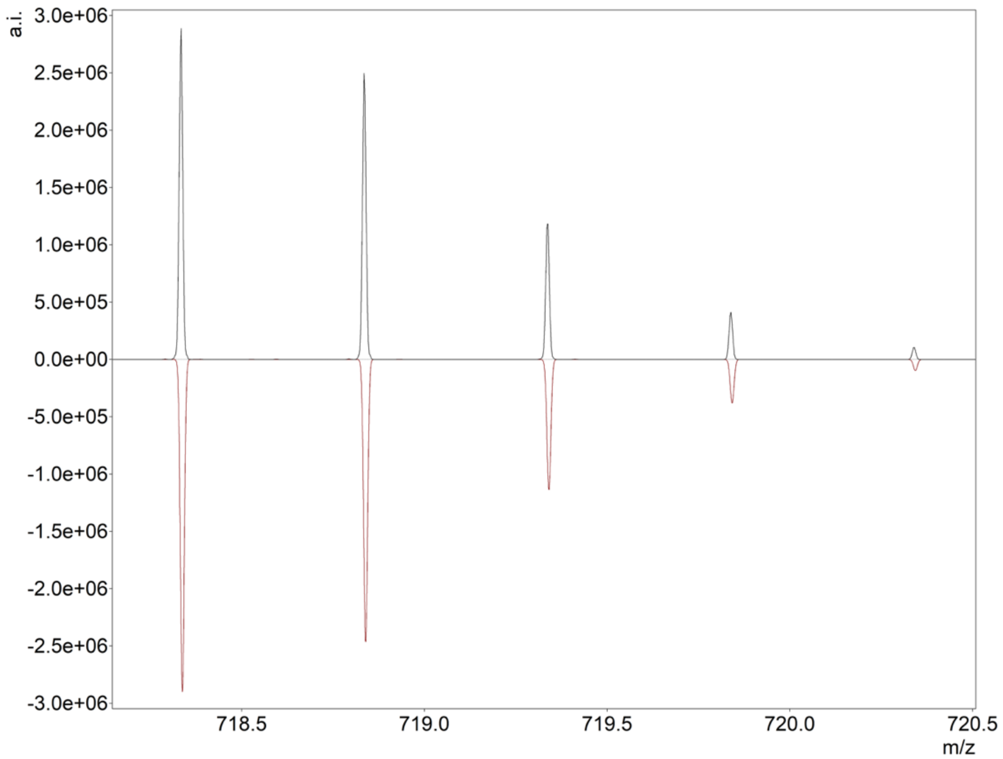

Figure S40. HRMS experimental (top) and calculated (bottom) isotope patterns of [**L**^{4,S} + 2H]$^{2+}$.

4. Synthesis of Metal Peptidic Cages

The diastereoisomeric splitting that often occurs in cages results in a significant increase in complexity from the starting ligand.

For **$Pd_3L^{7,R}_4$**, in adopting the *cis CCNN* isomer, the ligand environment splits into two distinct sets of peaks, the 'left' and the 'right' side from the cage.[S3]

Where it is possible to discern signals that belong to a particular 'side', the peaks will be noted in red or blue respectively, the colour being chosen arbitrarily as we are unable to unambiguously assign a 'left' or 'right', but the colour being consistent, by following correlations through spin systems. Signal overlap and weak HMBC signal prevented full assignments being made, but where possible specific peaks have been assigned, and will be reported below.

As previously reported,[S3] NMR relaxation times complicated assignments. The increased T2 relaxation rate, particularly on the atoms closest to the palladium centre, caused loss of signals in HMBC spectra, where a long evolution delay is required (60 ms) to transfer polarisation *via* long range couplings. HSQC provided stronger signals, where the delay is only of the order of 12 ms. Likewise, ^{13}C direct-detected experiments suffered low signal-to-noise, and the weaker signals from quaternary carbons were often absent.

As the cages are prepared in D_2O, there is a level of deuterium exchange on the terminal free amide, which has been accounted for in the MS simulations.

Metal-Peptidic Cage $\mathbf{Pd_3L^{7,R}}_4(BF_4)_6$

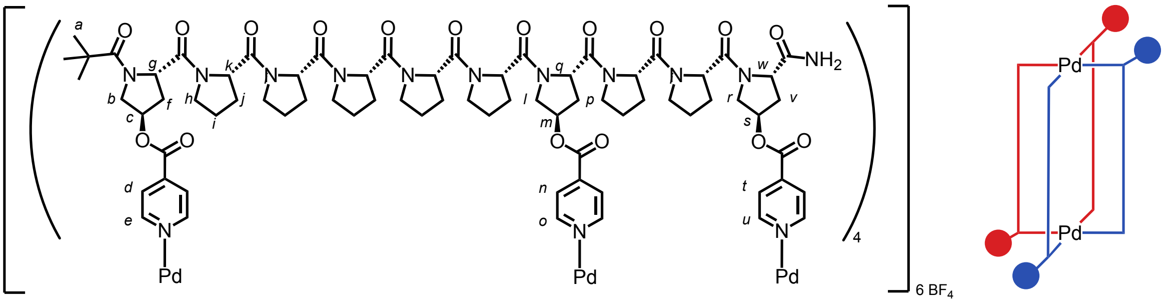

$\mathbf{Pd_3L^{7,\mathit{R}}_4}(BF_4)_6$ was prepared by submitting $\mathbf{L^{7,\mathit{R}}}$ to the **General Cage Procedure**. This yielded 500 µL of $\mathbf{Pd_3L^{7,\mathit{R}}_4}(BF_4)_6$ as a pale-yellow solution, at 0.535 mM, which was used without further purification.

^{1}H NMR (950 MHz, D_2O) δ: 9.31 (d, J = 6.8 Hz, 4H, H_o), 9.19 (d, J = 6.9 Hz, 4H, H_o), 9.04 – 8.94 (m, 16H, H_e, and H_u) 8.21 (d, J = 6.9 Hz, 2H, H_n), 8.11 – 8.02 (m, 20H, H_d, H_n, and H_t), 5.95 (br s, 2H, and H_m), 5.79 (s, 2H, $H_{s\text{ or }s}$)*, 5.73 – 5.69 (m, 2H, H_c), 5.67 – 5.63 (m, 6H, H_c, H_m, and $H_{s\text{ or }s}$), 4.95 – 4.55 (m, 36H, H_g, H_k, and H_q), 4.57 – 4.54 (app t., 2H, $H_{w\text{ or }w}$), 4.47 – 4.43 (m, 4H, H_l, $H_{w\text{ or }w}$), 4.35 – 4.30 (m, 4H, H_b), 4.24 – 4.18 (m, 6H, $H_{b'}$, H_l, and $H_{r\text{ or }r}$) 4.12 (d, J = 12.2 Hz, 2H, $H_{r\text{ or }r}$), 4.09 – 4.07 (m, 2H, $H_{g'}$) 4.03 – 4.01 (m, 2H, H $H_{r'\text{ or }r'}$), 3.98 – 3.92 (m, 6H, Hl’, and $H_{r'\text{ or }r'}$), 3.92 – 3.51 (m, 26H, H_h), 3.39 – 3.36 (m, 2H, 2 of H_h), 2.98 – 2.92 (m, 2H, H_p), 2.71 – 2.65 (m, 6H, H_p, and H_v), 2.46 – 1.81 (m, 128H, H_f, H_j, H_i, $H_{p'}$, and $H_{v'}$), 1.27 (s, 18H, H_a), 1.26, (s, 18H, H_a). ^{13}C NMR (151 MHz, D_2O) δ: 180.9, 176.2, 172.2, 164.2, 163.9, 152.7, 141.9, 127.3, 127.1, 126.8, 126.6, 76.7, 59.5, 59.2, 48.2, 39.0, 35.3, 35.0, 32.8, 28.5, 26.8, 25.2. ESI-MS: m/z $[Pd_3\mathbf{L}^{7,\mathit{R}}{}_4]^{6+}$,1010.7286, $[Pd_3\mathbf{L}^{7,\mathit{R}}{}_4 + BF_4]^{5+}$, 1230.6733. HRMS: m/z observed 1010.7286, calc. 1010.7213 (Figure S49).

*The *C*-terminus hydroxyproline cannot be connected to either blue and red spin system for the rest of the ligand, so one spin system for the *C*-terminus Hyp has been arbitrarily labelled as blue-or-red, i.e. $H_{s\text{ or }s}$, and the other as red-or-blue, i.e. $H_{s\text{ or }s}$, and are internally self-consistent with the rest of the isolated *C*-terminus hydroxyproline signals.

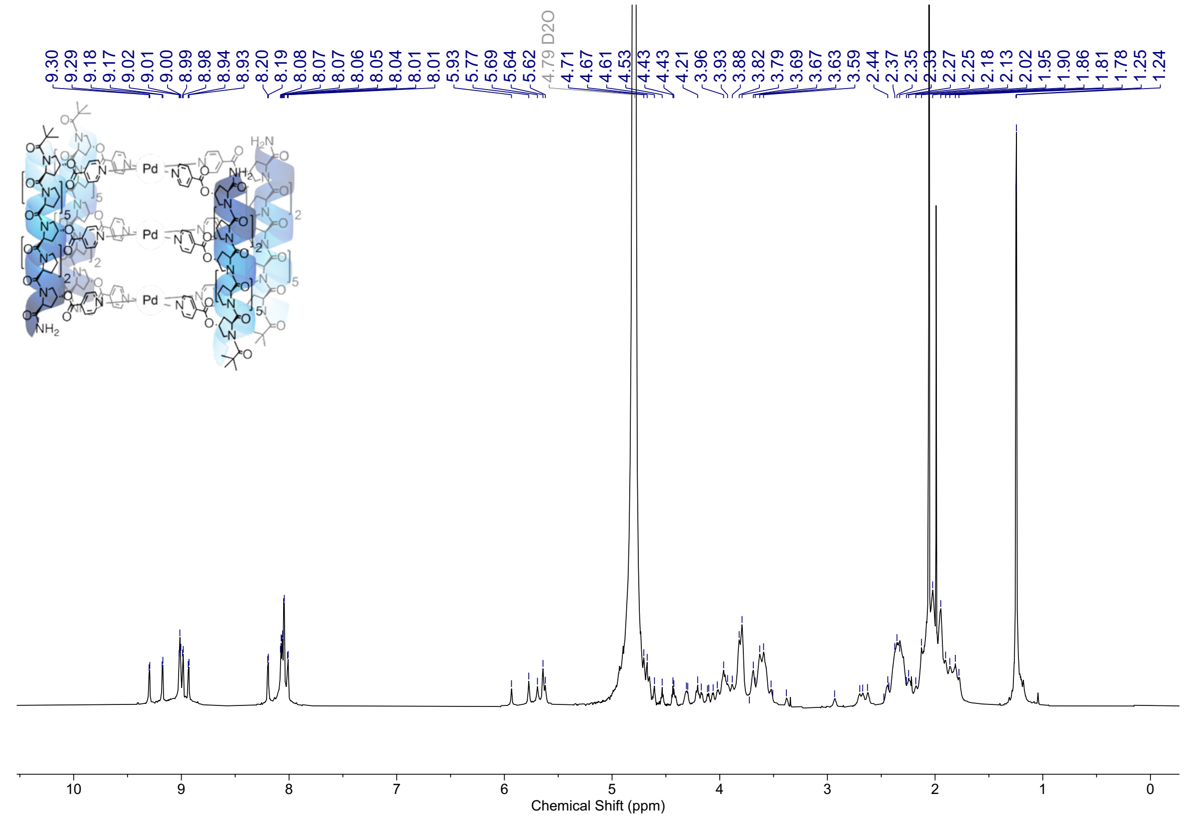

Figure S41. ¹H NMR (D_2O, 950 MHz, 298 K) of $\mathbf{Pd_3L^{7,R}_4}(BF_4)_6$.

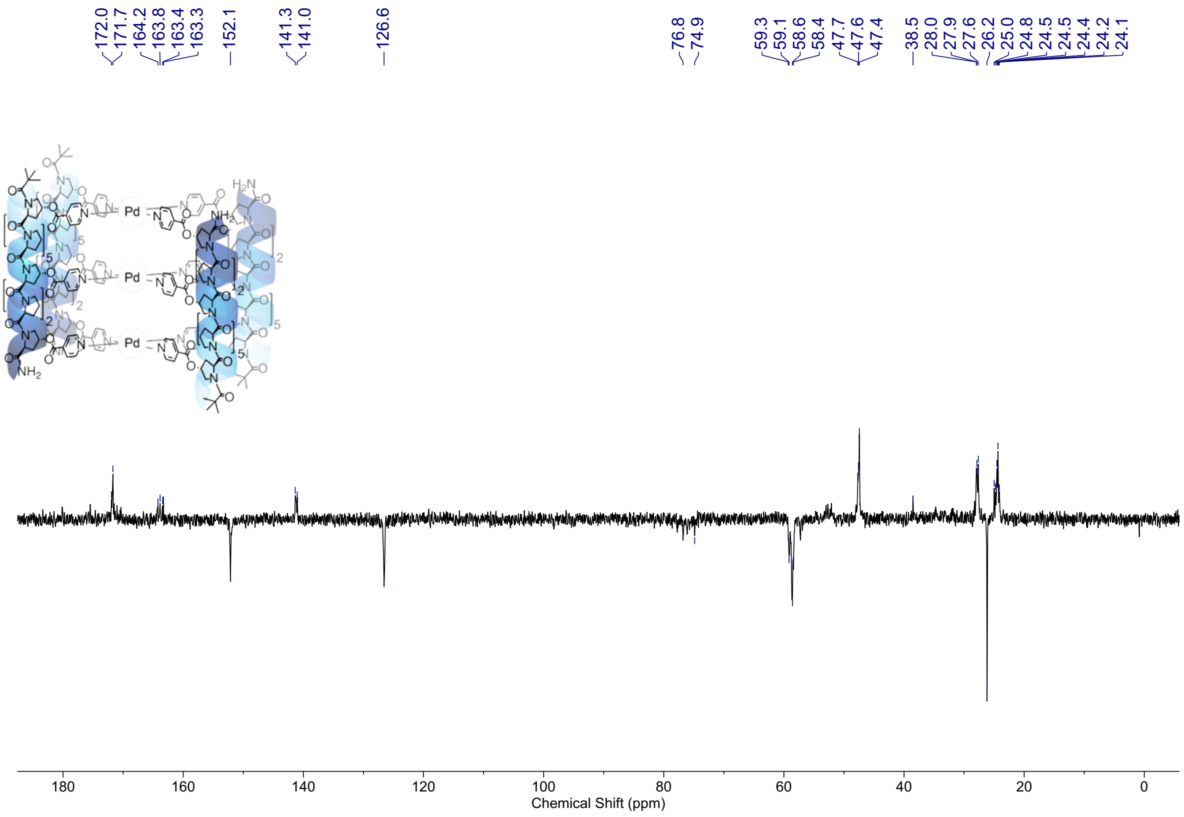

Figure S42. ¹³C JMOD (D_2O, 151 MHz, 298 K) of $\mathbf{Pd_3L^{7,R}_4}(BF_4)_6$.

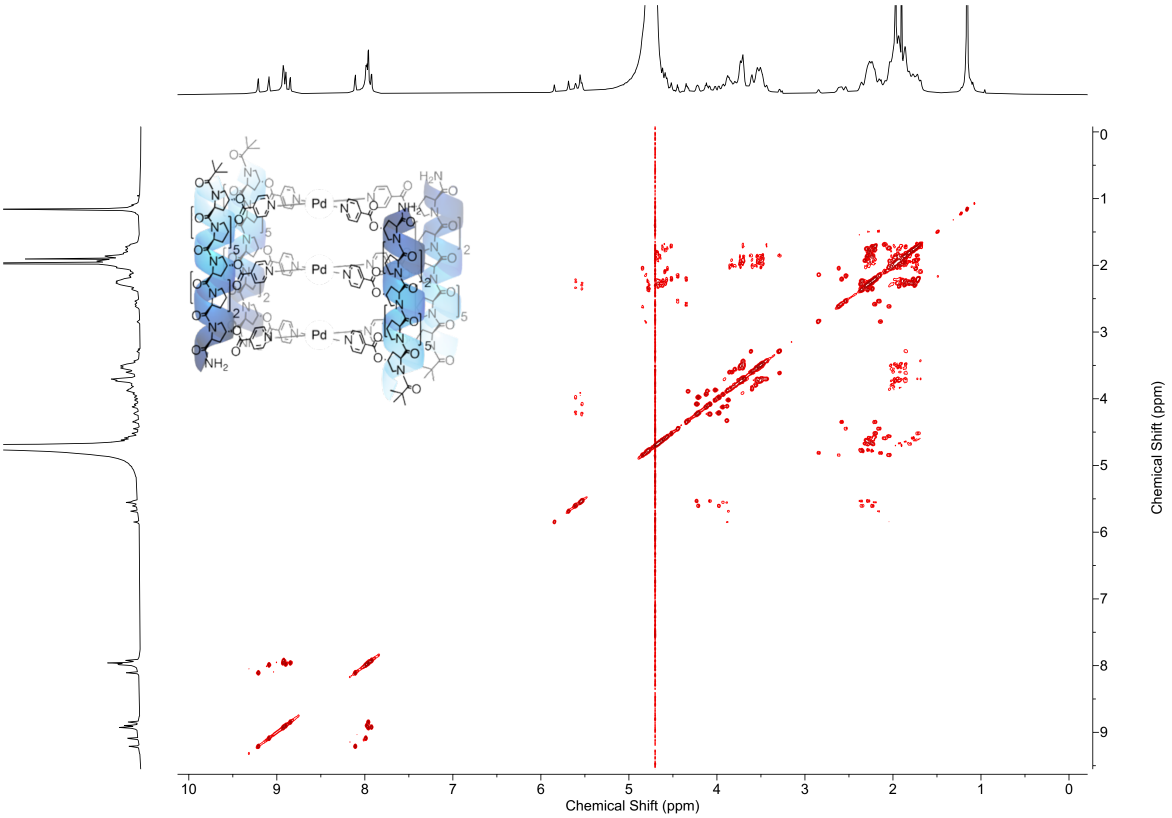

Figure S43. ^{1}H-^{1}H COSY (D_2O, 600 MHz, 298 K) of $\mathbf{Pd_3L^{7,R}_4}(BF_4)_6$.

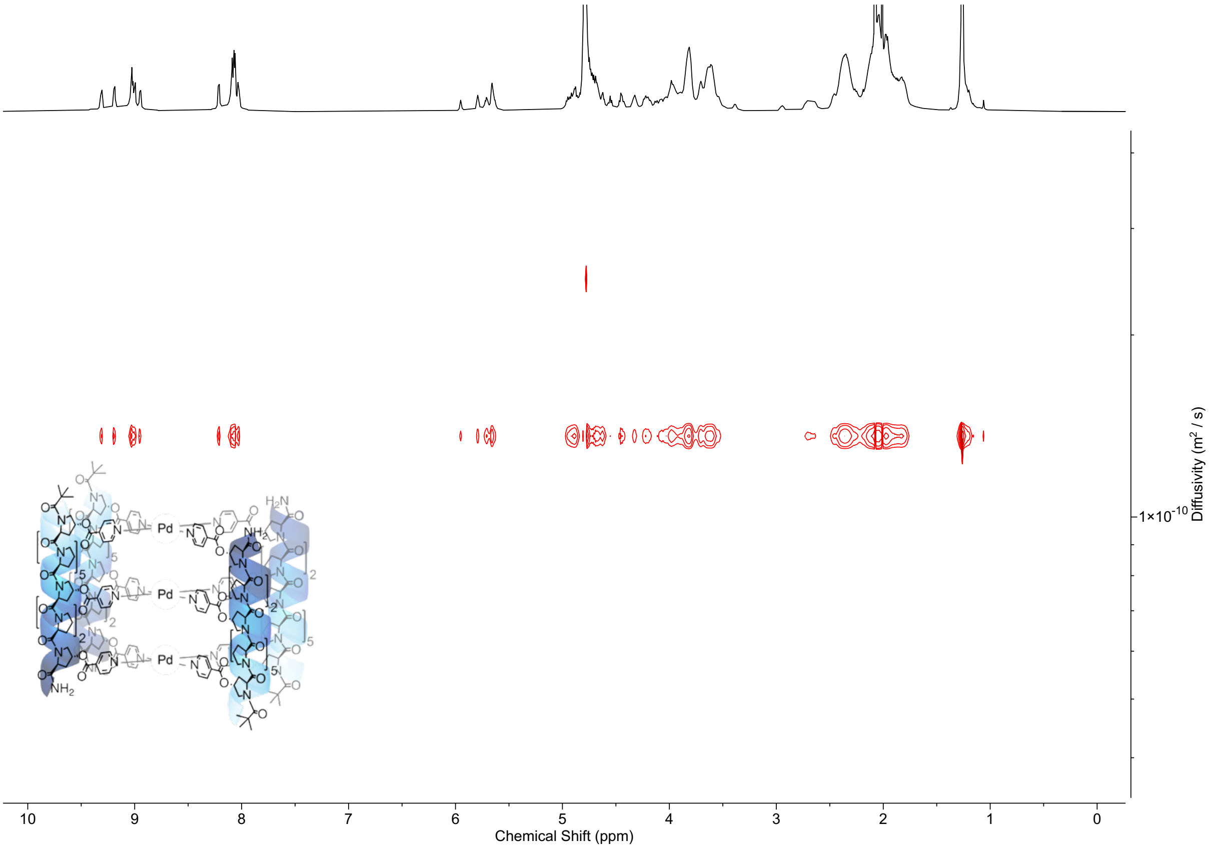

Figure S44. ^{1}H DOSY (D_2O, 600 MHz, 298 K) of $\mathbf{Pd_3L^{7,R}_4}(BF_4)_6$. The diffusion coefficient of $\mathbf{Pd_3L^{7,R}_4}(BF_4)_6$ was 1.360 x 10^{-9} m^2 s^{-1}, giving a hydrodynamic radius of 24.0 Å.

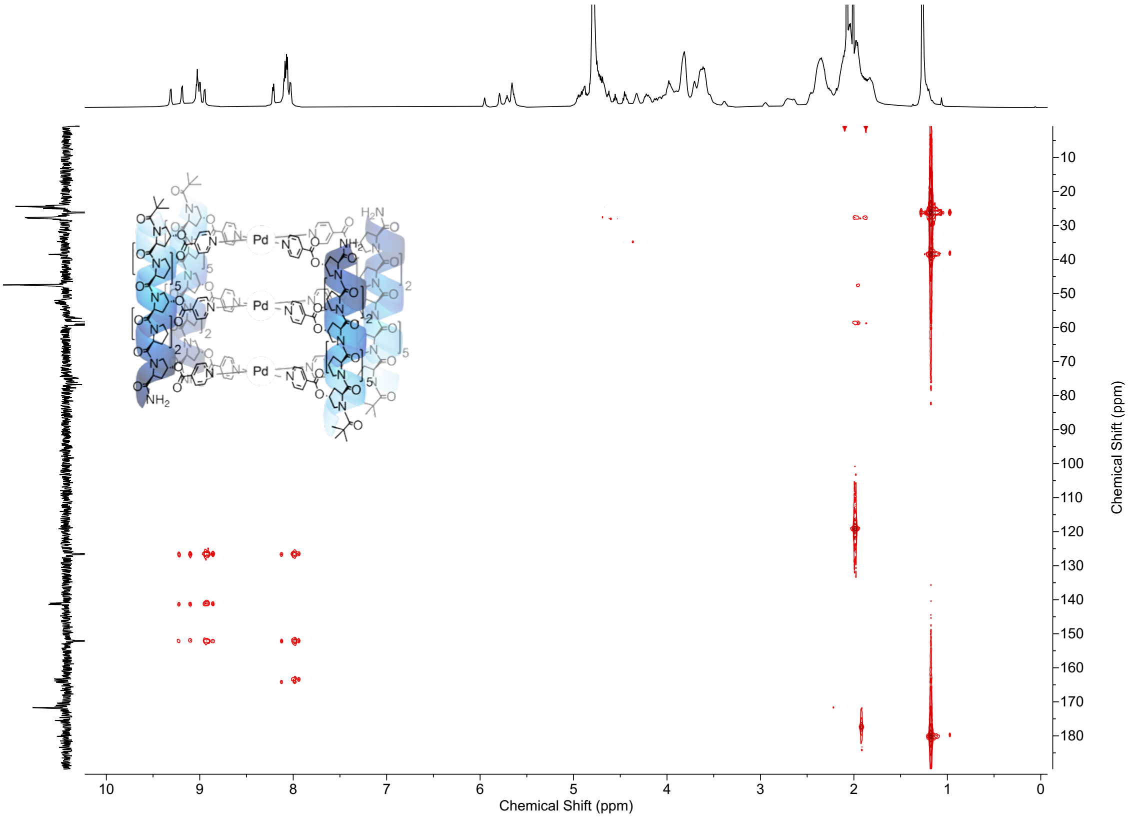

Figure S45. ^{1}H-^{13}C HMBC (D_2O, 600 MHz, 298 K) of **$Pd_3L^{7,R}_4(BF_4)_6$**.

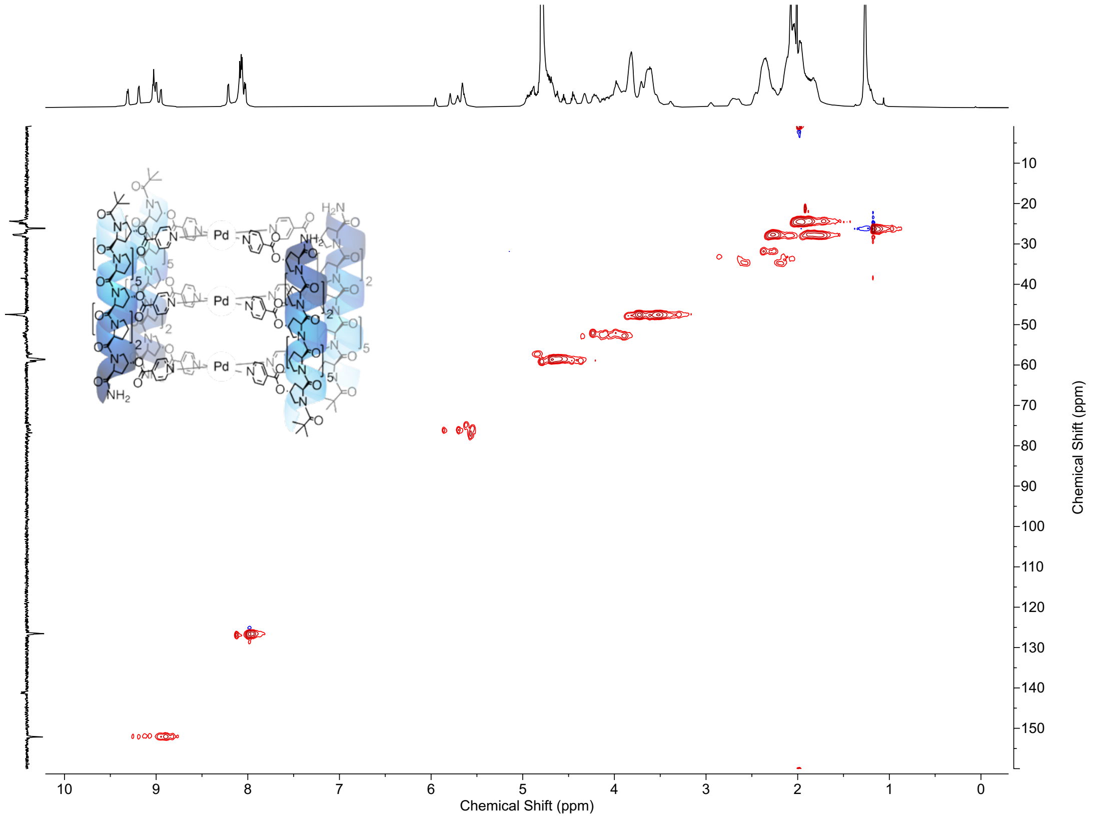

Figure S46. ^{1}H-^{13}C HSQC (D_2O, 600 MHz, 298 K) of **$Pd_3L^{7,R}_4(BF_4)_6$**.

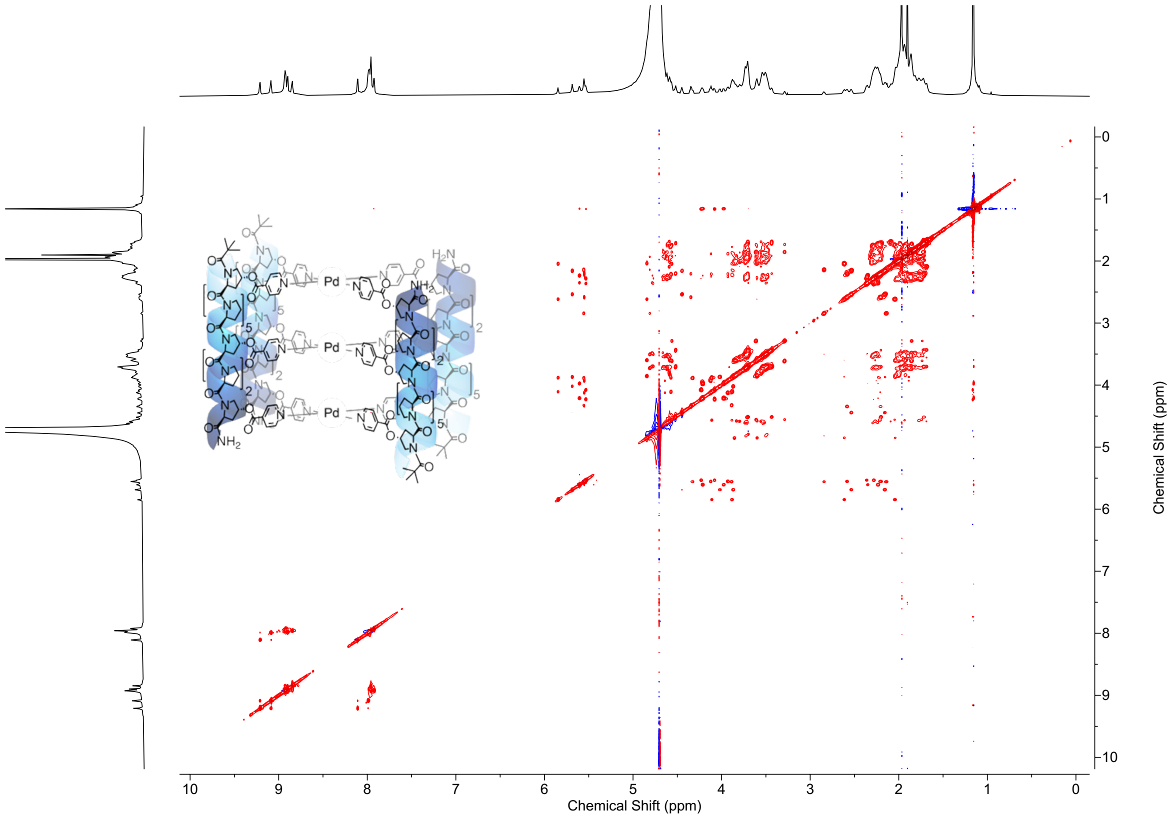

Figure S47. ^{1}H-^{1}H NOESY (D_2O, 600 MHz, 298 K, Mixing time = 0.300 s) of $\mathbf{Pd_3L^{7,R}_4}(BF_4)_6$.

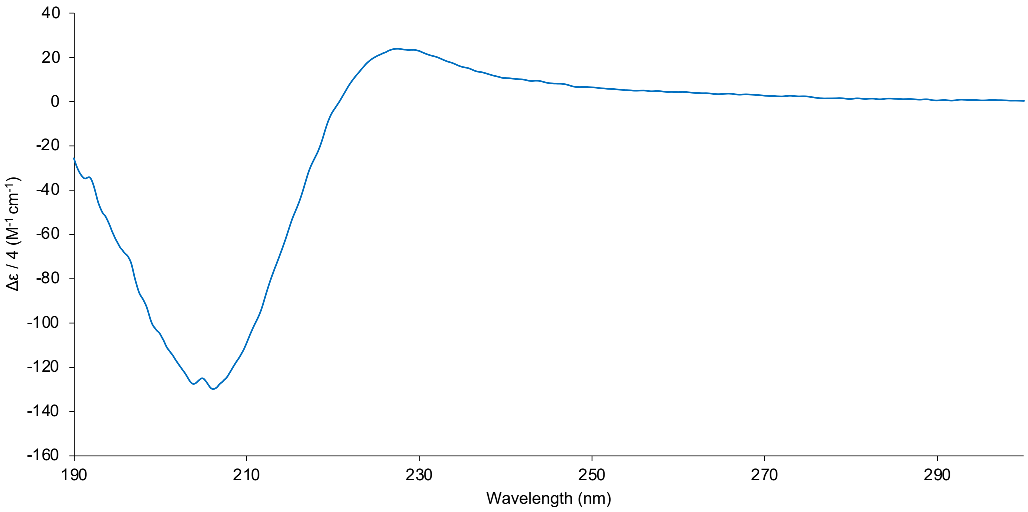

Figure S48. CD spectrum of $\mathbf{Pd_3L^{7,R}_4}(BF_4)_6$ in H_2O (293 K, 0.2002 mM, 0.1 mm) standardised to ligand concentration. Minima and maxima observed at 206 nm and 227 nm consistent with folding to the PPII helix retained from the ligand.

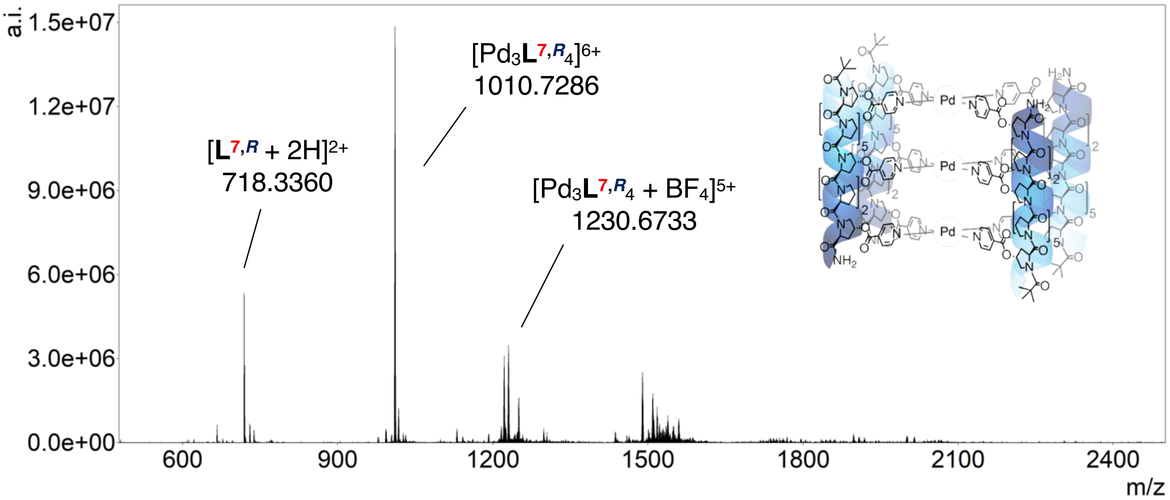

Figure S49. ESI-MS (+ve ion, H_2O/D_2O) of $\mathbf{Pd_3L}^{7,R}_{\mathbf{4}}(BF_4)_6$: m/z $[Pd_3\ \mathbf{L}^{7,R}_4]^{6+}$,1010.7286, $[Pd_3\mathbf{L}^{7,R}_4 + BF_4]^{5+}$, 1230.6733. Minor peaks around 1200 and 1500 predominantly consist of cages with varying anions.

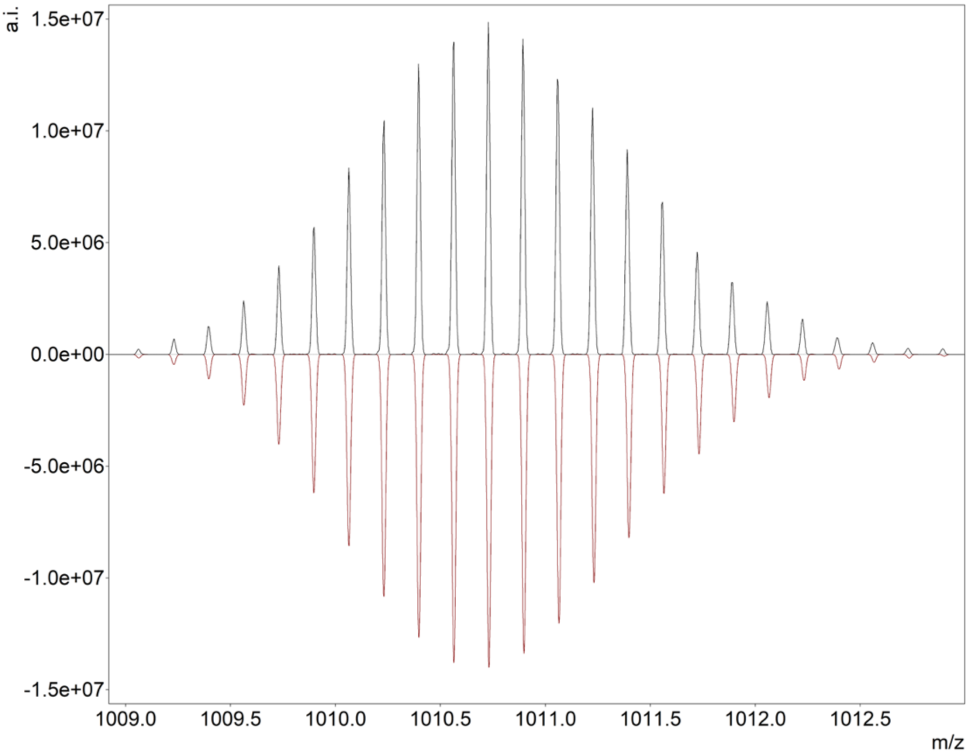

Figure S50. HRMS experimental (top) and calculated (bottom) isotope patterns of $[Pd_3\mathbf{L}^{7,R}_4]^{6+}$; m/z observed 1010.7286, calc. 1010.7213.

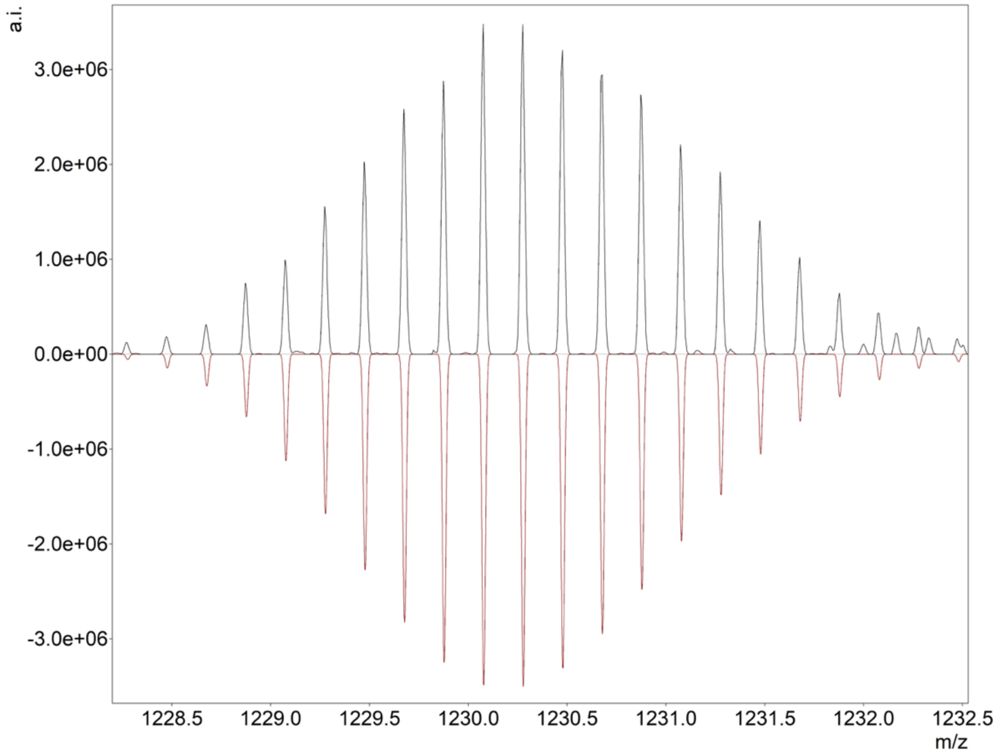

Figure S51. HRMS experimental (top) and calculated (bottom) isotope patterns of $[Pd_3\mathbf{L}^{7,R}{}_4 + BF_4]^{5+}$; m/z observed 1230.2764, calc. 1230.2781.

Metal-Peptidic Cage ***Pd$_3$L^{4,R}$_4$***

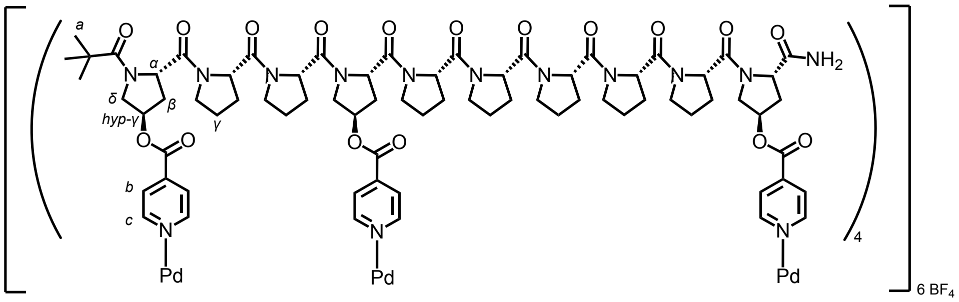

Pd$_3$L^{4,R}$_4$(BF$_4$)$_6$ was prepared by submitting **L^{4,R}** to the **General Cage Procedure**. This yielded 500 µL of **Pd$_3$L^{4,R}$_4$**(BF$_4$)$_6$ as a pale-yellow solution, at 0.538 mM, which was used without further purification.

Due to the complexity of the spectra, and the presence of all four head-to-tail isomers, unambiguous assignment of the ^{1}H NMR of this mixture of complexes was not possible.

^{1}H NMR (950 MHz, D$_2$O) δ 9.38 – 8.76 (m, 24H, H$_c$), 8.33 – 7.91 (m, 24H, H$_b$), 5.95 – 5.54 (m, 12H, H$_{hyp\text{-}\gamma}$), 5.10 – 3.45 (m, 120H, H$_\alpha$ and H$_\delta$), 2.82 – 1.51 (m, 136H, H$_\beta$ and H$_\gamma$), 1.41 – 1.06 (m, 36H, H$_a$). ^{13}C NMR (151 MHz, D$_2$O) δ 172.4, 172.2, 171.8, 171.7, 171.7, 170.7, 170.6, 163.9, 163.8, 163.7, 163.6, 163.6, 163.5, 152.2, 152.2, 152.1, 152.1, 152.0, 152.0, 151.9, 151.8, 141.3, 126.6, 126.5, 126.4, 126.3, 126.3, 86.2, 59.0, 58.9, 58.8, 58.8, 58.7, 58.6, 58.6, 58.5, 58.5, 58.3, 58.2, 47.9, 47.9, 47.8, 47.7, 47.7, 47.6, 47.5, 47.5, 47.4, 47.4, 47.3, 47.2, 47.1, 28.1, 28.0, 27.9, 27.9, 27.7, 27.6, 27.5, 27.5, 27.4, 26.3, 26.2, 26.1, 24.7, 24.7, 24.6, 24.5, 24.4, 24.3, 24.2, 24.2. ESI-MS: m/z [Pd$_3$**L^{4,R}**$_4$]$^{6+}$,1010.2244, [Pd$_3$**L^{4,R}**$_4$ + BF$_4$]$^{5+}$, 1230.6733, [Pd$_3$**L^{4,R}**$_4$ + 2BF$_4$]$^{4+}$ 1558.8378. HRMS m/z observed 1010.2244, calc. 1010.2276 (Figure S60).

This cage species in particular suffers from low resolution of the ^{13}C spectra as it is a reasonably equimolar mixture of isomers and so relative concentration is quartered. This, combined with the difficulties due to relaxation time, limit the carbon spectra.

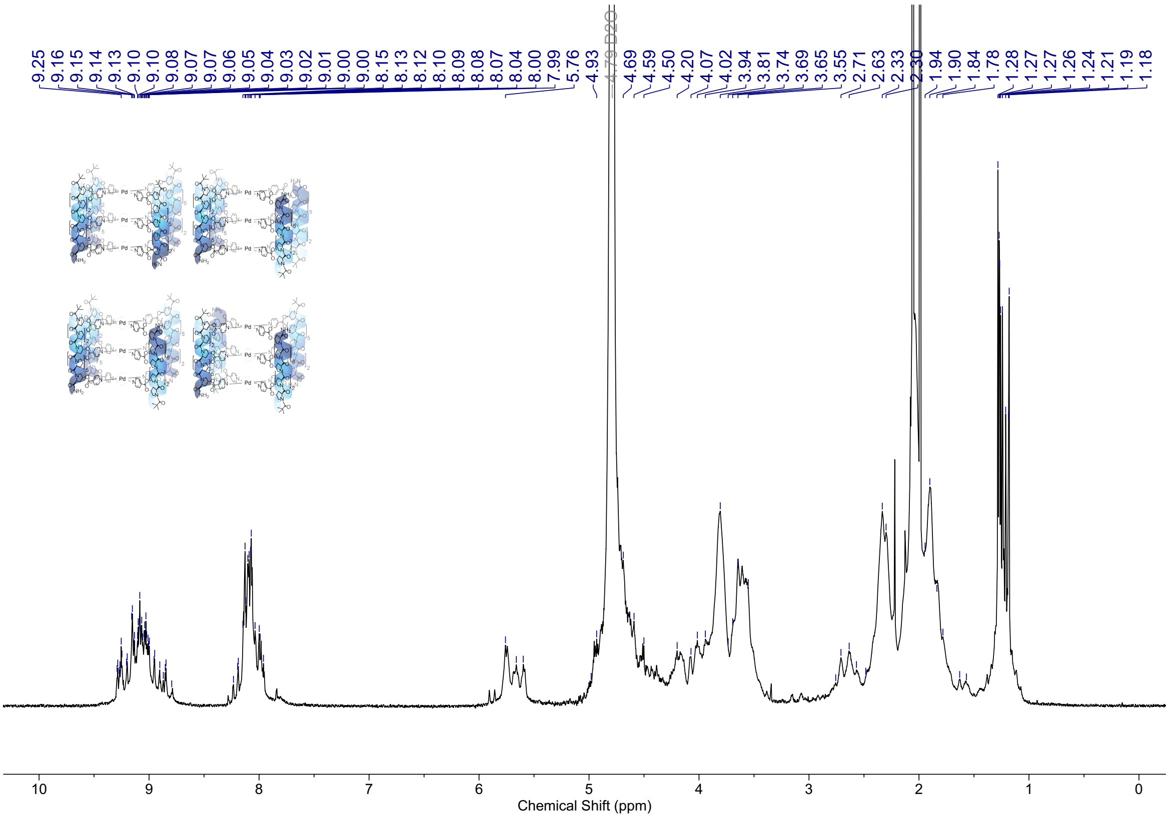

Figure S52. ^{1}H NMR (D_2O, 950 MHz, 298 K) of **Pd_3L**^{4,R}**$_4$**$(BF_4)_6$.

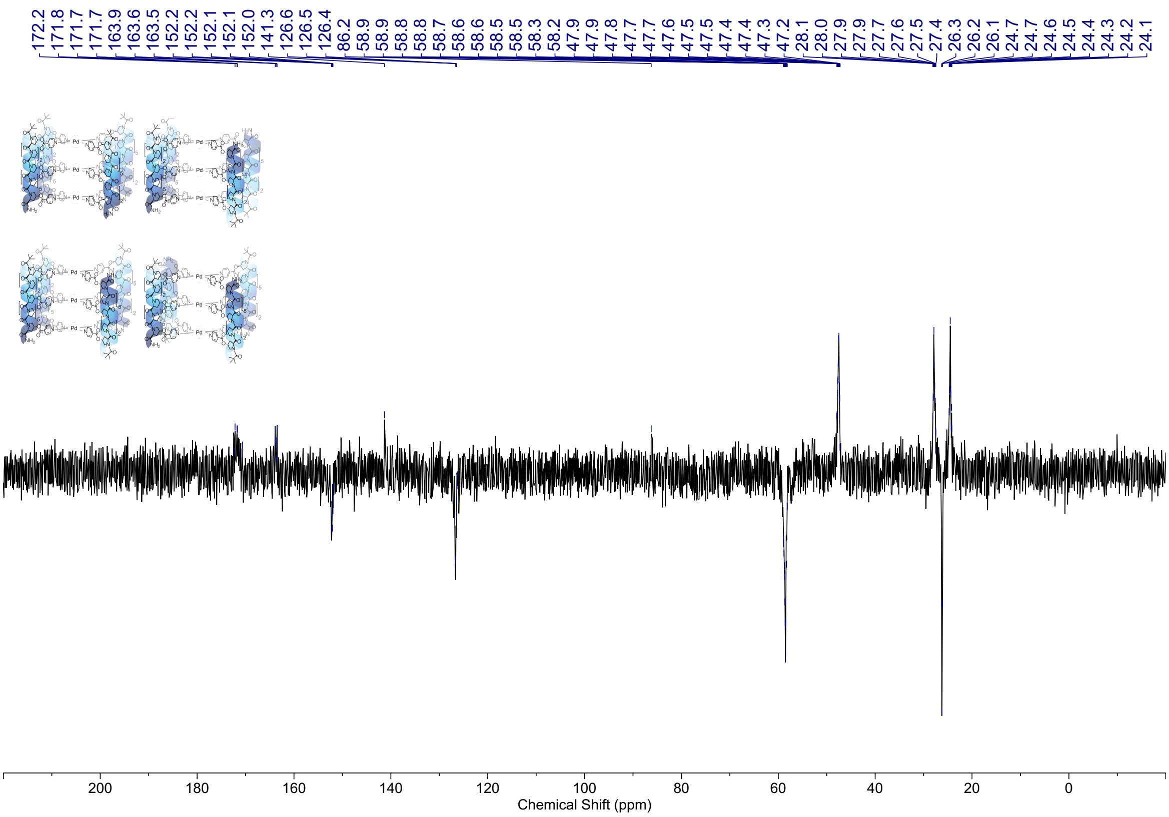

Figure S53. ^{13}C JMOD (D_2O, 151 MHz, 298 K) of **Pd_3L**^{4,R}**$_4$**$(BF_4)_6$.

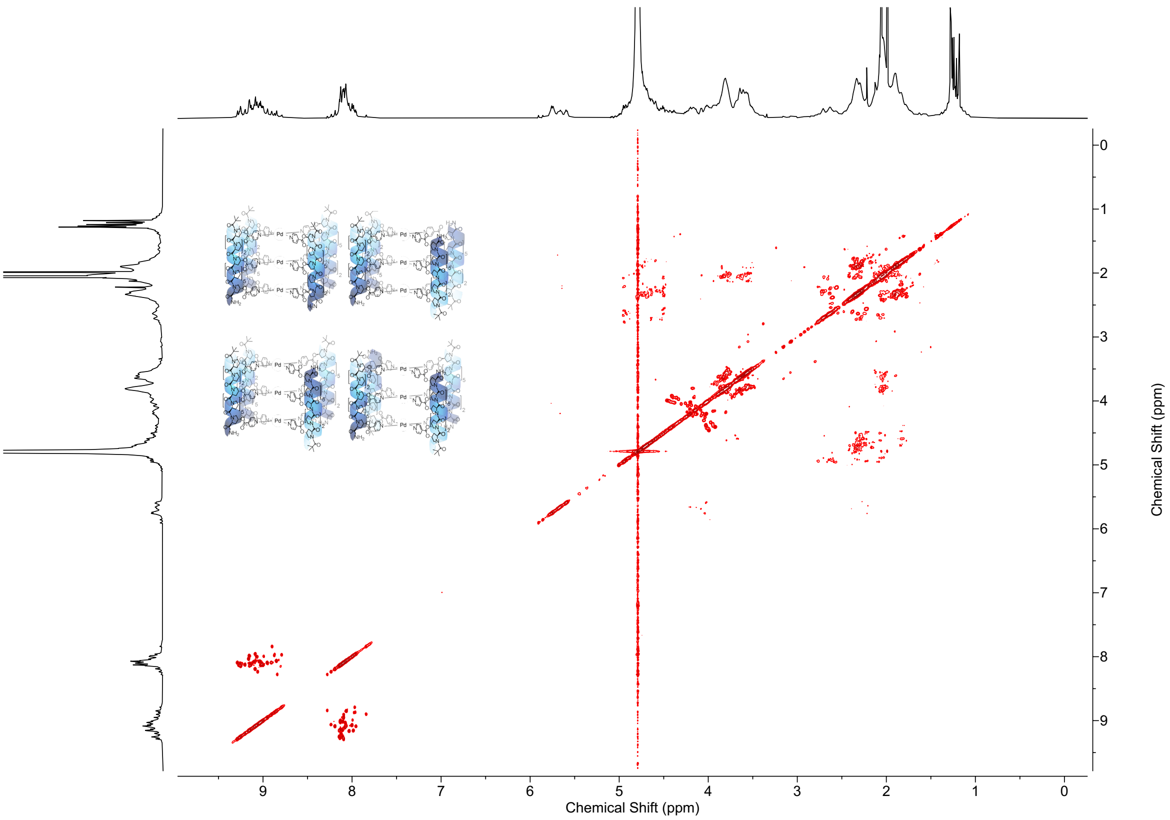

Figure S54. ^{1}H-^{1}H COSY (D_2O, 600 MHz, 298 K) of $\mathbf{Pd_3L^{4,R}_4}(BF_4)_6$.

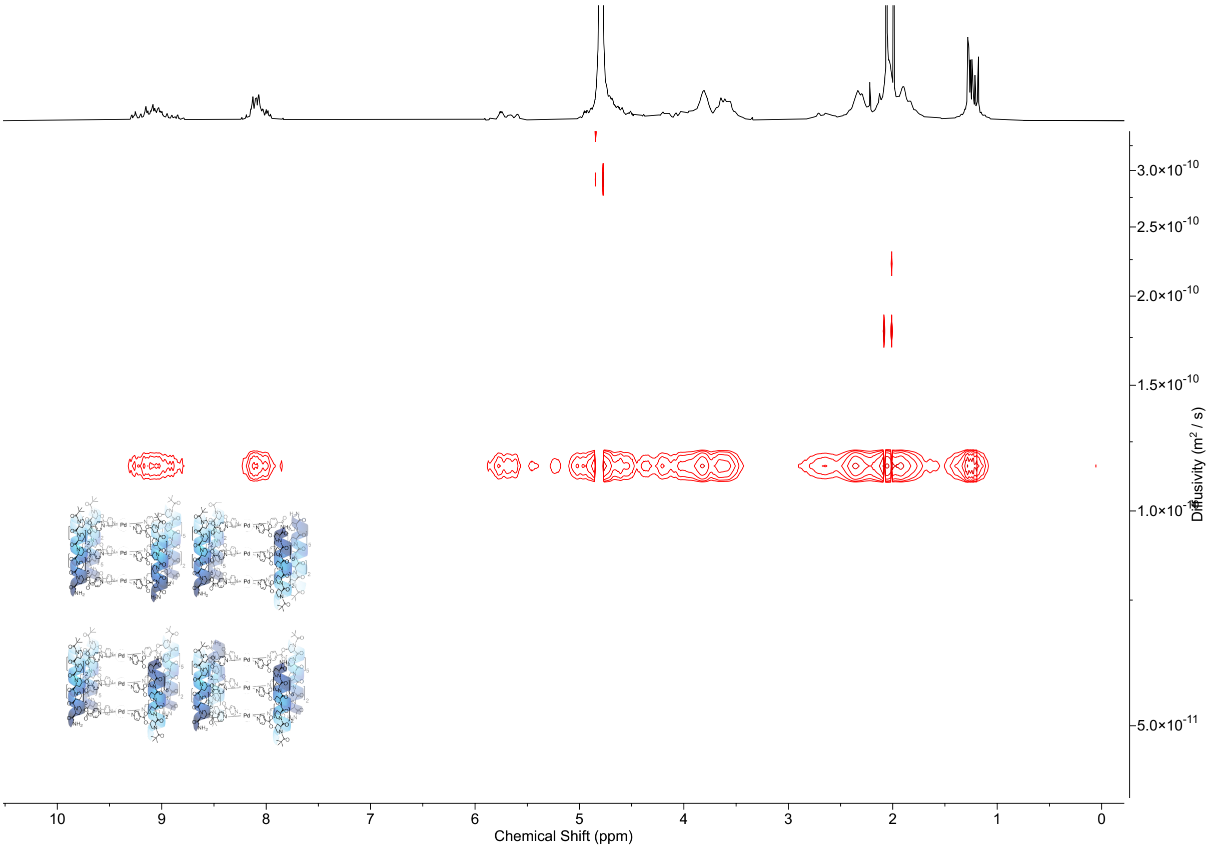

Figure S55. ^{1}H DOSY (D_2O, 600 MHz, 298 K) of $\mathbf{Pd_3L^{4,R}_4}(BF_4)_6$. The diffusion coefficient of $\mathbf{Pd_3L^{4,R}_4}(BF_4)_6$ was 1.155 x 10^{-9} m^2 s^{-1}, giving a hydrodynamic radius of 28.3 Å.

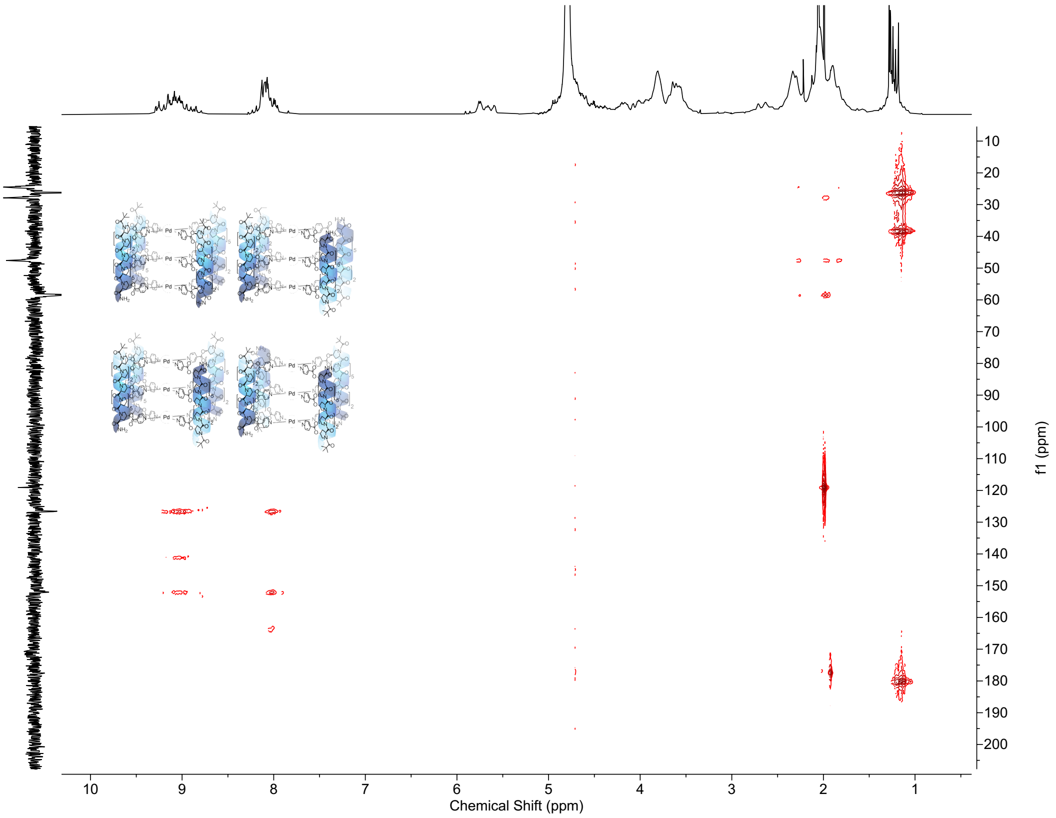

Figure S56. ¹H-¹³C HMBC (D_2O, 600 MHz, 298 K) of $\mathbf{Pd_3L^{4,R}_4}(BF_4)_6$.

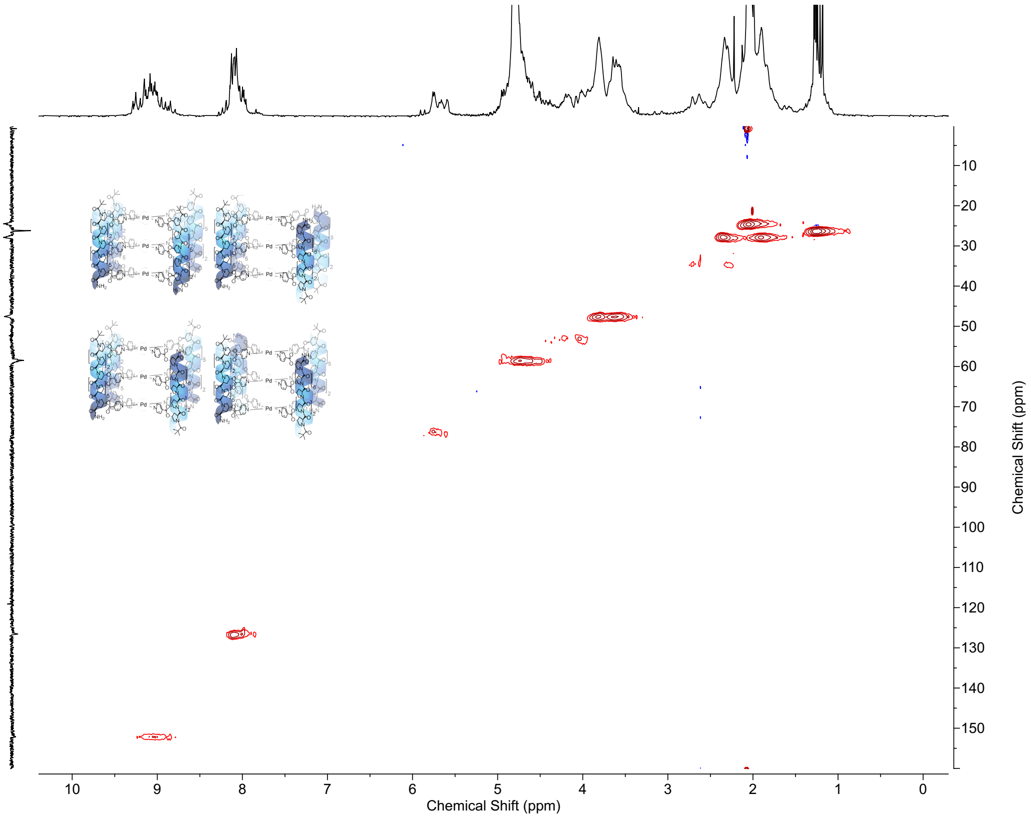

Figure S57. ¹H-¹³C HSQC (D_2O, 600 MHz, 298 K) of $\mathbf{Pd_3L^{4,R}_4}(BF_4)_6$.

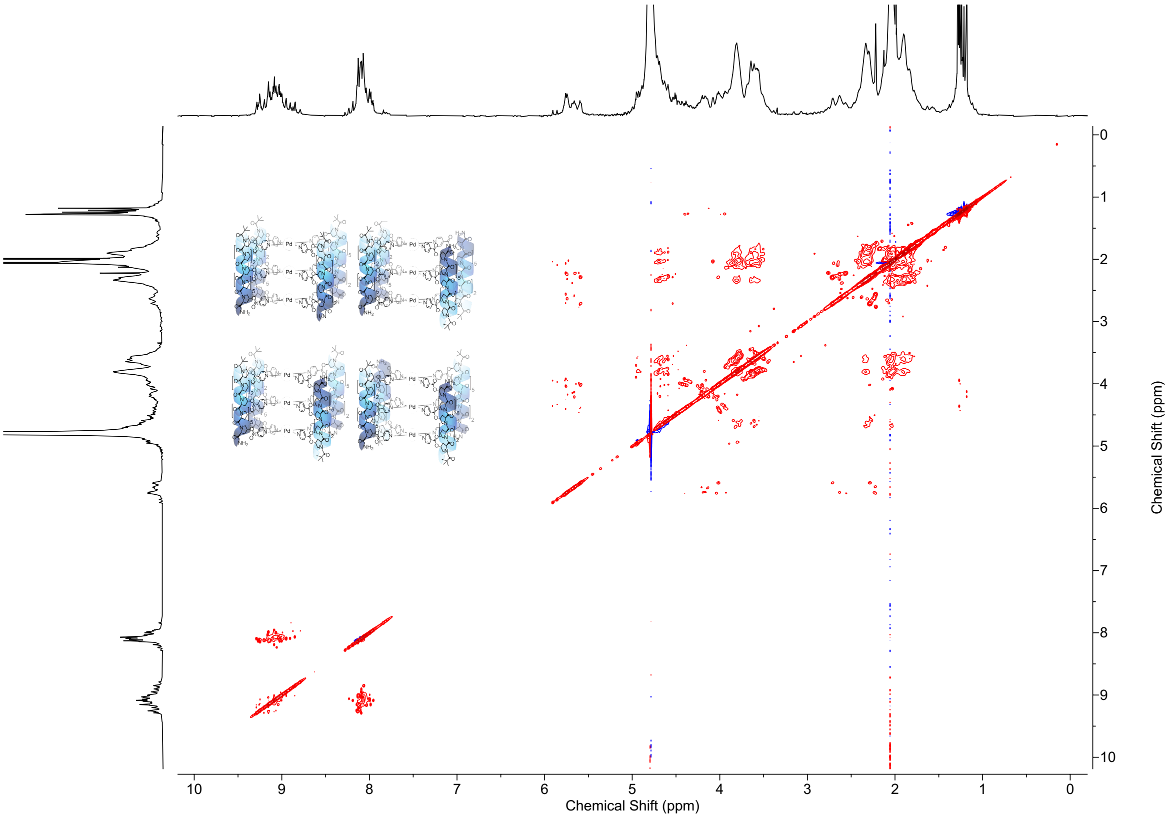

Figure S58. ^{1}H-^{1}H NOESY (D_2O, 600 MHz, 298 K) of **Pd$_3$L^{4,R}$_4$**$(BF_4)_6$.

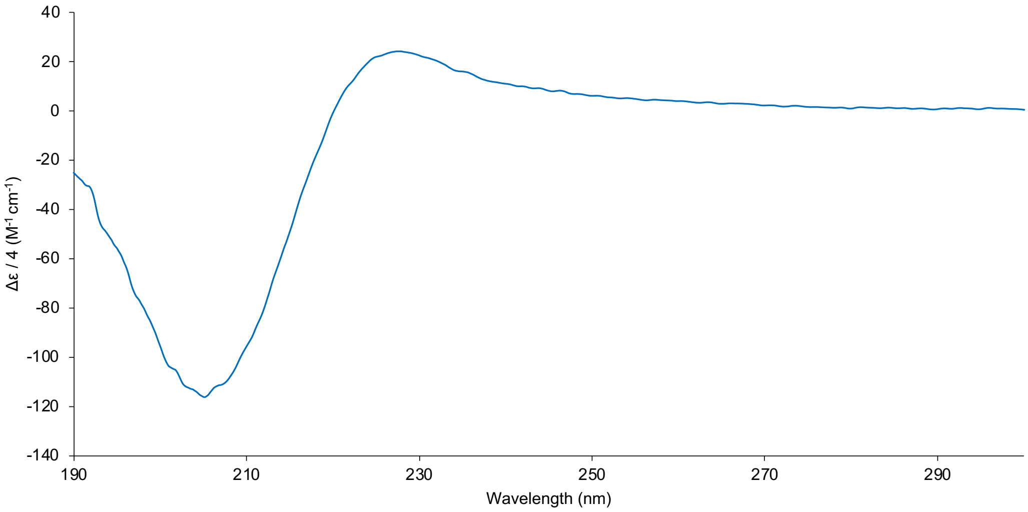

Figure S59. CD spectrum of **Pd$_3$L^{4,R}$_4$**$(BF_4)_6$ in H_2O (293 K, 0.2000 mM, 0.1 mm) standardised to ligand concentration. Minima and maxima observed at 205 nm and 228 nm consistent with folding to the PPII helix.

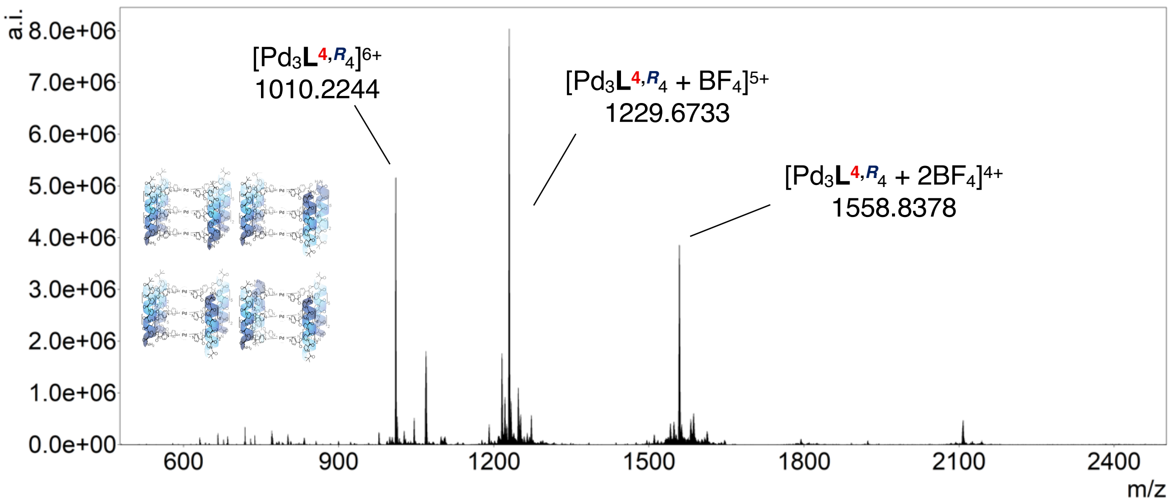

Figure S60. ESI-MS (+ve ion, H_2O/D_2O) of **$Pd_3L^{4,R}{}_4$**$(BF_4)_6$; m/z $[Pd_3\mathbf{L}^{4,R}{}_4]^{6+}$,1010.2244, $[Pd_3\mathbf{L}^{4,R}{}_4 + BF_4]^{5+}$, 1230.6733, $[Pd_3\mathbf{L}^{4,R}{}_4 + 2BF_4]^{4+}$ 1558.8378.

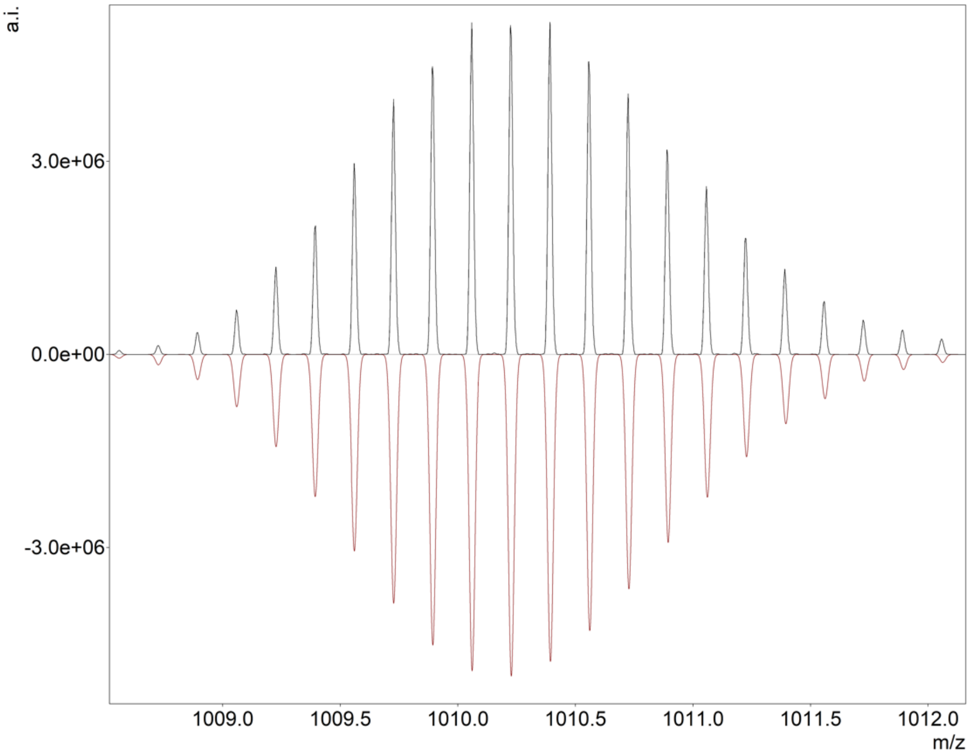

Figure S61. HRMS experimental (top) and calculated (bottom) isotope patterns of $[Pd_3\mathbf{L}^{4,R}{}_4]^{6+}$; m/z observed 1010.2244, calc. 1010.2276.

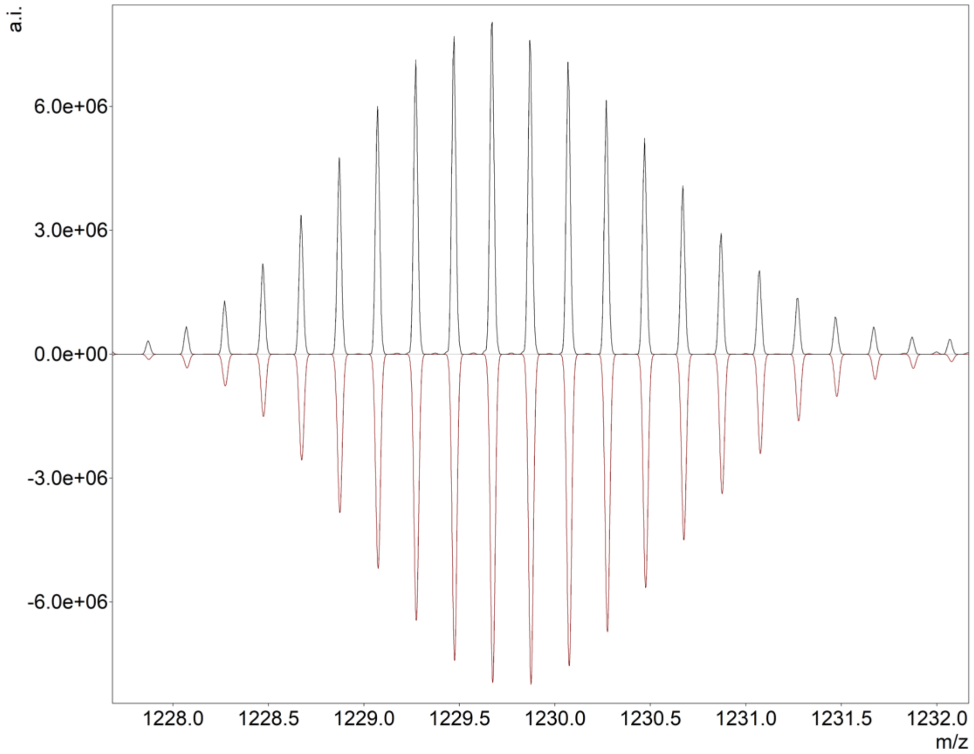

Figure S62. HRMS experimental (top) and calculated (bottom) isotope patterns of $[Pd_3\mathbf{L}^{4,\mathit{R}}_4 + BF_4]^{5+}$; m/z observed 1229.6733, calc. 1229.6735.

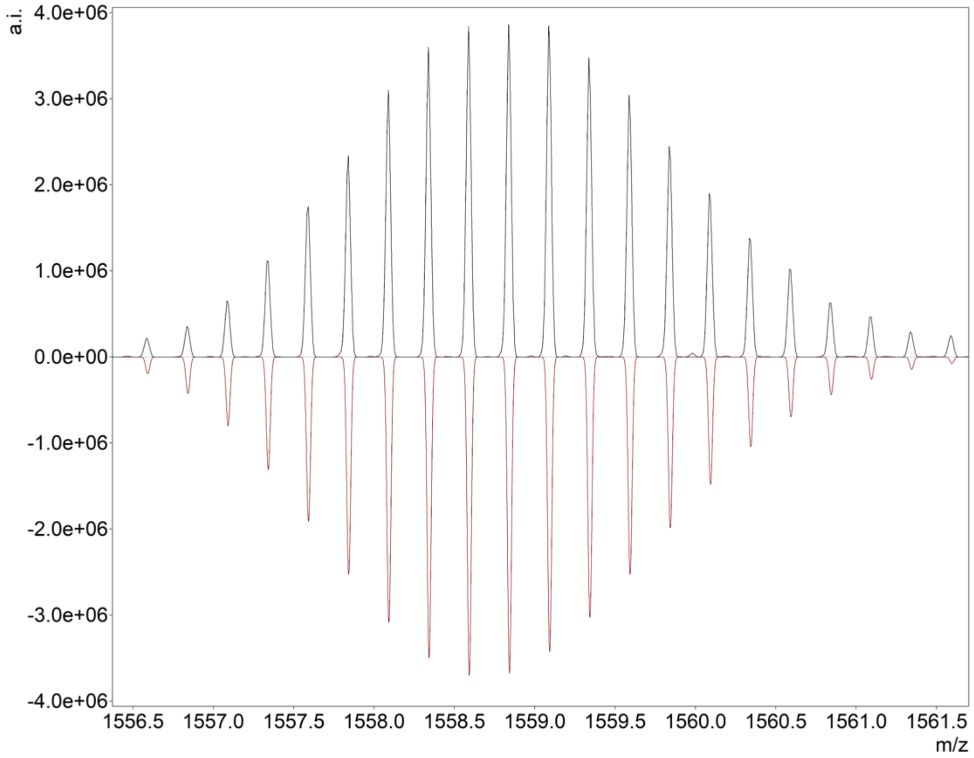

Figure S63. HRMS experimental (top) and calculated (bottom) isotope patterns of $[Pd_3\mathbf{L}^{4,\mathit{R}}_4 + 2BF_4]^{4+}$; m/z observed 1558.8378, calc. 1558.8430.

In order to probe the isomer distribution with relation to temperature the synthesis was repeated at 5 °C, and also a sample of **8**$(BF_4)_6$ was submitted to V.T. NMR. Neither of these showed significant deviation to the isomeric distribution recorded at r.t., but rather shifts in ppm.

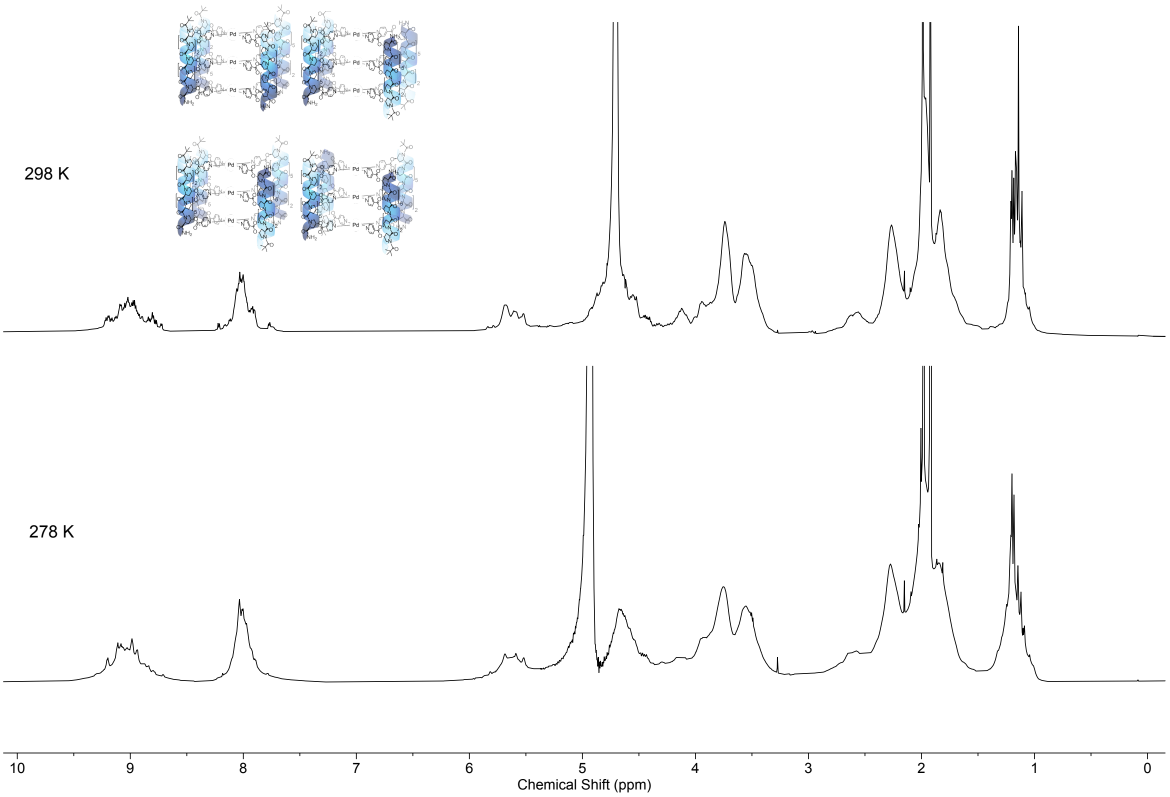

Figure S64. Stacked ^1H NMR (D_2O, 600 MHz) of cage **$Pd_3L^{4,R}_4$**$(BF_4)_6$ after incubation for five days at 298 K (top) and 278 K (bottom). ^1H NMR for each sample was recorded at the same temperature as the incubation period, and samples maintained at incubation temperature when transferring to NMR machine.

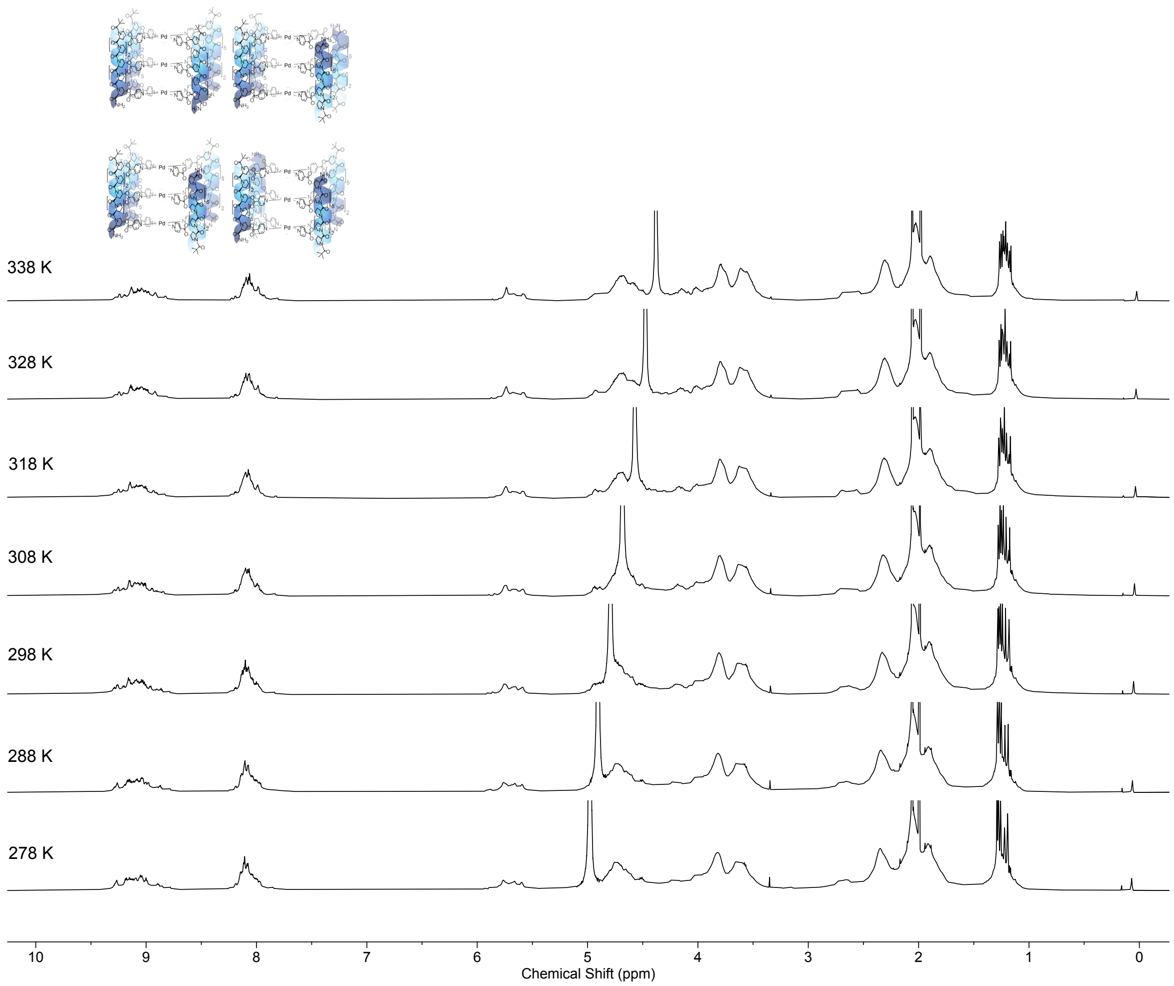

Figure S65. Stacked ^{1}H NMR (D_2O, 600 MHz) of cage $\mathbf{Pd_3L^{4,R}_4}(BF_4)_6$ at 338K, 328 K, 318 K, 308 K, 298 K, 288 K and 278 K, listed in order from top of spectra to bottom. Signal have been references to residual CH_3CN due to temperature variation of D_2O signal.

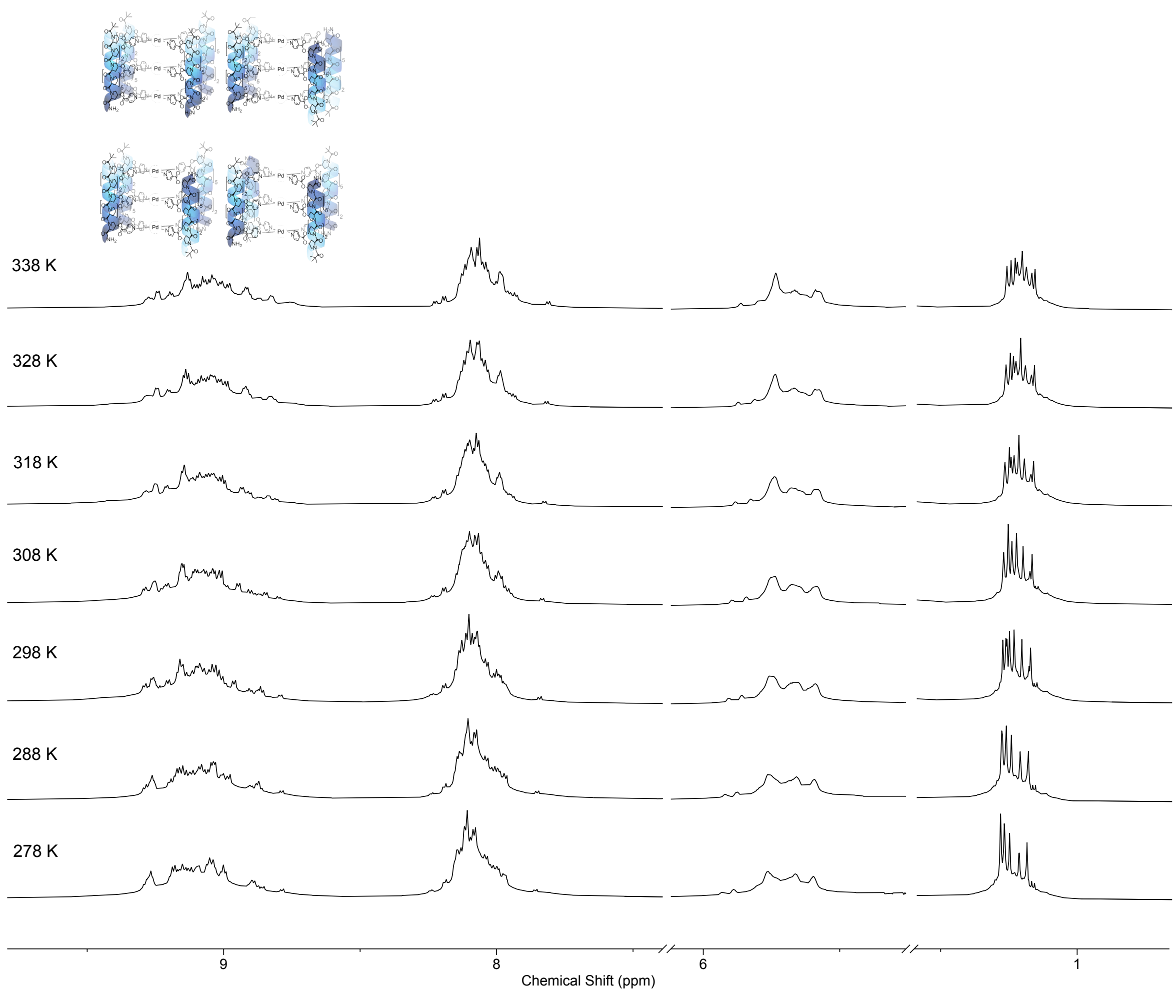

Figure S66. Partial stacked ^{1}H NMR (D_2O, 600 MHz) of cage **$Pd_3L^{4,R}_4$**$(BF_4)_6$ at 338K, 328 K, 318 K, 308 K, 298 K, 288 K and 278 K, listed in order from top of spectra to bottom. Signal have been references to residual CH_3CN due to temperature variation of D_2O signal. Signals at ~1 ppm have been scaled independently to the rest of the spectrum due to relative intensity to show splitting of *t*Bu peak into two environments.

*Metal-Peptidic Cage [**$Pd_3L^{7,S}{}_4$**$(BF_4)_6]_2$*

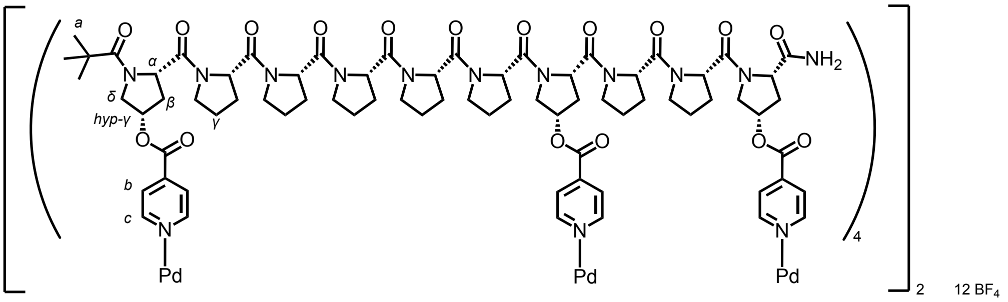

[**$Pd_3L^{7,S}{}_4$**$(BF_4)_6]_2$ was prepared by submitting **$L^{7,S}$** to the **General Cage Procedure**. This yielded 500 µL of [**$Pd_3L^{7,S}{}_4$**$(BF_4)_6]_2$ as a pale-yellow solution, at 0.873 mM, which was used without further purification.

Due to the broadening of the spectra, unambiguous assignment of the ^1H NMR signals in this interpenetrated species was not possible.

^1H NMR (950 MHz, D_2O) δ 9.47 – 8.75 (m, 48H, H_c), 8.26 – 7.79 (m, 48H, H_b), 5.93 – 5.44 (m, 24H, $H_{hyp\text{-}\gamma}$), 5.10 – 3.32 (m, 240H, H_α, and H_δ), 2.93 – 1.60 (m, 272H, H_β, and H_γ), 1.33 – 1.06 (m, 72H, H_a). ^{13}C NMR (151 MHz, D_2O) δ 172.6, 172.6, 172.5, 172.5, 172.2, 172.1, 171.9, 171.9, 171.8, 171.7, 171.7, 171.6, 171.5, 171.5, 171.4, 171.4, 171.4, 171.3, 171.3, 170.8, 168.8, 163.7, 163.6, 163.5, 163.4, 163.3, 163.2, 163.1, 162.2, 158.5, 158.4, 158.3, 152.3, 152.3, 152.2, 152.2, 152.1, 152.0, 152.0, 151.9, 151.9, 151.8, 151.8, 151.7, 151.7, 151.6, 141.4, 141.4, 141.3, 141.2, 141.1, 126.9, 126.9, 126.8, 126.8, 126.8, 126.7, 126.7, 126.7, 126.6, 126.6, 126.6, 126.5, 126.4, 126.3, 126.3, 126.2, 126.1, 126.1, 126.1, 126.0, 119.2, 119.1, 59.5, 59.5, 59.0, 59.0, 58.8, 58.7, 58.6, 58.6, 58.5, 58.5, 58.4, 58.4, 58.3, 58.2, 58.1, 58.0, 57.8, 57.8, 47.9, 47.8, 47.8, 47.7, 47.6, 47.6, 47.5, 47.5, 47.4, 47.3, 47.3, 47.2, 47.1, 47.0, 46.9, 28.2, 28.1, 28.1, 27.9, 27.7, 27.6, 27.5, 26.5, 26.4, 26.4, 26.4, 26.3, 26.3, 26.3, 26.2, 26.2, 26.1, 25.1, 25.0, 24.9, 24.8, 24.7, 24.7, 24.7, 24.6, 24.5, 24.4, 24.2, 24.1, 24.1, 24.0, 24.0, 23.9. ESI-MS: m/z $[Pd_3$**$L^{7,S}$**${}_4]^{6+}$,1010.5648, $[Pd_3$**$L^{7,S}$**${}_4\ BF_4]^{5+}$/$[Pd_6$**$L^{7,S}$**${}_8$ + $2BF_4]^{10}$, 1229.9780, $[Pd_6$**$L^{7,S}$**${}_8$ + $2BF_4]^{10+}$ 1376.5330, $[Pd_6$**$L^{7,S}$**${}_8$ + $4BF_4]^{8+}$ 1559.5964, $[Pd_6$**4**$_8$ + $5BF_4]^{7+}$, 1794.5378. HRMS: m/z observed 1794.5378, calc. 1794.5400 (Figure S75).

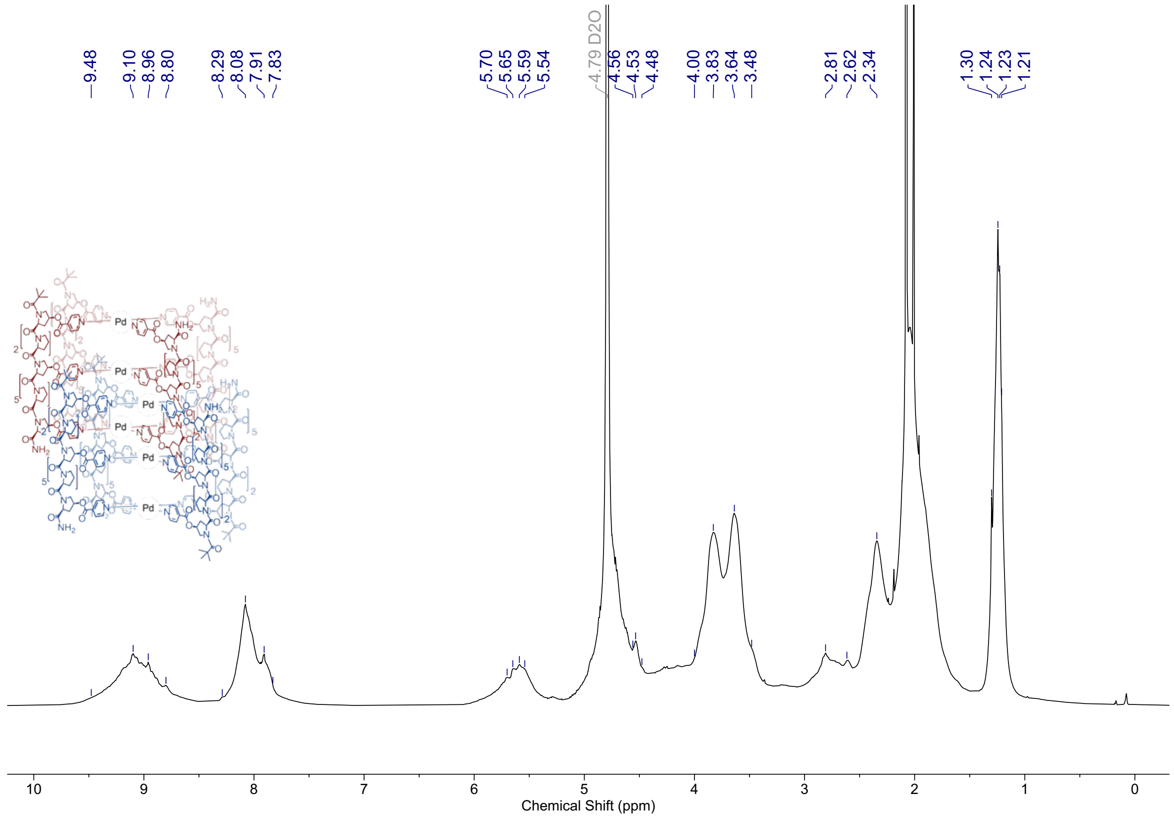

Figure S67. ^{1}H NMR (D_2O, 600 MHz, 298 K) of [$\mathbf{Pd_3L^{7,S}_4}$$(BF_4)_6$]$_2$.

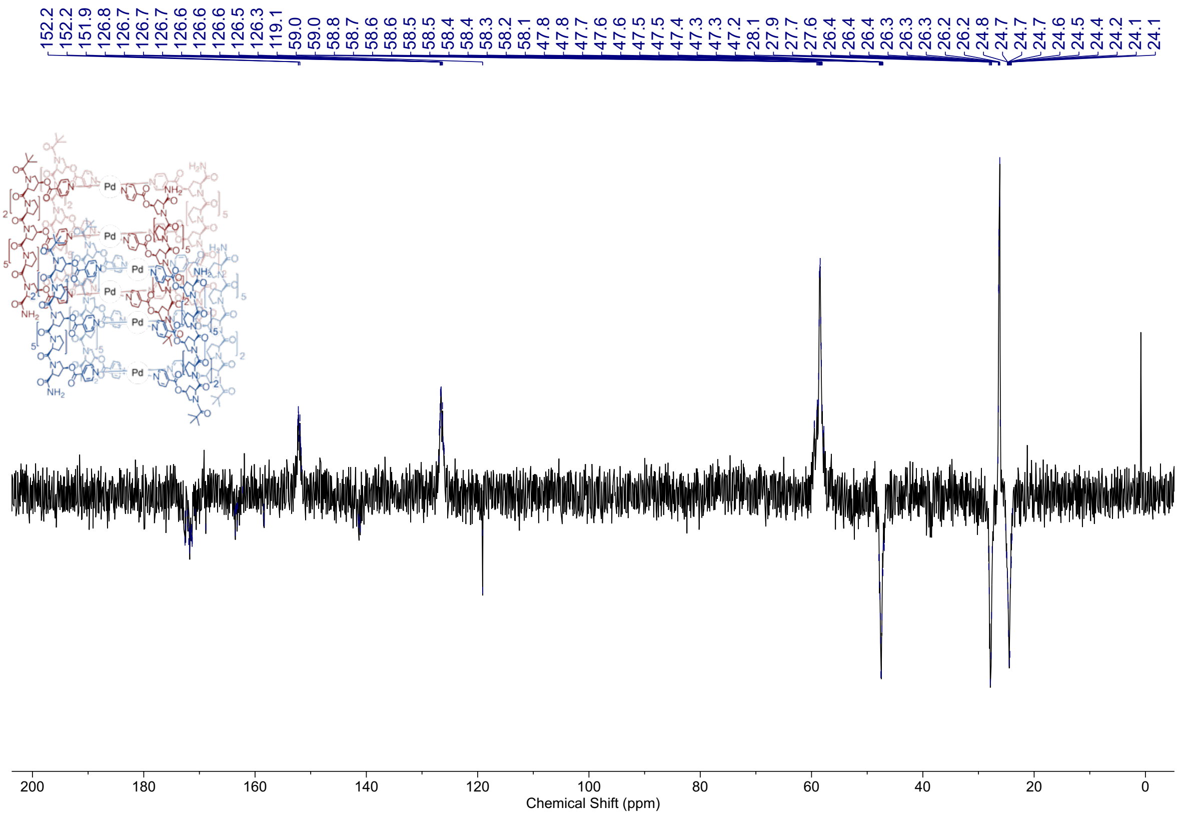

Figure S68. ^{13}C JMOD (D_2O, 151 MHz, 298 K) of [$\mathbf{Pd_3L^{7,S}_4}$$(BF_4)_6$]$_2$.

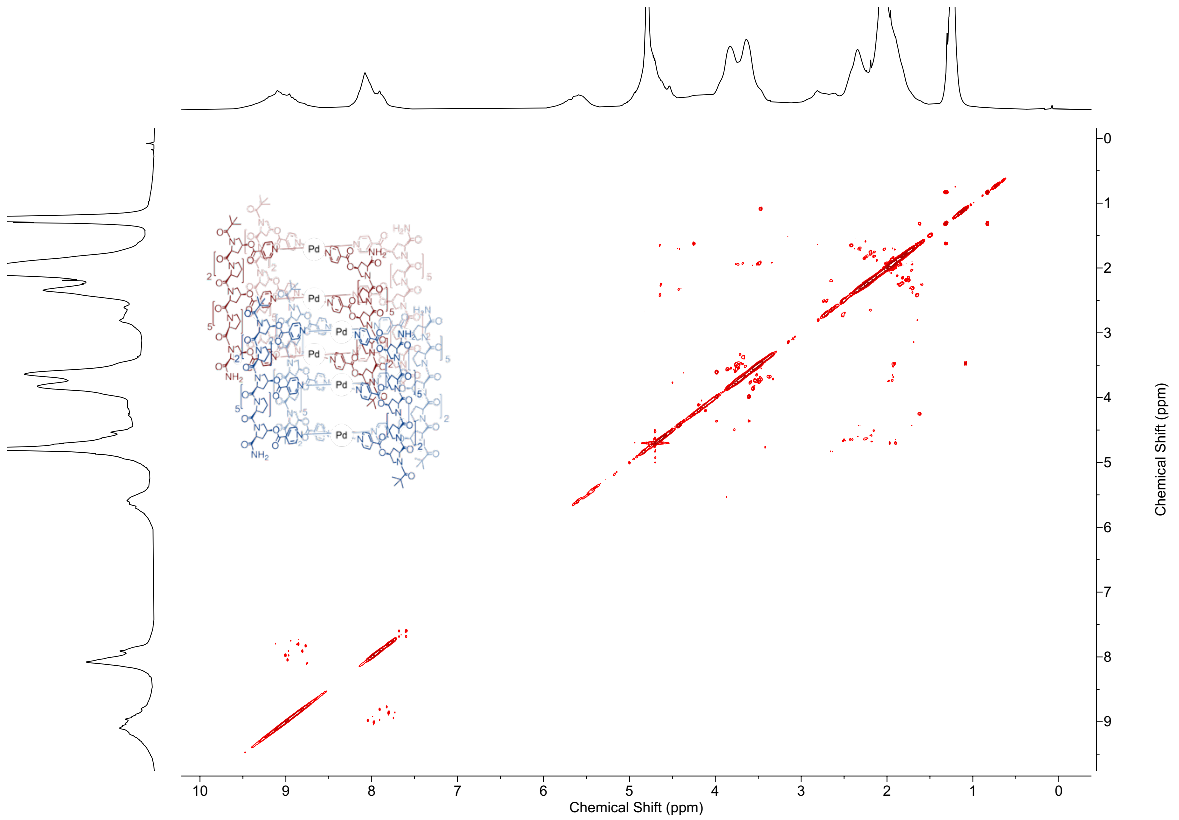

Figure S69. ^1H-^1H COSY (D_2O, 600 MHz, 298 K) of **$[Pd_3L^{7,S}_4(BF_4)_6]_2$**.

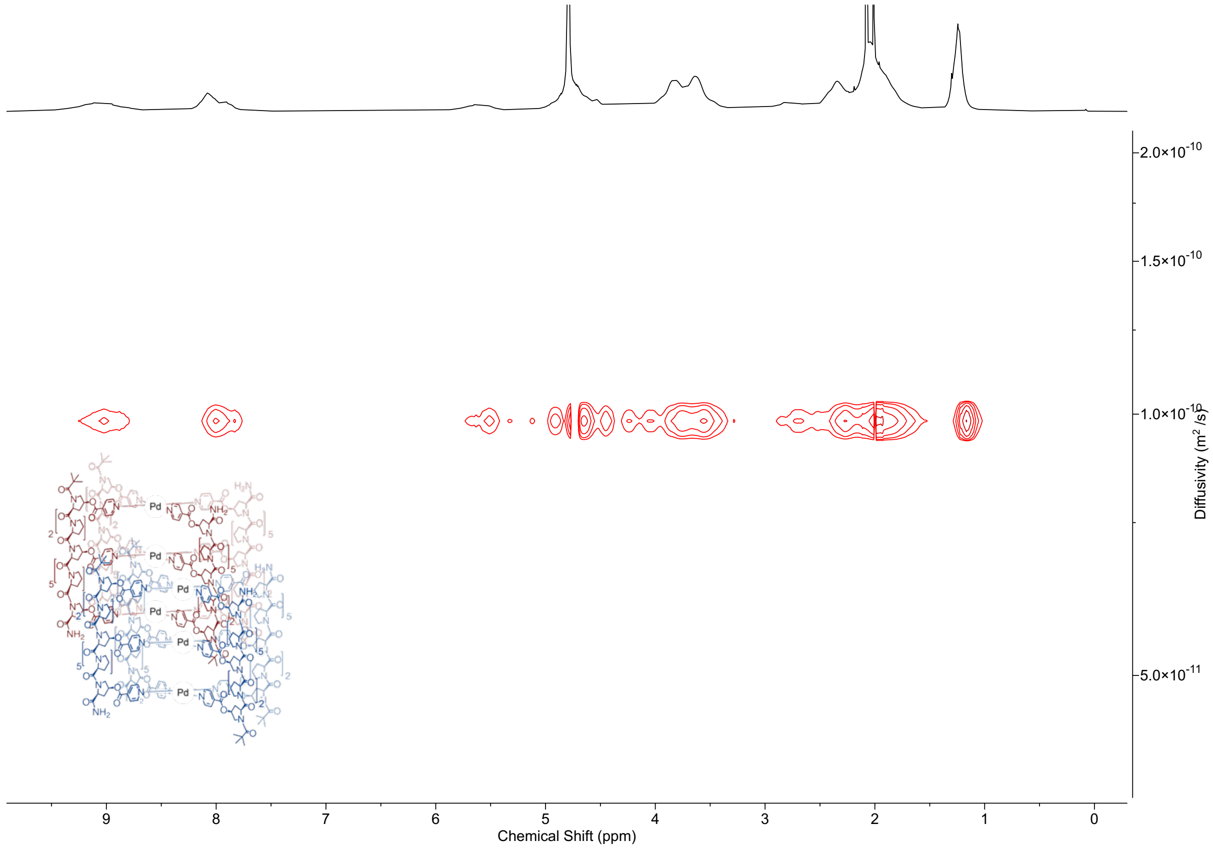

Figure S70. ^1H DOSY (D_2O, 600 MHz, 298 K) of **$[Pd_3L^{7,S}_4(BF_4)_6]_2$**.The diffusion coefficient of **$[Pd_3L^{7,S}_4(BF_4)_6]_2$** was 9.820 x 10^{-10} m^2 s^{-1}, giving a hydrodynamic radius of 33.3 Å.

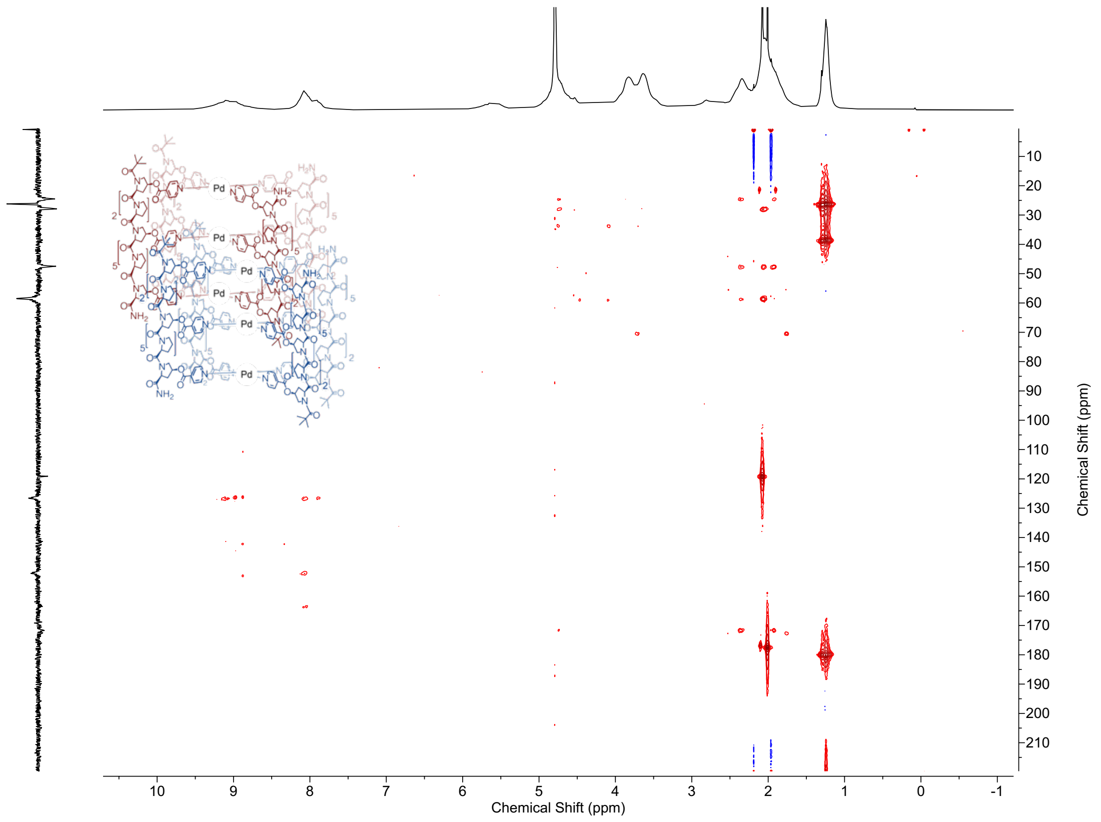

Figure S71. ¹H-¹³C HMBC (D_2O, 600 MHz, 298 K) of [**$Pd_3L^{7,S}{}_4$**$(BF_4)_6$]$_2$.

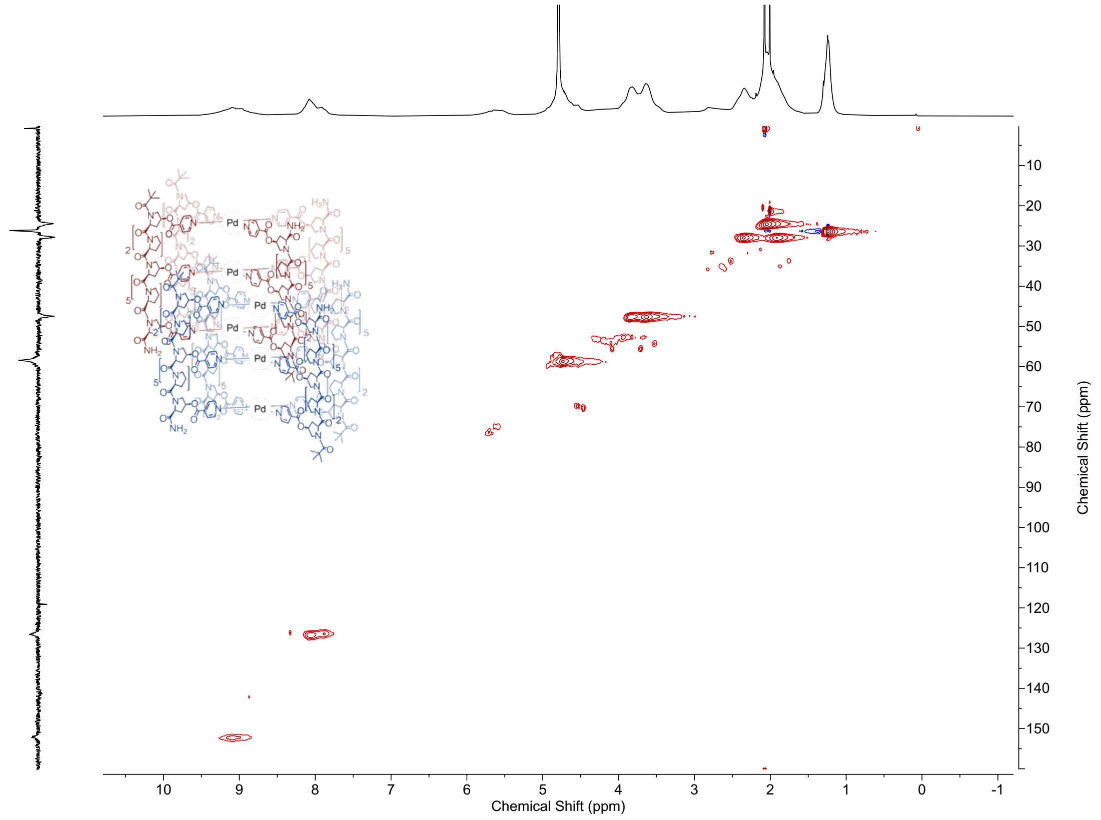

Figure S72. ¹H-¹³C HSQC (D_2O, 600 MHz, 298 K) of [**$Pd_3L^{7,S}{}_4$**$(BF_4)_6$]$_2$.

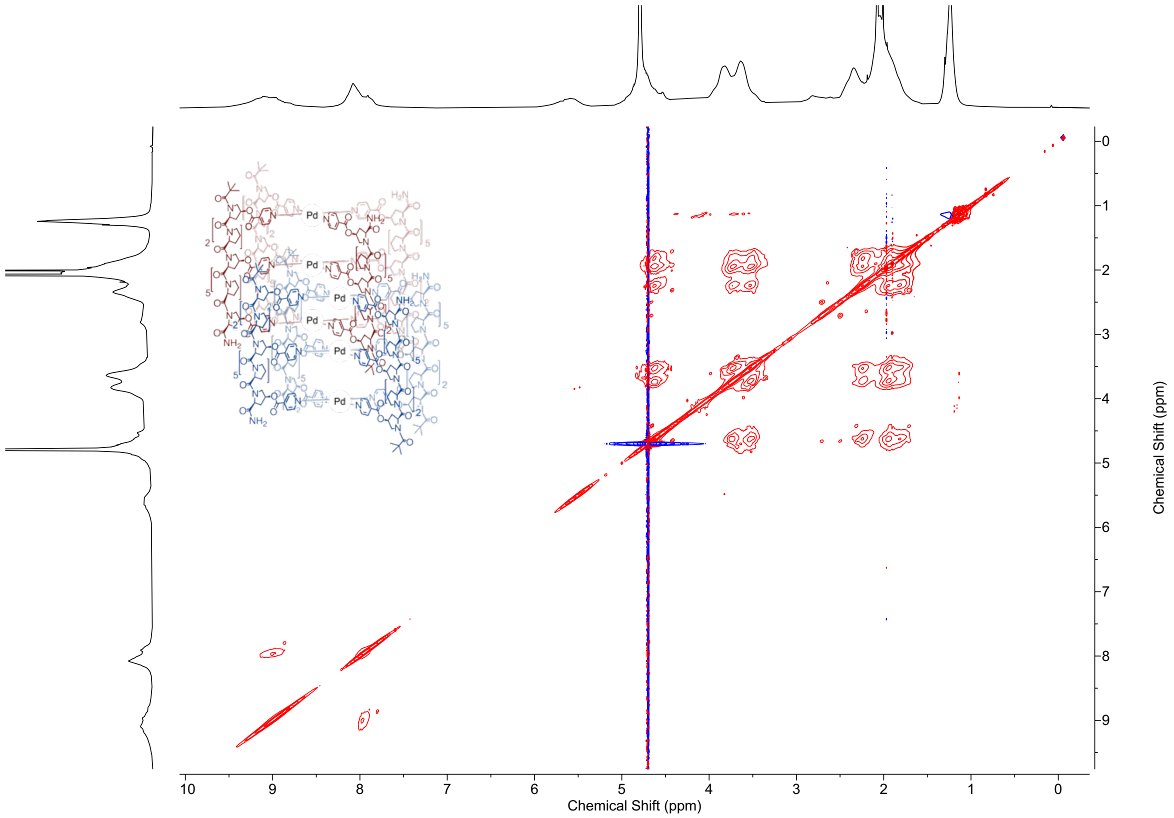

Figure S73. ^1H-^1H NOESY (D_2O, 600 MHz, 298 K, Mixing time = 0.300 s) of **$[Pd_3L^{7,S}_4(BF_4)_6]_2$**.

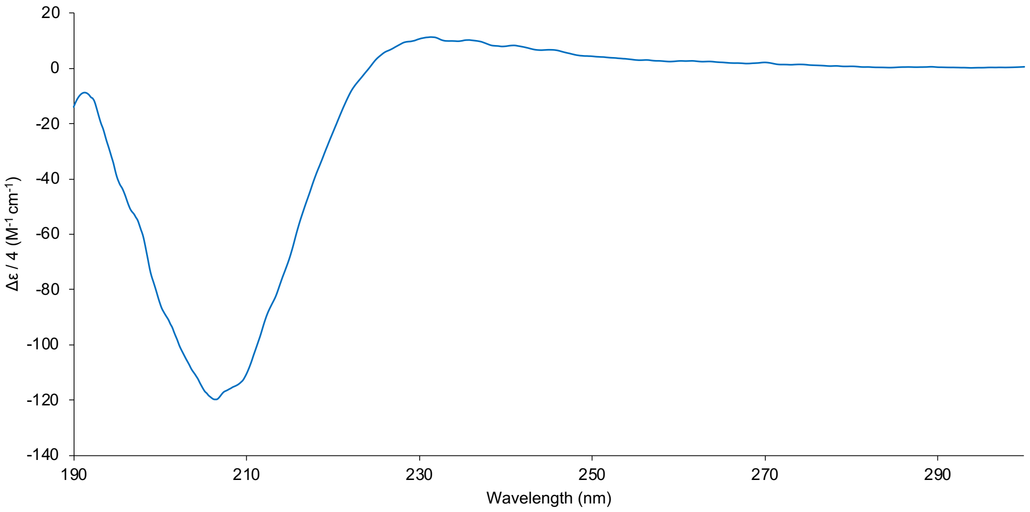

Figure S74. CD spectrum of **$[Pd_3L^{7,S}_4(BF_4)_6]_2$** in H_2O (293 K, 0.1980 mM, 0.1 mm) standardised to ligand concentration. Minima and maxima observed at 207 nm and 231 nm. Reduction in intensity of maximum consistent with decrease in PPII character.

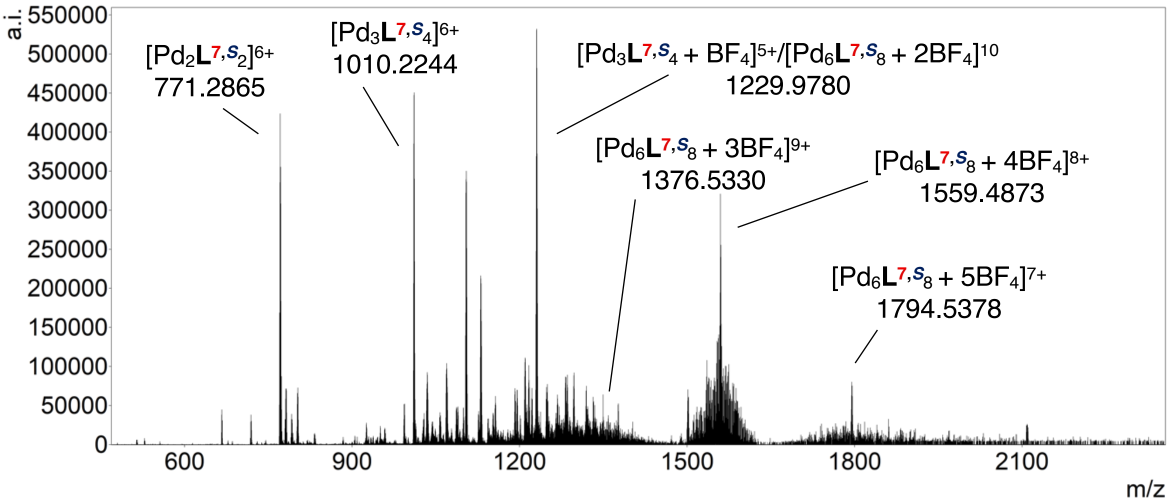

Figure S75. ESI-MS (+ve ion, H_2O/D_2O, 0.843 mM) of **[Pd$_3$L^{7,S}$_4$**(BF$_4$)$_6$]$_2$; m/z [Pd$_3$**L**^{7,S}$_4$]$^{6+}$ 1010.5648, [Pd$_3$**L**^{7,S}$_4$ BF$_4$]$^{5+}$/[Pd$_6$**L**^{7,S}$_8$ + 2BF$_4$]^{10} 1229.9780, [Pd$_6$**L**^{7,S}$_8$ + 2BF$_4$]$^{10+}$ 1376.5330, [Pd$_6$**L**^{7,S}$_8$ + 4BF$_4$]$^{8+}$ 1559.4873, [Pd$_6$**L**^{7,S}$_8$ + 5BF$_4$]$^{7+}$ 1794.5378.

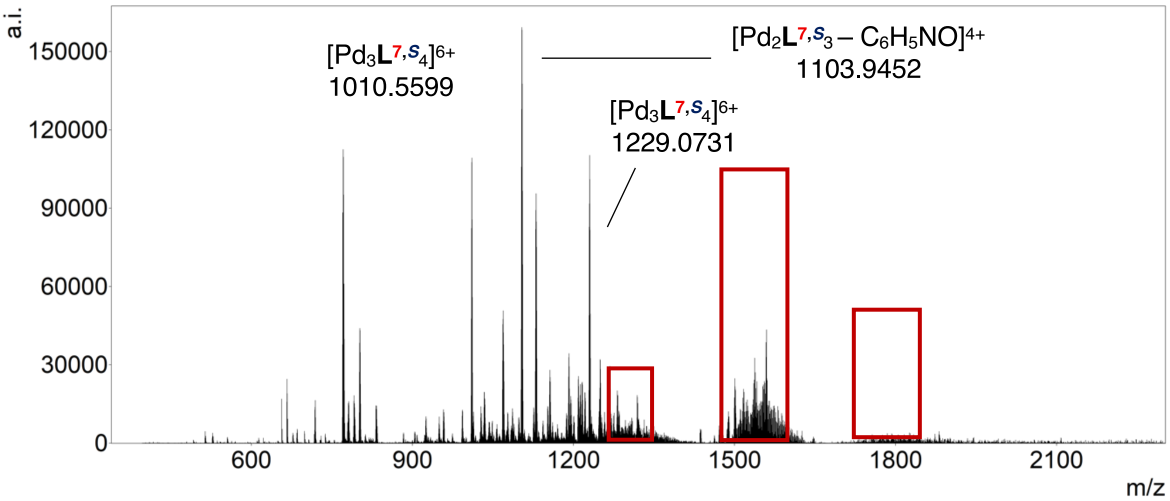

Figure S76. ESI-MS (+ve ion, H_2O/D_2O, 0.422 mM) of **[Pd$_3$L^{7,S}$_4$**(BF$_4$)$_6$]$_2$, showing disappearance/reduction of interpenetrated peaks upon dilution (highlighted)*;* m/z 1010.5599, [Pd$_3$ **L**^{7,S}$_4$]$^{6+}$, 1230.0731, [Pd$_3$**L**^{7,S}$_4$ + BF$_4$]^{5}.

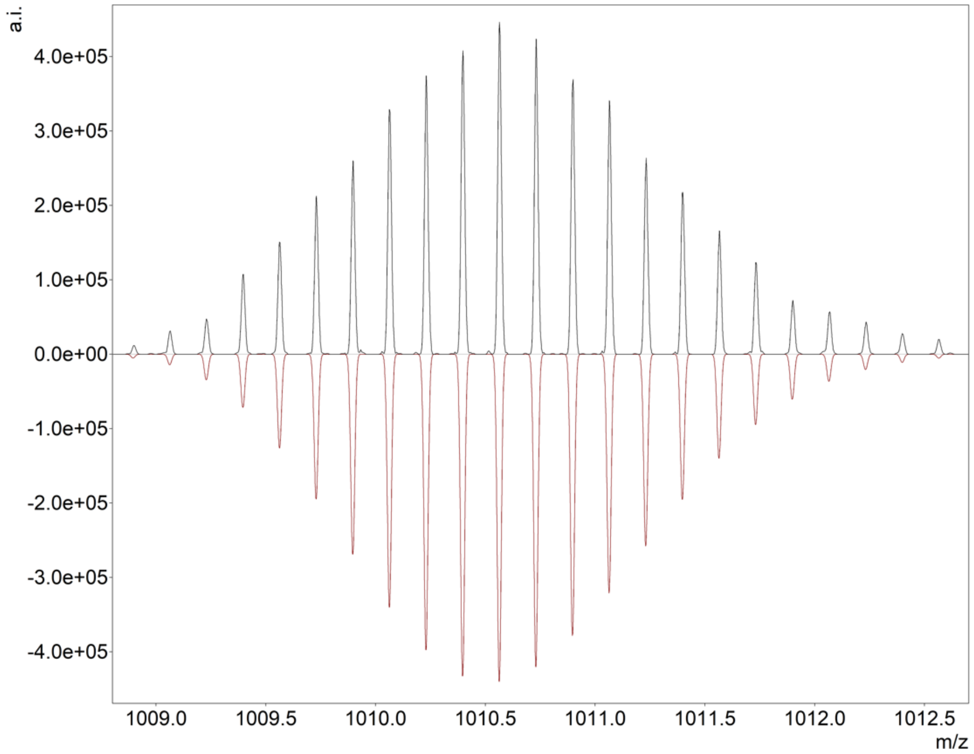

Figure S77. HRMS experimental (top) and calculated (bottom) isotope patterns of $[Pd_3\mathbf{L}^{7,S}_4]^{6+}$; m/z observed 1010.5648, calc. 1010.5632.

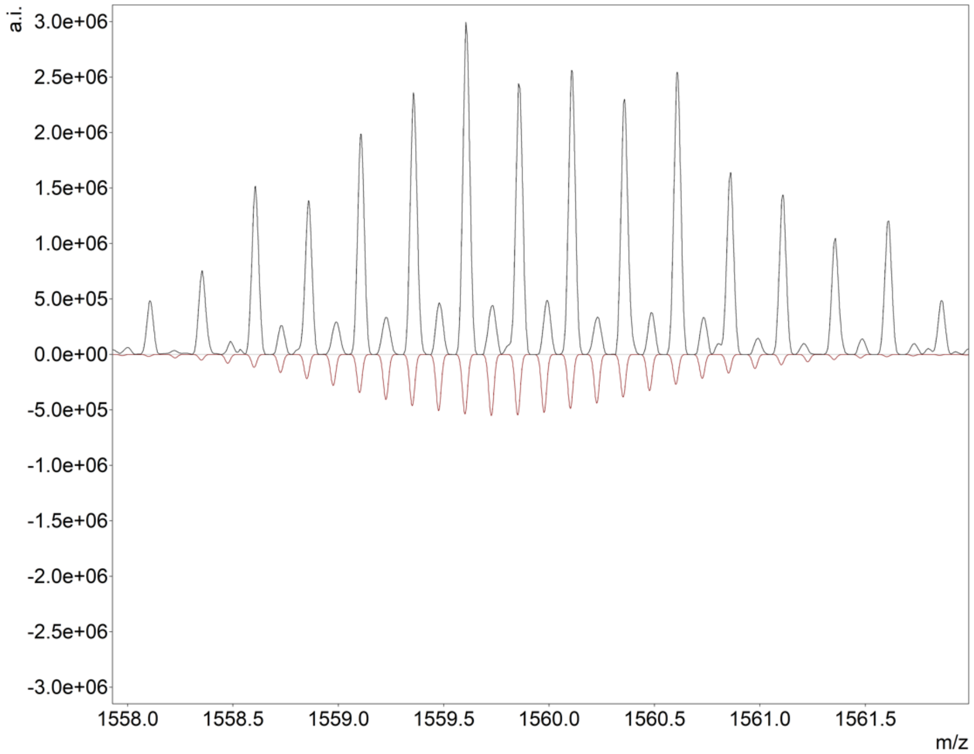

Figure S78. HRMS experimental (top) and calculated (bottom) isotope patterns of $[Pd_3\mathbf{L}^{7,S}_6 + 4BF_4]^{8+}$; m/z observed 1559.4873, calc. 1559.4758.

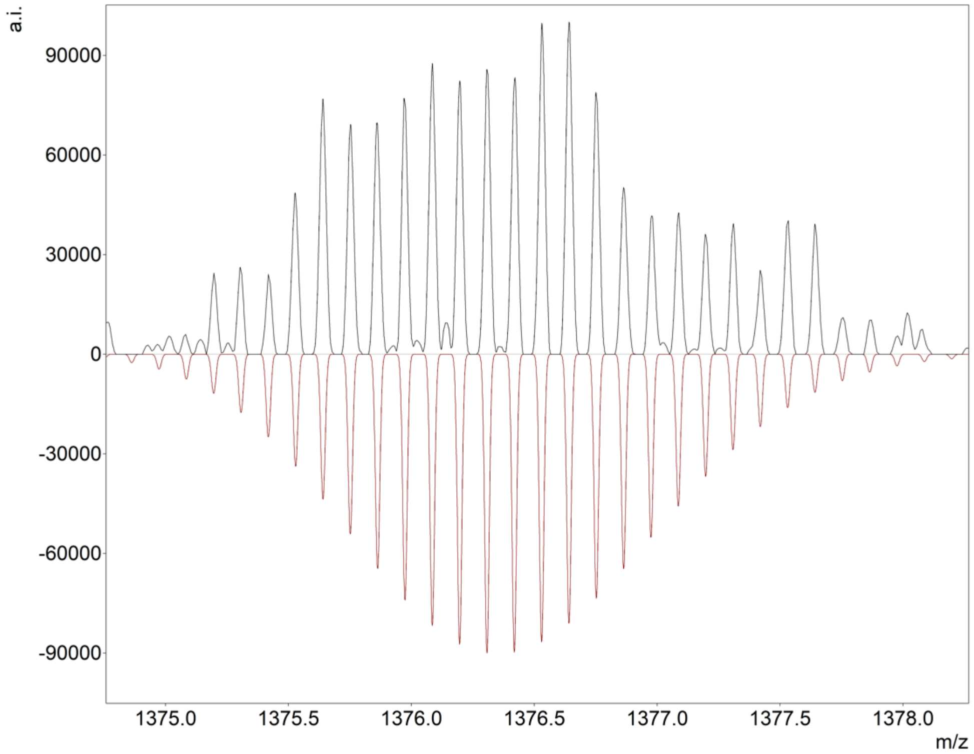

Figure S79. HRMS experimental (top) and calculated (bottom) isotope patterns of $[Pd_6\mathbf{L}^{7,S}{}_8 + 3BF_4]^{9+}$; m/z observed 1376.5330, calc. 1376.5301.

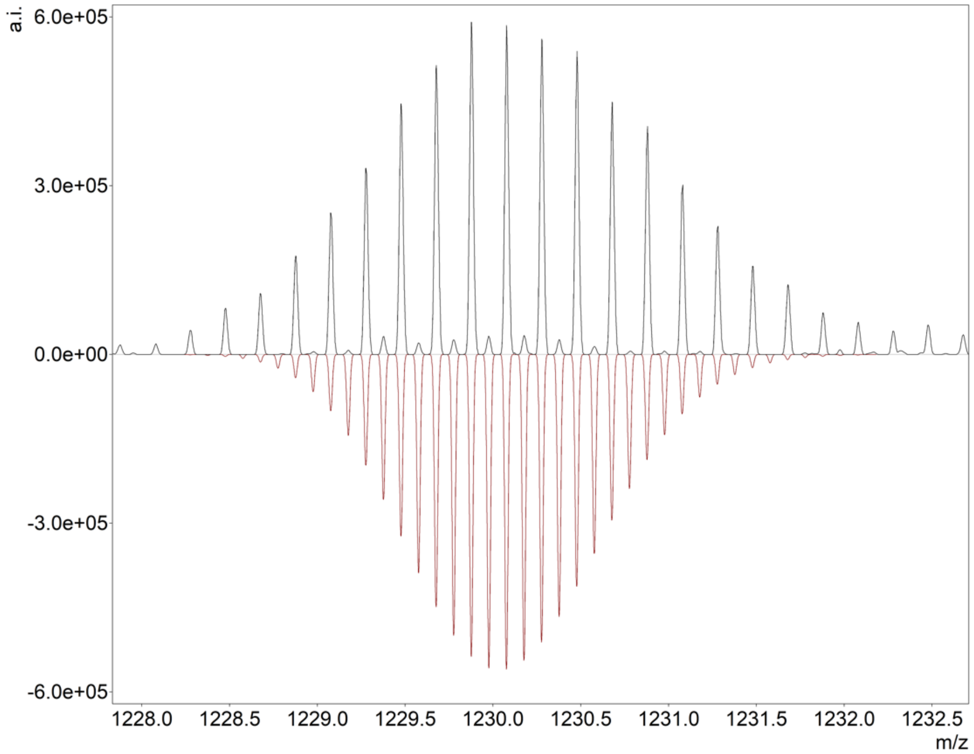

Figure S80. HRMS experimental (top) and calculated (bottom) isotope patterns of $[Pd_3\mathbf{L}^{7,S}{}_4 + BF_4]^{5+}$/minor $[Pd_6\mathbf{L}^{7,S}{}_8 + 2BF_4]^{10+}$; m/z observed 1229.9780, calc. 1229.9765.

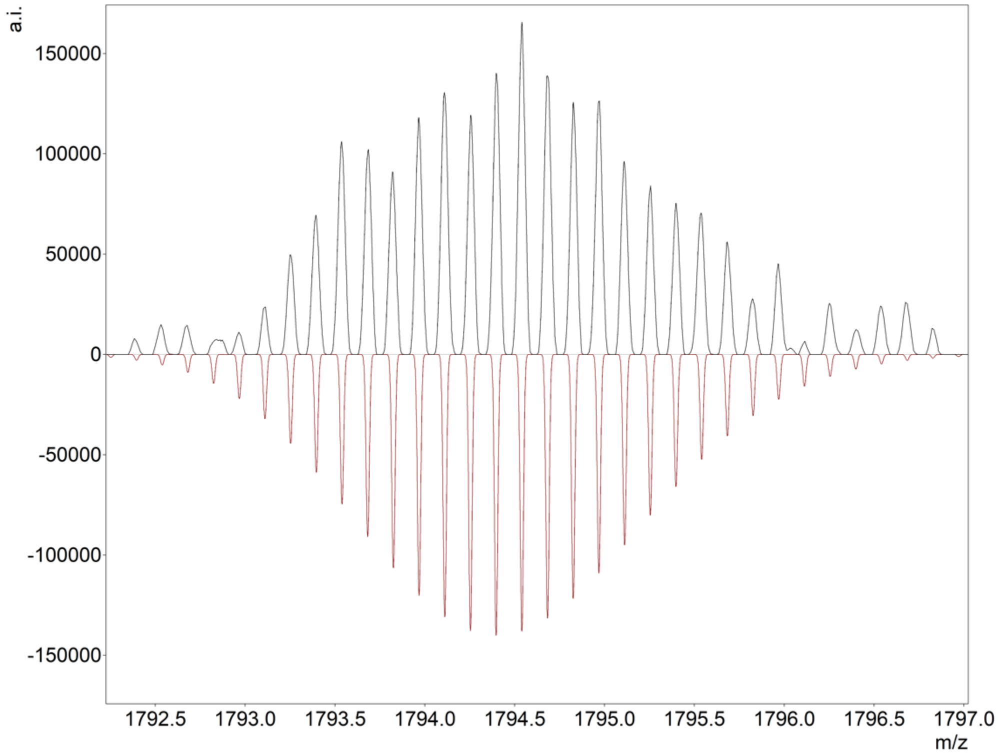

Figure S81. HRMS experimental (top) and calculated (bottom) isotope patterns of [Pd_6**L**$^{7,S}{}_8$ + $5BF_4$]$^{7+}$; m/z observed 1794.5378, calc. 1794.5400.

Metal-Peptidic Cage ***Pd*****$_3$*****L*****^{4,S}*$_4$(BF$_4$)$_6$***

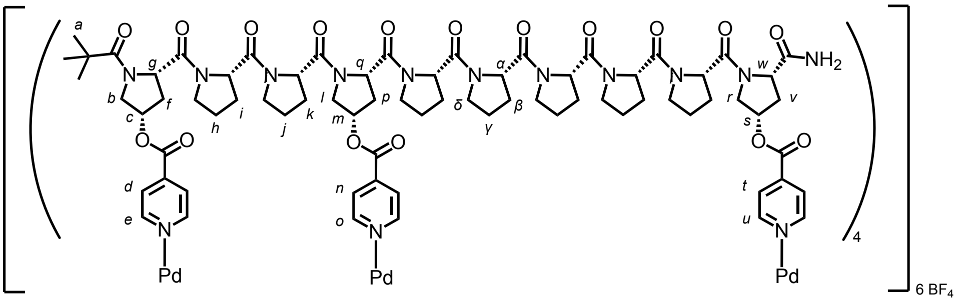

Pd$_3$L^{4,S}$_4$(BF$_4$)$_6$ was prepared by submitting **L^{4,S}** to the **General Cage Procedure**. This yielded 500 µL of **Pd$_3$L^{4,S}$_4$**(BF$_4$)$_6$ as a pale-yellow solution, at 0.937 mM, which was used without further purification.

The ^{1}H NMR indicates formation of minor alternate cage isomers, whose signals overlap with the major cage product. Due to the complexity of the spectra, and multiple overlaps, precluding their assignment, these signals have not been reported below.

^{1}H NMR (950 MHz, D$_2$O) δ 9.30 (d, *J* = 6.0 Hz, 8H, H$_o$), 9.15 (d, *J* = 6.0 Hz, 8H, H$_e$), 8.98 (d, *J* = 6.0 Hz, 8H, H$_u$), 8.16-8.13 (app. t, *J* = 6.0 Hz, 16H, H$_d$ and H$_t$), 7.96 (d, *J* = 6.0 Hz, 8H, H$_n$), 5.81 (br s, 4H, H$_s$), 5.69 (br s, 4H, H$_m$), 5.46 (br s, 4H, H$_c$), 4.93 - 4.90 (m, 4H, H$_g$), 4.87 – 4.86 (m, 4H, H$_w$), 4.71 – 4.59 (m, 8H, 8 of H$_k$), 4.38 - 4.36 (app. d, *J* = 11.6 Hz, 4H, H$_b$), 4.11 – 4.08 (m, 4H, H$_r$), 4.04 – 4.01 (m, 4H, H$_{b'}$), 3.99 – 3.90 (m, 16H, H$_l$ and 12 of H$_δ$), 3.88 – 3.78 (m, 12H, 12 of H$_δ$), 3.76 – 3.72 (m, 4H, 4 of H$_δ$), 3.72 – 3.64 (m, 8H, H$_{l'}$ and 4 of H$_δ$), 3.65 – 3.57 (m, 8H, H$_{r'}$ and 4 of H$_δ$), 3.56 – 3.46 (m, 4H, 4 of H$_δ$), 3.26 – 3.22 (m, 4H, 4 of H$_δ$), 2.97 – 2.92 (m, 8H, H$_v$), 2.86 – 2.80 (m, 4H, 4 of H$_α$*), 2.71 – 2.51 (m, 26H, H$_f$, H$_p$ and 10 of H$_β$), (m, 34H, 34 of H$_β$ and H$_γ$), 1.91 – 1.80 (m, 8H, 8 of H$_β$ and H$_γ$), 1.68 – 1.59 (m, 8H, 8 of H$_β$ and H$_γ$), 1.51 – 1.47 (4H, m, H$_h$ or H$_j$*), 1.40 – 1.36 (4H, m, H$_{i'}$ or H$_{k'}$), 1.32 (36H, s, H$_a$) 0.55 – 0.52 (m, 4H, H$_{h'}$ or H$_{j'}$*), 0.47 – 0.43 (m, 4H, 4 of H$_{i'}$ or H$_{k'}$).* ^{13}C NMR (151 MHz, D$_2$O) δ 179.8, 175.3, 175.0, 172.7, 171.6, 171.3, 170.2, 164.0, 163.8, 152.3, 152.3, 152.2, 152.0, 151.9, 151.9, 151.8, 142.4, 140.8, 126.8, 126.7, 126.7, 126.6, 126.6, 126.5, 119.1, 78.0, 76.7, 74.9, 59.6, 59.3, 58.8, 58.6, 57.2, 54.0, 52.8, 47.7, 47.4, 46.7, 46.1, 28.3, 28.2, 28.2, 28.1, 28.0, 28.0, 28.0, 27.7, 27.6, 27.6, 27.2, 26.4, 26.3, 24.9, 24.7, 24.3. ESI-MS: m/z [Pd$_3$**L^{4,S}**$_4$ + BF$_4$]$^{5+}$, 1229.8655, [Pd$_3$**L^{4,S}**$_4$ + 2BF$_4$]$^{4+}$, 1559.0851, [Pd$_3$**L^{4,S}**$_4$ + 3BF$_4$]$^{3+}$, 2107.7822. HRMS: m/z observed 1229.8665, calc. 1229.8752 (Figure S90).

*These protons are shifted significantly upfield due to ring currents from perpendicular pyridine groups in close proximity. NOEs from the relevant pyridines can be found to both H$_d$/H$_e$ and H$_n$/H$_o$, meaning whilst the location of these protons can be narrowed down to one of the two proline residues between the smaller segment of our ligand (i.e. Hyp-Pro-Pro-Hyp) in between these pyridines, it is not possible to assign with absolute certainty.

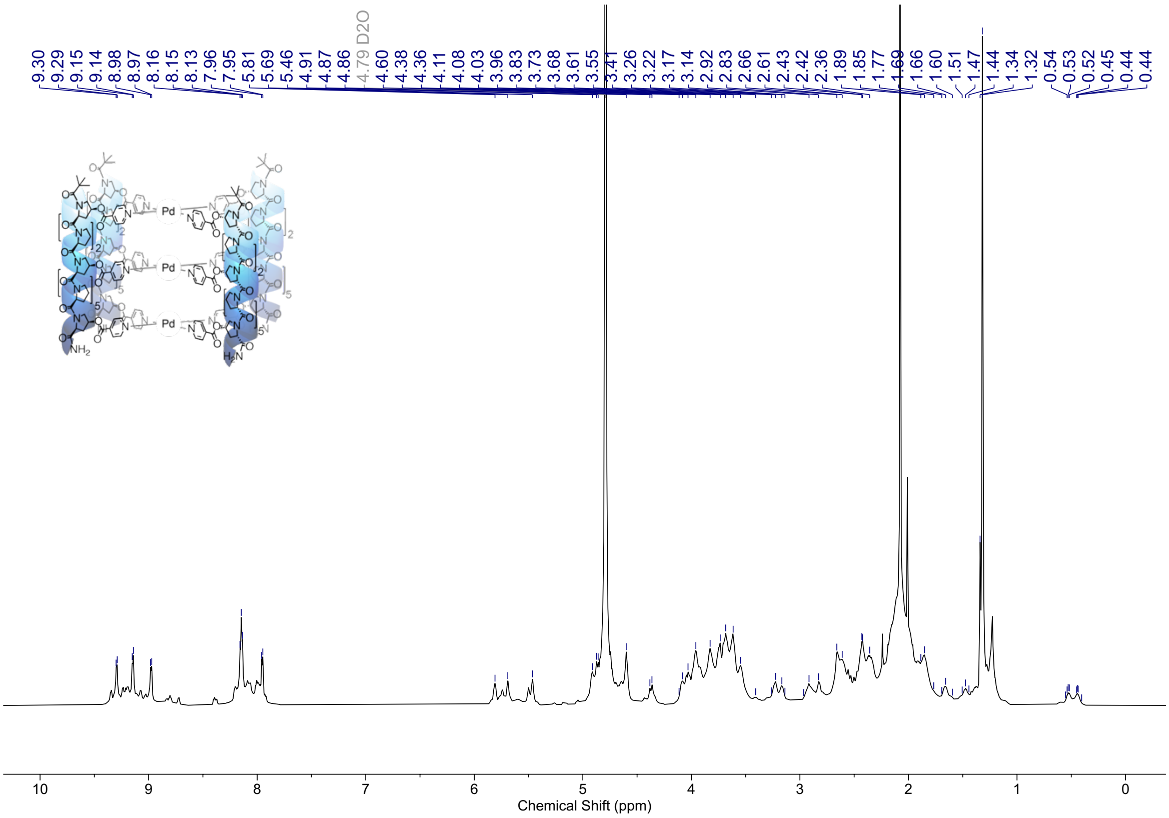

Figure S82. ^{1}H NMR (D_2O, 600 MHz, 298 K) of **$Pd_3L^{4,S}_4(BF_4)_6$**.

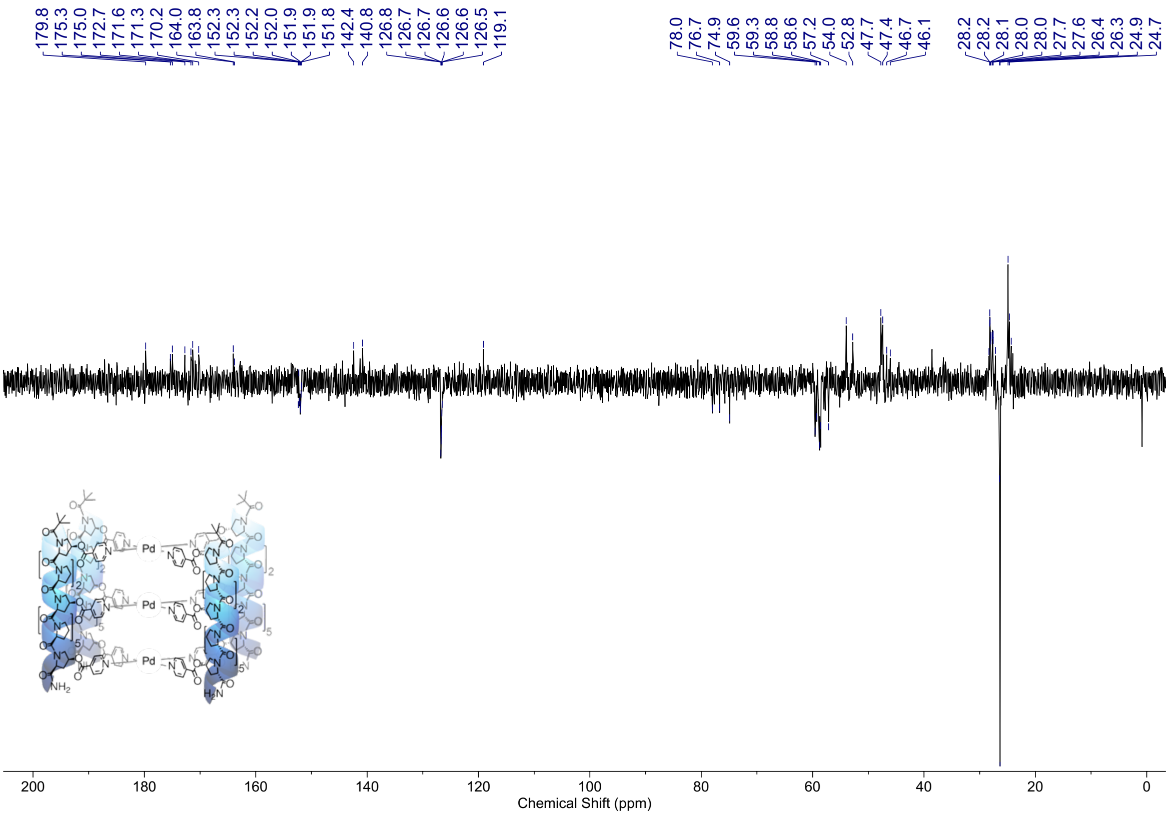

Figure S83. ^{13}C JMOD (D_2O, 151 MHz, 298 K) of **$Pd_3L^{4,S}_4(BF_4)_6$**.

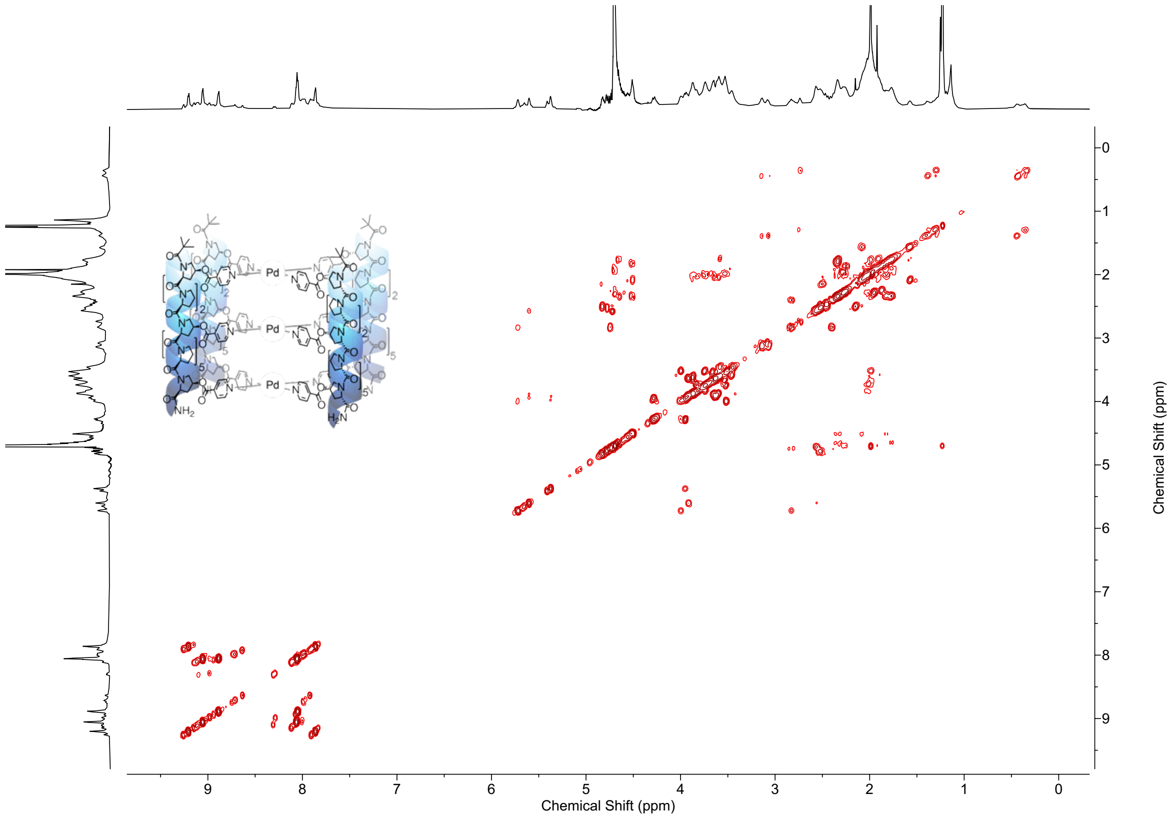

Figure S84. ^{1}H-^{1}H COSY (D_2O, 600 MHz, 298 K) of $\mathbf{Pd_3L^{4,\mathit{S}}_4}(BF_4)_6$.

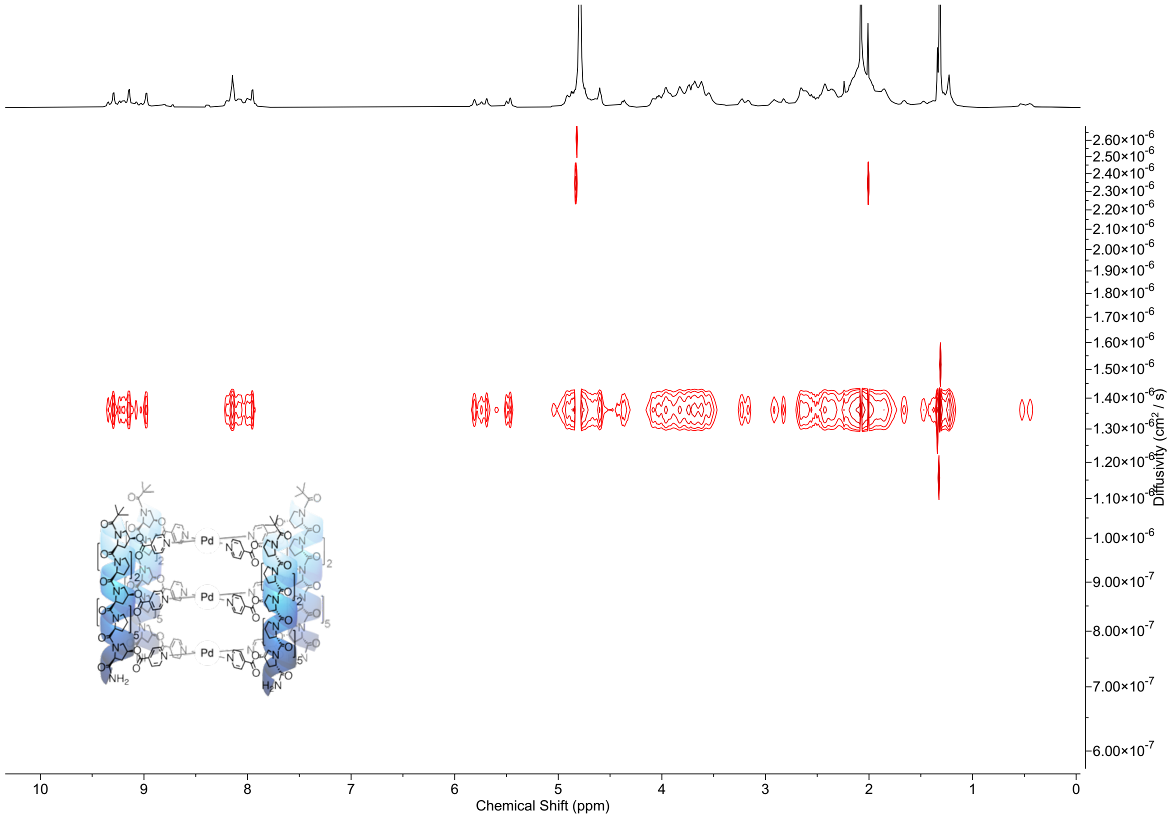

Figure S85. ^{1}H DOSY (D_2O, 600 MHz, 298 K) of $\mathbf{Pd_3L^{4,\mathit{S}}_4}(BF_4)_6$. The diffusion coefficient of $\mathbf{Pd_3L^{4,\mathit{S}}_4}(BF_4)_6$ was 1.361 x 10^{-6} cm^2 s^{-1}, giving a hydrodynamic radius of 24.0 Å.

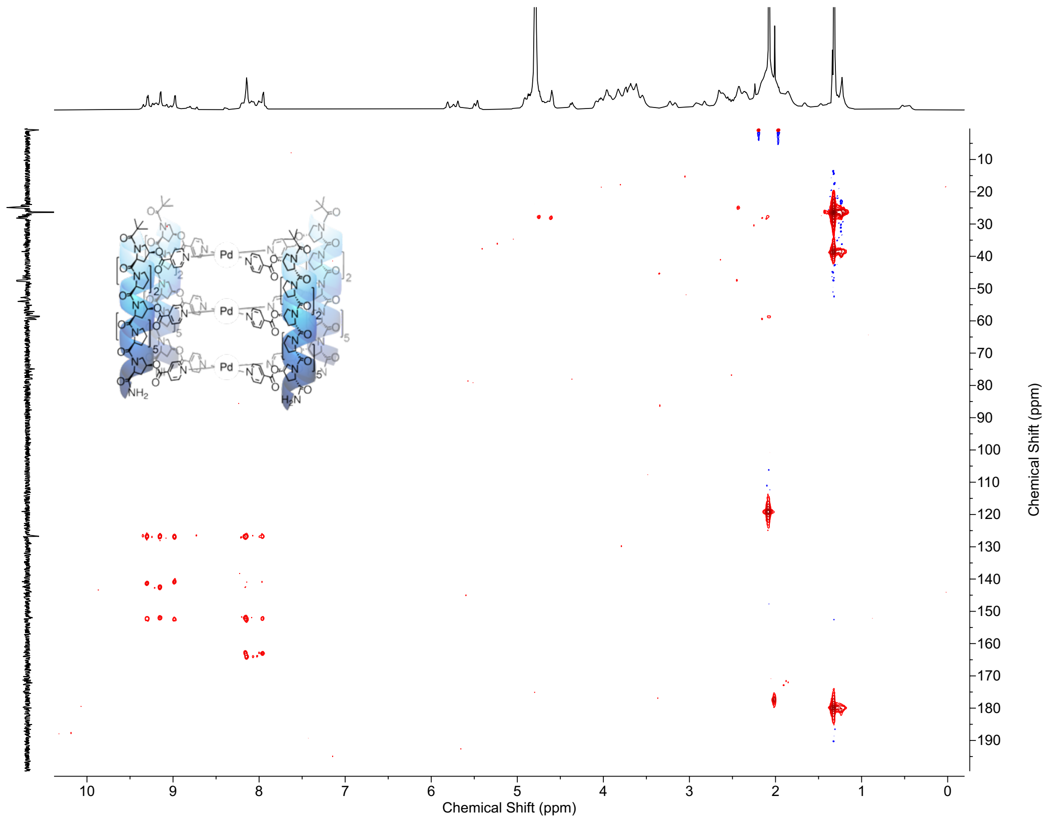

Figure S86. ^{1}H-^{13}C HMBC (D_2O, 600 MHz, 298 K) of $\mathbf{Pd_3L^{4,S}_4}(BF_4)_6$.

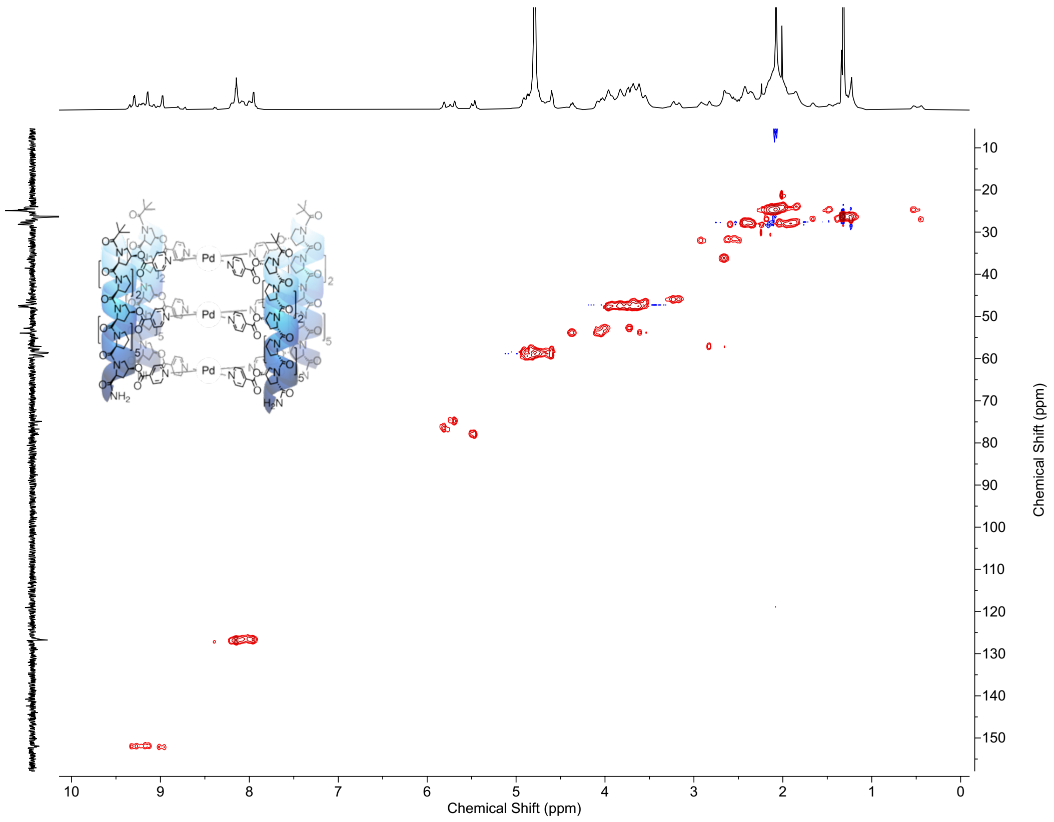

Figure S87. ^{1}H-^{13}C HSQC (D_2O, 600 MHz, 298 K) of $\mathbf{Pd_3L^{4,S}_4}(BF_4)_6$.

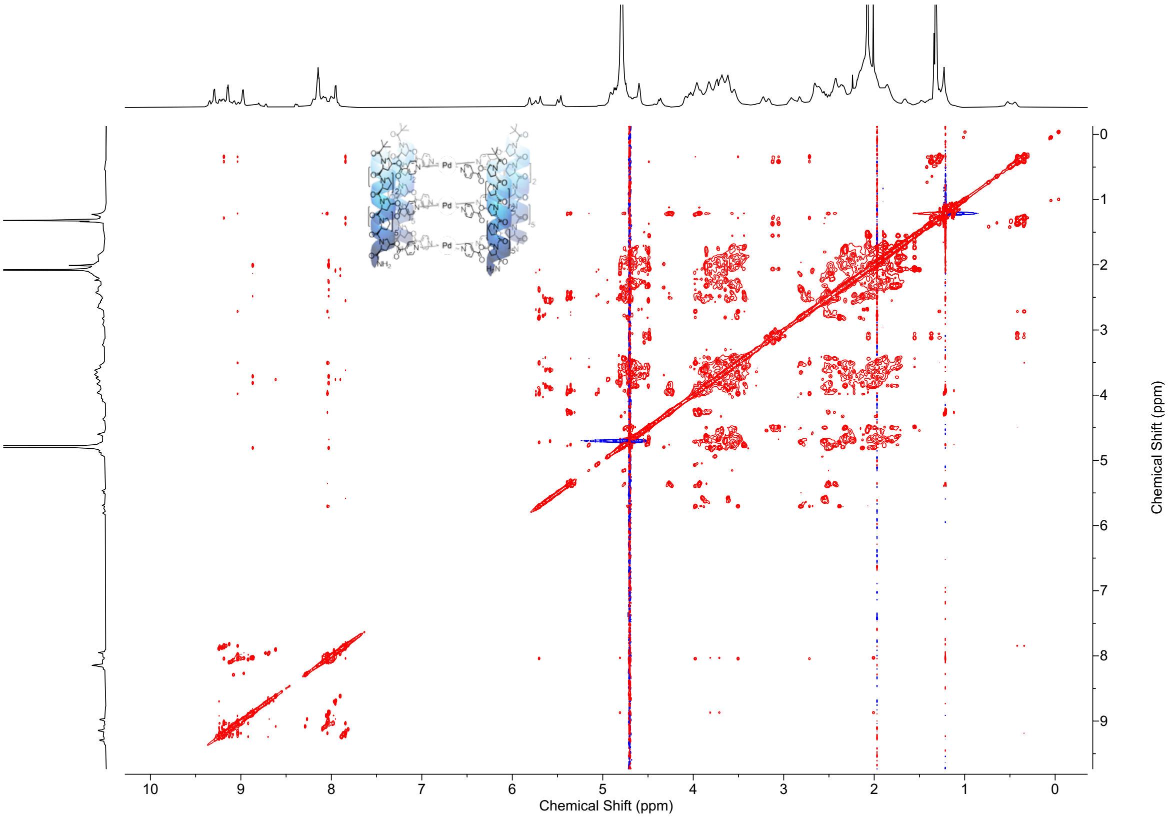

Figure S88. ^{1}H-^{1}H NOESY (D_2O, 600 MHz, 298 K, Mixing time = 0.300 s) of $\mathbf{Pd_3L^{4,S}_4(BF_4)_6}$.

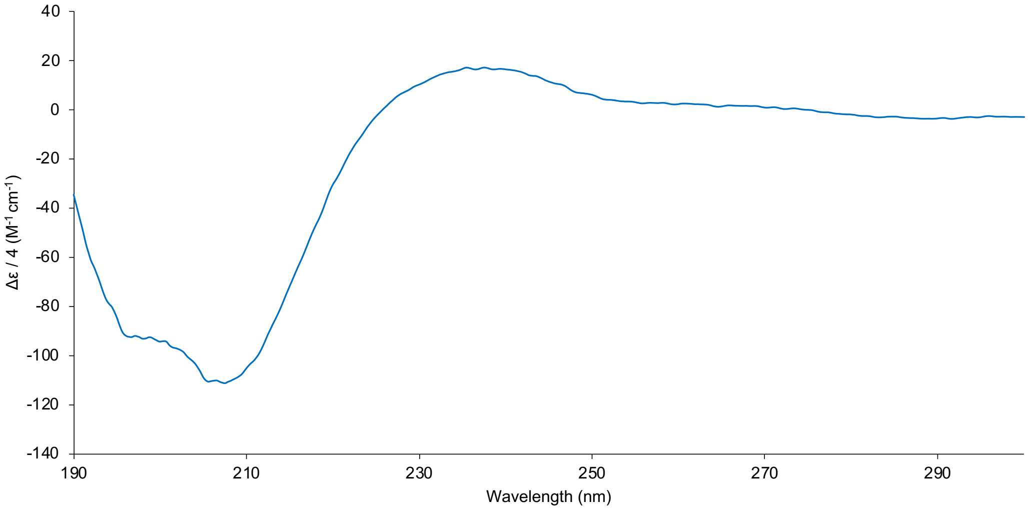

Figure S89. CD spectrum of $\mathbf{Pd_3L^{4,S}_4(BF_4)_6}$ in H_2O (293 K, 0.1867 mM, 0.1 mm), standardised to ligand concentration. Minima and maxima observed at 207 nm and 238 nm.

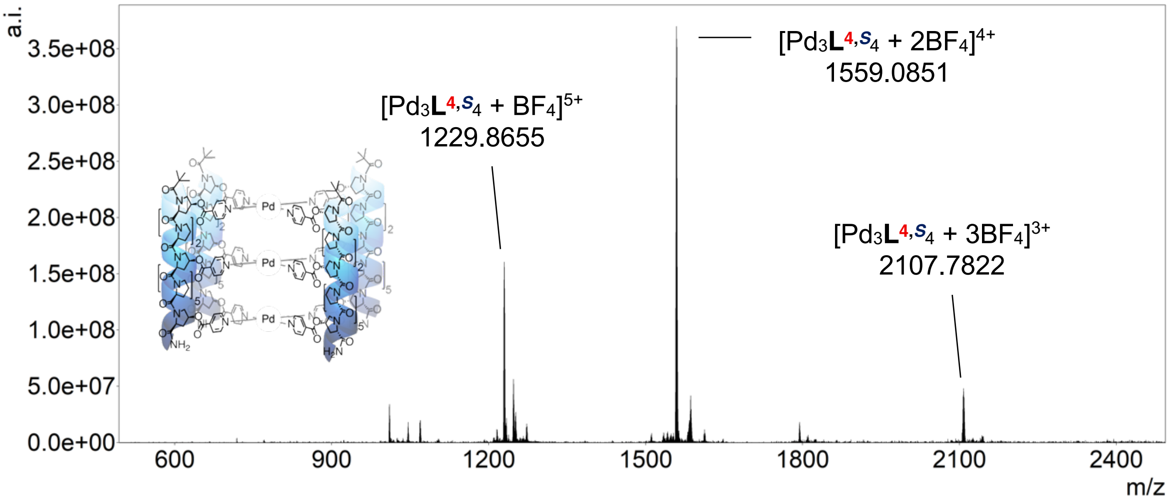

Figure S90. ESI-MS (+ve ion, H_2O/D_2O) **$Pd_3L^{4,S}{}_4$**$(BF_4)_6$**;** m/z $[Pd_3\mathbf{L}^{4,S}{}_4 + BF_4]^{5+}$, 1229.8655, $[Pd_3\mathbf{L}^{4,S}{}_4 + 2BF_4]^{4+}$, 1559.0851, $[Pd_3\mathbf{L}^{4,S}{}_4 + 3BF_4]^{3+}$, 2107.7822.

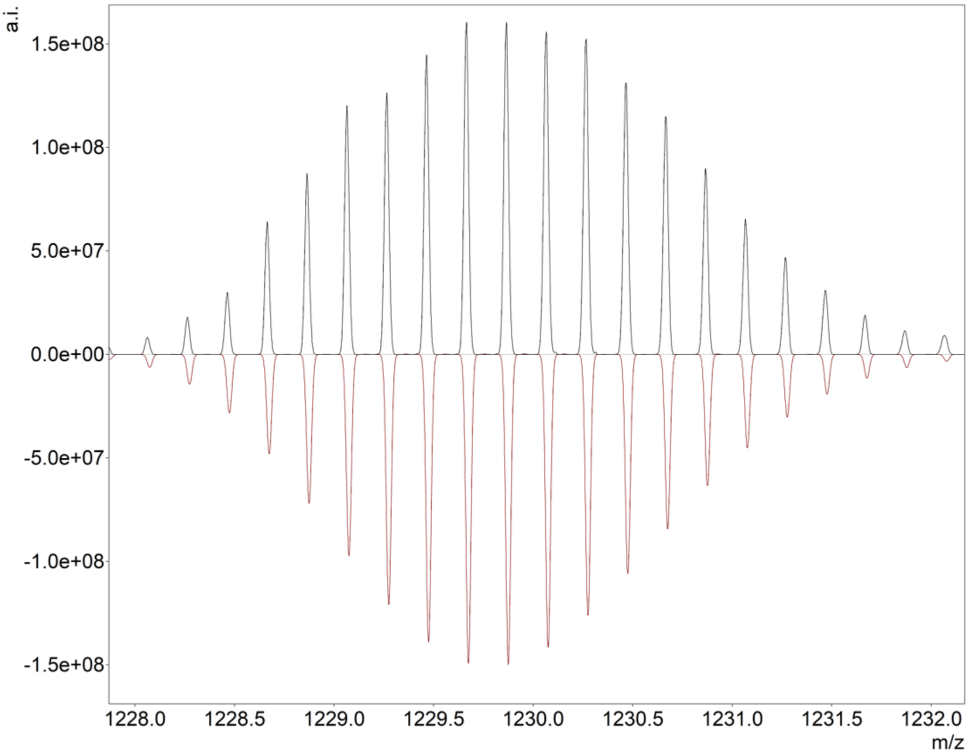

Figure S91. HRMS experimental (top) and calculated (bottom) isotope patterns of $[Pd_3\mathbf{L}^{4,S}{}_4 + BF_4]^{5+}$; m/z observed 1229.8665, calc. 1229.8752.

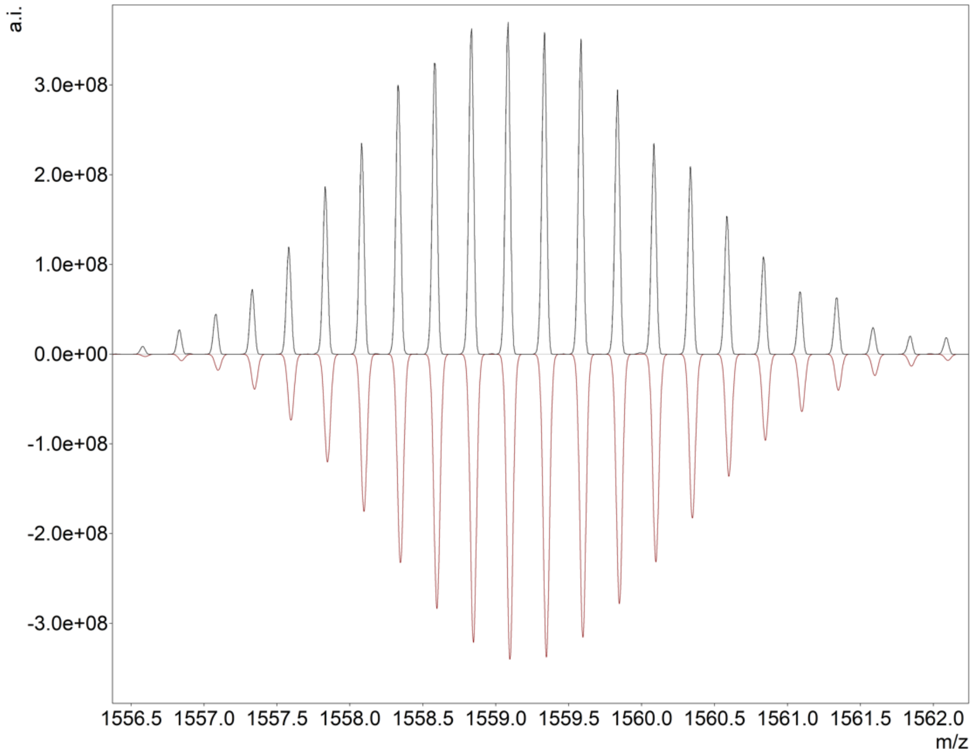

Figure S92. HRMS experimental (top) and calculated (bottom) isotope patterns of $[Pd_3\mathbf{L}^{4,S}_4 + 2BF_4]^{4+}$; m/z observed 1559.0851, calc. 1559.0971.

5. Cage Titrations

Increasing quantities of $Pd(CH_3CN)_4(BF_4)_2$ (0 – 0.75 eq) were titrated against ligands **L**^{7,R} (Figures S93 + S94) and **L**^{7,S} (Figures S95 – S96), and the species formed at sub-stoichiometric quantities of $Pd(CH_3CN)_4(BF4)_2$ monitored by ^1H NMR and ESI-HRMS.

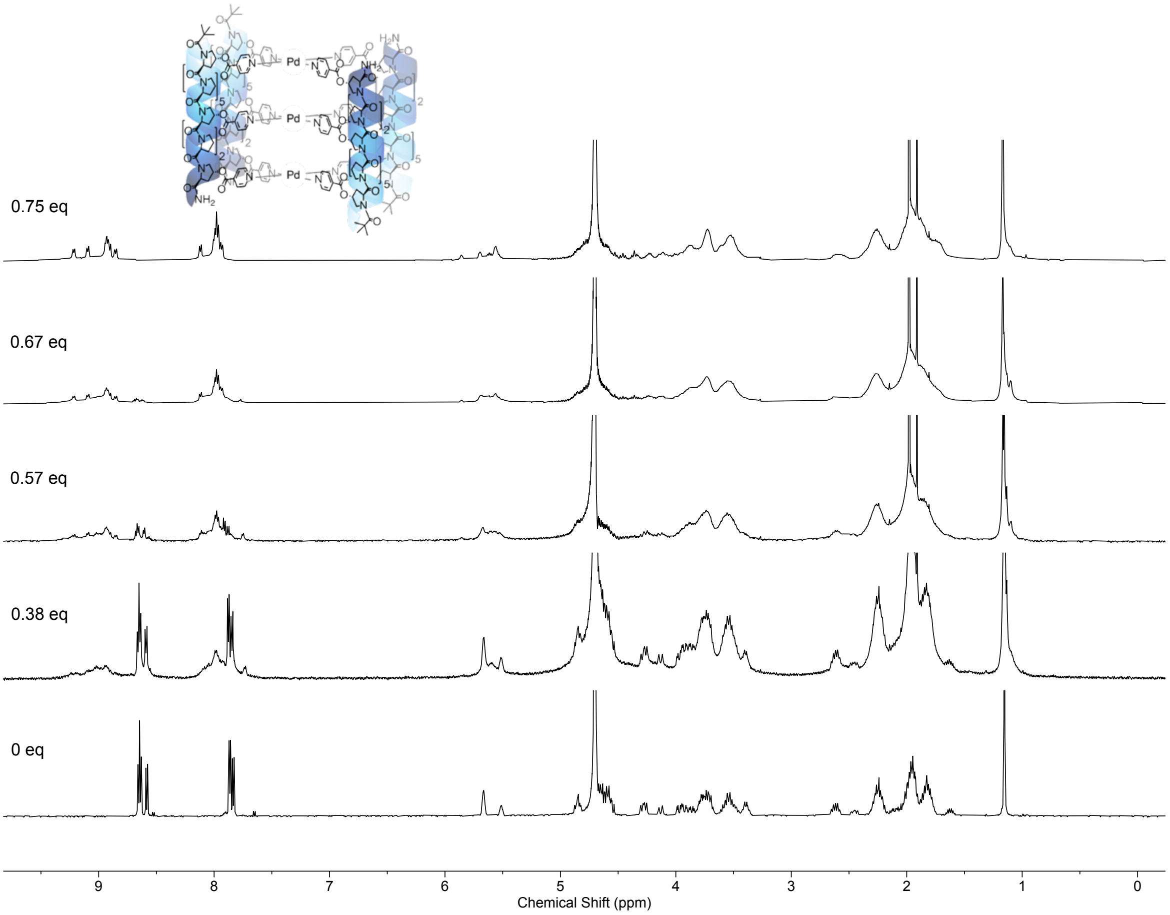

Figure S93. ^1H NMR titration (D_2O, 600 MHz, 298 K) of $Pd(CH_3CN)_4(BF_4)_2$ against ligand **L**^{7,R}.

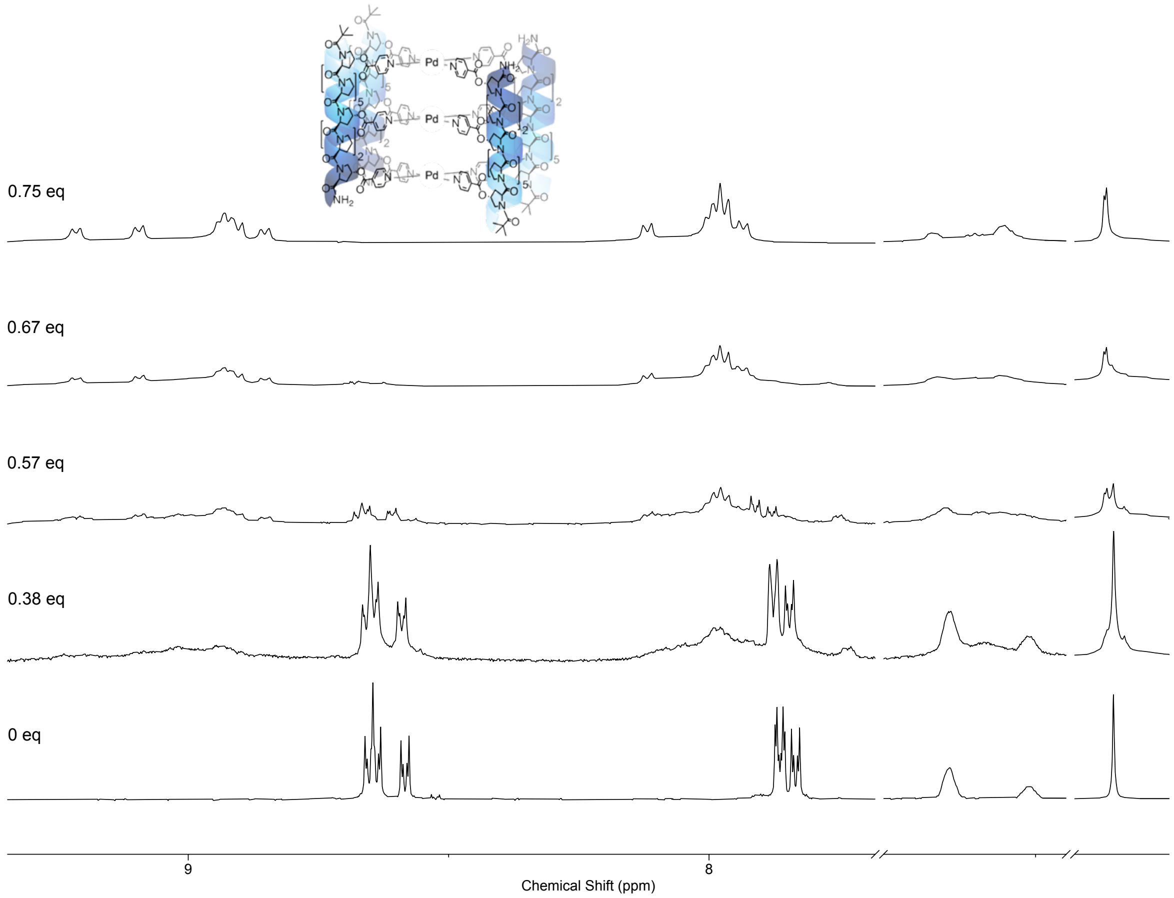

Figure S94. Partial ^{1}H NMR titration (D_2O, 600 MHz, 298 K) of $Pd(CH_3CN)_4(BF_4)_2$ against ligand **L**^{7,R}. Signals at ~1 ppm have been scaled independently to the rest of the spectrum due to relative intensity to show splitting of ^{t}Bu peak into two environments.

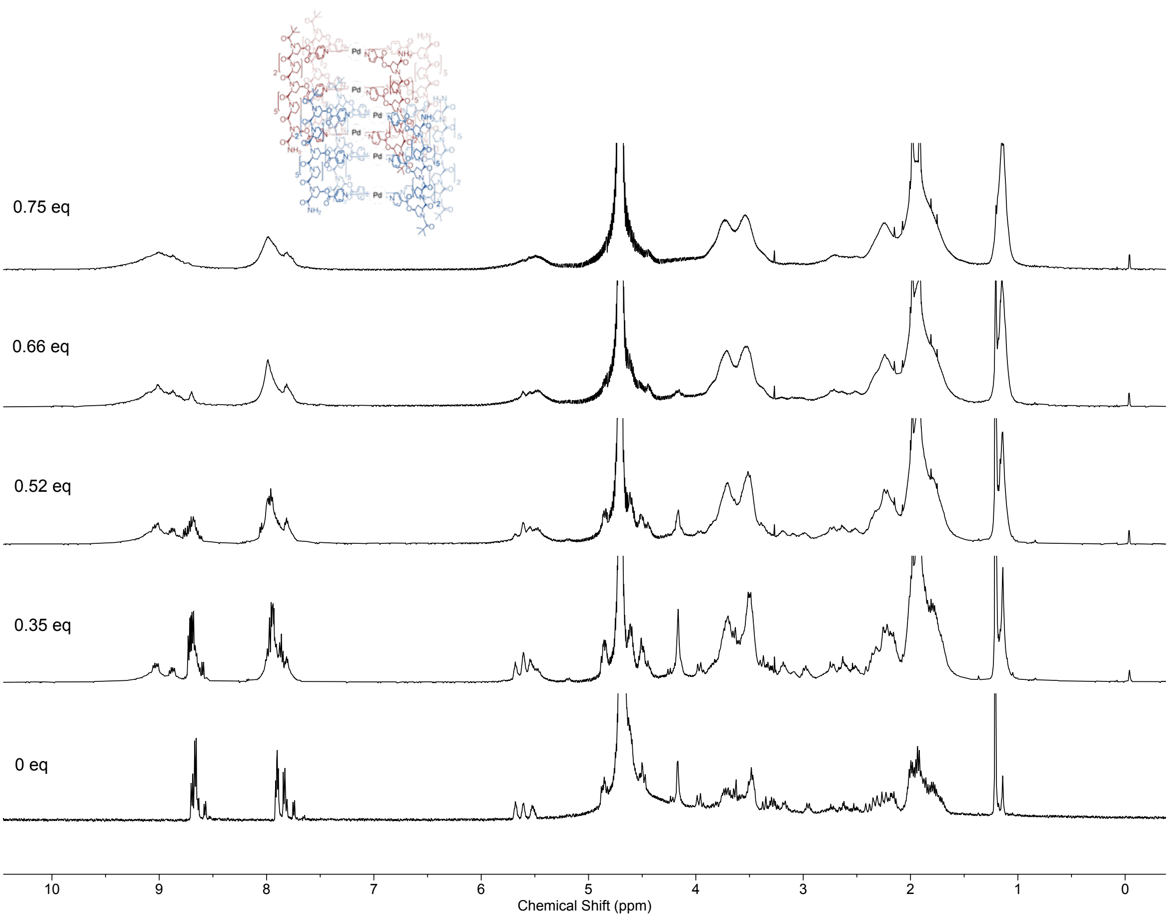

Figure S95. ^{1}H NMR titration (D_2O, 600 MHz, 298 K) of $Pd(CH_3CN)_4(BF_4)_2$ against ligand $\mathbf{L}^{7,S}$.

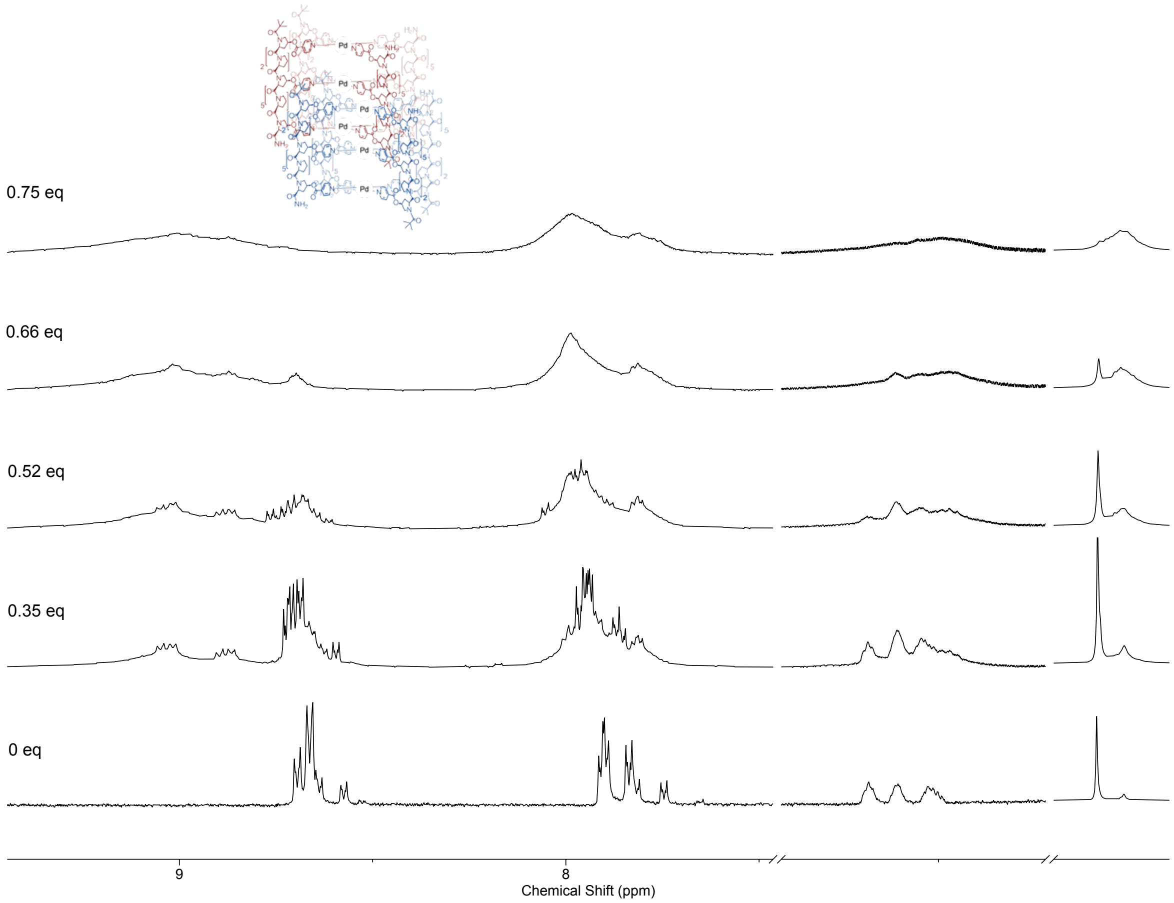

Figure S96. Partial ^{1}H NMR titration (D_2O, 600 MHz, 298 K) of $Pd(CH_3CN)_4(BF_4)_2$ against ligand **L**^{7,S}. Signals at ~1 ppm have been scaled independently to the rest of the spectrum due to relative intensity.

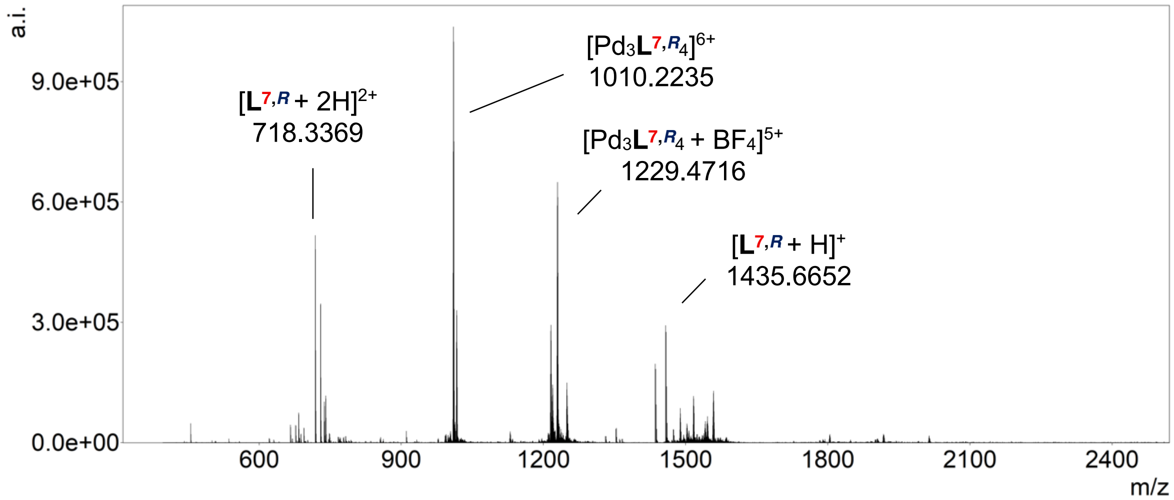

Figure S97. ESI-MS (+ve ion, H_2O/D_2O) of **L**^{7,R} with 0.6 eq. $Pd(CH_3CN)_4(BF_4)_2$; m/z [Pd$_3$**L**^{7,R}$_4$ + BF$_4$]$^{5+}$.

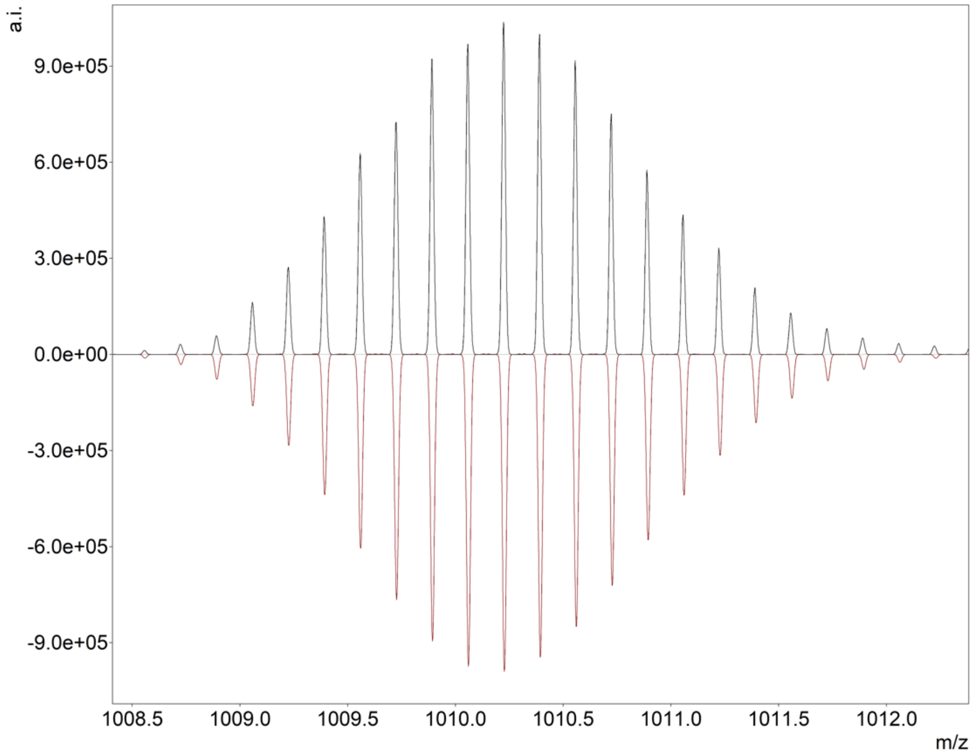

Figure S98. HRMS experimental (top) and calculated (bottom) isotope patterns of $[Pd_3\mathbf{L}^{7,R}{}_4 + BF_4]^{6+}$; m/z observed 1010.2235, calc. 1010.2272.

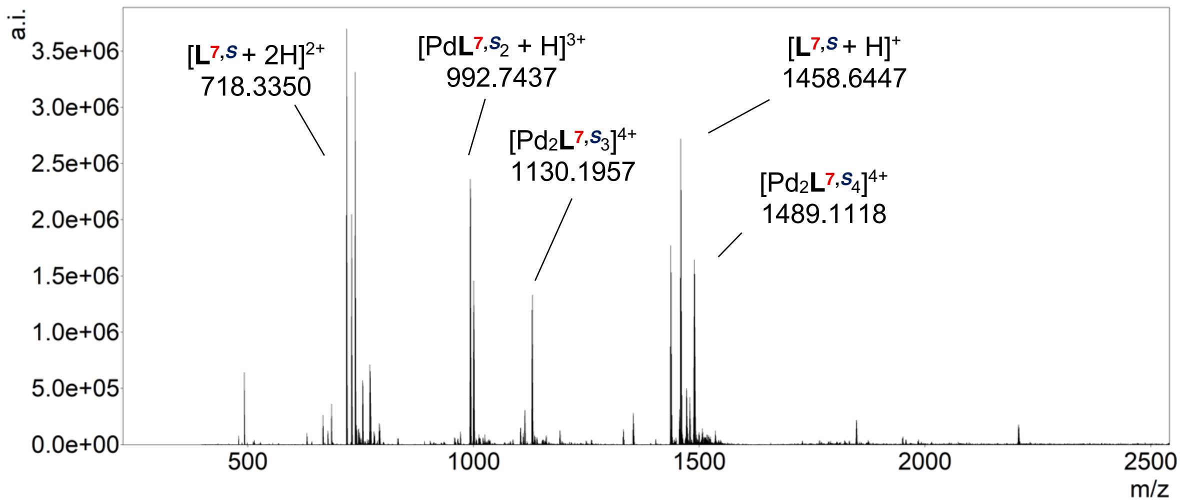

Figure S99. ESI-MS (+ve ion, H_2O/D_2O) of $\mathbf{L}^{7,S}$ with 0.6 eq. $Pd(CH_3CN)_4(BF_4)_2$.

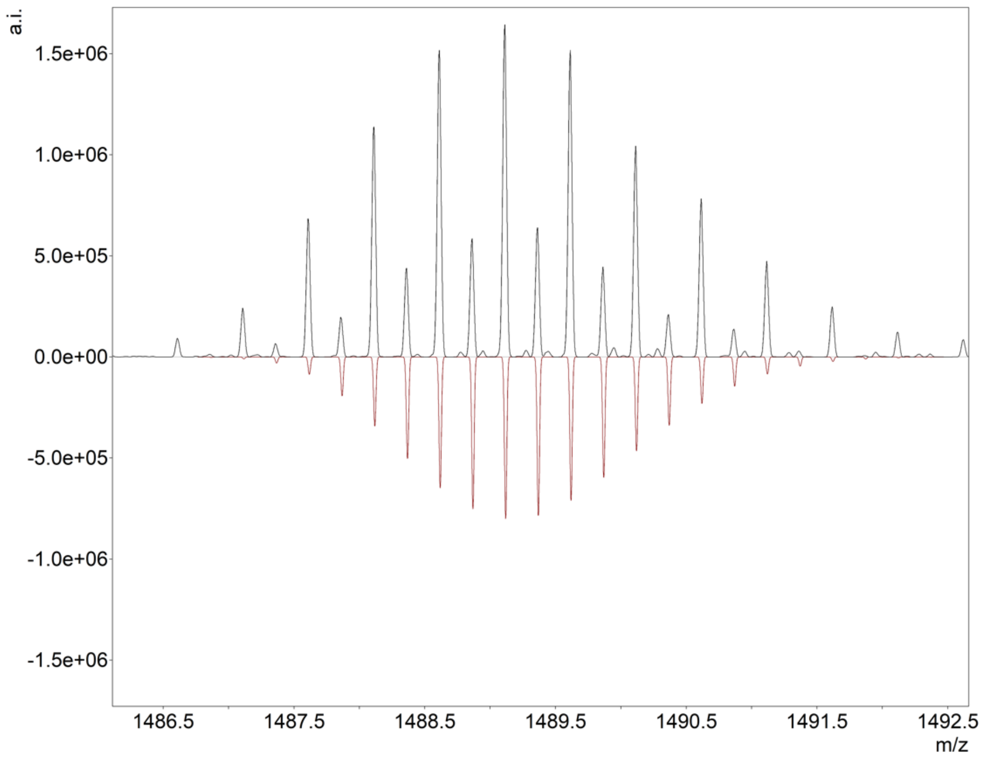

Figure S100. HRMS experimental (top) and calculated (bottom) isotope patterns of $[Pd_2\mathbf{L}^{7,S}{}_4]^{4+}$; m/z observed 1489.1118, calc. 1489.1154. Peak coincides with $[Pd\mathbf{L}^{7,S}{}_2]^{2+}$ fragment peak.

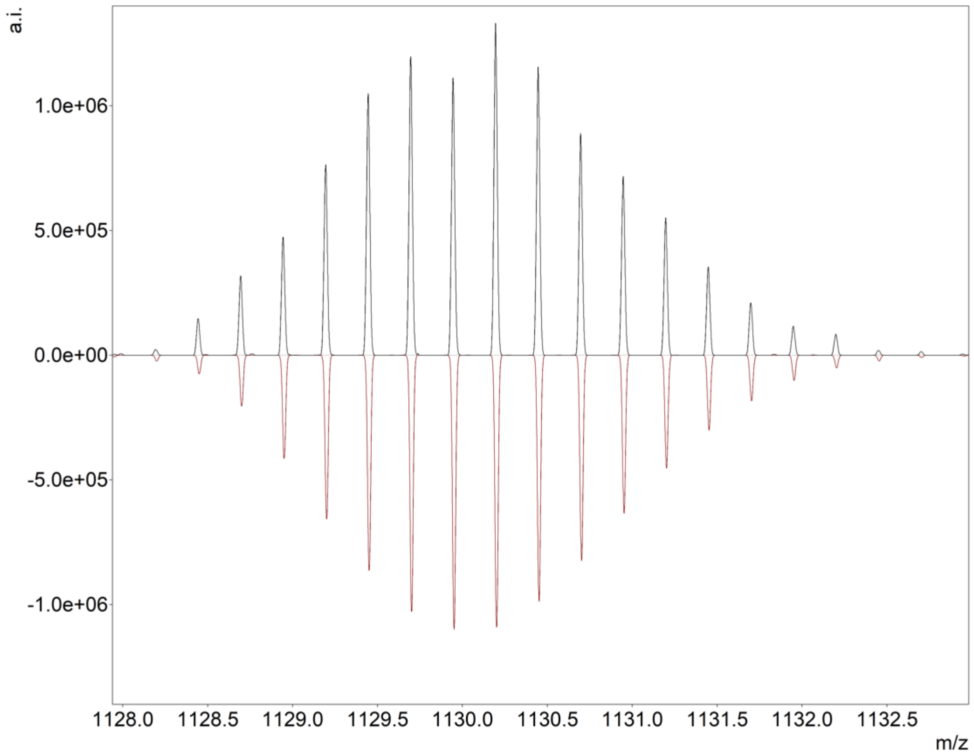

Figure S101. HRMS experimental (top) and calculated (bottom) isotope patterns of $[Pd_2\mathbf{L}^{7,S}_3]^{4+}$; m/z observed 1130.1957, calc. 1130.2012.

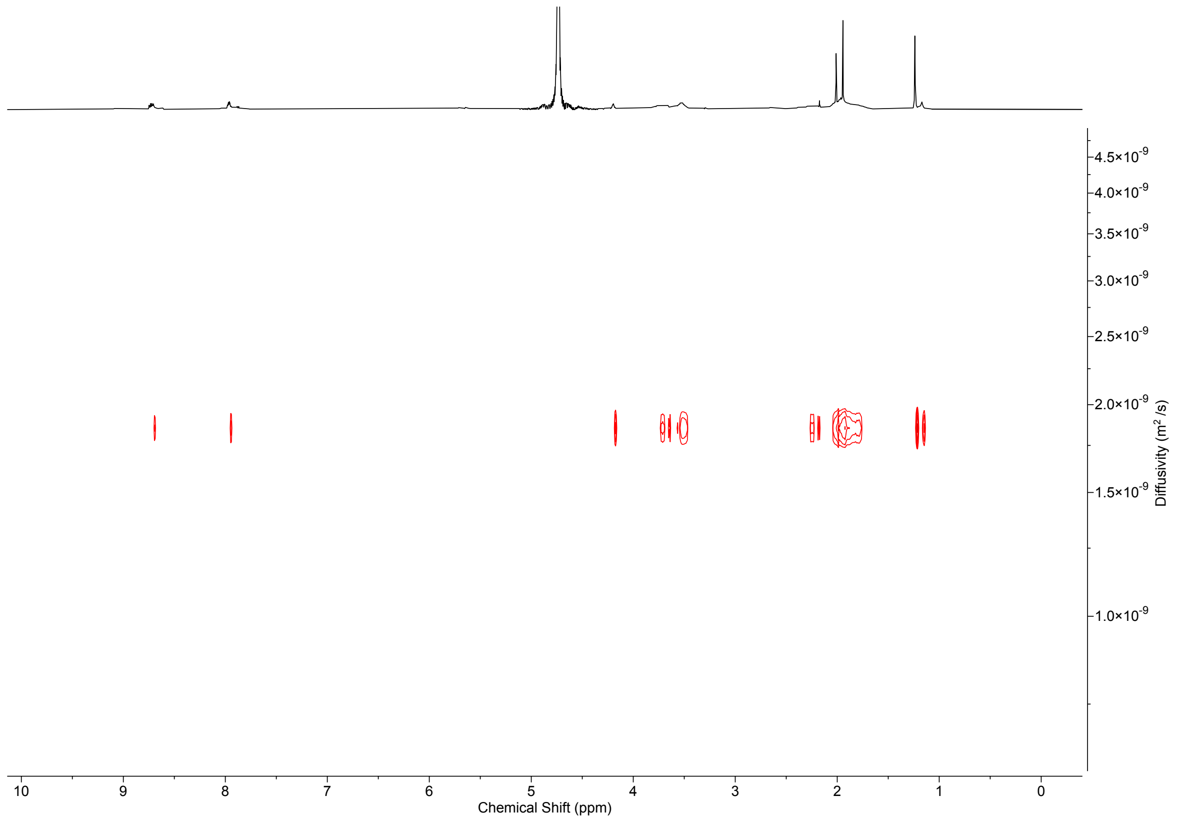

Figure S102. ^{1}H DOSY (D_2O, 600 MHz, 298 K) of intermediate $Pd_2\mathbf{L}^{7,S}_4$**.** The diffusion coefficient of the obtained species was 1.843 x 10^{-9} m^2 s^{-1}, giving a hydrodynamic radius of 17.7 Å.

6. DOSY Analysis

To confirm calculated diffusion coefficients using processing in Mestrenova, DOSY spectra were additionally processed in Bruker TopSpin and Dynamics Centre using the parameters outlined in Table S2.

Fitted function:	f (x) = Io * exp (-D * x^2 * gamma^2 * littleDelta^2 (bigDelta-littleDelta/3)* 10^4
Used gamma:	26752 rad/(s*Gauss)
Used little delta:	0.0020000 s
Used big delta:	0.099900 s
Used gradient strength:	variable
Random error estimation of data:	RMS per spectrum (or trace/plane)
Systematic error estimation of data:	worst case per peak scenario
Fit parameter Error estimation method:	from fit using arbitrary y uncertainties
Confidence level:	95%
Used peaks:	automatically picked peaks
Used integrals:	peak intensities
Used Gradient strength:	all values (including replicates) used

Table S2. DOSY processing parameters.

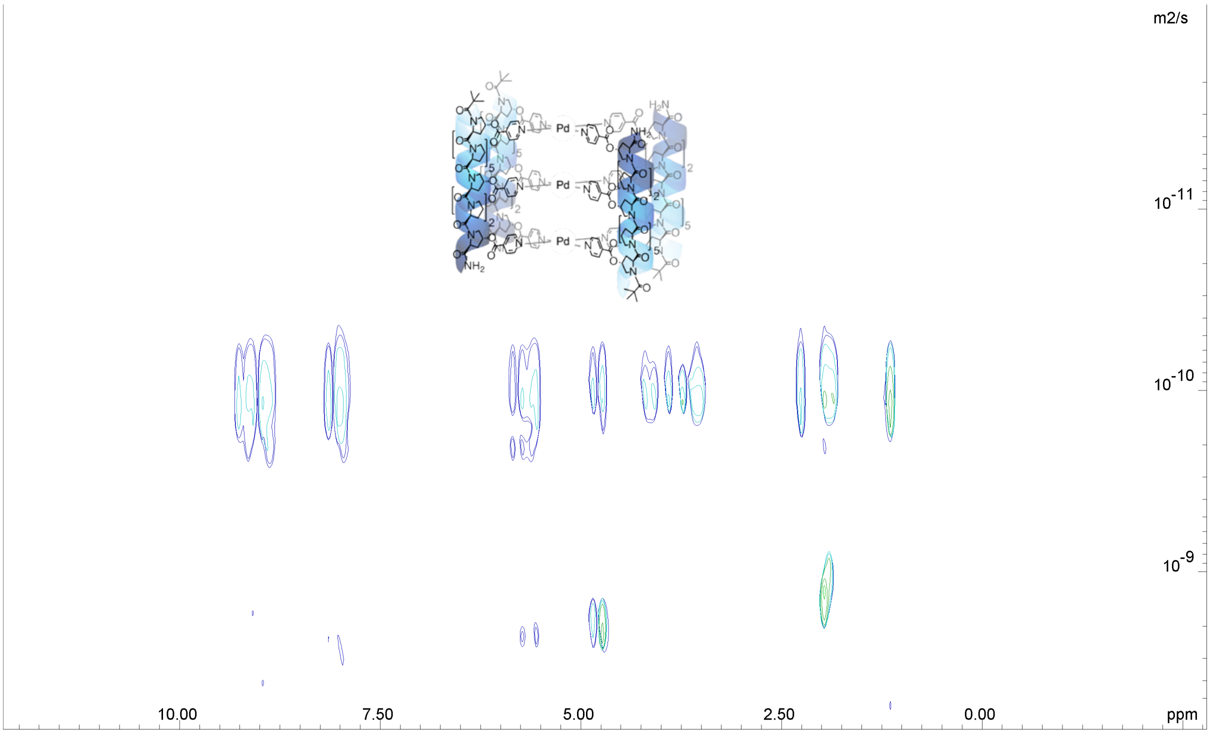

Figure S103. ^{1}H DOSY (D_2O, 600 MHz, 298 K) of **$Pd_3L^{7,R}_4$**$(BF_4)_6$. The diffusion coefficient of of **$Pd_3L^{7,R}_4$**$(BF_4)_6$ was 1.392 x 10^{-10} m^2 s^{-1}.

Peak name	F2 (ppm)	D (m²/s)	Error
1	9.223	1.30E-10	2.19E-12
2	9.212	1.32E-10	2.34E-12
3	9.105	1.28E-10	2.49E-12
4	9.093	1.31E-10	2.83E-12
5	8.943	1.24E-10	1.65E-12
6	8.933	1.28E-10	1.78E-12
7	8.923	1.25E-10	1.95E-12
8	8.911	1.25E-10	2.16E-12
9	8.901	1.27E-10	1.69E-12
10	8.861	1.30E-10	2.29E-12
11	8.853	1.29E-10	1.83E-12
12	8.124	1.31E-10	2.19E-12
13	8.114	1.30E-10	2.66E-12
14	8.002	1.25E-10	2.25E-12
15	7.99	1.24E-10	1.35E-12
16	7.979	1.24E-10	1.02E-12
17	7.966	1.27E-10	1.77E-12
18	7.936	1.25E-10	2.33E-12
19	7.928	1.24E-10	2.61E-12
20	5.86	1.40E-10	4.67E-12
21	5.696	1.40E-10	6.69E-12
22	5.615	1.30E-10	3.18E-12
23	5.561	1.43E-10	5.96E-12
24	4.833	2.90E-10	5.33E-11
25	4.708	1.55E-09	1.83E-10
26	4.221	9.96E-11	8.17E-12
27	4.109	1.12E-10	4.23E-12
28	3.885	1.08E-10	4.31E-12
29	3.721	1.21E-10	5.84E-13
30	3.608	1.18E-10	4.62E-13
31	3.545	1.16E-10	1.13E-12
32	3.515	1.22E-10	3.25E-13
33	2.268	1.21E-10	5.93E-13
34	1.981	1.04E-09	1.04E-10
35	1.943	1.17E-10	5.81E-13
36	1.911	4.29E-10	5.64E-11
37	1.875	1.15E-10	1.31E-12
38	1.169	1.31E-10	5.26E-13

Table S3. ^{1}H DOSY (D_2O, 600 MHz, 298 K) of of **$Pd_3L^{7,R}_4$**$(BF_4)_6$ diffusion coefficients for picked peaks.

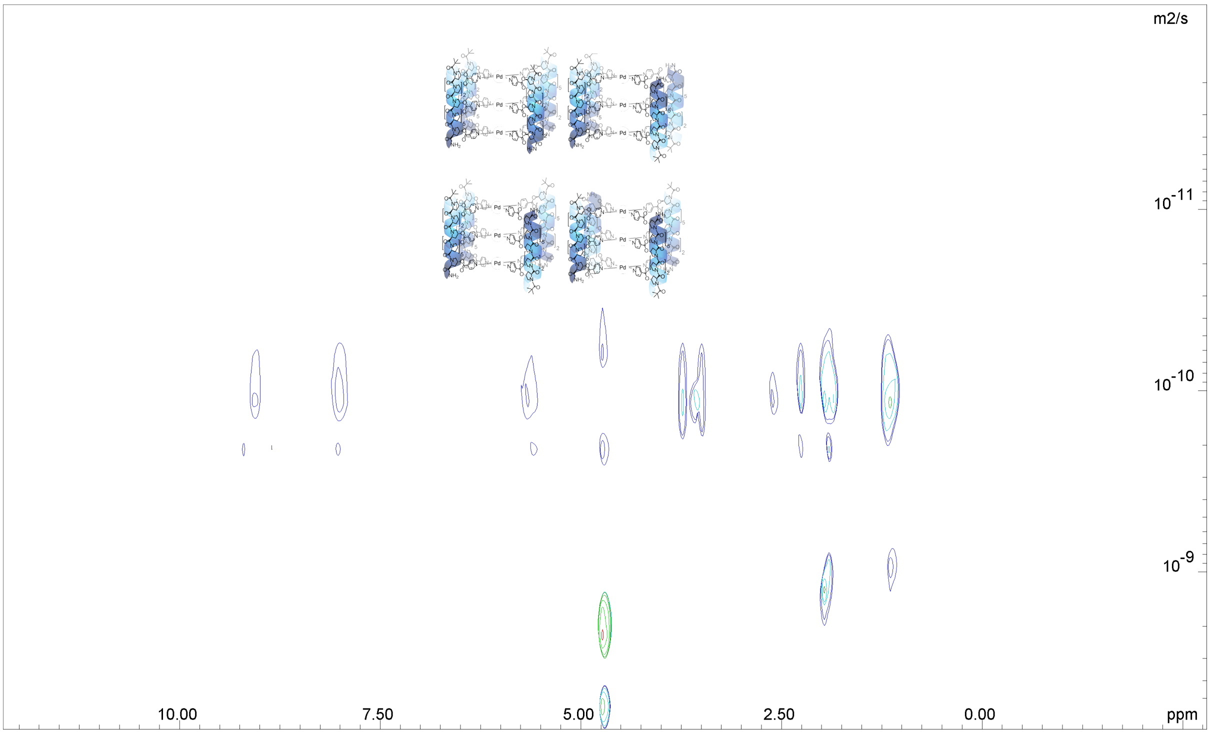

Figure S104. ^{1}H DOSY (D_2O, 600 MHz, 298 K) of **$Pd_3L^{4,R}_4(BF_4)_6$**. The diffusion coefficient of of **$Pd_3L^{4,R}_4(BF_4)_6$** was 1.321 x 10^{-10} m^2 s^{-1}.

Peak name	F2 (ppm)	D (m^2/s)	Error
1	9.178031	1.186E-10	4.49E-12
2	9.0854909	1.165E-10	1.83E-12
3	8.9929509	1.101E-10	1.98E-12
4	8.8171246	1.067E-10	4.01E-12
5	8.7060763	9.752E-11	9.67E-12
6	8.0397872	1.181E-10	1.17E-12
7	7.9287389	1.092E-10	1.5E-12
8	5.6892677	1.226E-10	2.46E-12
9	5.6059814	1.269E-10	3.19E-12
10	5.5226951	1.038E-10	5.96E-12
24	4.7018306	2.174E-09	2.73E-11
23	3.7335613	1.221E-10	8.58E-13
22	3.577179	1.195E-10	1.05E-12
21	3.5034129	1.175E-10	9.91E-13
20	2.5887212	1.188E-10	2.4E-12
19	2.2612017	1.177E-10	9.02E-13
18	1.9838435	3.289E-10	4.9E-11
17	1.9218806	2.042E-10	2E-11
16	1.836313	1.152E-10	4.16E-13
15	1.210781	1.187E-10	5.29E-13
14	1.1989786	1.199E-10	7.27E-13
13	1.1694725	1.182E-10	1.15E-12
12	1.1399664	1.481E-10	3.02E-12

11	1.1075088	1.25E-10	2.25E-12

Table S4. ^{1}H DOSY (D_2O, 600 MHz, 298 K) of **Pd$_3$L^{4,R}$_4$**$(BF_4)_6$ diffusion coefficients for picked peaks.

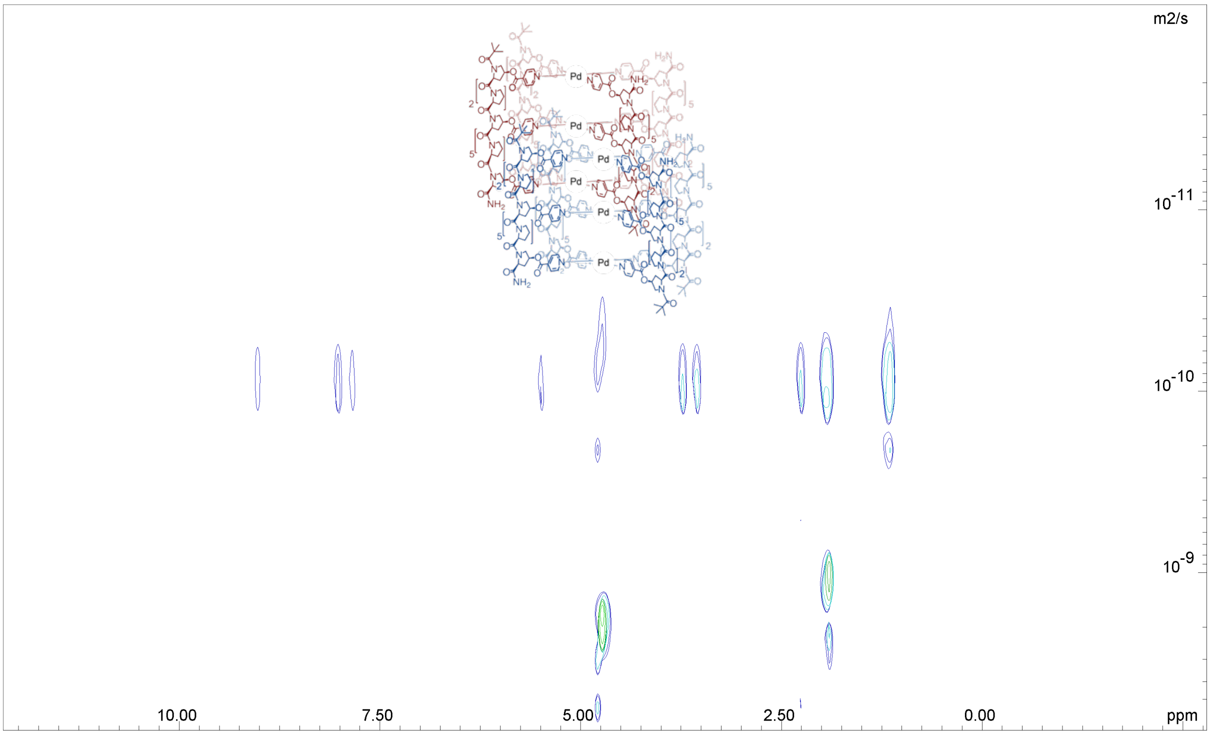

Figure S105. ^{1}H DOSY (D_2O, 600 MHz, 298 K) of [**Pd$_3$L^{7,S}$_4$**$(BF_4)_6]_2$. The diffusion coefficient of [**Pd$_3$L^{7,S}$_4$**$(BF_4)_6]_2$ was 1.187 x 10^{-10} m^2 s^{-1}.

Peak name	F2 (ppm)	D (m²/s)	Error
1	9.0207129	1.129E-10	5.33E-12
2	7.9935173	9.938E-11	1.95E-12
3	7.8084367	1.149E-10	6.36E-12
4	5.4949332	6.319E-11	2.32E-11
12	4.7649708	8.336E-10	2.45E-10
13	4.7026318	1.605E-09	8.66E-11
11	3.7371731	1.042E-10	2.1E-12
10	3.546959	1.08E-10	9.55E-13
9	2.2536739	1.136E-10	3.04E-12
8	1.9844581	1.764E-10	1.86E-11
7	1.920145	8.651E-10	7.21E-11
6	1.2052278	1.129E-10	1.11E-12
5	1.1543759	1.07E-10	5.94E-13

Table S5. ^{1}H DOSY (D_2O, 600 MHz, 298 K) of [**Pd$_3$L^{7,S}$_4$**$(BF_4)_6]_2$ diffusion coefficients for picked peaks.

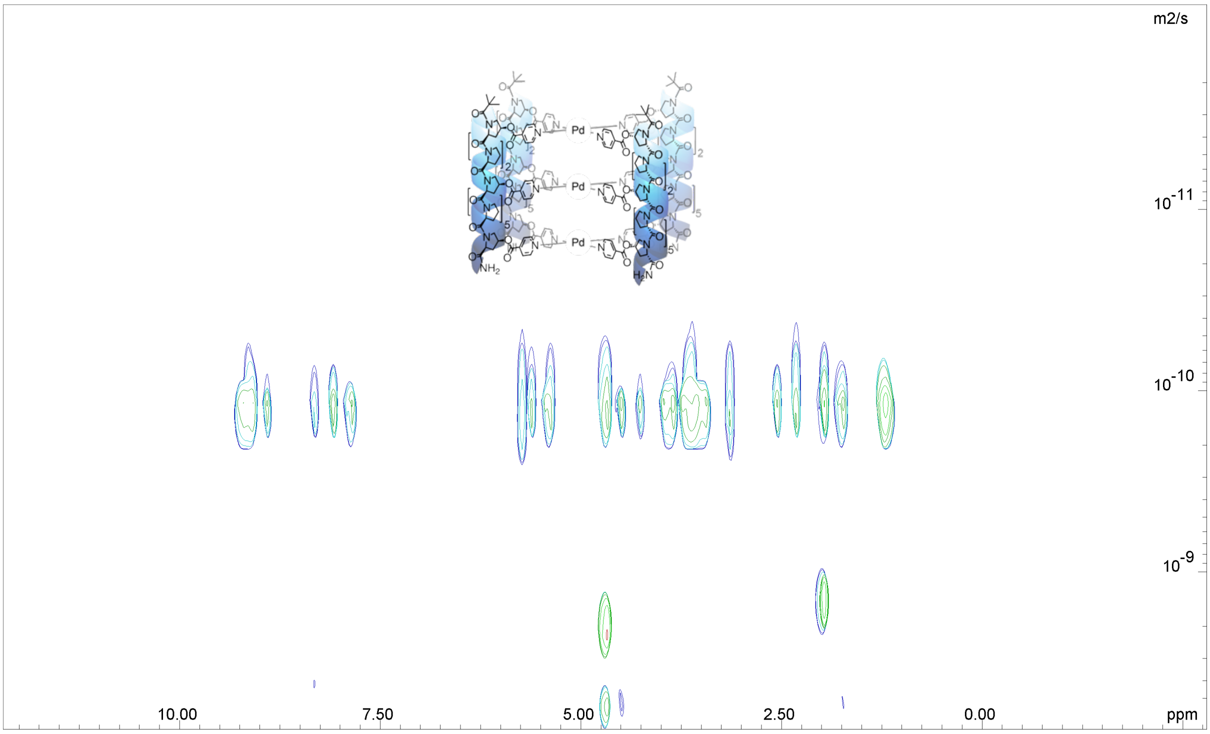

Figure S106. ^{1}H DOSY (D_2O, 600 MHz, 298 K) of **$Pd_3L^{4,S}{}_4$**$(BF_4)_6$. The diffusion coefficient of **$Pd_3L^{4,S}{}_4$**$(BF_4)_6$ was 1.419 x 10^{-10} m^2 s^{-1}.

Peak name	F2 (ppm)	D (m²/s)	Error
1	9.2523603	1.427E-10	1.83E-12
2	9.2064123	1.425E-10	1.5E-12
3	9.1439227	1.433E-10	2.17E-12
4	9.1090021	1.43E-10	2.27E-12
5	9.0575402	1.412E-10	1.43E-12
6	8.893965	1.42E-10	1.75E-12
9	8.2948015	1.551E-10	5.55E-12
7	8.0540334	1.409E-10	1.7E-12
8	7.8628888	1.418E-10	1.07E-12
10	5.7150936	1.416E-10	2.53E-12
11	5.5979723	1.451E-10	1.95E-12
12	5.4144825	1.393E-10	3.4E-12
13	5.3734902	1.441E-10	2.48E-12
14	4.6961401	1.853E-09	1.7E-10
15	4.5067938	1.446E-10	2.51E-12
16	4.2686479	1.392E-10	2.82E-12
17	3.9426601	1.417E-10	1.08E-12
18	3.8723876	1.393E-10	1.63E-12
19	3.7318427	1.398E-10	9.81E-13
20	3.647906	1.404E-10	9.36E-13
21	3.5952012	1.397E-10	1.06E-12
22	3.5249287	1.411E-10	5.8E-13
23	3.4585597	1.426E-10	1.05E-12

24	3.1442848	1.453E-10	2.28E-12
25	2.5682476	1.405E-10	1.97E-12
26	2.3343201	1.394E-10	1.63E-12
27	1.9895854	8.94E-10	1.04E-10
28	1.7626935	1.425E-10	1.14E-12
29	1.2491094	1.372E-10	2.79E-12
30	1.2262444	1.395E-10	2E-12
31	1.1365434	1.391E-10	5.63E-13

Table S6. ^{1}H DOSY (D_2O, 600 MHz, 298 K) of $\mathbf{Pd_3L^{4,S}_4}(BF_4)_6$ diffusion coefficients for picked peaks.

7. Cage Stabilities

$\mathbf{Pd_3L^{7,R}_4}(BF_4)_6$, $\mathbf{Pd_3L^{4,R}_4}(BF_4)_6$ and $\mathbf{Pd_3L^{4,S}_4}(BF_4)_6$ showed initial broadness in ^{1}H NMR that resolved over time. Minimal change was observed in cage [$\mathbf{Pd_3L^{7,S}_4}(BF_4)_6$]$_2$ after 7 days. Figures S107 – S114 show this in greater detail.

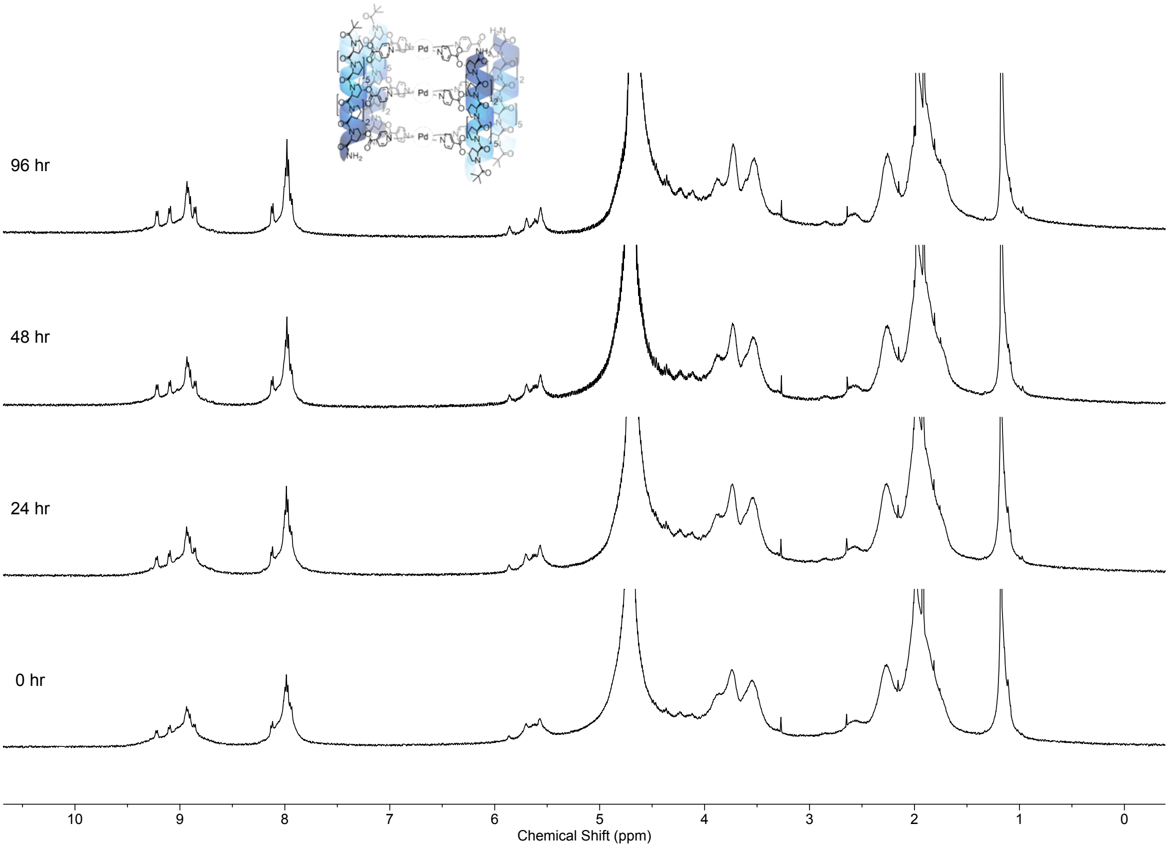

Figure S107. Stacked ^{1}H NMR (D_2O, 600 MHz, 298 K) of cage $\mathbf{Pd_3L^{7,R}_4}(BF_4)_6$ over time at 298 K.

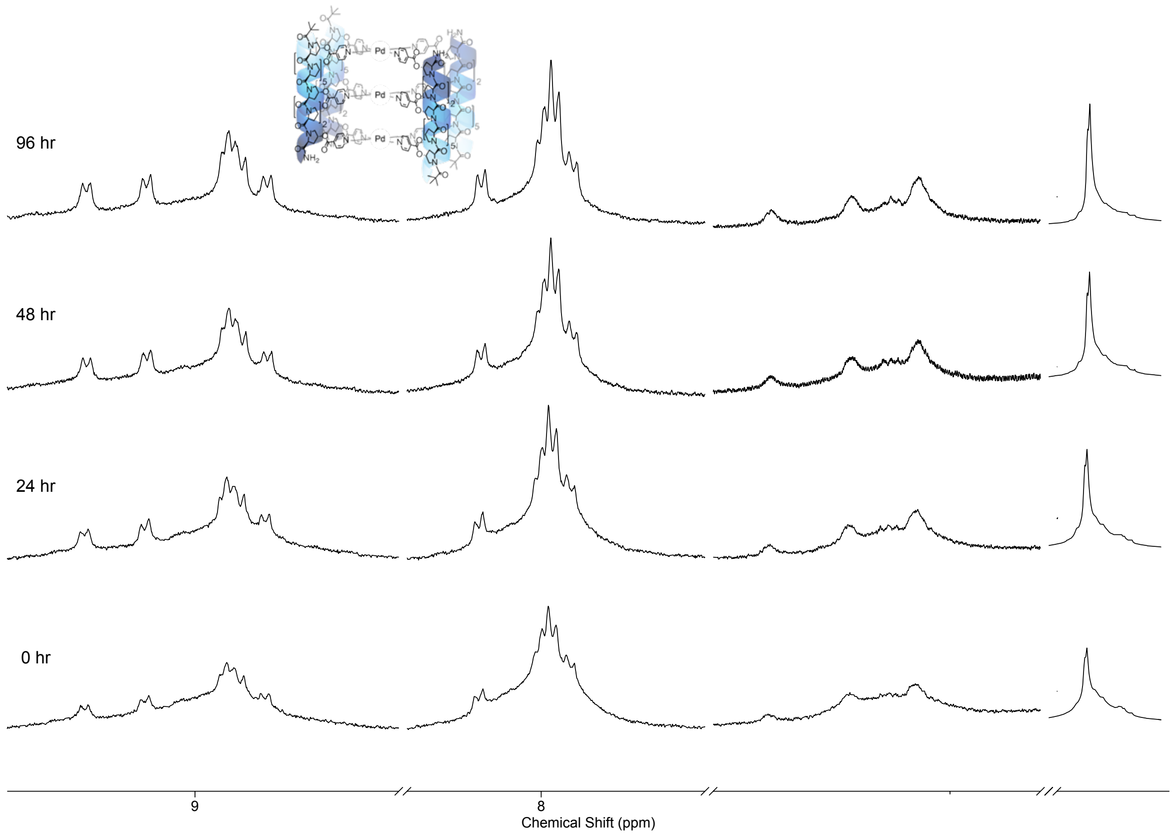

Figure S108. Partial stacked ^{1}H NMR (D_2O, 600 MHz, 298 K) of cage **$Pd_3L^{7,R}_4$**$(BF_4)_6$ over time at 298 K. Peaks at ~1 ppm have been scaled independently to rest of spectra due to relative intensity.

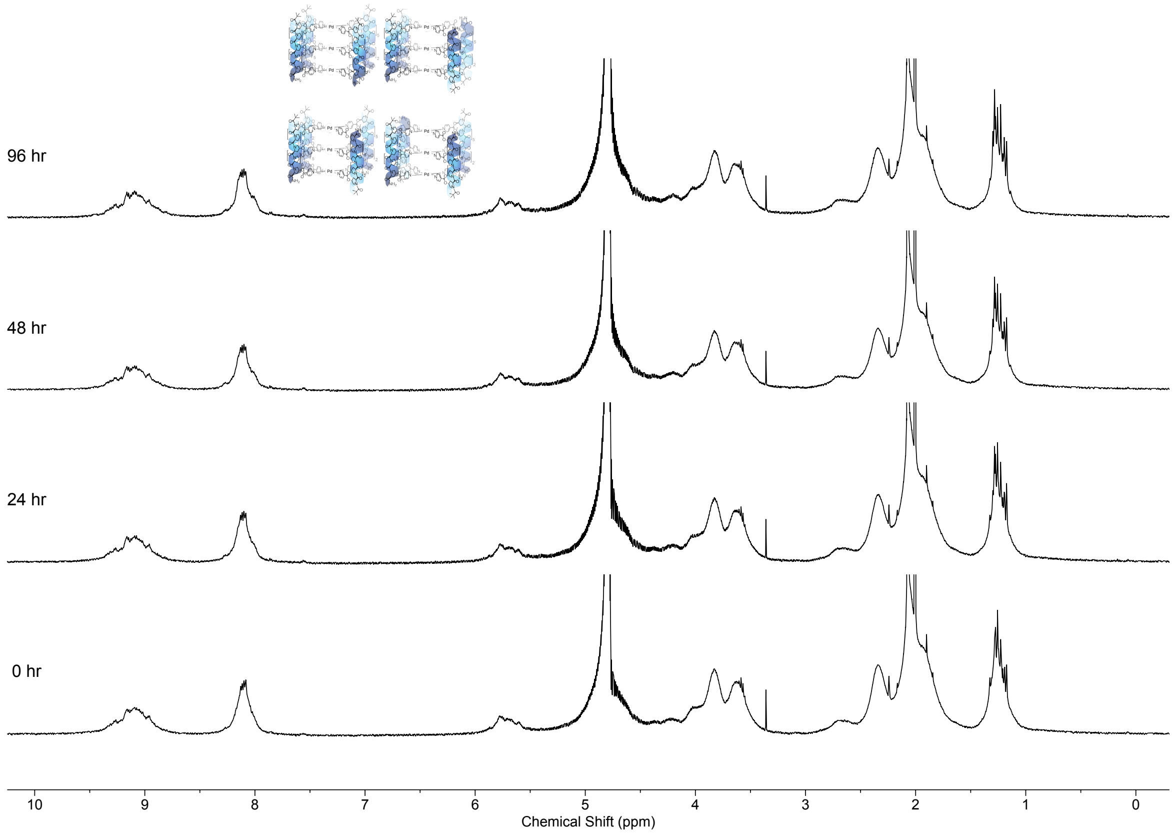

Figure S109. Stacked ^{1}H NMR (D_2O, 600 MHz, 298 K) of cage **$Pd_3L^{4,R}_4$**$(BF_4)_6$ over time at 298 K.

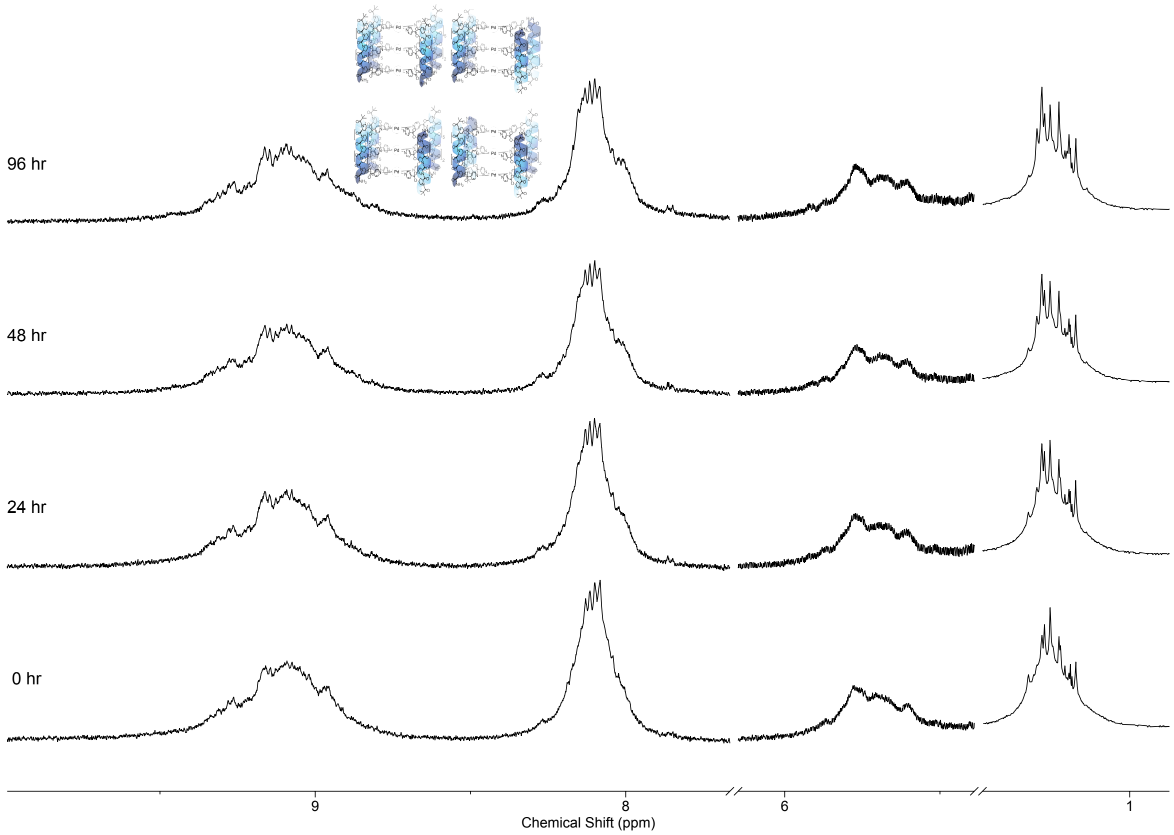

Figure S110. Partial stacked ^{1}H NMR (D_2O, 600 MHz, 298 K) of cage $\mathbf{Pd_3L^{4,R}{}_4}(BF_4)_6$ over time at 298 K. Peaks at ~1 ppm have been scaled independently to rest of spectra due to relative intensity.

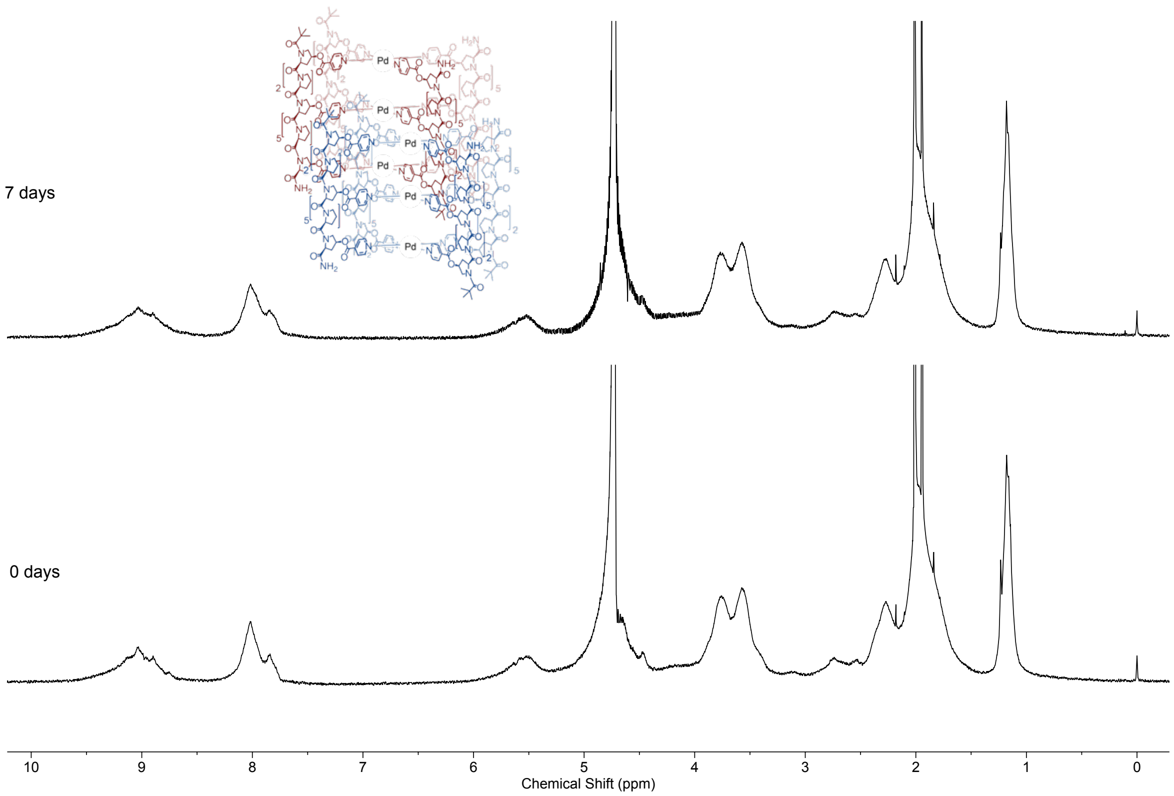

Figure S111. Stacked ^{1}H NMR (D_2O, 600 MHz, 298 K) of cage $[\mathbf{Pd_3L^{7,S}{}_4}(BF_4)_6]_2$ over time at 298 K.

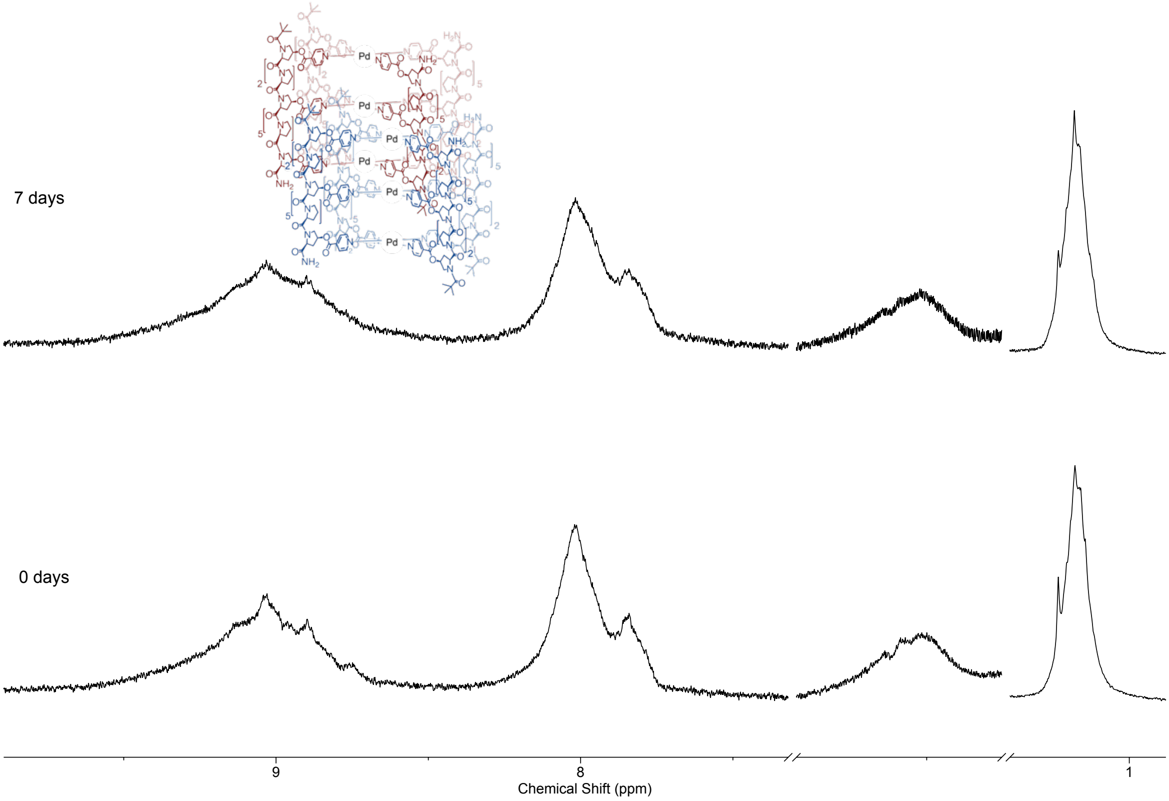

Figure S112. Partial stacked ^{1}H NMR (D_2O, 600 MHz, 298 K) of cage [$\mathbf{Pd_3L^{7,S}_4}(BF_4)_6]_2$ over time at 298 K. Peaks at ~1 ppm have been scaled independently to rest of spectra due to relative intensity.

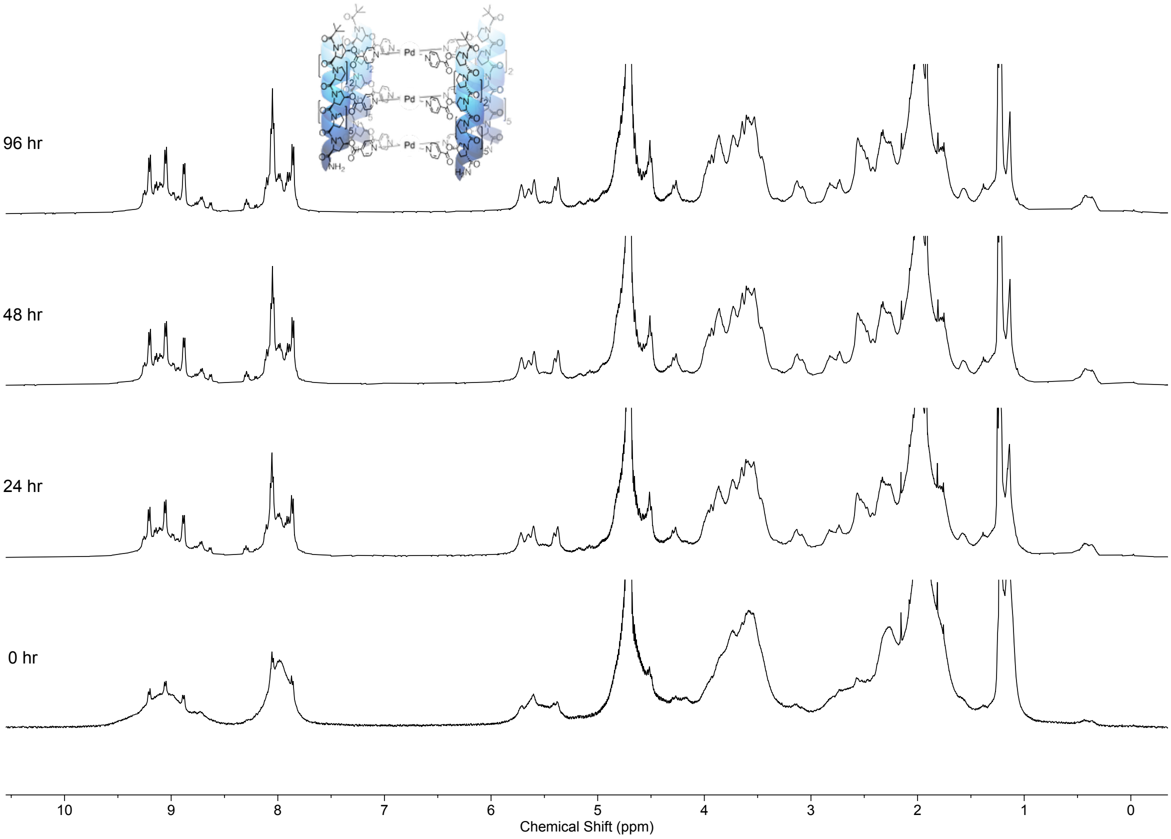

Figure S113. Stacked ^{1}H NMR (D_2O, 600 MHz, 298 K) of cage $\mathbf{Pd_3L^{4,S}_4}(BF_4)_6$ over time at 298 K.

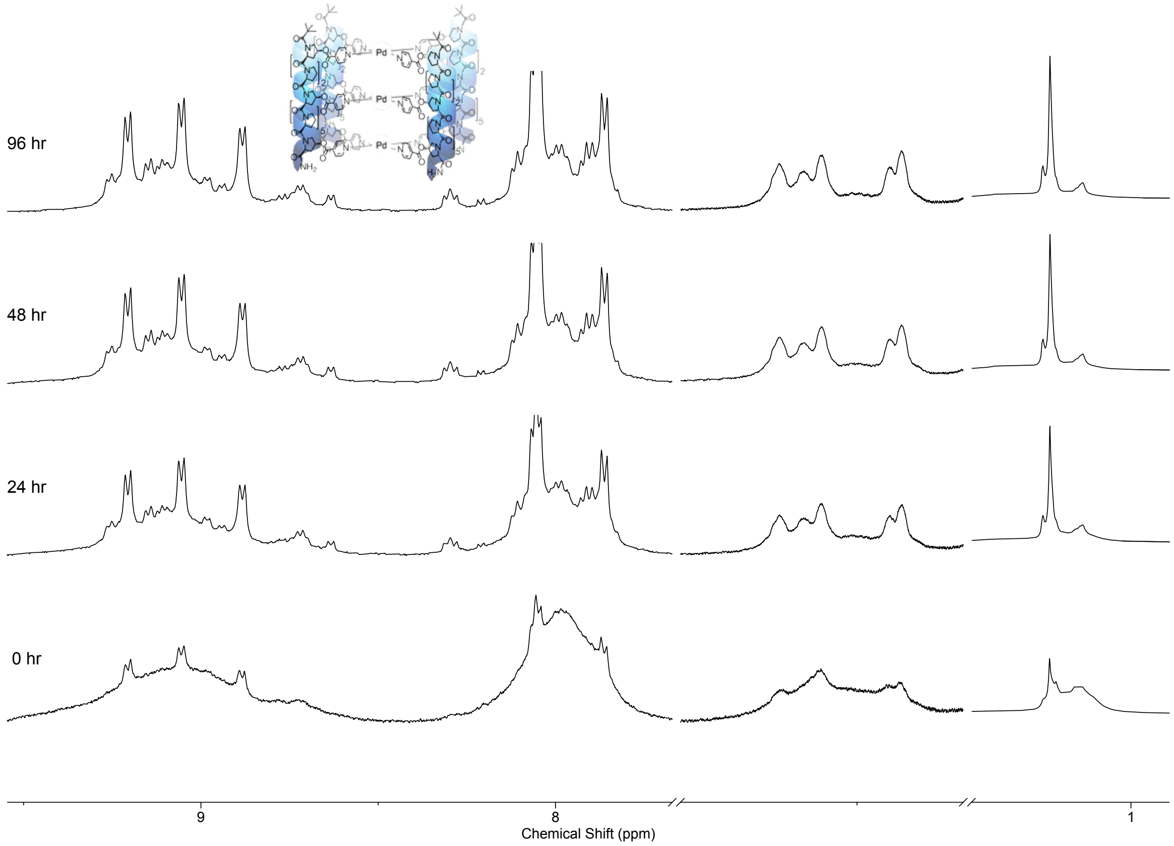

Figure S114. Partial stacked ^{1}H NMR (D_2O, 600 MHz, 298 K) of cage $\mathbf{Pd_3L^{4,\mathit{S}}_4}(BF_4)_6$ over time at 298 K. Peaks at ~1 ppm have been scaled independently to rest of spectra due to relative intensity.

Dilution Study

To determine cage stability towards dilution, stock solutions of each of four cages were diluted with D_2O from 1000 to 20 μM and monitored by ^{1}H NMR. ^{1}H NMR was recorded immediately after dilution in each case. Cages were deemed unstable at concentrations where NMR signals for free ligand appeared.

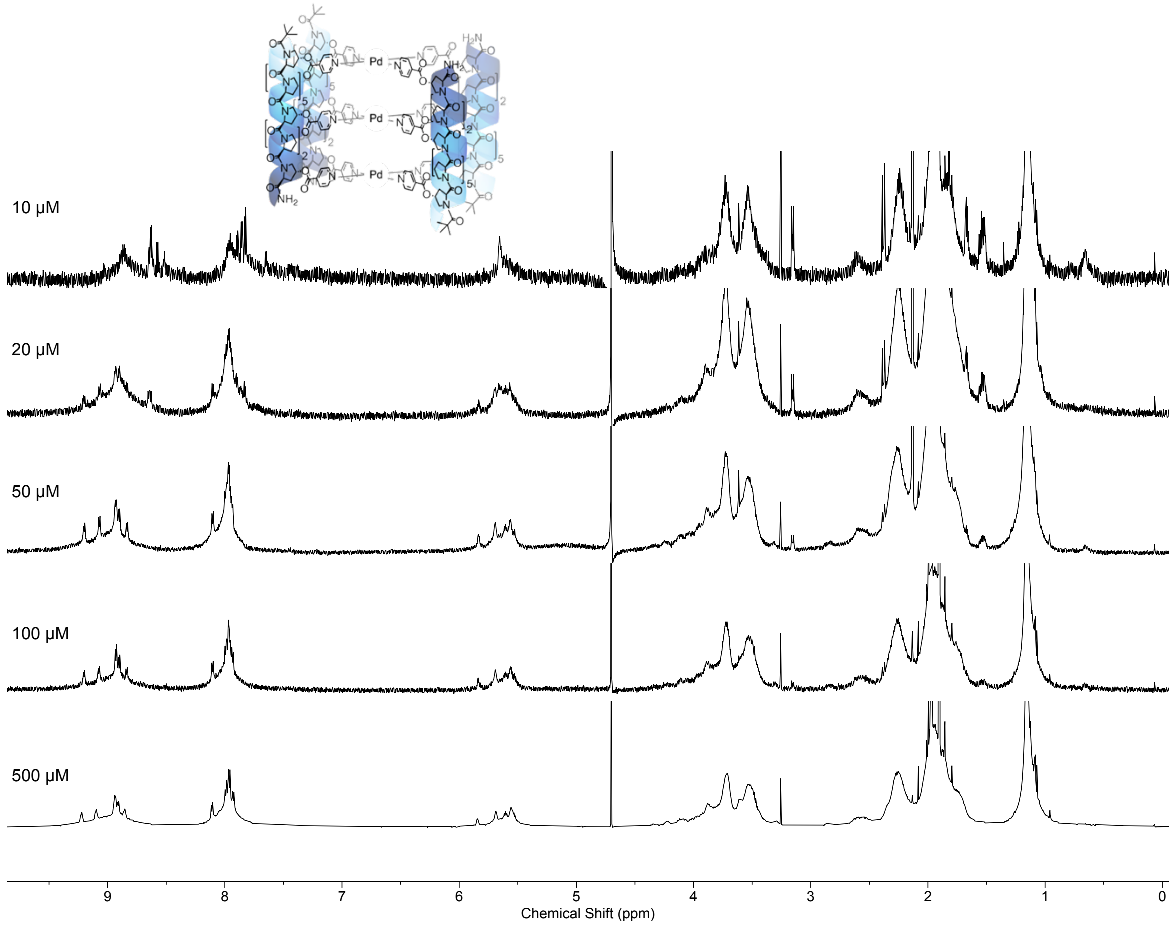

Figure S115. Stacked ^{1}H NMR (D_2O, 600 MHz, 298 K) of cage $\mathbf{Pd_3L^{7,R}_4}(BF_4)_6$ at various concentrations to determine stability to dilution, showing appearance of free ligand signals at 8.65 ppm below concentrations of 50 μM. Water suppression NMR has been used for clarity.

\

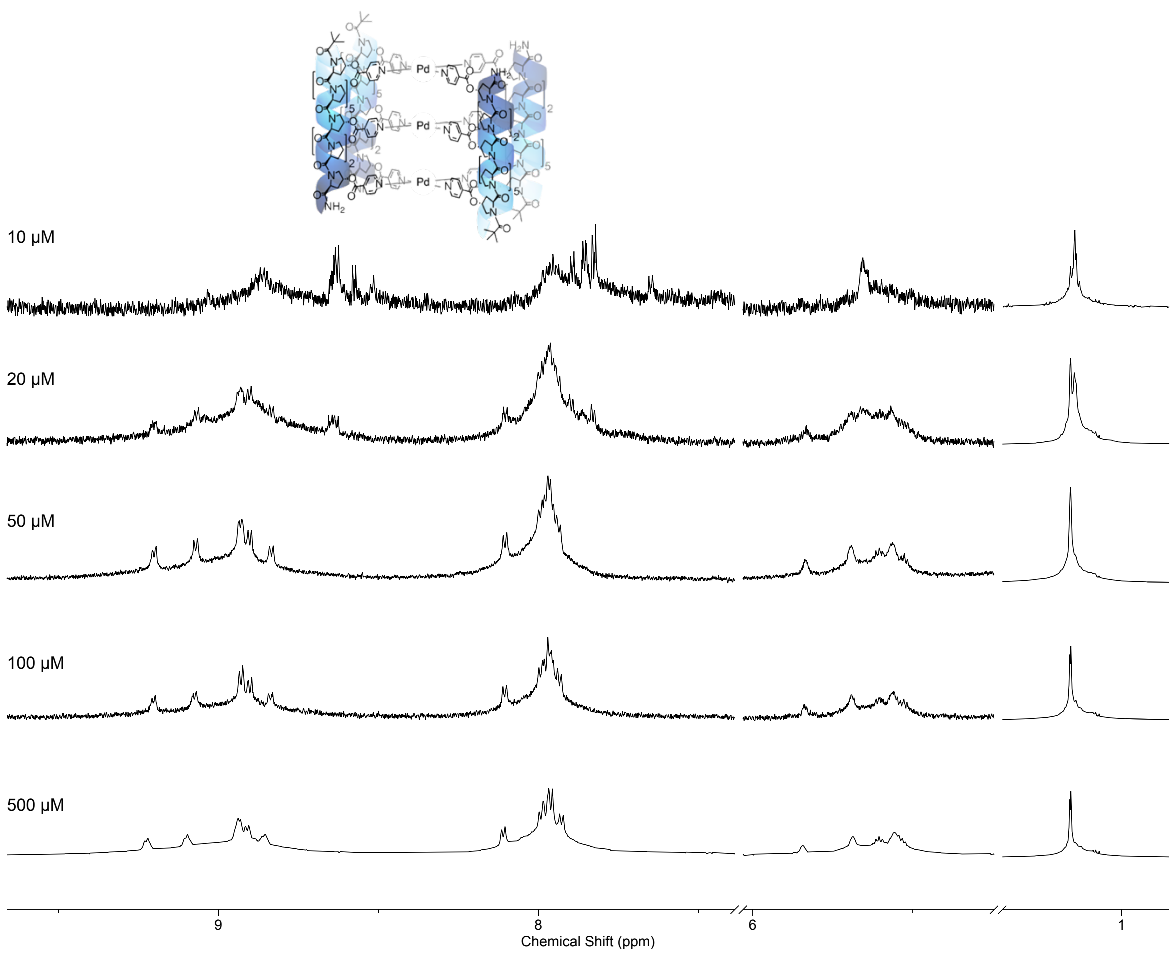

Figure S116. Partial stacked ^{1}H NMR (D_2O, 600 MHz, 298 K) of cage $\mathbf{Pd_3L^{7,R}_4}(BF_4)_6$ at various concentrations to determine stability to dilution, showing appearance of free ligand signals at 8.65 ppm below concentrations of 50 μM. Peaks at ~1 ppm have been scaled independently to rest of spectra due to relative intensity. Water suppression NMR has been used for clarity.

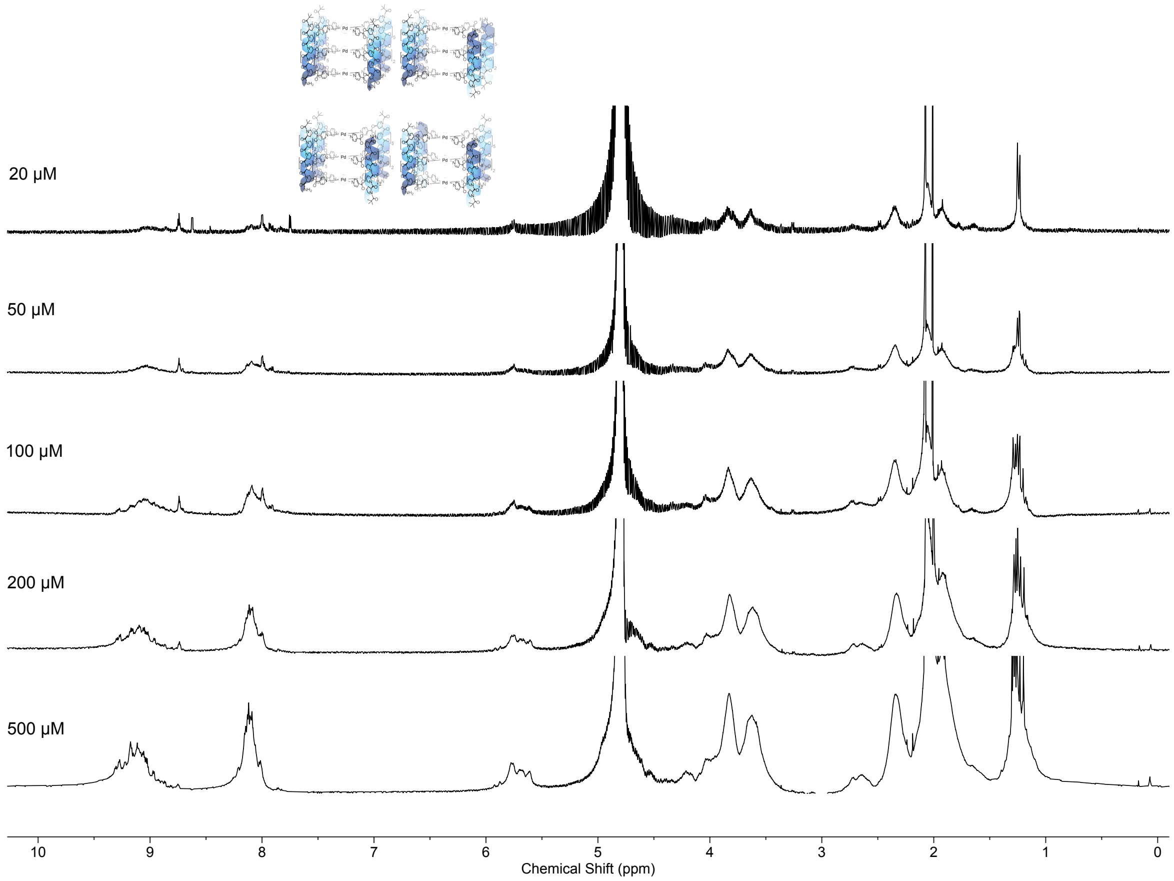

Figure S117. Stacked ^{1}H NMR (D_2O, 600 MHz, 298 K) of cage **$Pd_3L^{4,R}_4$**$(BF_4)_6$ at various concentrations to determine stability to dilution, showing appearance of free ligand signals at 8.74 ppm below concentrations of 500 μM.

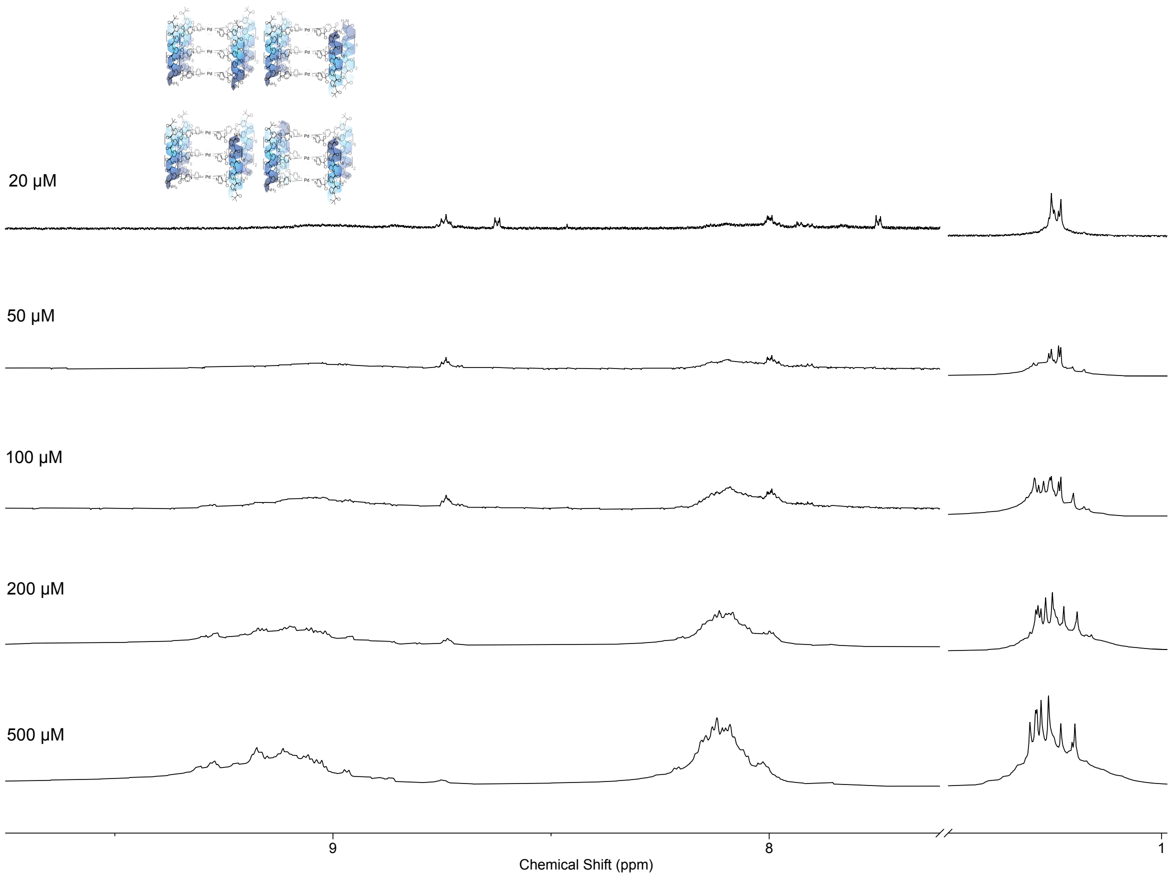

Figure S118. Stacked ^{1}H NMR (D_2O, 600 MHz, 298 K) of cage $\mathbf{Pd_3L^{4,\mathit{R}}_4}(BF_4)_6$ at various concentrations to determine stability to dilution, showing appearance of free ligand signals at 8.74 ppm below concentrations of 500 μM. Peaks at ~1 ppm have been scaled independently to rest of spectra due to relative intensity.

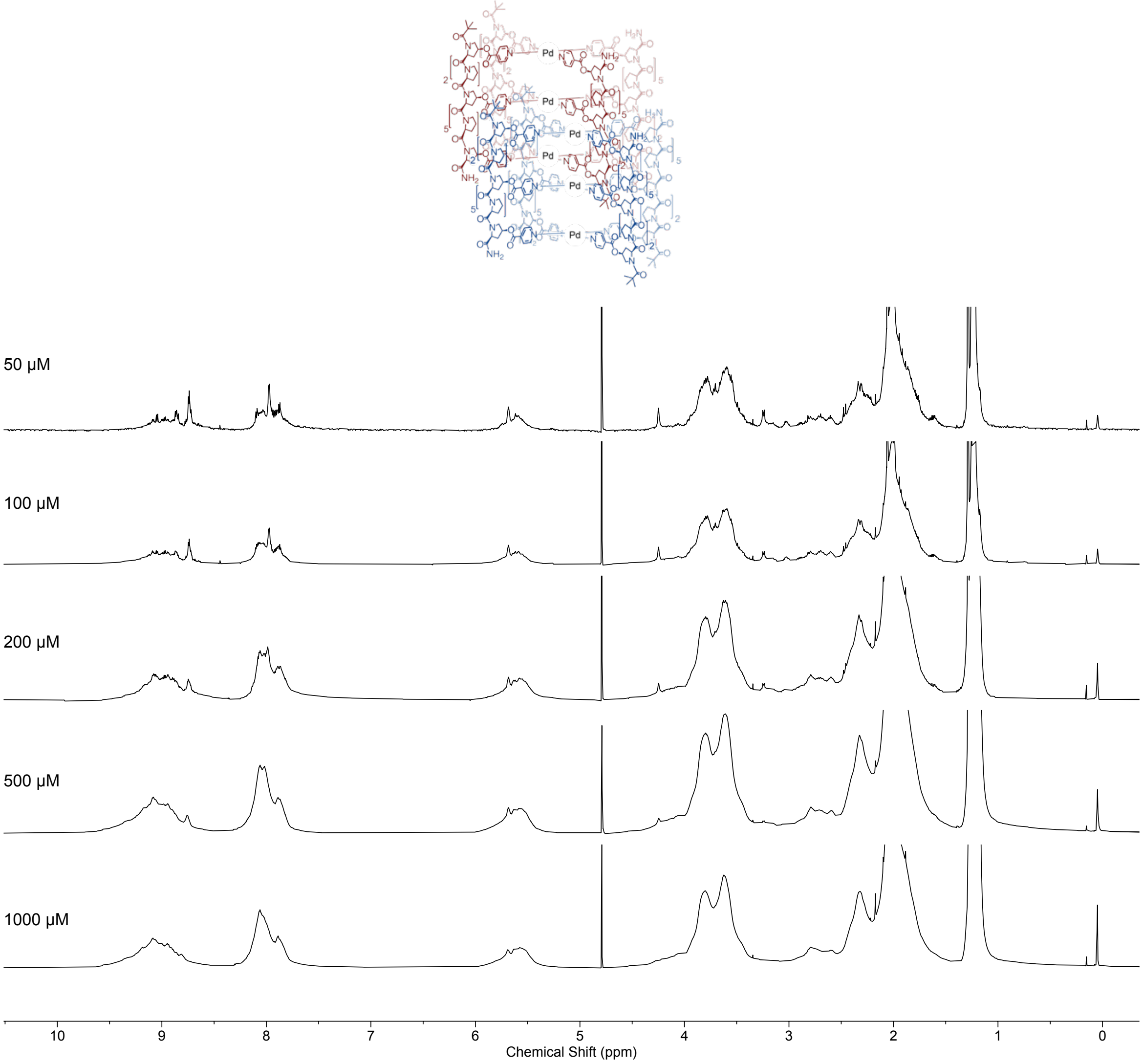

Figure S119. Stacked ^{1}H NMR (D_2O, 600 MHz, 298 K) of cage $[\mathbf{Pd_3L^{7,S}_4}(BF_4)_6]_2$ at various concentrations to determine stability to dilution, showing appearance of free ligand signals at 8.79 ppm below concentrations of 1000 μM. Water suppression NMR has been used for clarity.

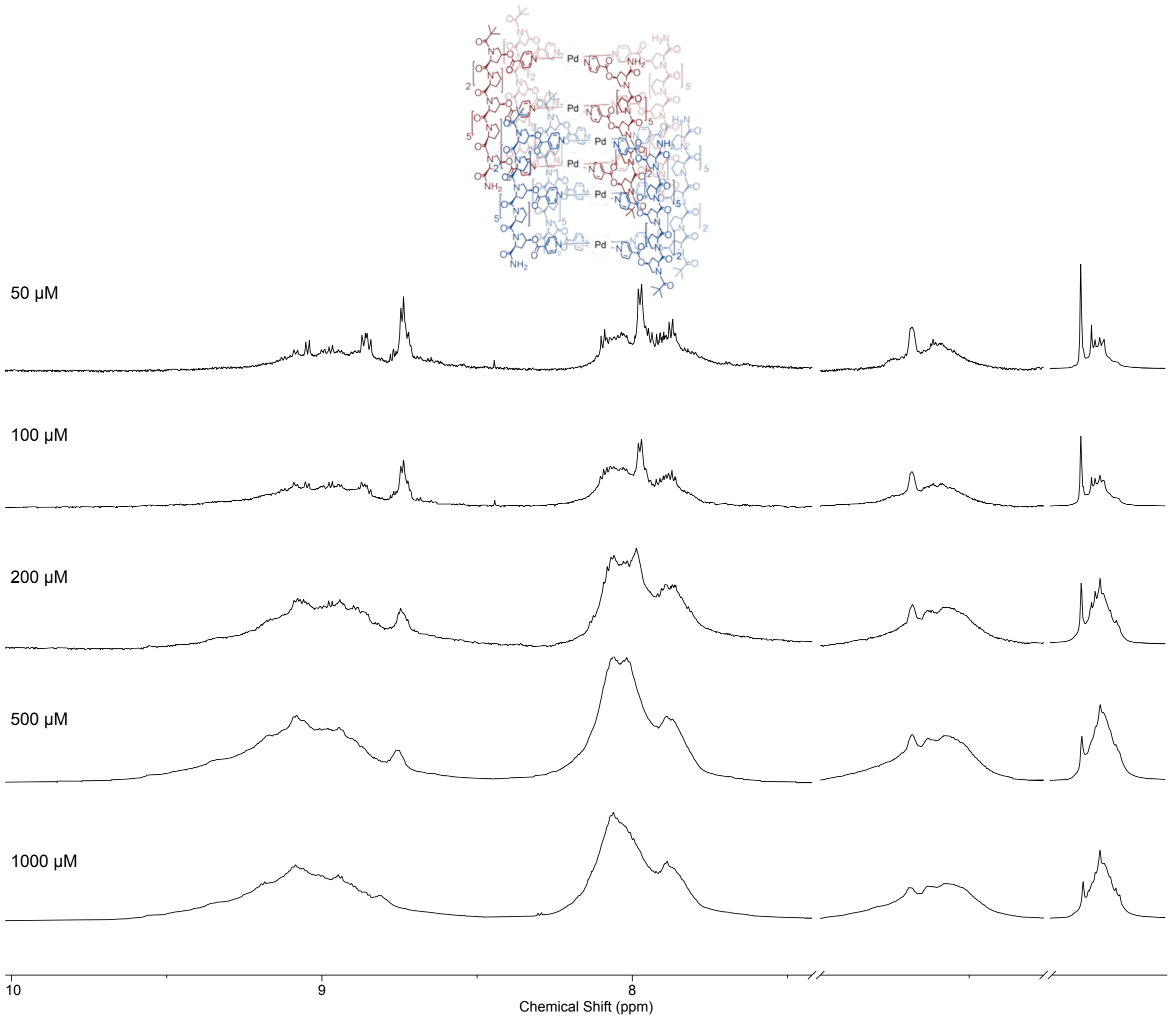

Figure S120. Partial stacked ^{1}H NMR (D_2O, 600 MHz, 298 K) of cage [$\mathbf{Pd_3L^{7,S}_4}(BF_4)_6$]$_2$ at various concentrations to determine stability to dilution, showing appearance of free ligand signals at 8.79 ppm below concentrations of 1000 *µ*M. Peaks at ~1 ppm have been scaled independently to rest of spectra due to relative intensity. Water suppression NMR has been used for clarity.

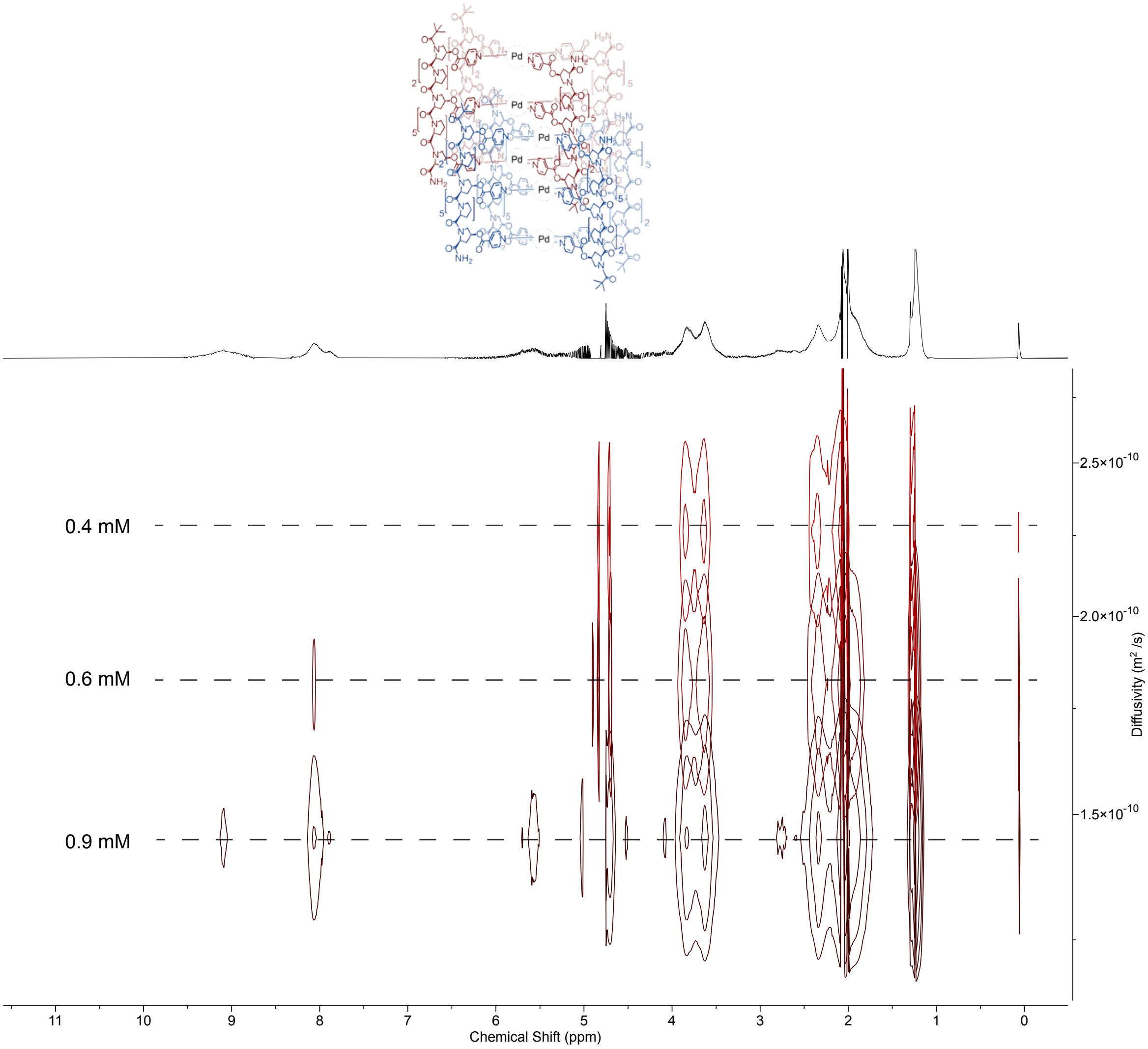

Figure S121. Stacked DOSY NMR (D_2O, 600 MHz, 298 K) of cage $[\mathbf{Pd_3L^{7,S}_4}(BF_4)_6]_2$ at 0.8, 0.4 and 0.2 mM showing increase in proportion of monomeric $[\mathbf{Pd_3L^{7,S}_4}(BF_4)_6]_2$ in equilibrium upon dilution, reflected in larger diffusion coefficients upon dilution. The diffusion coefficient of the obtained species at 0.9, 0.6 and 0.4 mM respectively were 1.450 x 10^{-9}, 1.826 x 10^{-9} and 2.267 x 10^{-9} m^2 s^{-1}, giving hydrodynamii radii of 22.5, 17.9 and 14.4 Å.

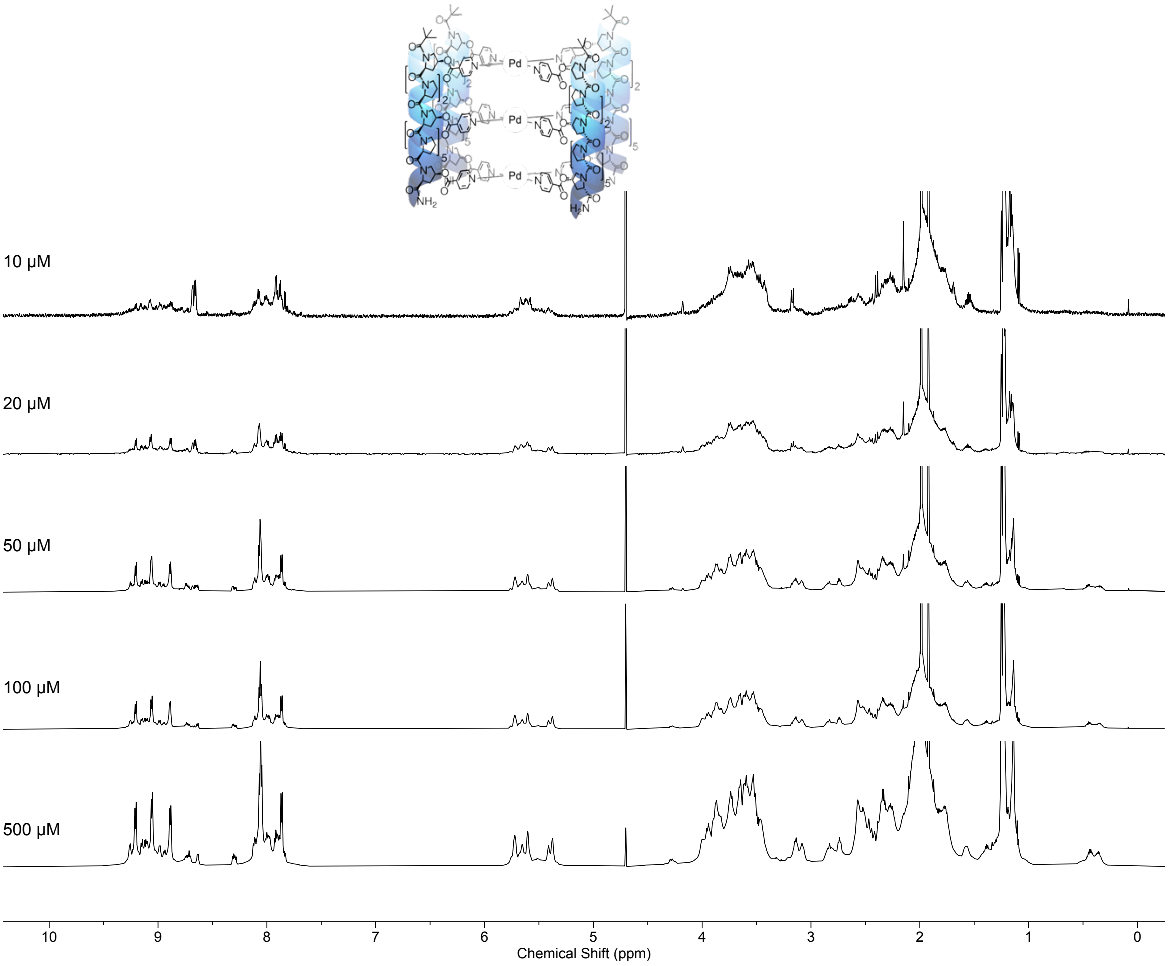

Figure S122. Stacked ^{1}H NMR (D_2O, 600 MHz, 298 K) of cage **$Pd_3L^{4,S}_4$**$(BF_4)_6$ at various concentrations to determine stability to dilution, showing appearance of free ligand signals at 8.69 ppm below concentrations of 50 μM. Water suppression NMR has been used for clarity.

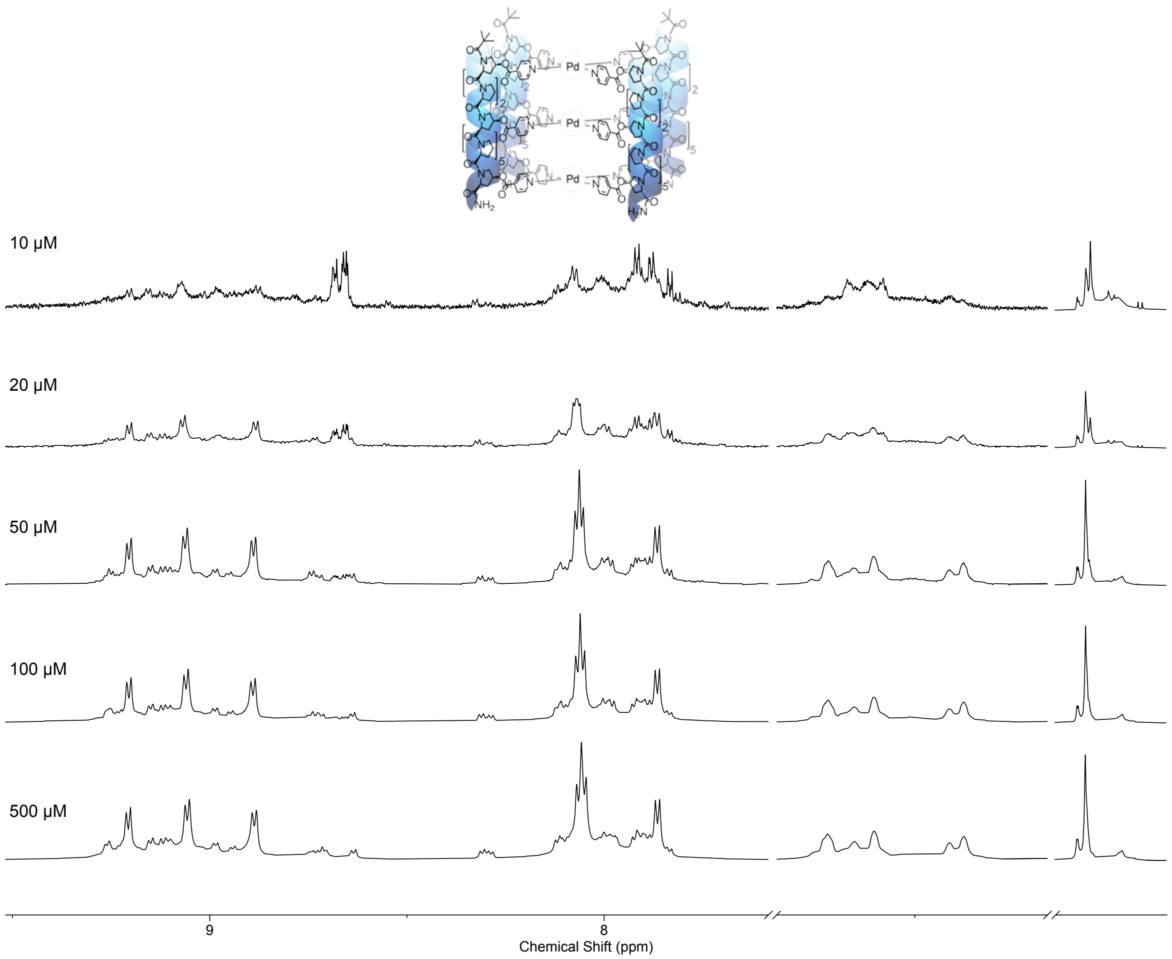

Figure S123. Partial stacked ^{1}H NMR (D_2O, 600 MHz, 298 K) of cage **$Pd_3L^{4,\mathit{S}}_4$**$(BF_4)_6$ at various concentrations to determine stability to dilution showing appearance of free ligand signals at 8.69 ppm below concentrations of 50 μM. Peaks at ~1 ppm have been scaled independently to rest of spectra due to relative intensity. Water suppression NMR has been used for clarity.

To determine cage stability DCl (Figures S124 – S132), NaOD (Figures S133 – S142), pyridine (Figures S143 – S154), and glutathione (reduced form) (Figures S155 – S162) were added separately to 0.250 mM solution of all four cage species and monitored by ^{1}H NMR.

Acid Stability Studies

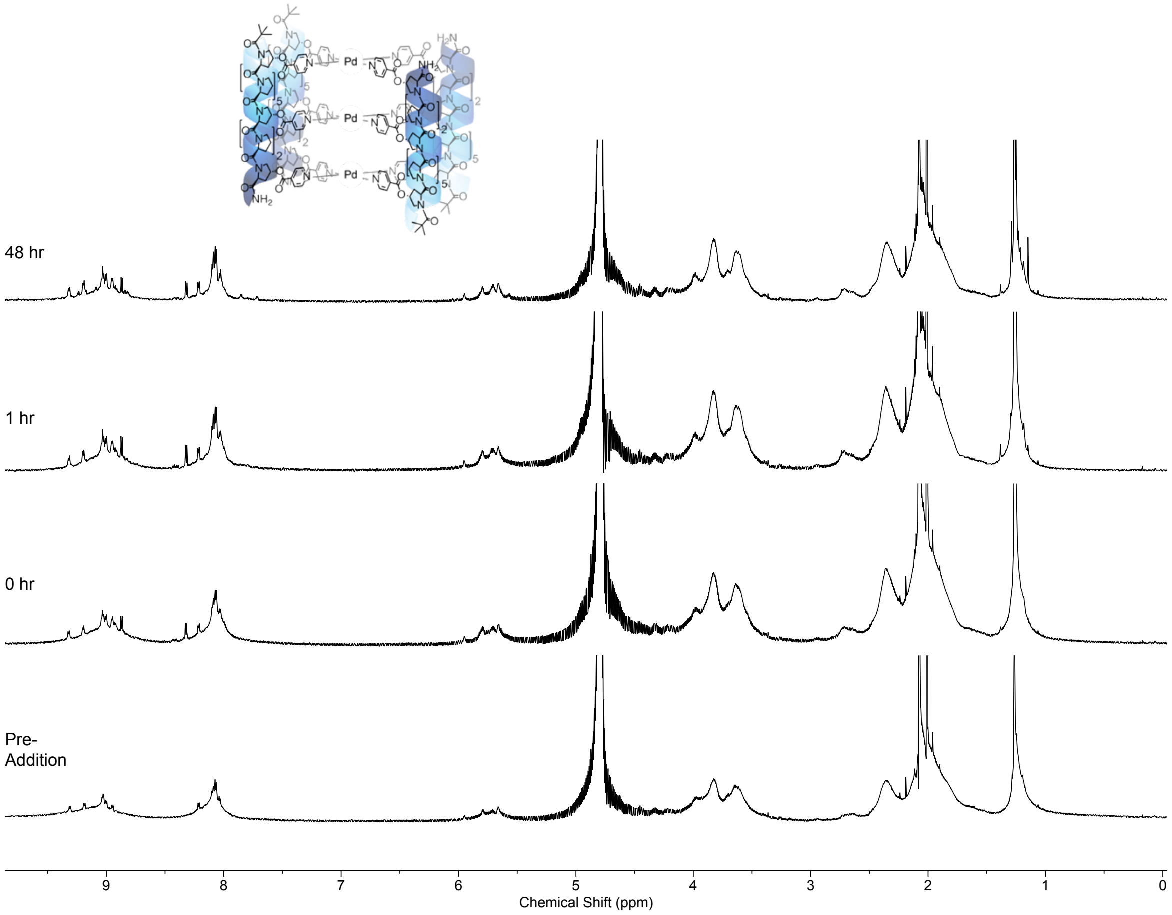

Figure S124. Stacked ^{1}H NMR (D_2O, 600 MHz, 298 K) of cage $\mathbf{Pd_3L^{7,R}_4}(BF_4)_6$ before (bottom) and after addition of 4 eq DCl over time at 298 K.

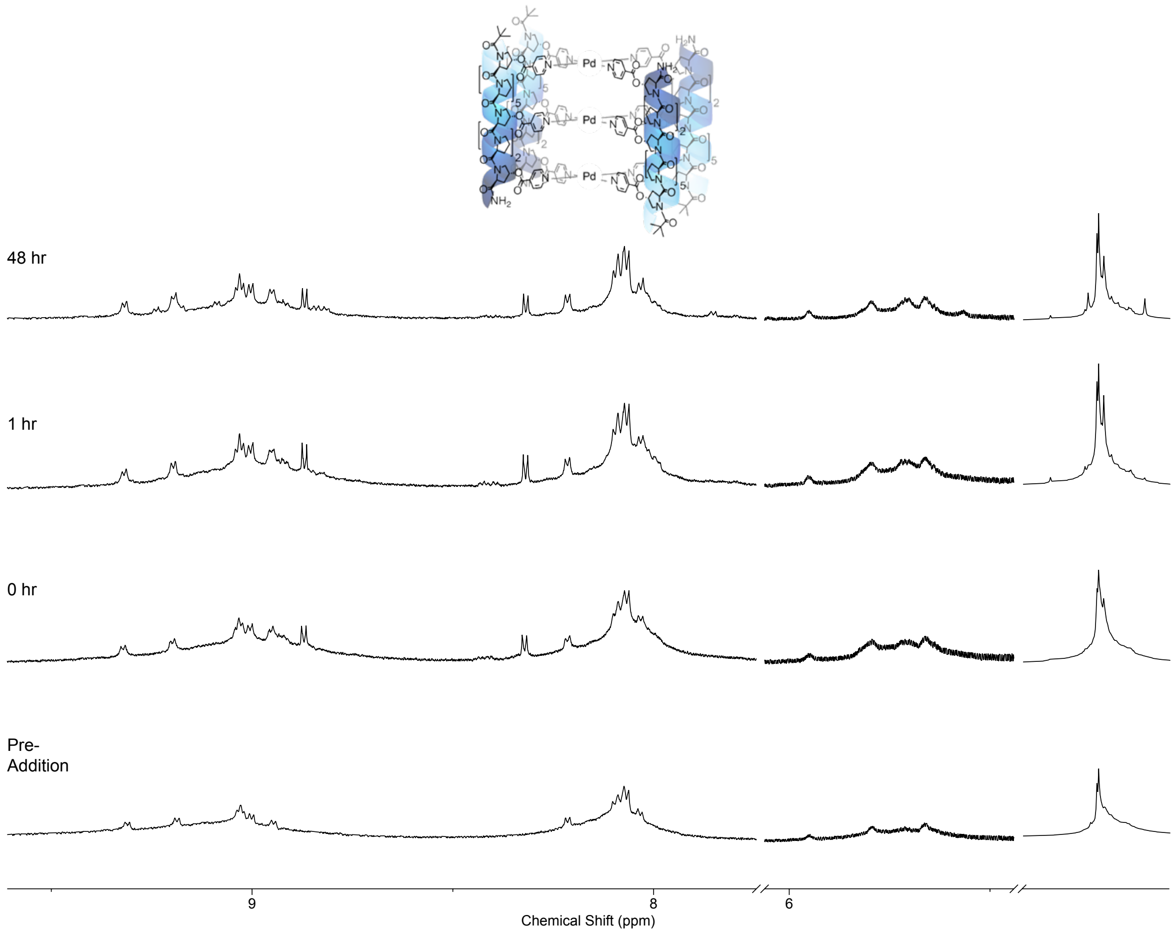

Figure S125. Partial stacked ^{1}H NMR (D_2O, 600 MHz, 298 K) of cage **$Pd_3L^{7,R}_4$**$(BF_4)_6$ $(BF_4)_6$ before (bottom) and after addition of 4 eq DCl over time at 298 K. Peaks at ~1 ppm have been scaled independently to rest of spectra due to relative intensity.

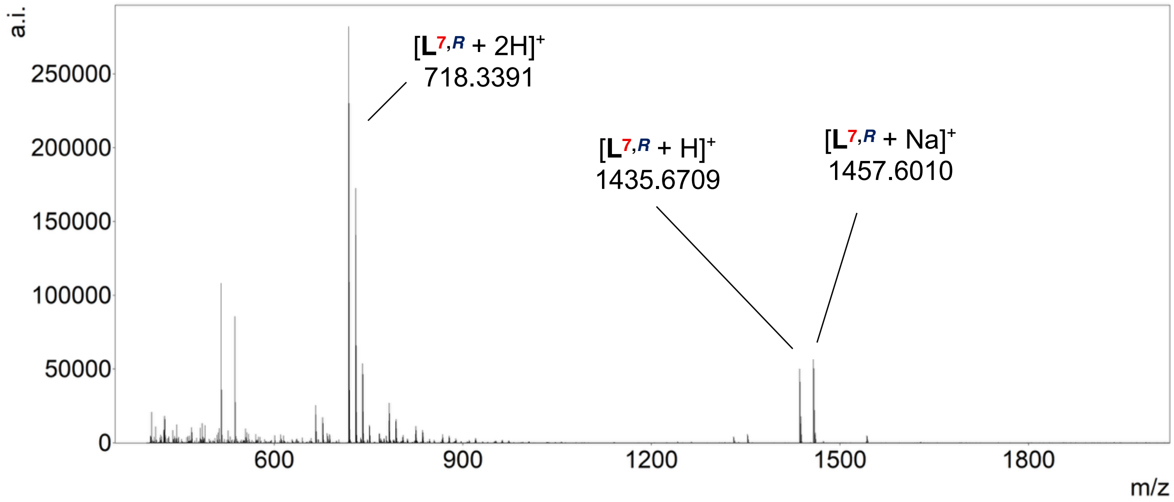

Figure S126. ESI-MS (+ve ion, H_2O/D_2O) of cage **$Pd_3L^{7,R}_4$**$(BF_4)_6$ after addition of 12 eq. HCl after ~30 mins at 298 K

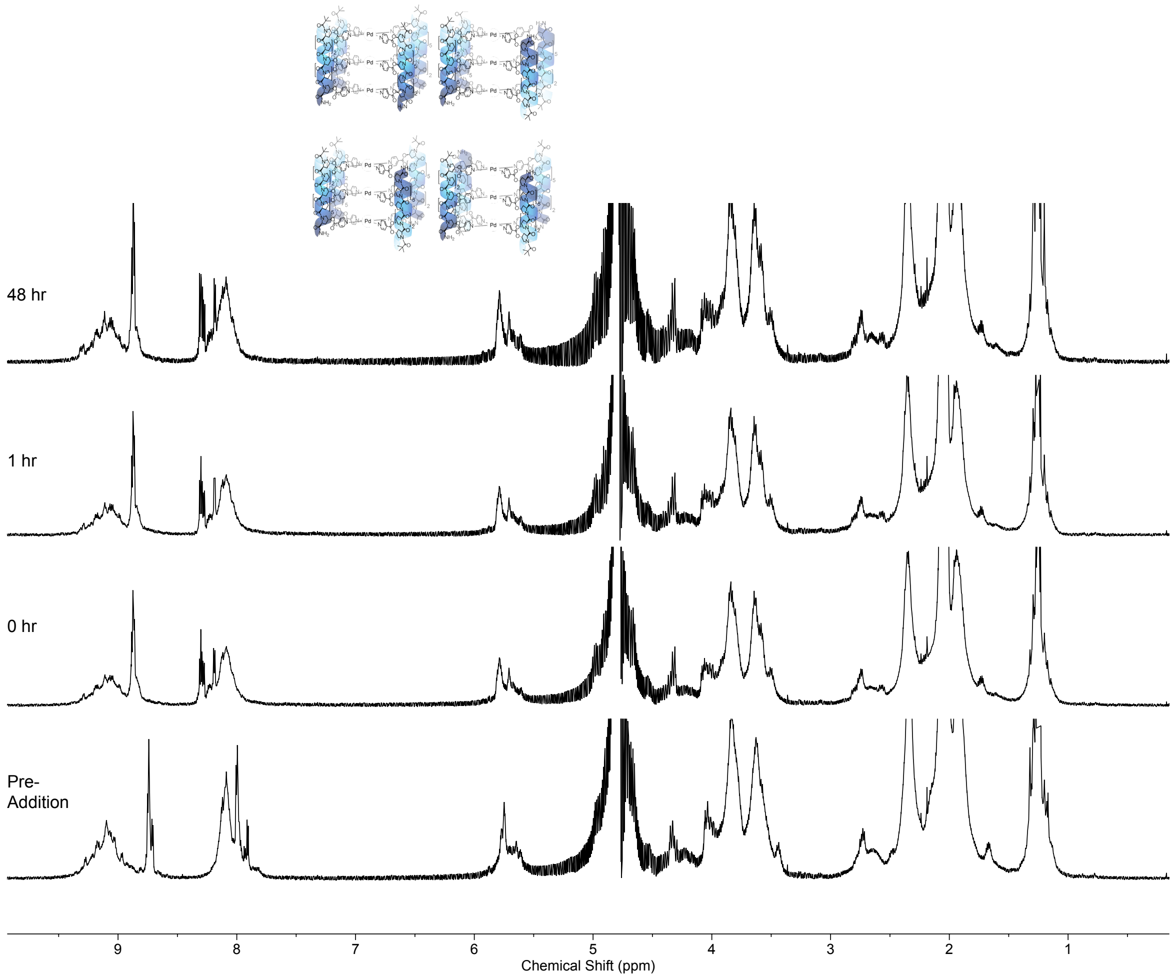

Figure S127. Stacked ¹H NMR (D_2O, 600 MHz, 298 K) of cage **$Pd_3L^{4,R}{}_4$**$(BF_4)_6$ before (bottom) and after addition of 4 eq DCl over time at 298 K.

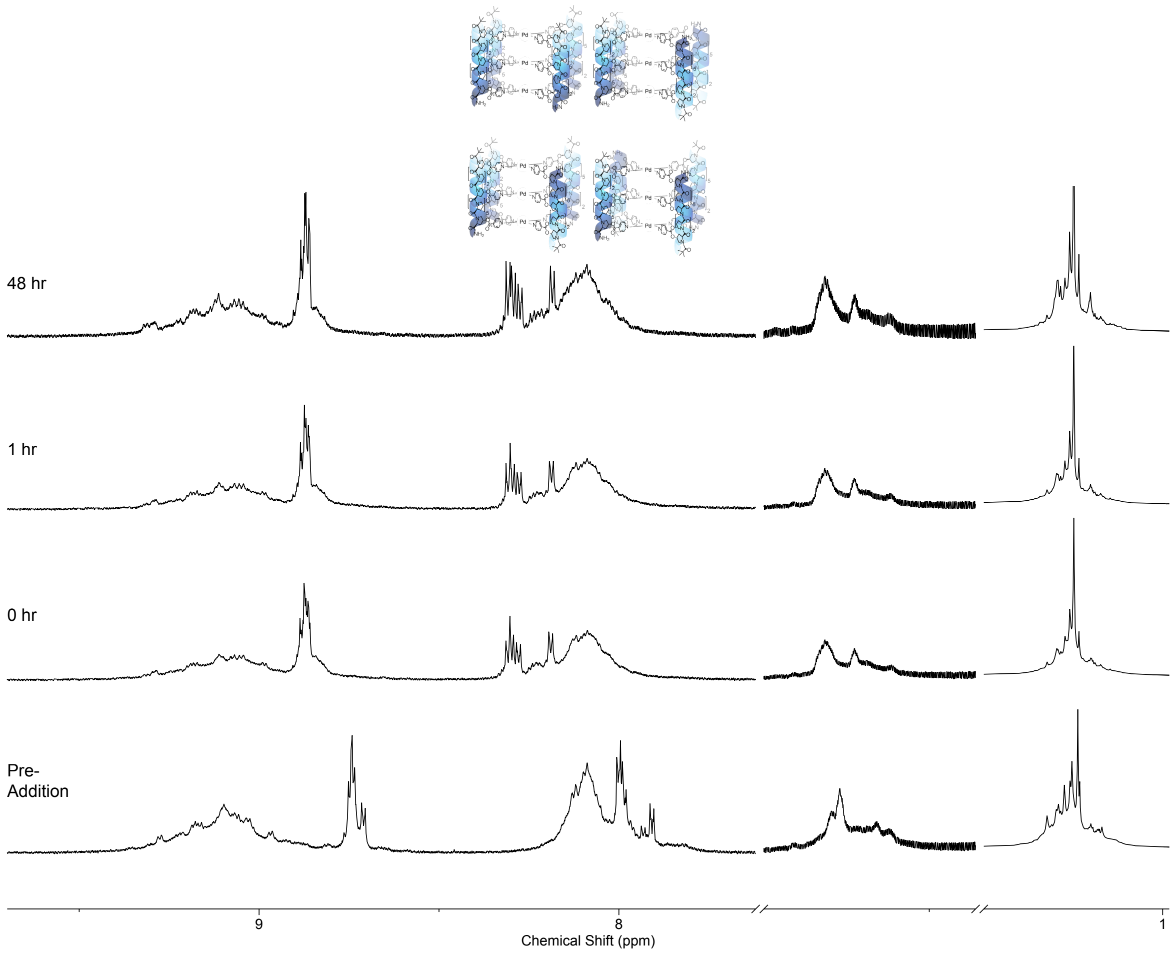

Figure S128. Partial stacked ^1H NMR (D_2O, 600 MHz, 298 K) of cage $\mathbf{Pd_3L^{4,R}_4}(BF_4)_6$ before (bottom) and after addition of 4 eq DCl over time at 298 K. Peaks at ~1 ppm have been scaled independently to rest of spectra due to relative intensity.

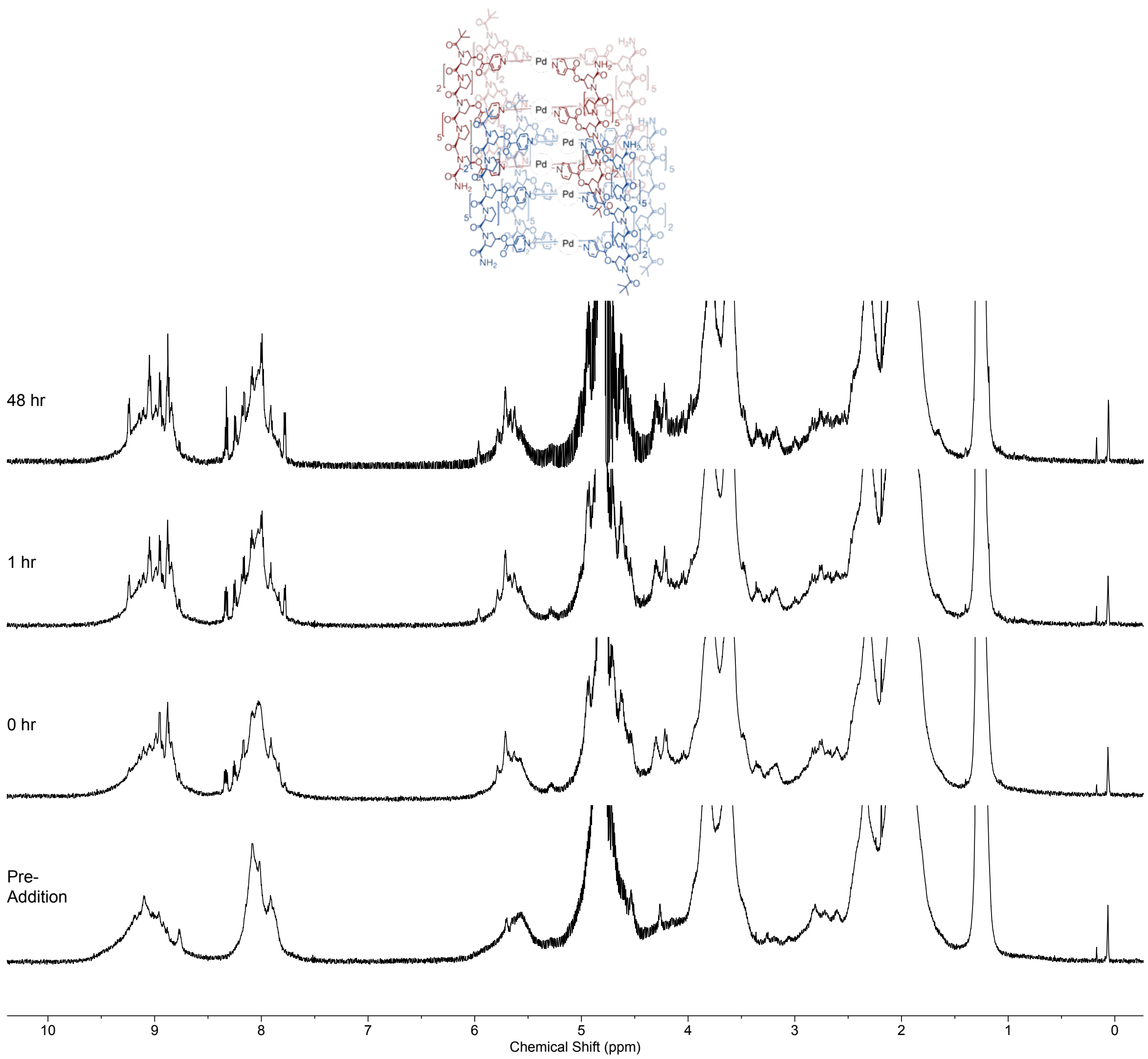

Figure S129. Stacked ^{1}H NMR (D_2O, 600 MHz, 298 K) of cage [$\mathbf{Pd_3L^{7,S}_4}(BF_4)_6$]$_2$ before (bottom) and after addition of 4 eq DCl over time at 298 K.

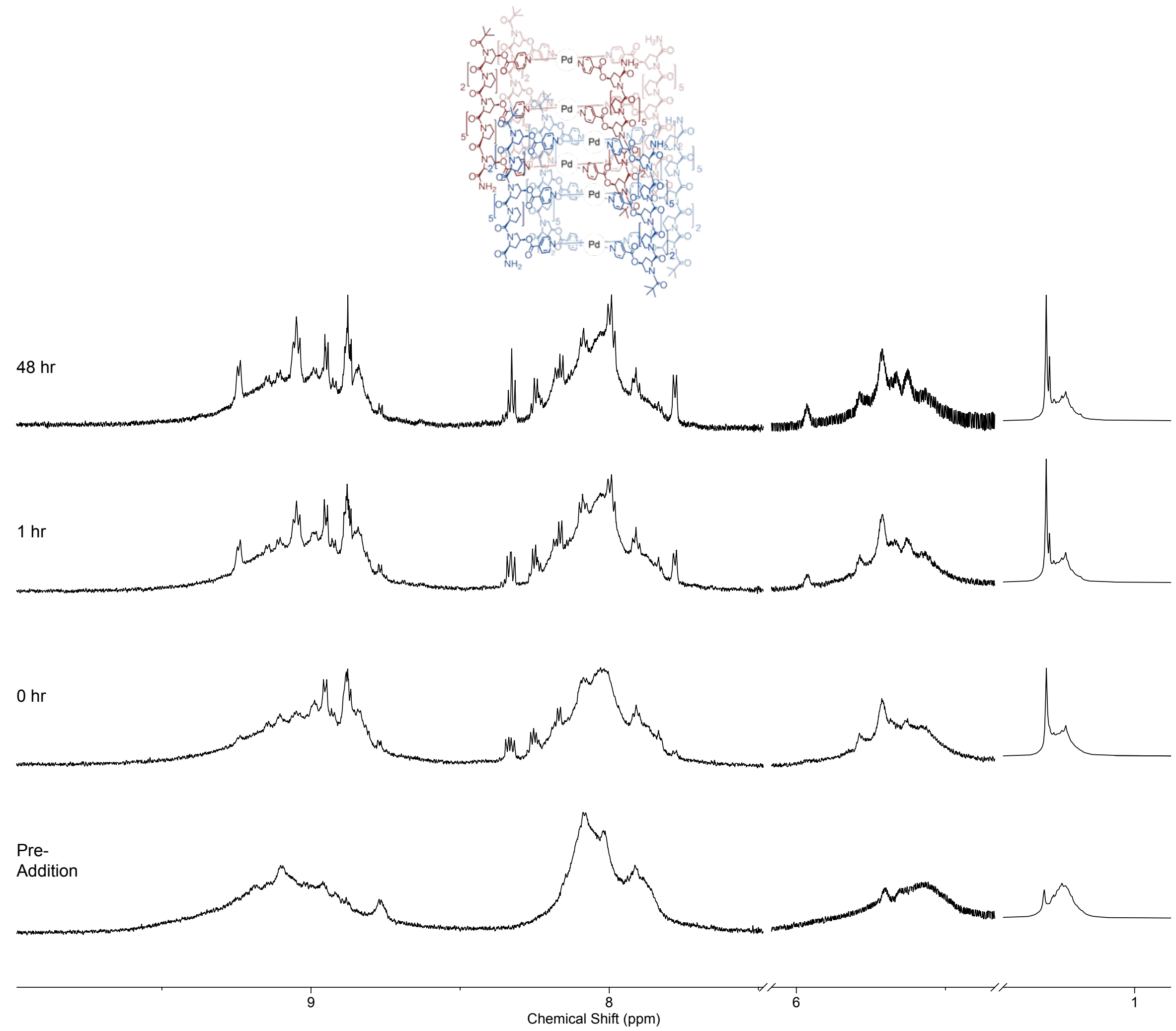

Figure S130. Partial stacked ^{1}H NMR (D_2O, 600 MHz, 298 K) of cage $[\mathbf{Pd_3L^{7,S}{}_4}(BF_4)_6]_2$ before (bottom) and after addition of 4 eq DCl over time at 298 K. Peaks at ~1 ppm have been scaled independently to rest of spectra due to relative intensity.

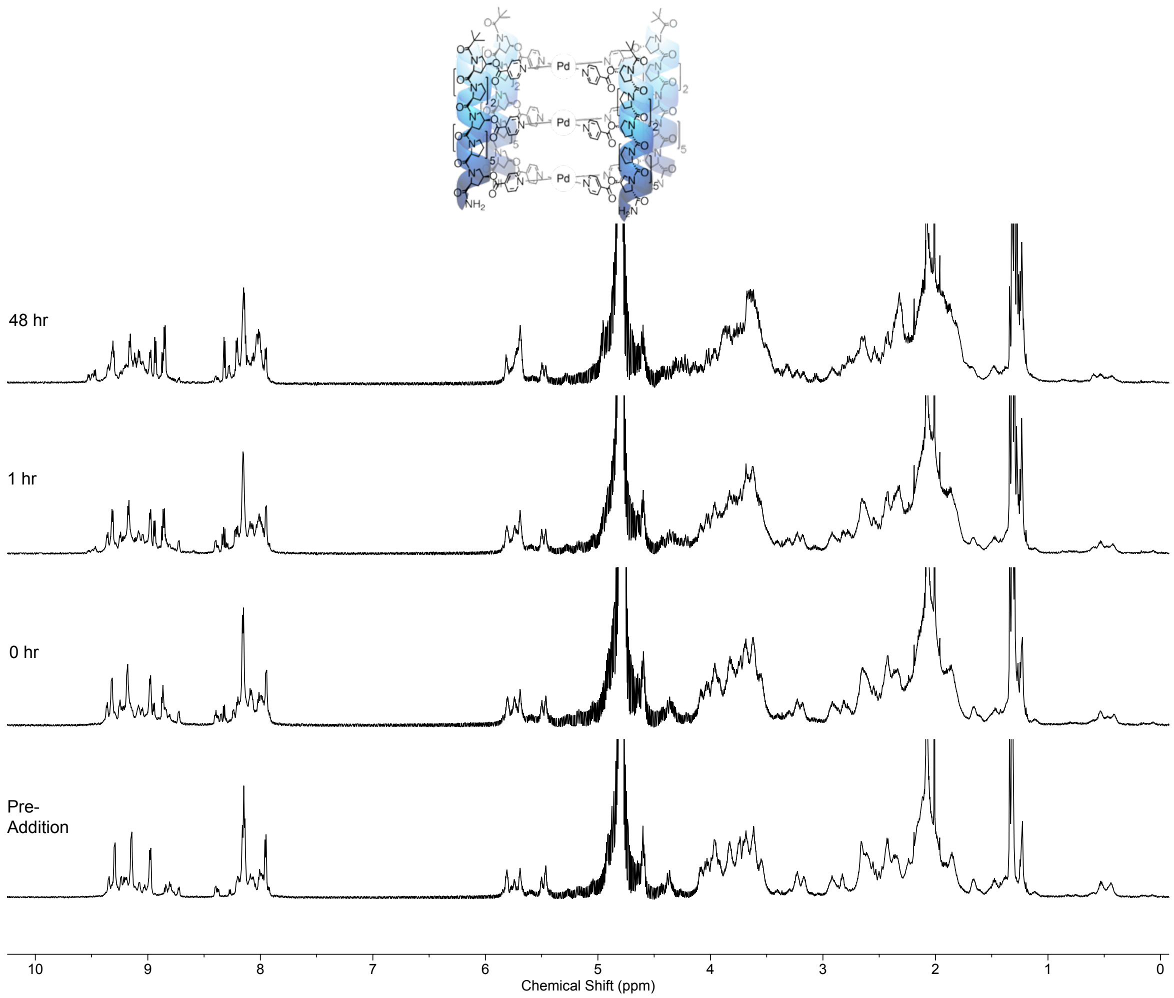

Figure S131. Stacked ^{1}H NMR (D_2O, 600 MHz, 298 K) of cage **$Pd_3L^{4,S}_4$**$(BF_4)_6$ before (bottom) and after addition of 4 eq DCl over time at 298 K.

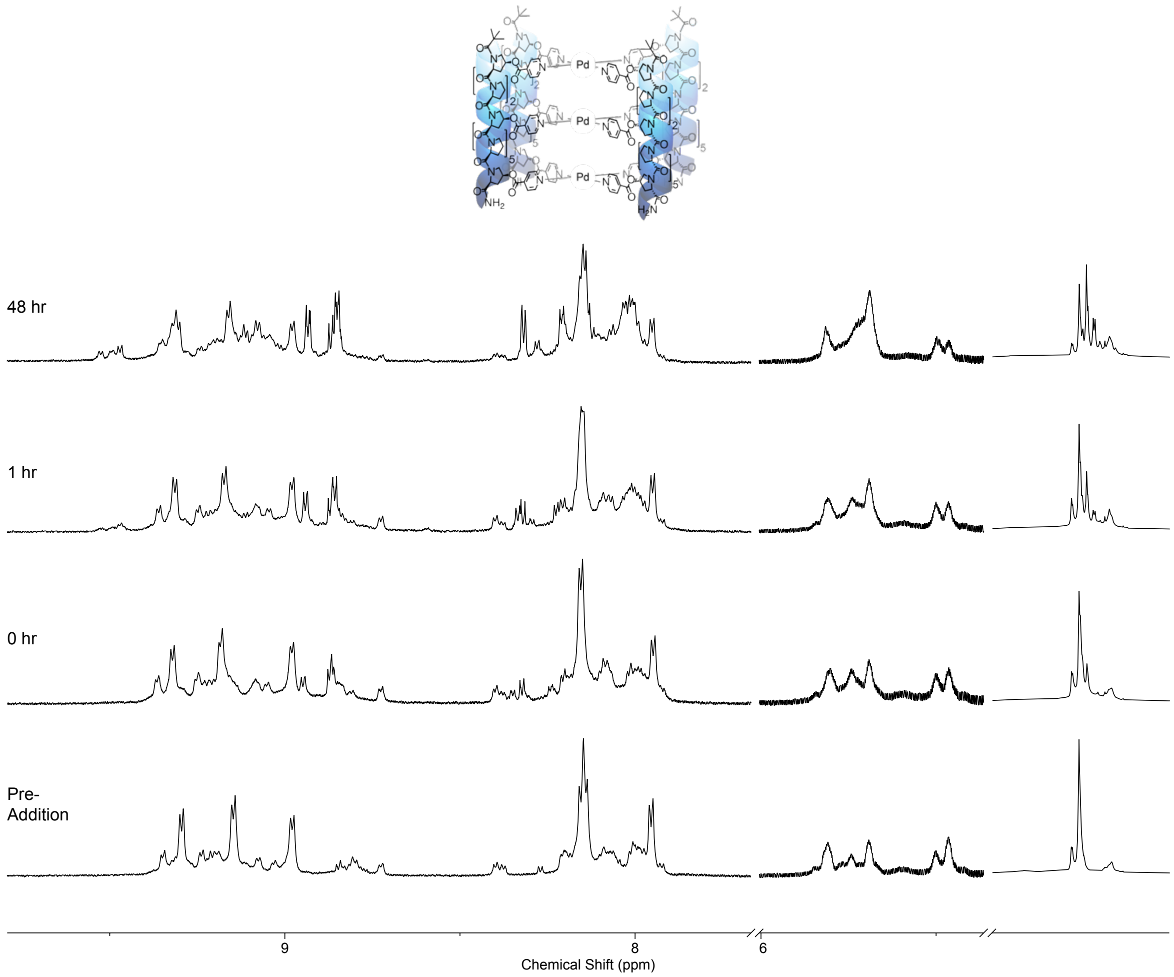

Figure S132. Partial stacked ^1H NMR (D_2O, 600 MHz, 298 K) of cage $\mathbf{Pd_3L^{4,S}_4}(BF_4)_6$ before (bottom) and after addition of 4 eq DCl over time at 298 K. Peaks at ~1 ppm have been scaled independently to rest of spectra due to relative intensity.

Base Stability Studies

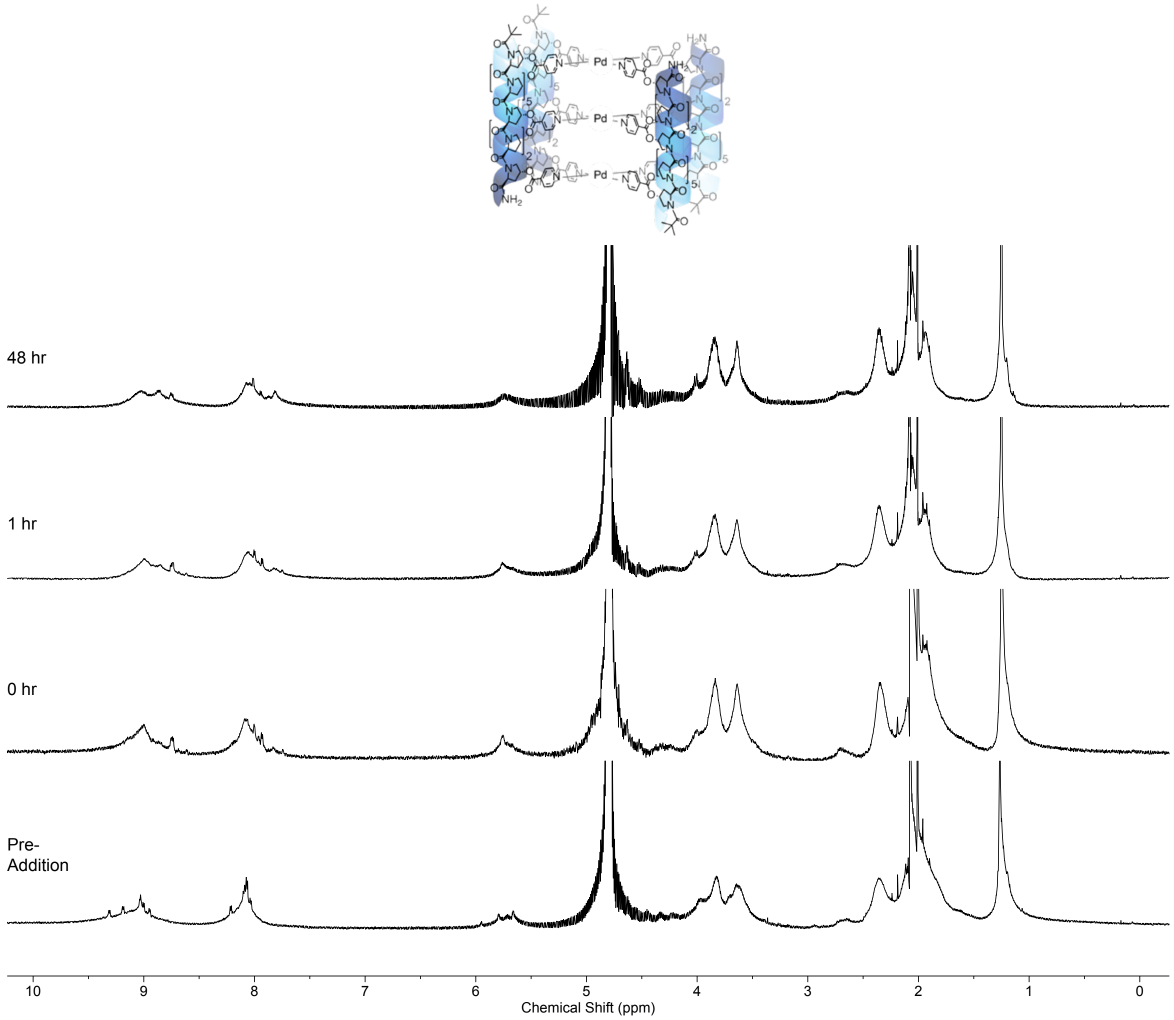

Figure S133. Stacked ^1H NMR (D_2O, 600 MHz, 298 K) of cage $\mathbf{Pd_3L^{7,R}_4}(BF_4)_6$ before (bottom) and after addition of 4 eq NaOD over time at 298 K.

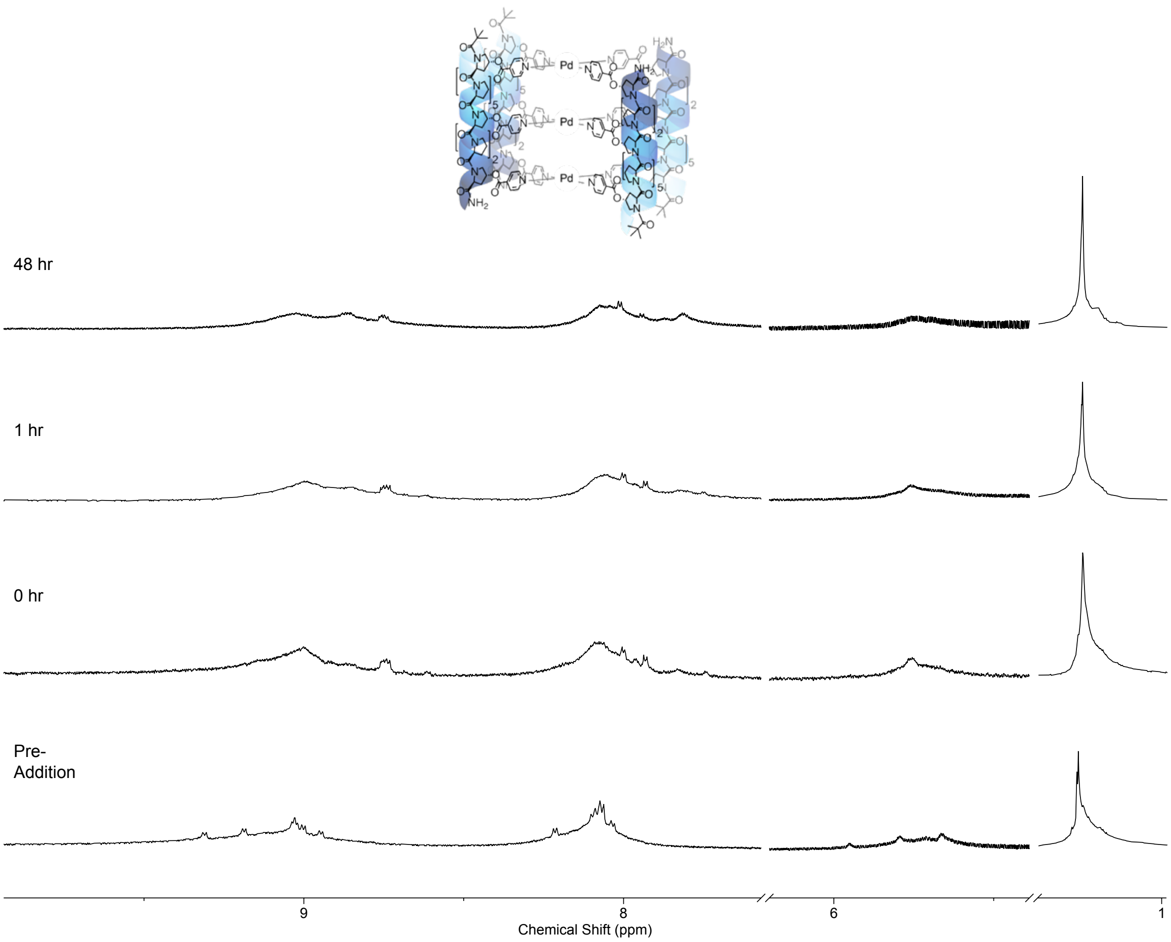

Figure S134. Partial stacked ^{1}H NMR (D_2O, 600 MHz, 298 K) of cage $\mathbf{Pd_3L^{7,\mathit{R}}_4}(BF_4)_6$ before (bottom) and after addition of 4 eq NaOD over time at 298 K. Peaks at ~1 ppm have been scaled independently to rest of spectra due to relative intensity.

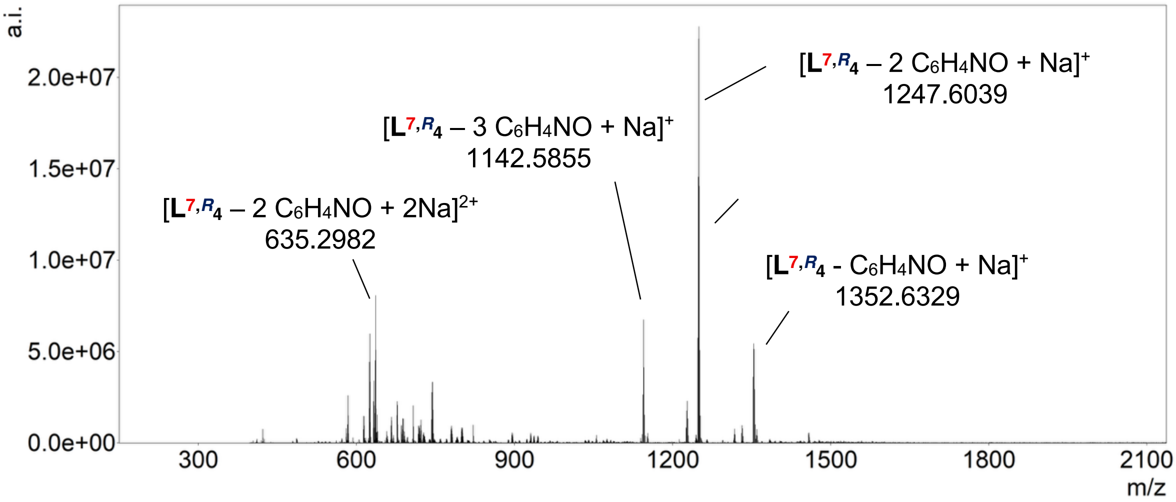

Figure S135. ESI-MS (+ve ion, H_2O/D_2O) of cage $\mathbf{Pd_3L^{7,\mathit{R}}_4}(BF_4)_6$ after addition of 4 eq. NaOD after 48 hrs at 298 K.

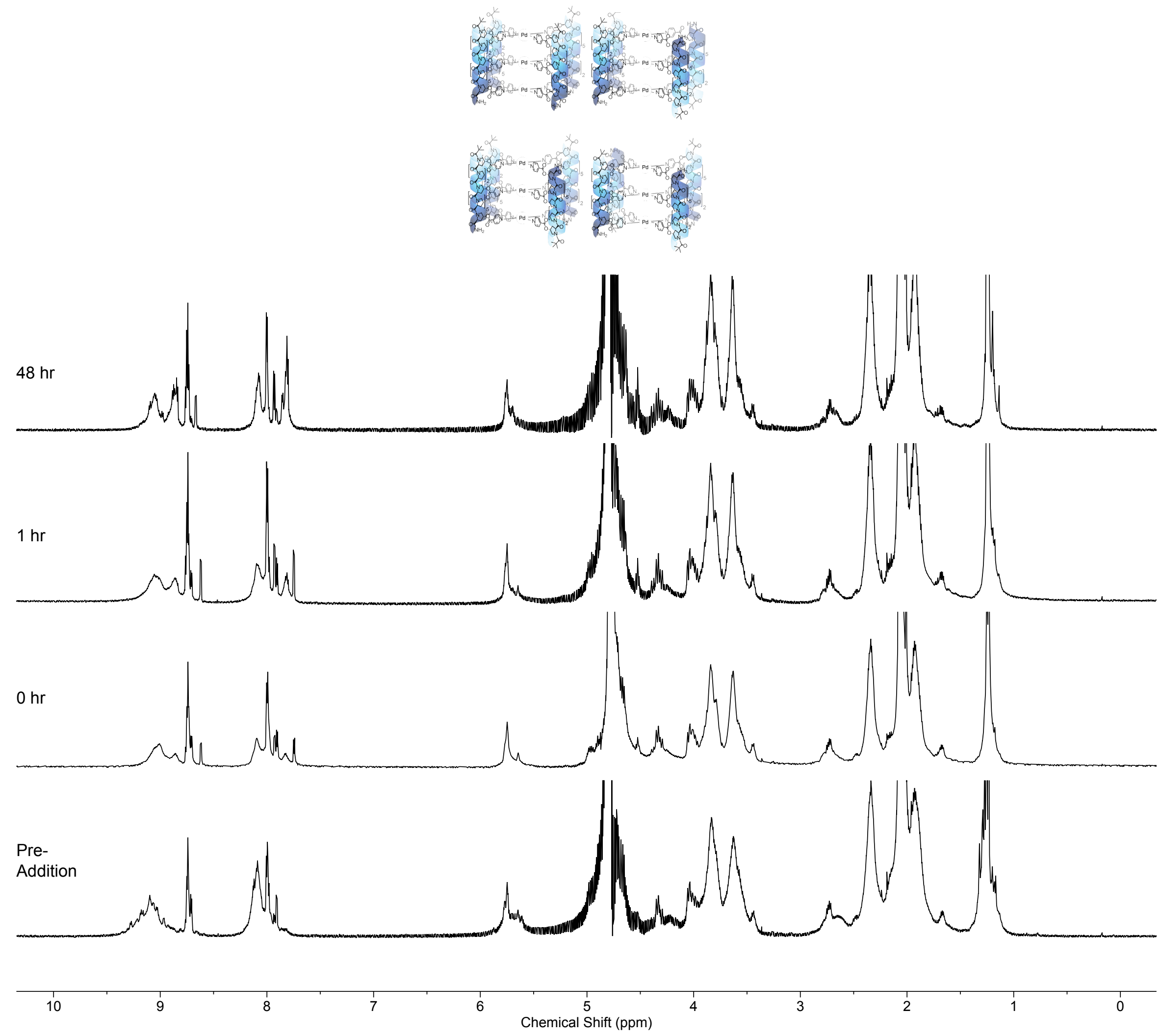

Figure S136. Stacked ^{1}H NMR (D_2O, 600 MHz, 298 K) of cage $\mathbf{Pd_3L^{4,\mathit{R}}_4}(BF_4)_6$ before (bottom) and after addition of 4 eq NaOD over time at 298 K. Peaks at ~1 ppm have been scaled independently to rest of spectra due to relative intensity.

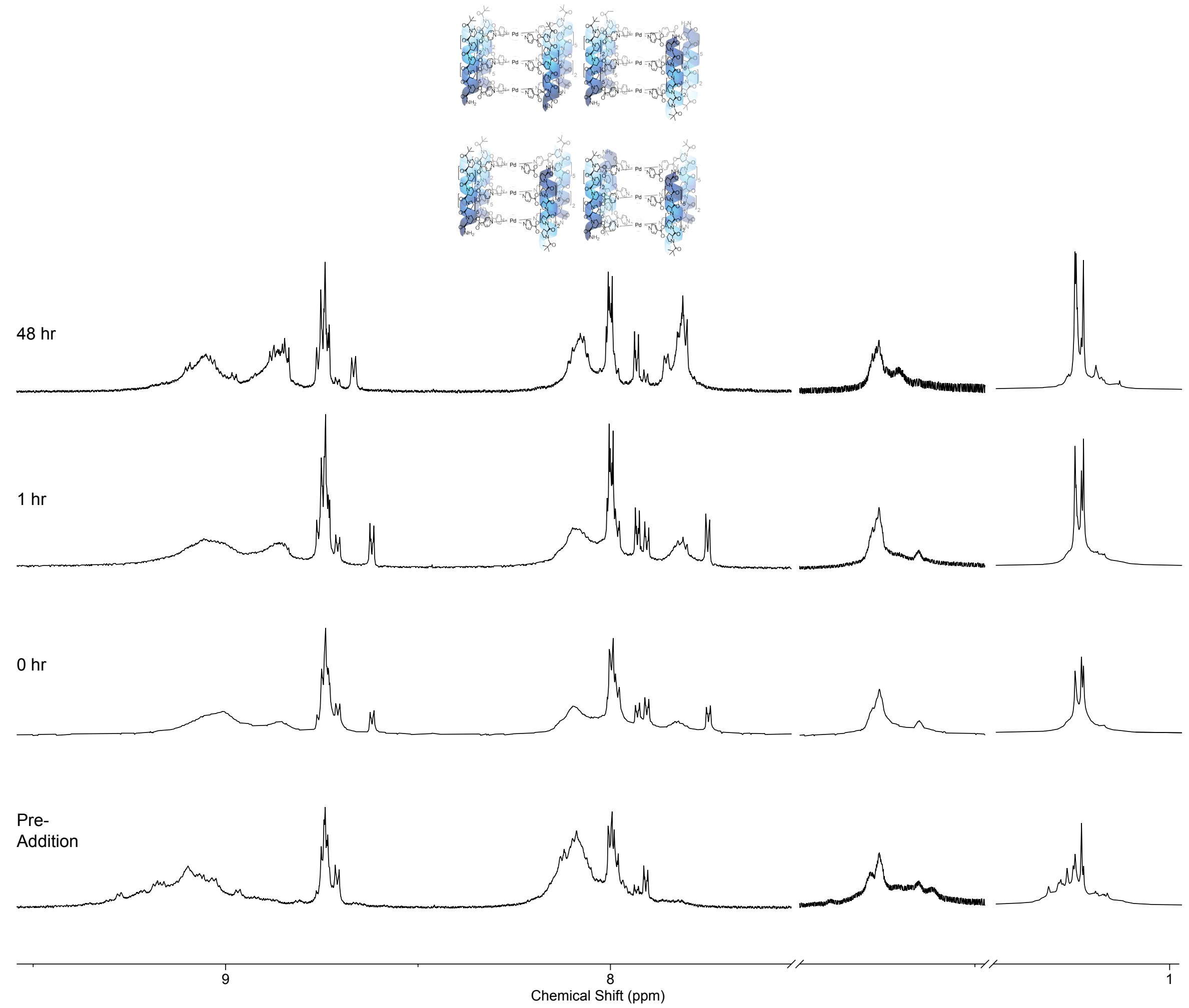

Figure S137. Partial stacked ^{1}H NMR (D_2O, 600 MHz, 298 K) of cage $\mathbf{Pd_3L^{4,R}_4}(BF_4)_6$ before (bottom) and after addition of 4 eq NaOD over time at 298 K. Peaks at ~1 ppm have been scaled independently to rest of spectra due to relative intensity.

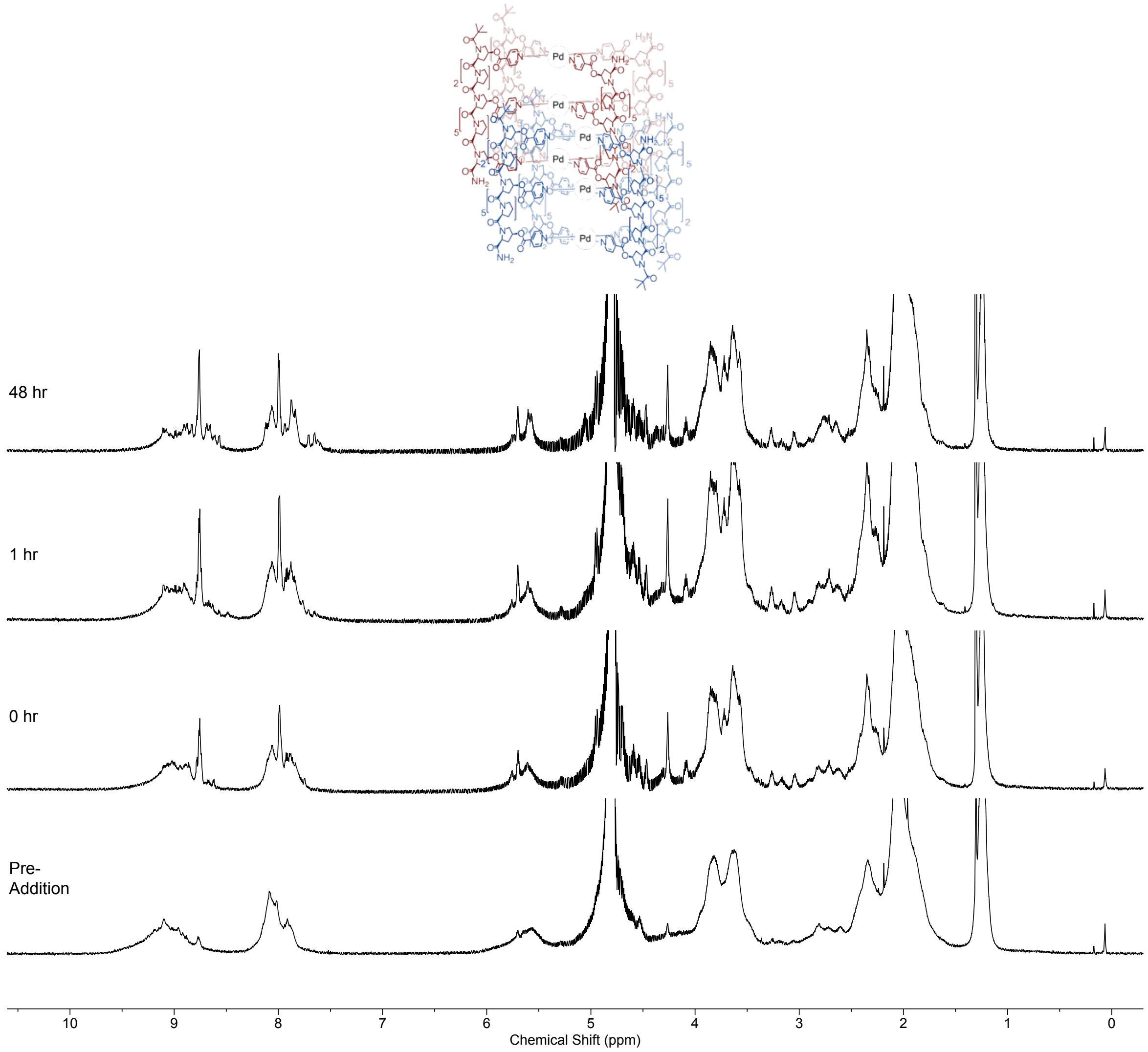

Figure S138. Stacked ¹H NMR (D_2O, 600 MHz, 298 K) of cage [$\mathbf{Pd_3L^{7,S}_4}$$(BF_4)_6$]$_2$ before (bottom) and after addition of 4 eq NaOD over time at 298 K.

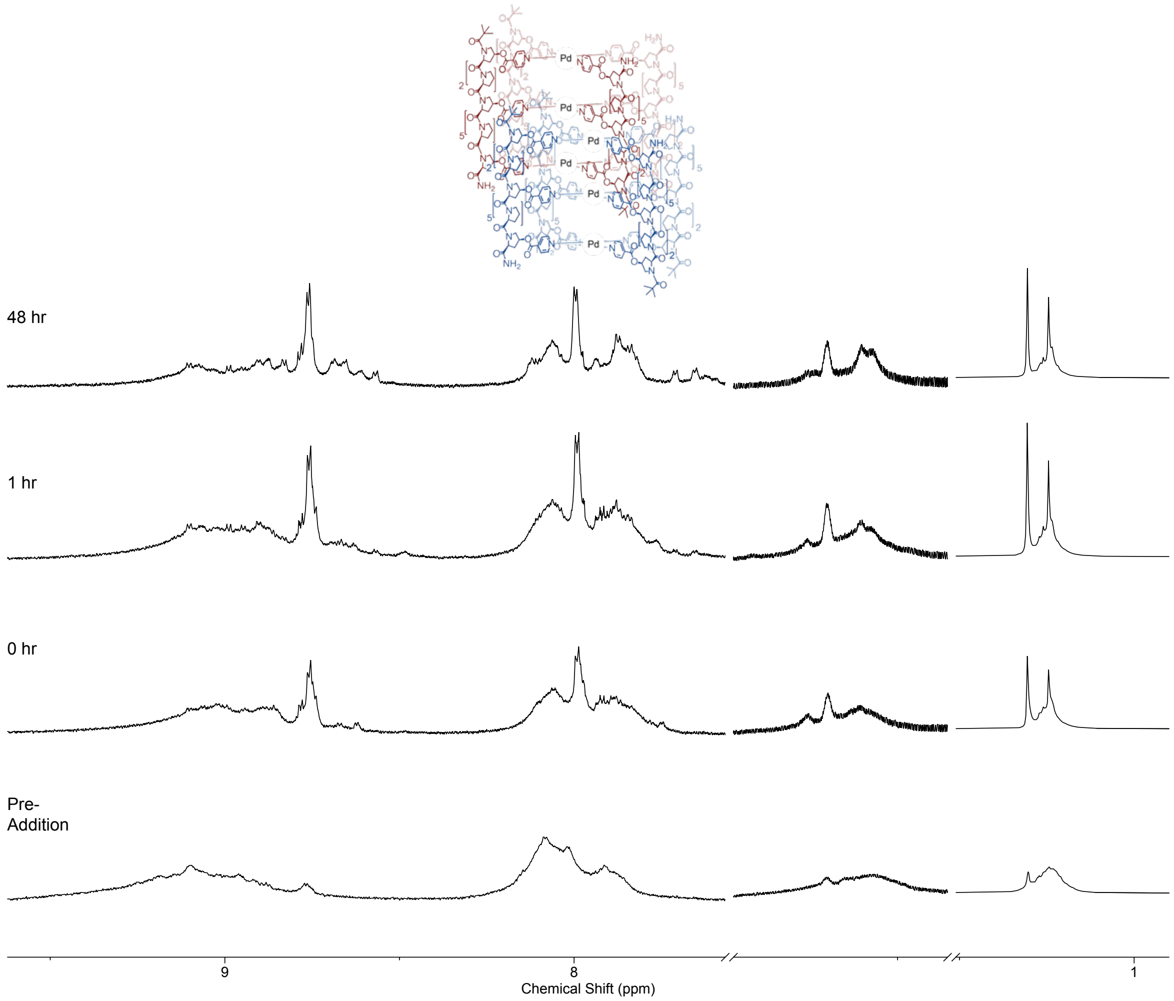

Figure S139. Partial stacked ^{1}H NMR (D_2O, 600 MHz, 298 K) of cage [**$Pd_3L^{7,S}{}_4$**$(BF_4)_6$]$_2$ before (bottom) and after addition of 4 eq NaOD over time at 298 K. Peaks at ~1 ppm have been scaled independently to rest of spectra due to relative intensity.

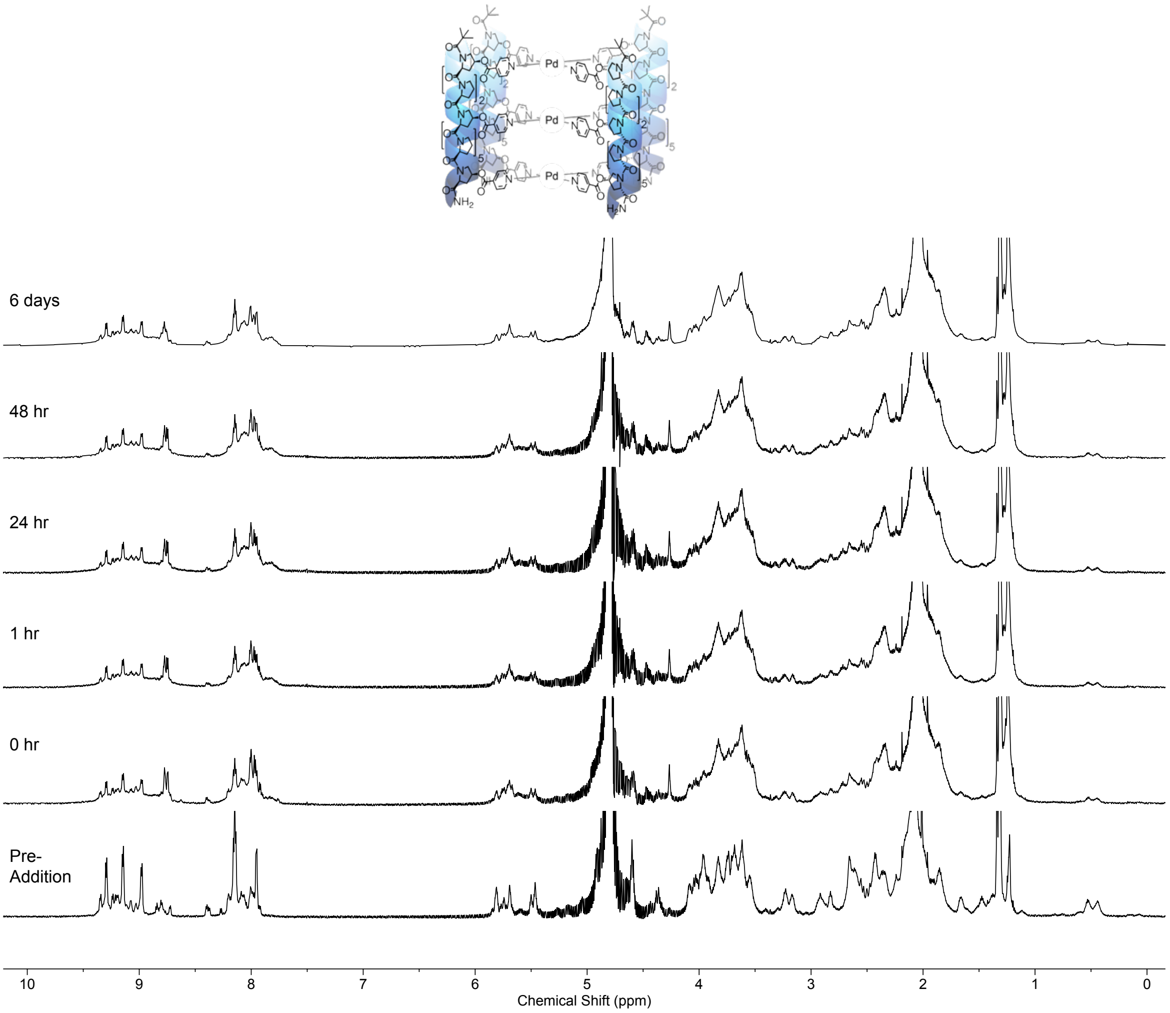

Figure S140 Stacked ^{1}H NMR (D_2O, 600 MHz, 298 K) of cage **$Pd_3L^{4,S}_4$**$(BF_4)_6$ before (bottom) and after addition of 4 eq NaOD over time at 298 K.

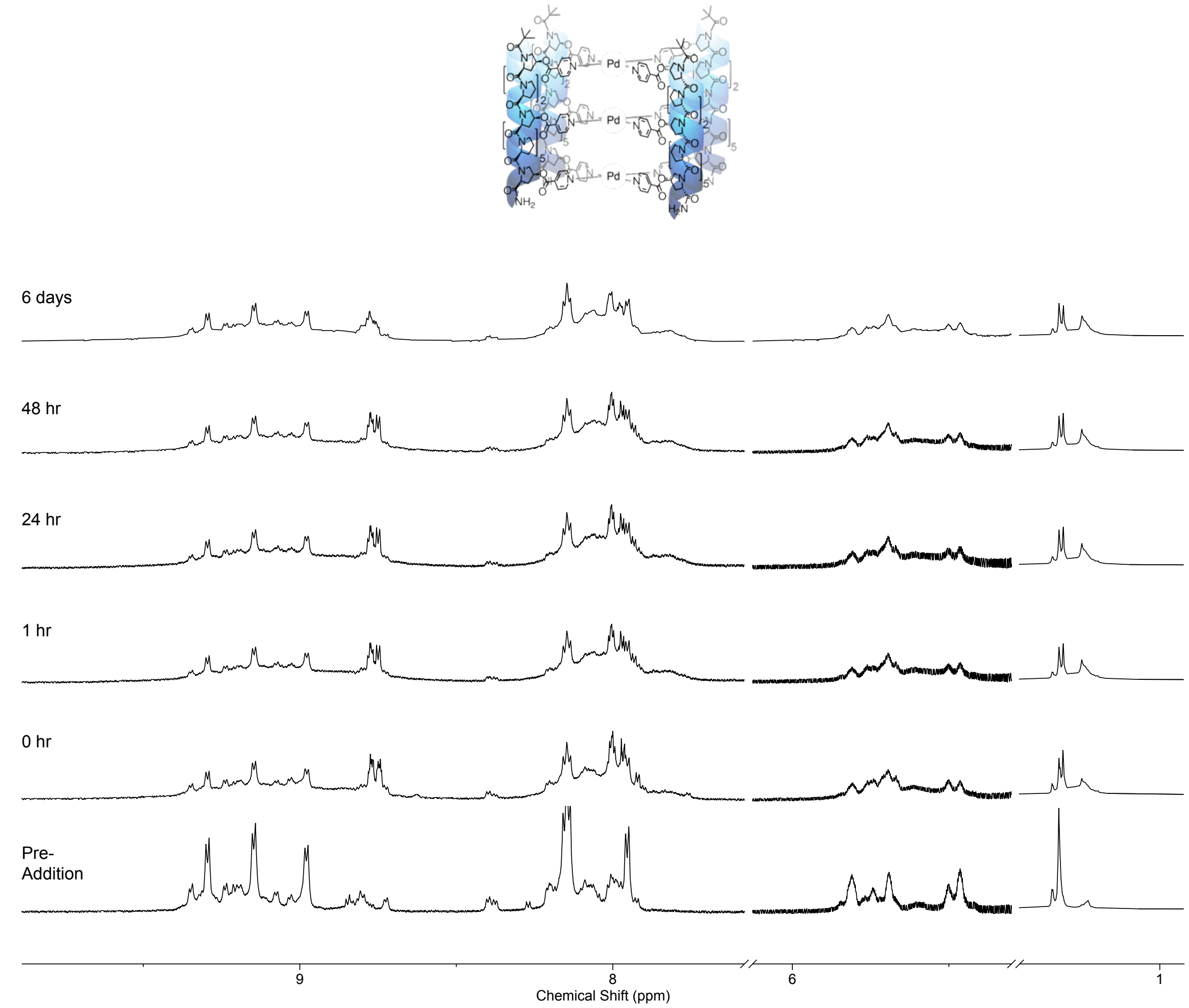

Figure S141. Partial stacked ^{1}H NMR (D_2O, 600 MHz, 298 K) of cage **Pd$_3$L^{4,S}$_4$**$(BF_4)_6$ before (bottom) and after addition of 4 eq NaOD over time at 298 K. Peaks at ~1 ppm have been scaled independently to rest of spectra due to relative intensity.

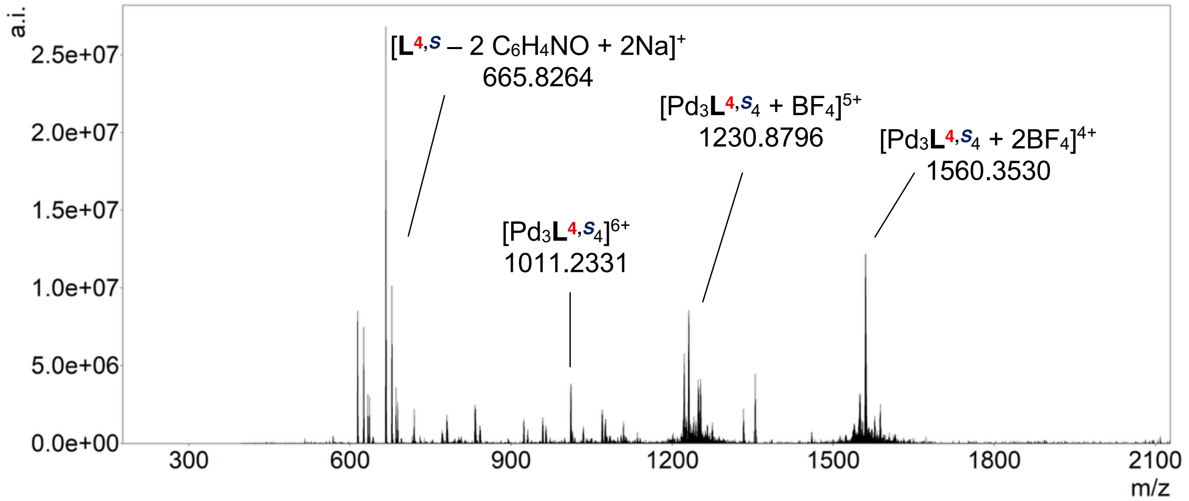

Figure S142. ESI-MS (+ve ion, H_2O/D_2O) of cage **Pd$_3$L^{4,S}$_4$**$(BF_4)_6$ after addition of 4 eq. NaOD after 48 hrs at 298 K.

Pyridine Stability Studies

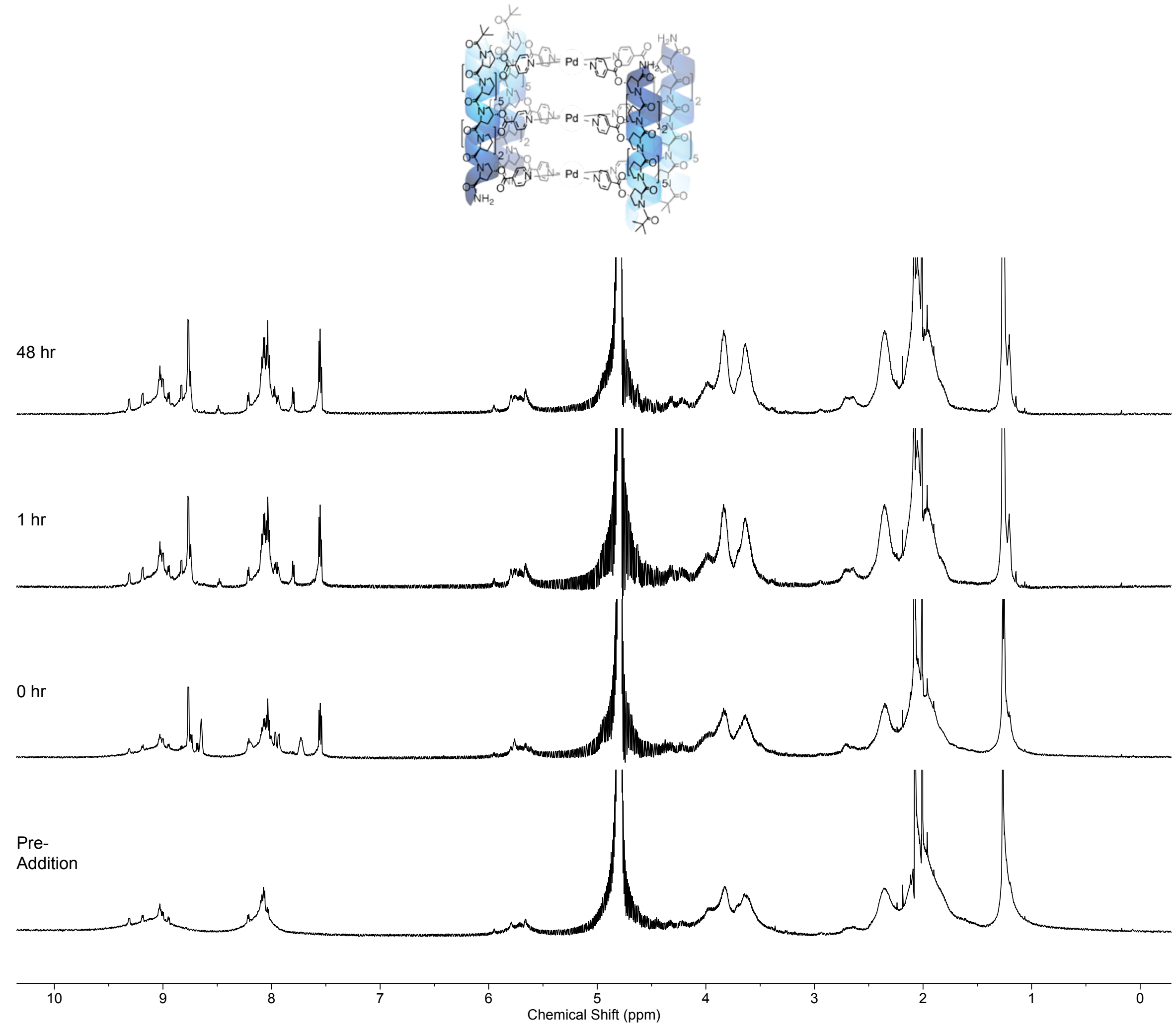

Figure S143. Stacked ^{1}H NMR (D_2O, 600 MHz, 298 K) of cage **$Pd_3L^{7,R}_4$**$(BF_4)_6$ before (bottom) and after addition of 4 eq pyridine over time at 298 K.

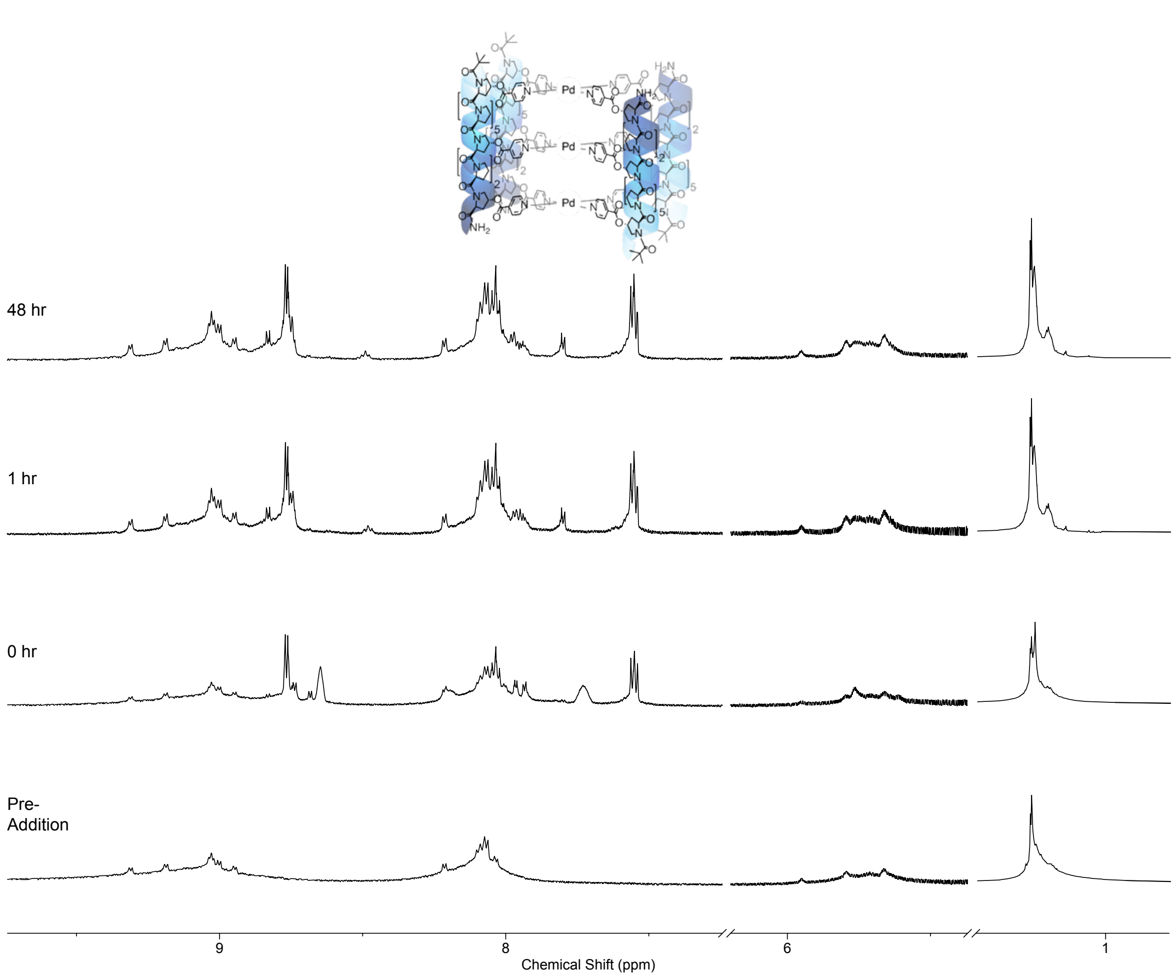

Figure S144. Partial stacked ^{1}H NMR (D_2O, 600 MHz, 298 K) of cage **$Pd_3L^{7,R}_4(BF_4)_6$** before (bottom) and after addition of 4 eq pyridine over time at 298 K. Peaks at ~1 ppm have been scaled independently to rest of spectra due to relative intensity.

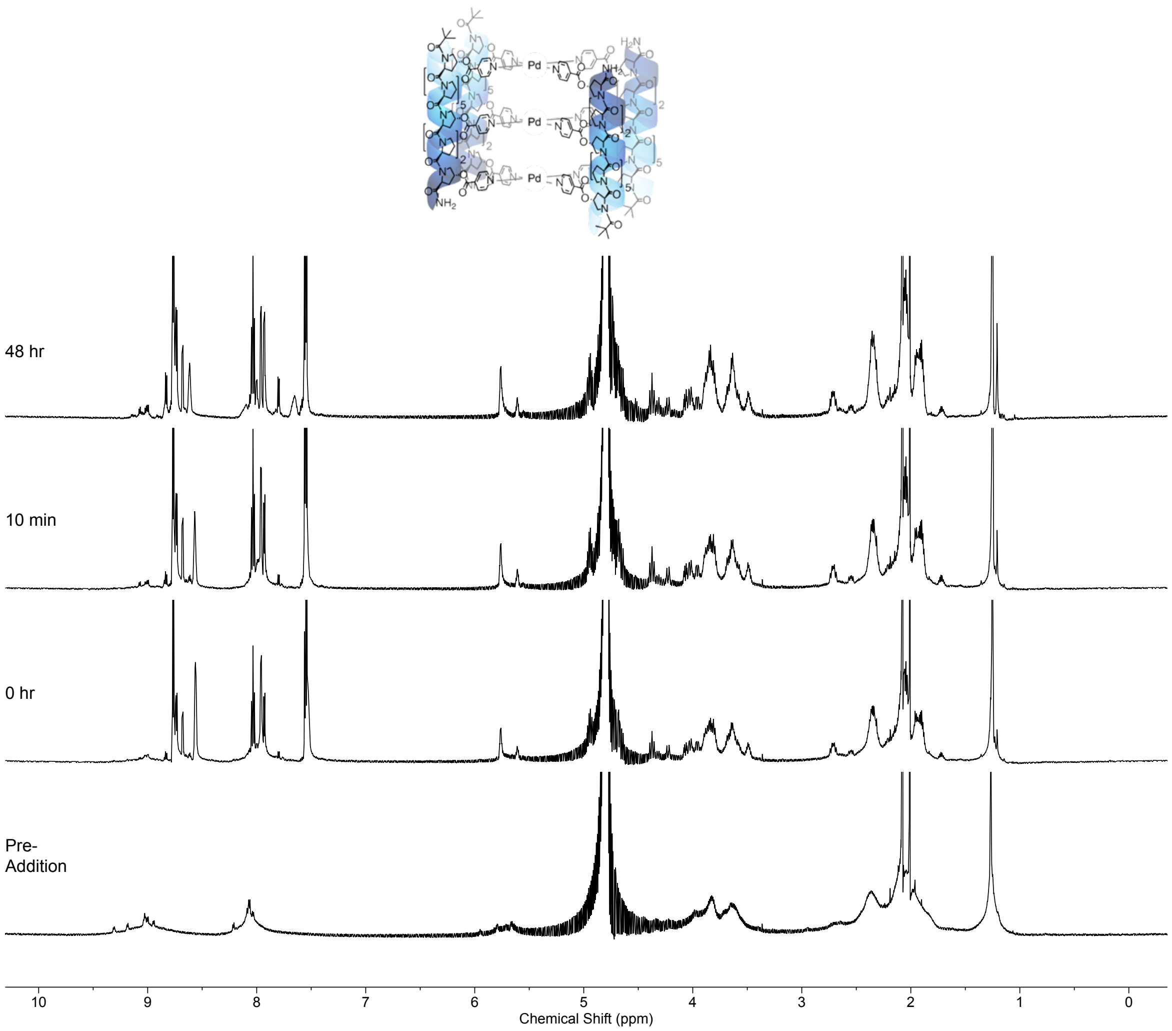

Figure S145. Stacked ^{1}H NMR (D_2O, 600 MHz, 298 K) of cage $\mathbf{Pd_3L^{7,R}_4}(BF_4)_6$ before (bottom) and after addition of 12 eq pyridine over time at 298 K.

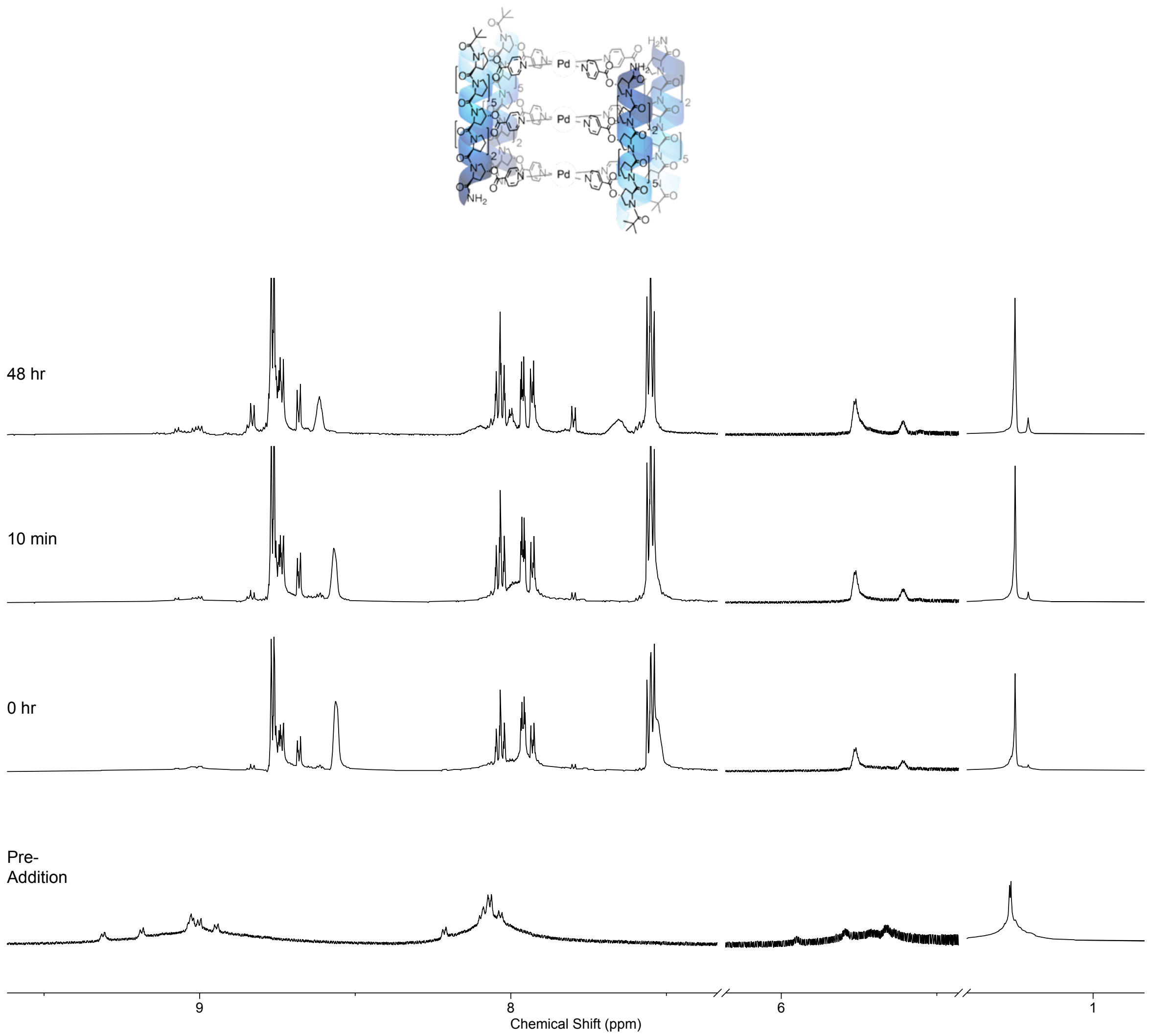

Figure S146. Partial stacked ^{1}H NMR (D_2O, 600 MHz, 298 K) of cage $\mathbf{Pd_3L^{7,R}_4}(BF_4)_6$ before (bottom) and after addition of 12 eq pyridine over time at 298 K. Peaks at ~1 ppm have been scaled independently to rest of spectra due to relative intensity.

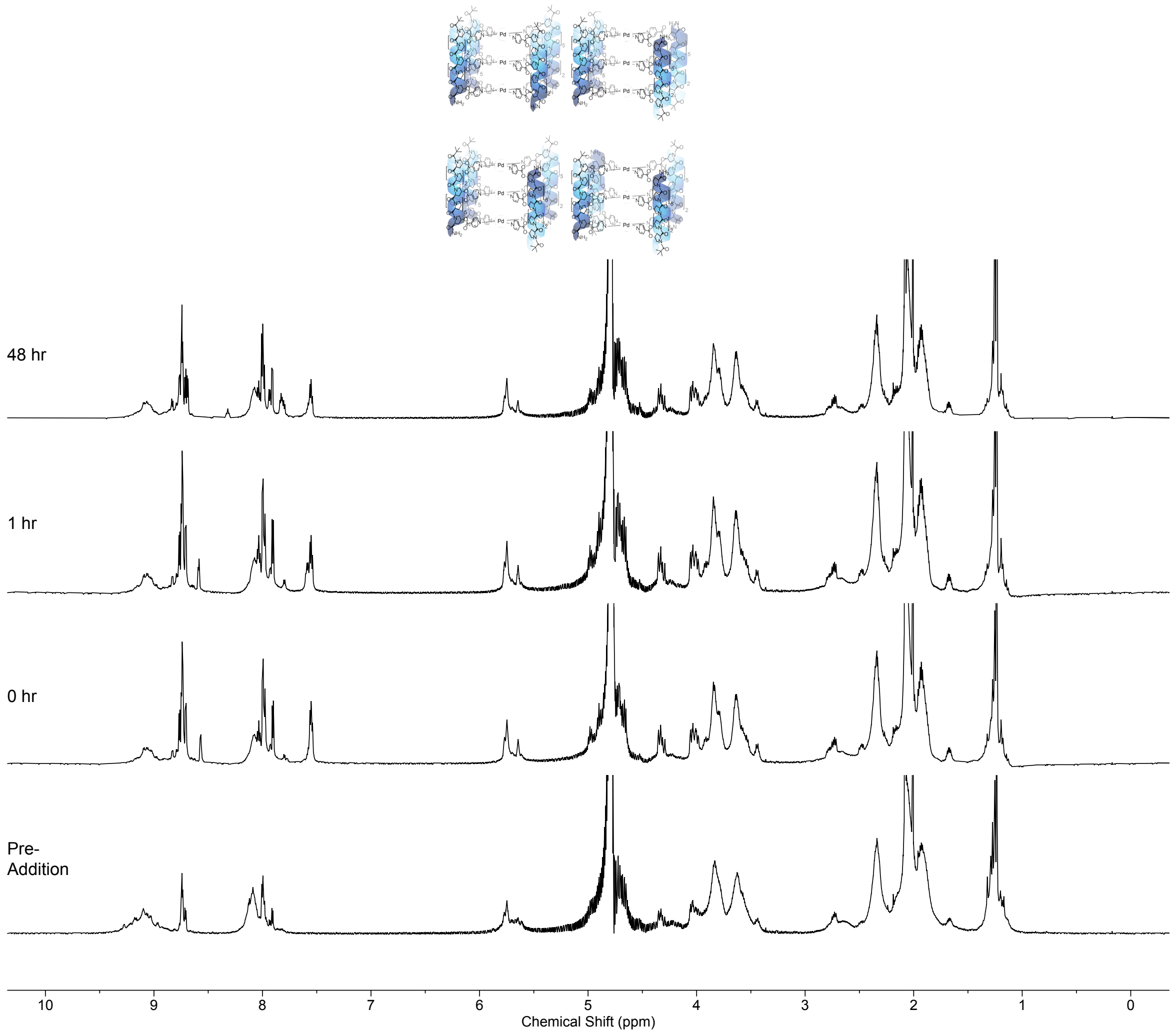

Figure S147. Stacked ^{1}H NMR (D_2O, 600 MHz, 298 K) of cage $\mathbf{Pd_3L^{4,R}{}_4}(BF_4)_6$ before (bottom) and after addition of 4 eq pyridine over time at 298 K.

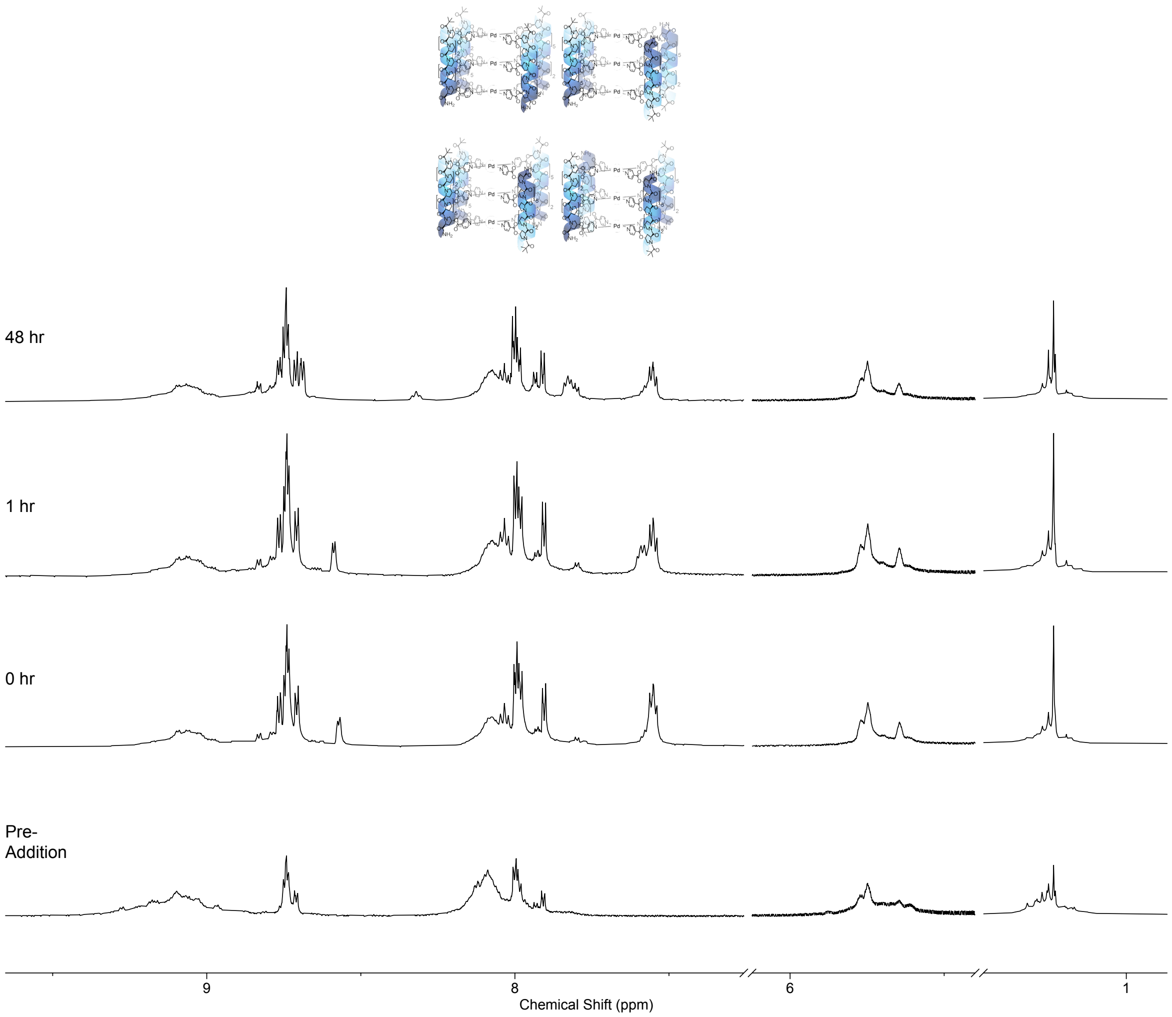

Figure S148. Partial stacked ^{1}H NMR (D_2O, 600 MHz, 298 K) of cage $\mathbf{Pd_3L^{4,\mathit{R}}_4}(BF_4)_6$ before (bottom) and after addition of 4 eq pyridine over time at 298 K. Peaks at ~1 ppm have been scaled independently to rest of spectra due to relative intensity.

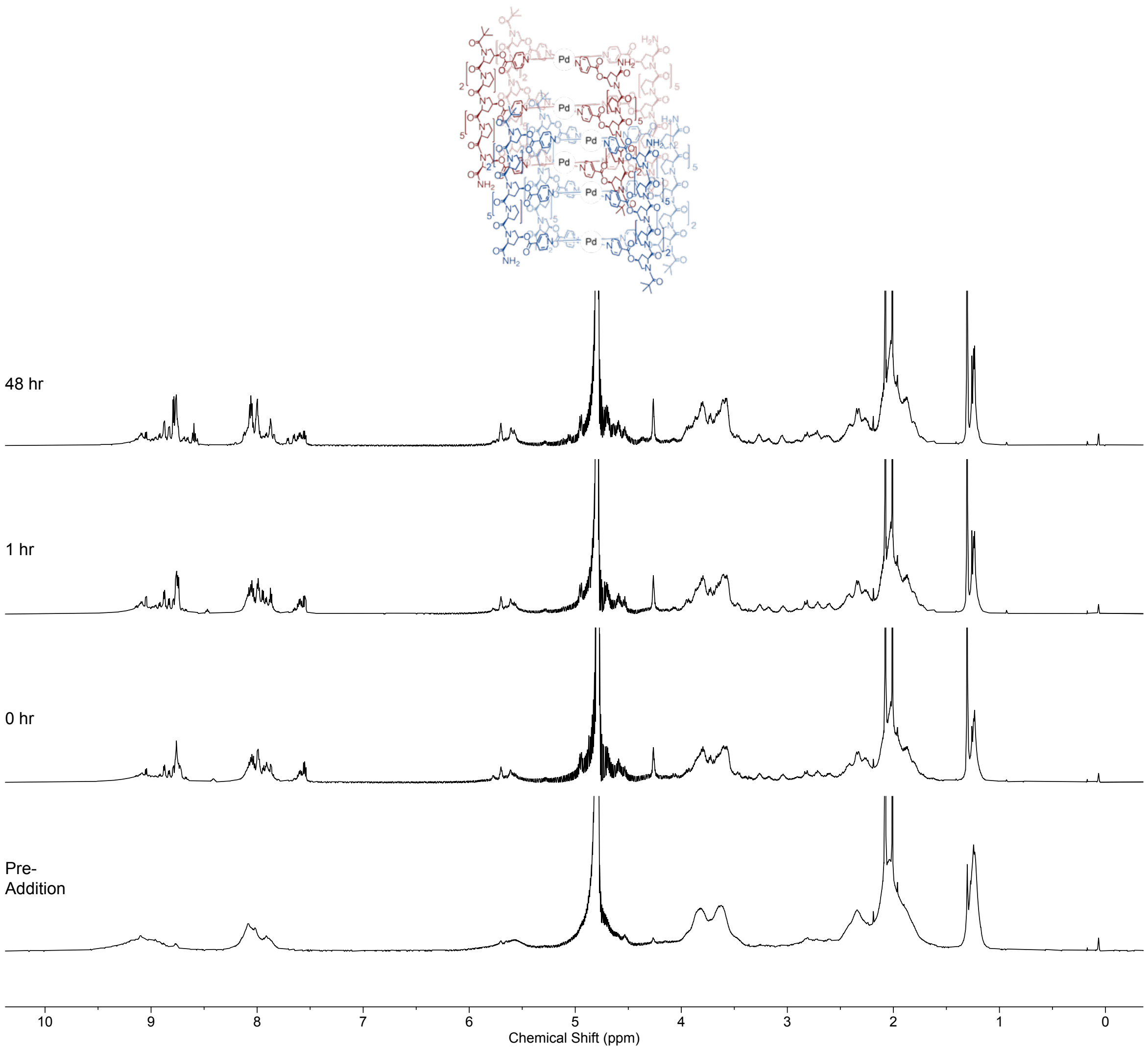

Figure S149. Stacked ^{1}H NMR (D_2O, 600 MHz, 298 K) of cage **$[Pd_3L^{7,\mathit{S}}{}_4(BF_4)_6]_2$** before (bottom) and after addition of 4 eq pyridine over time at 298 K.

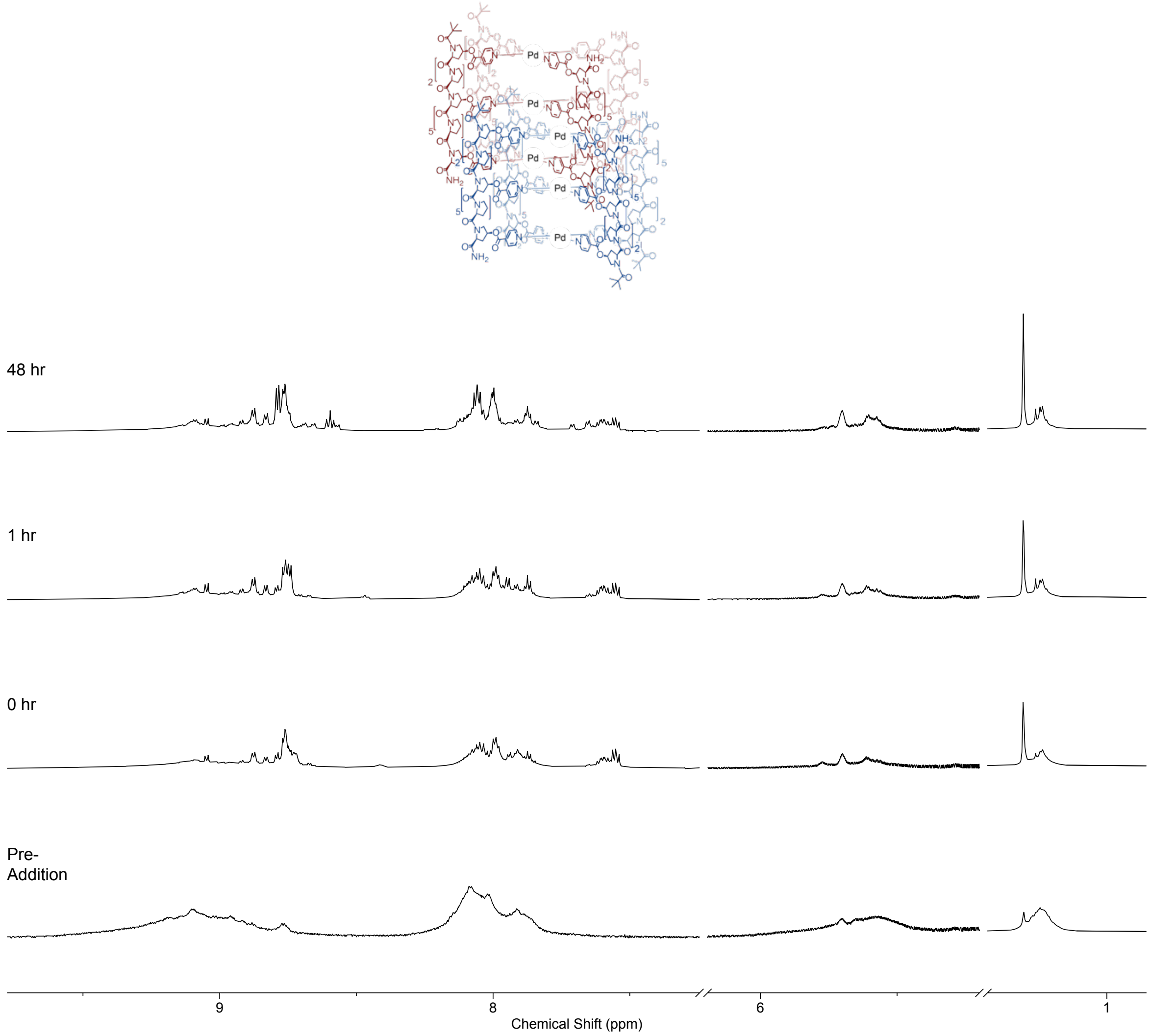

Figure S150. Partial stacked ^{1}H NMR (D_2O, 600 MHz, 298 K) of cage [$\mathbf{Pd_3L^{7,\mathit{S}}_4}$$(BF_4)_6]_2$ before (bottom) and after addition of 4 eq pyridine over time at 298 K. Peaks at ~1 ppm have been scaled independently to rest of spectra due to relative intensity.

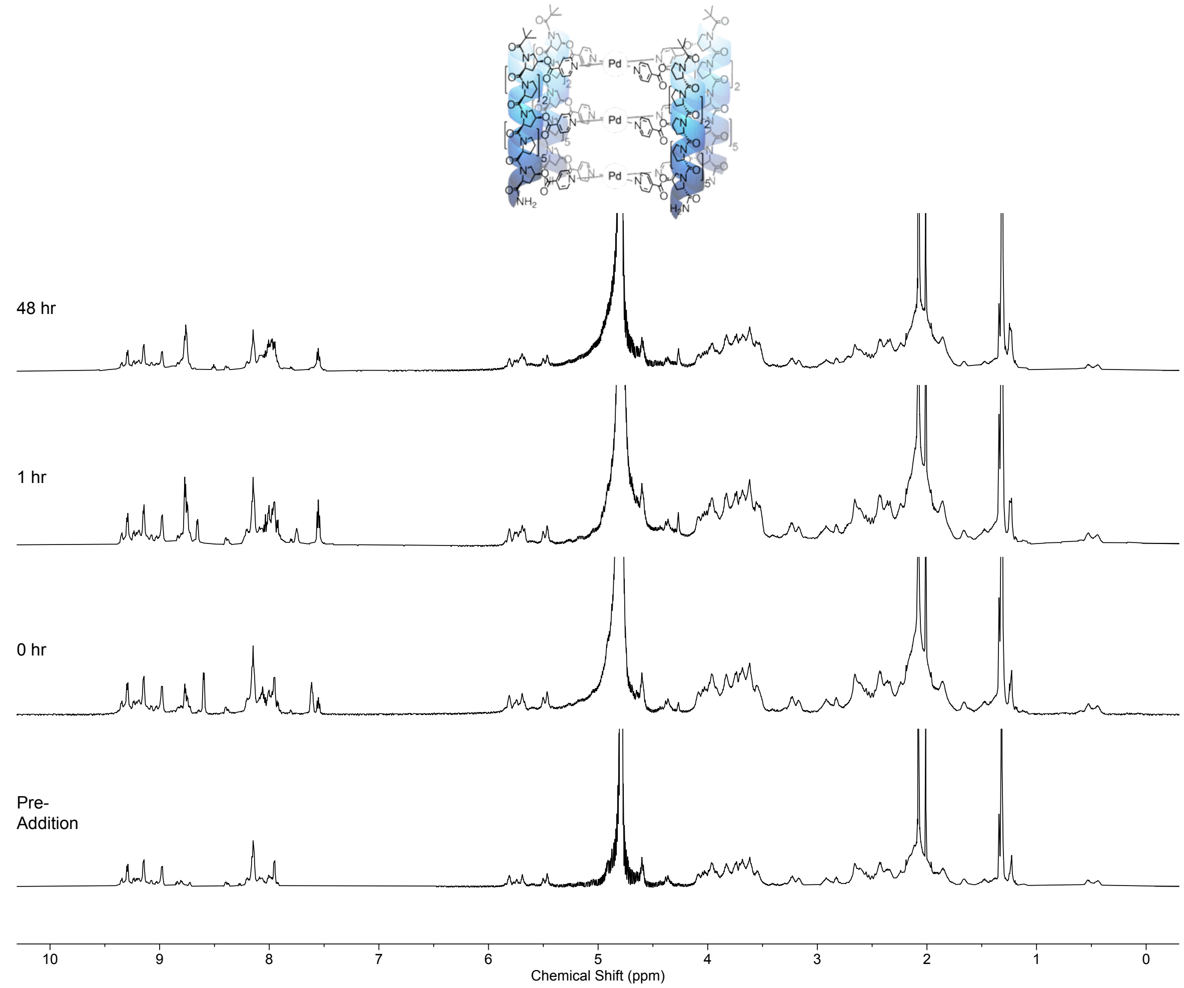

Figure S151. Stacked ^{1}H NMR (D_2O, 600 MHz, 298 K) of cage $\mathbf{Pd_3L^{4,\mathit{S}}_4}(BF_4)_6$ before (bottom) and after addition of 4 eq pyridine over time at 298 K.

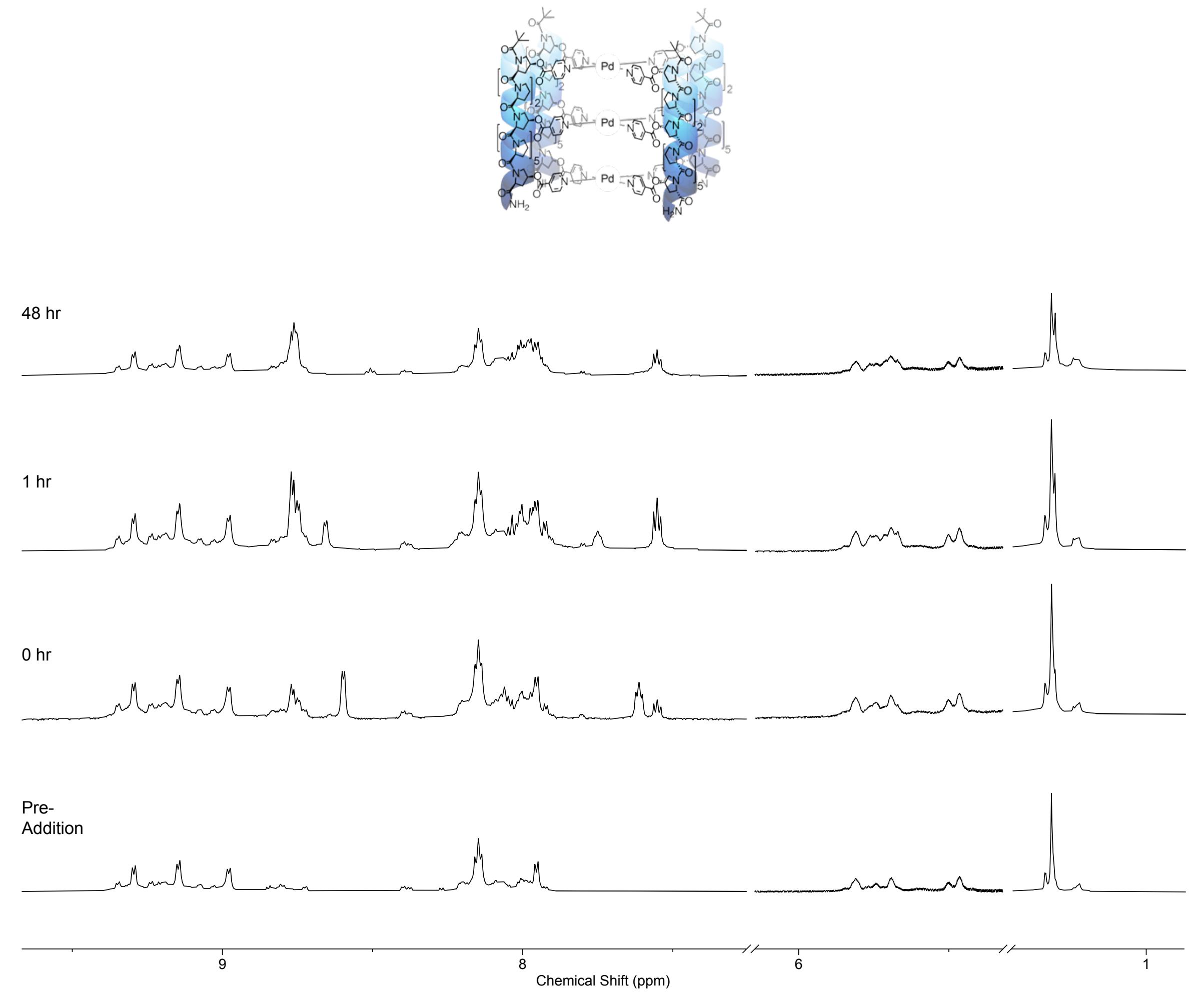

Figure S152. Partial stacked ^{1}H NMR (D_2O, 600 MHz, 298 K) of cage $\mathbf{Pd_3L^{4,S}_4}(BF_4)_6$ before (bottom) and after addition of 4 eq pyridine over time at 298 K. Peaks at ~1 ppm have been scaled independently to rest of spectra due to relative intensity.

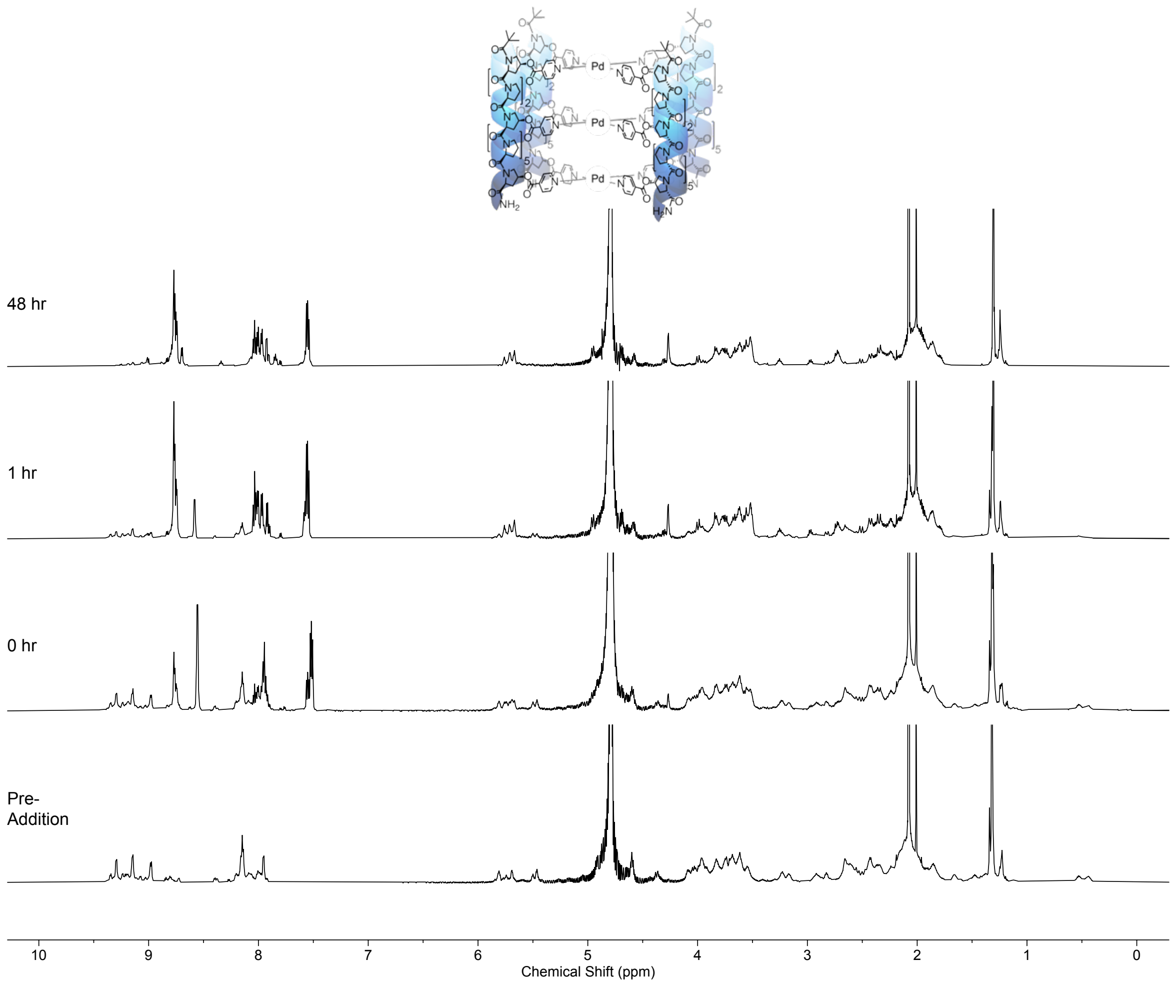

Figure S153. Stacked ^{1}H NMR (D_2O, 600 MHz, 298 K) of cage **$Pd_3L^{4,S}_4$**$(BF_4)_6$ before (bottom) and after addition of 12 eq pyridine over time at 298 K.

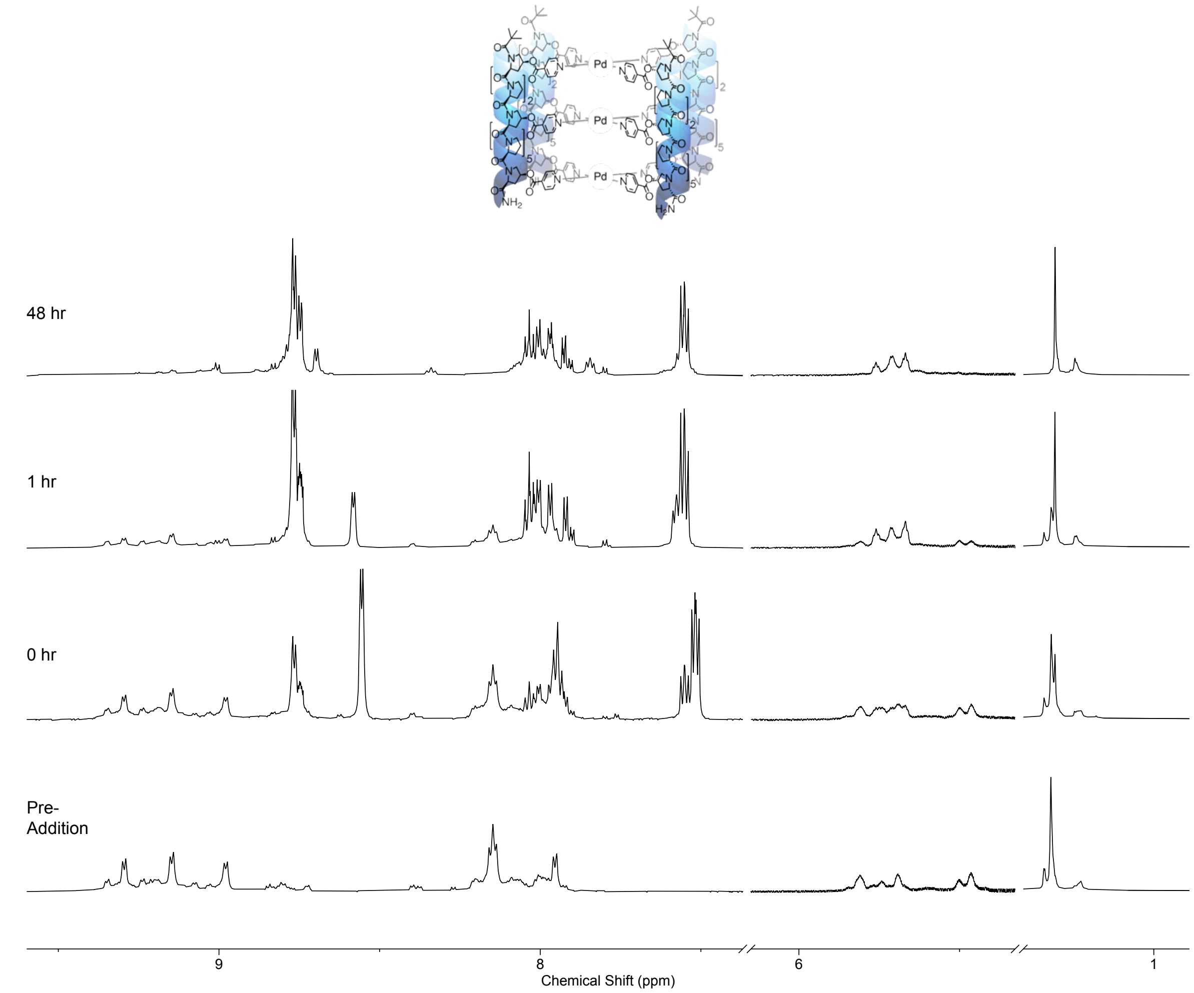

Figure S154. Partial stacked ^{1}H NMR (D_2O, 600 MHz, 298 K) of cage **$Pd_3L^{4,S}_4$**$(BF_4)_6$ before (bottom) and after addition of 12 eq pyridine over time at 298 K. Peaks at ~1 ppm have been scaled independently to rest of spectra due to relative intensity.

Glutathione Stability Studies

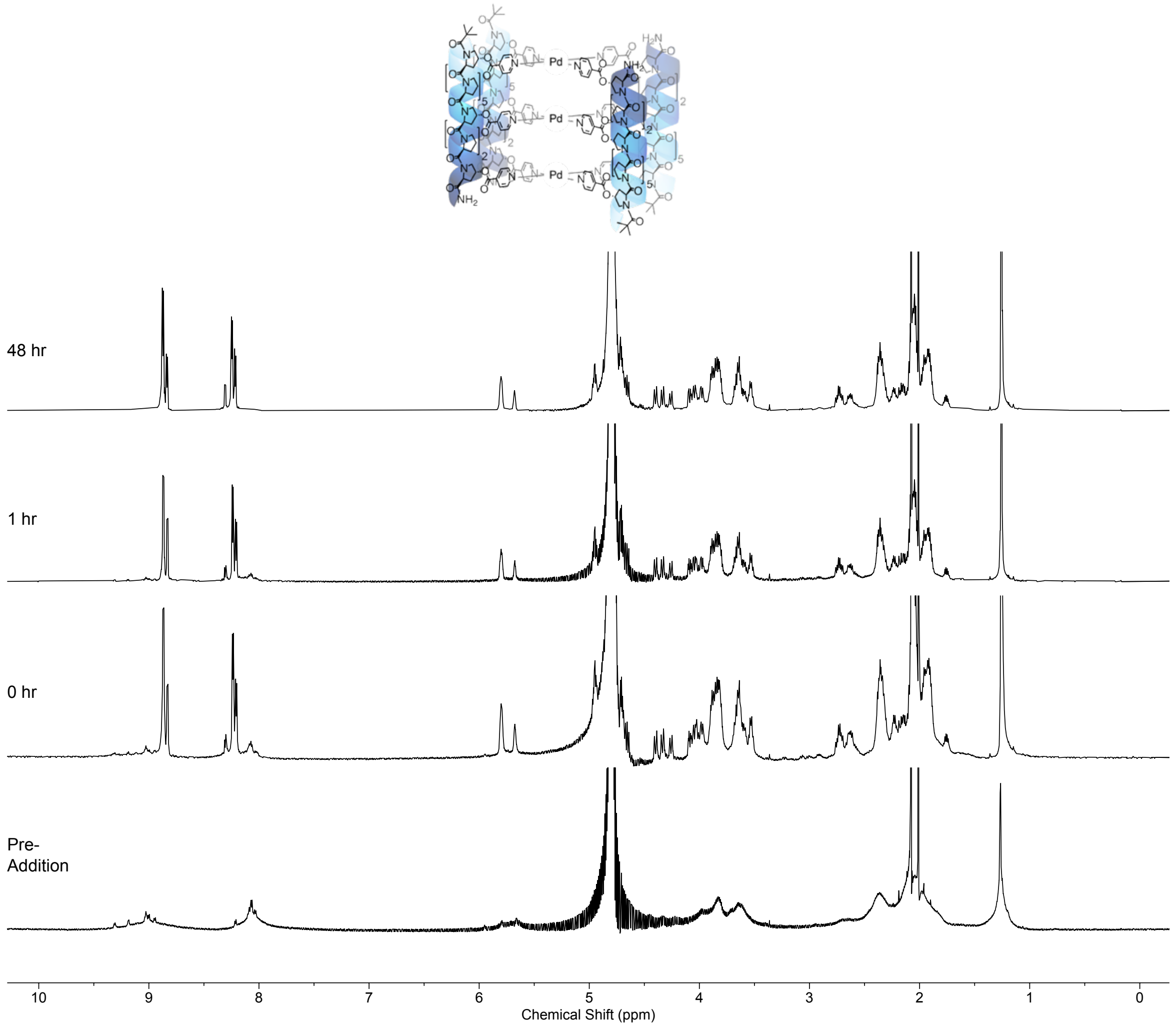

Figure S155. Stacked ^{1}H NMR (D_2O, 600 MHz, 298 K) of cage $\mathbf{Pd_3L^{7,R}_4}(BF_4)_6$ before (bottom) and after addition of 4 eq glutathione over time at 298 K.

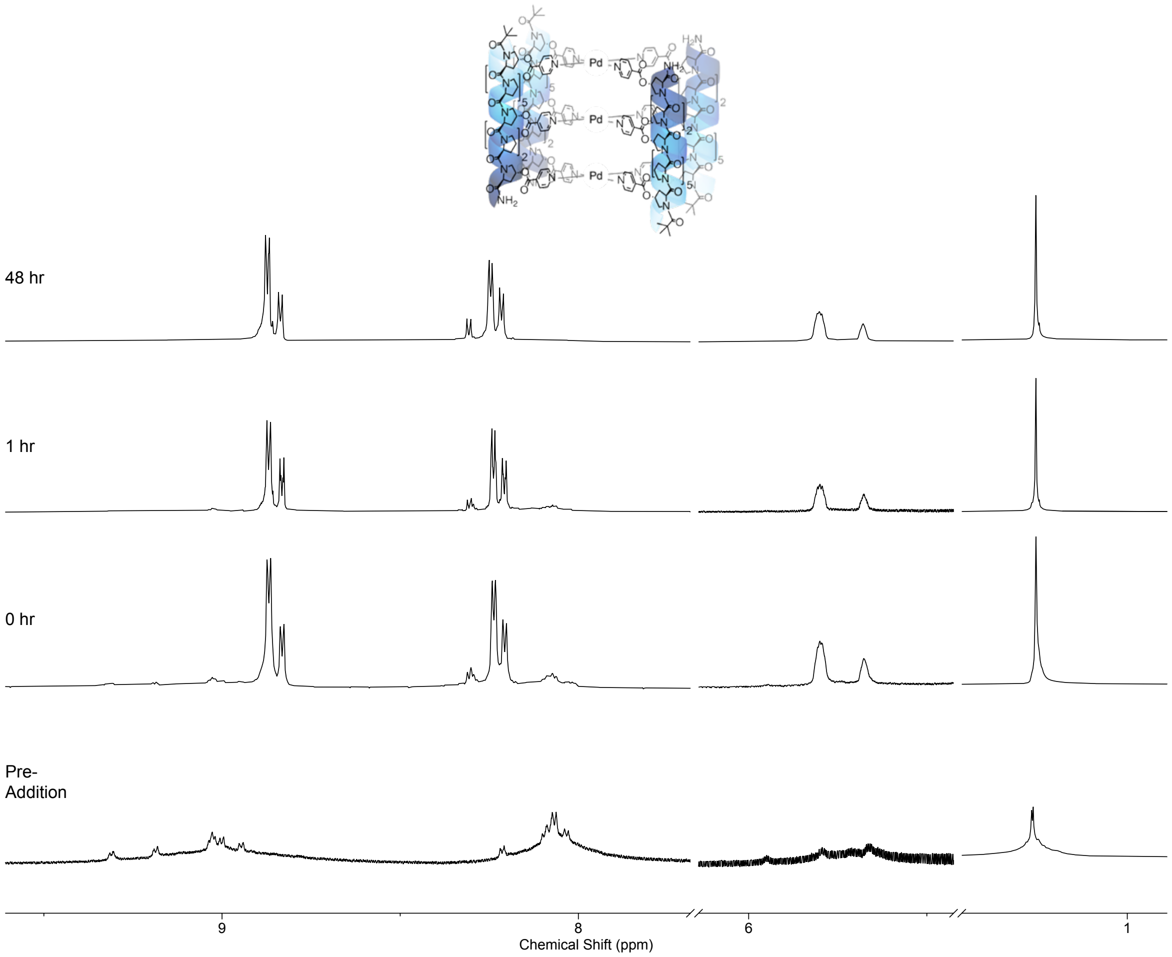

Figure S156. Partial stacked ^{1}H NMR (D_2O, 600 MHz, 298 K) of cage **$Pd_3L^{7,R}_4$**$(BF_4)_6$ before (bottom) and after addition of 4 eq glutathione over time at 298 K. Peaks at ~1 ppm have been scaled independently to rest of spectra due to relative intensity.

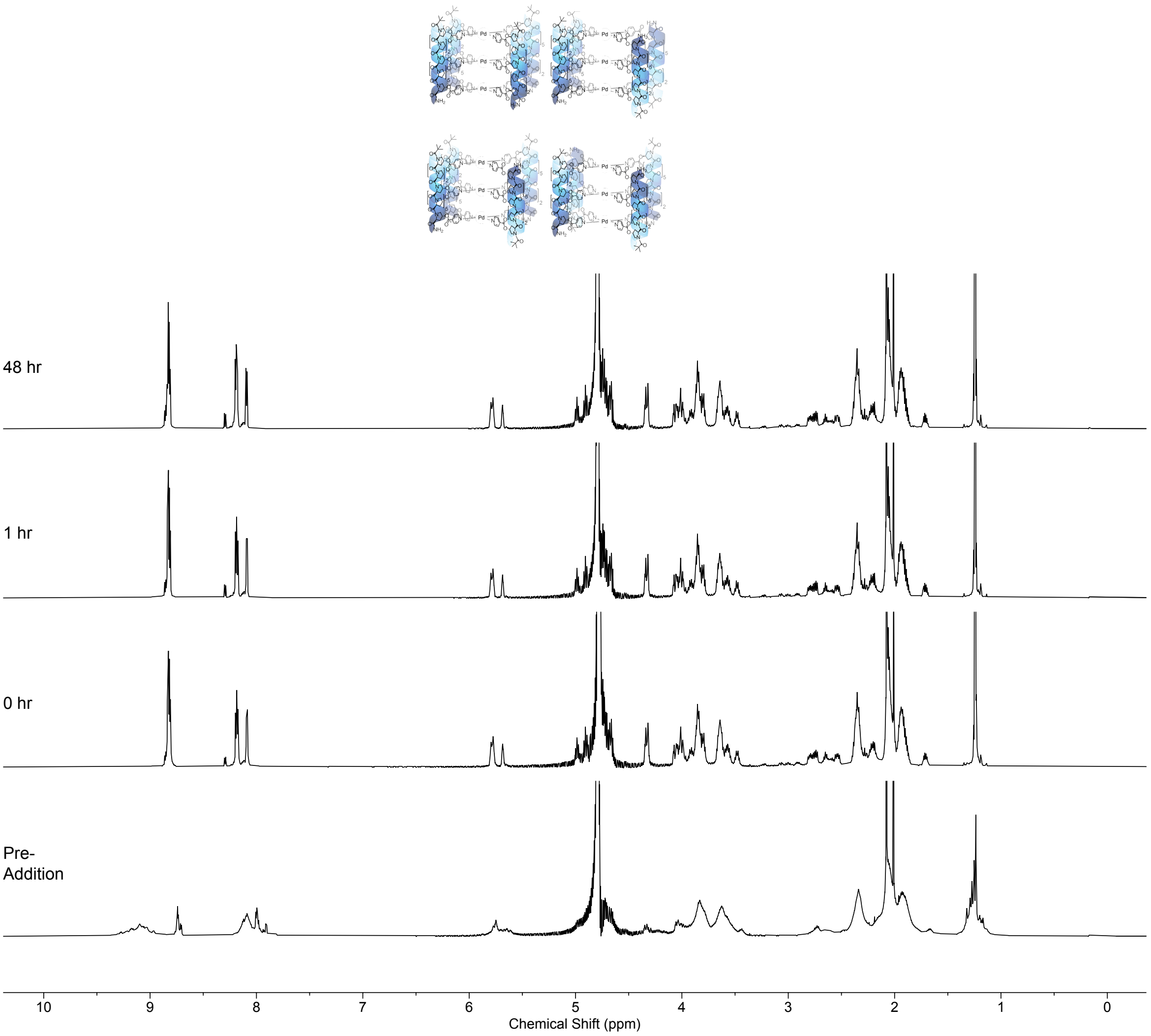

Figure S157. Stacked ^{1}H NMR (D_2O, 600 MHz, 298 K) of cage **$Pd_3L^{4,R}_4$**$(BF_4)_6$ before (bottom) and after addition of 4 eq glutathione over time at 298 K.

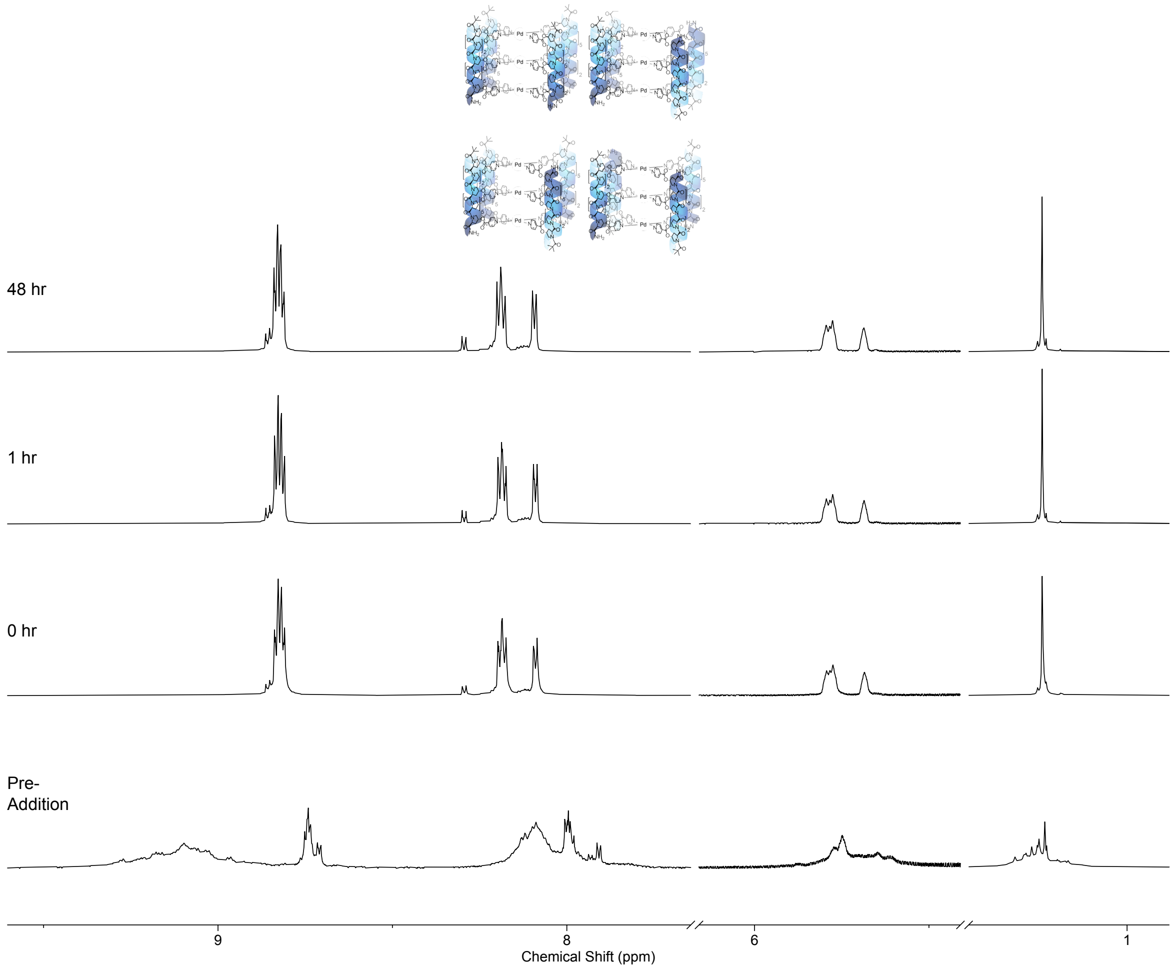

Figure S158. Partial stacked ¹H NMR (D_2O, 600 MHz, 298 K) of cage $\mathbf{Pd_3L^{4,R}_4}(BF_4)_6$ before (bottom) and after addition of 4 eq glutathione over time at 298 K. Peaks at ~1 ppm have been scaled independently to rest of spectra due to relative intensity.

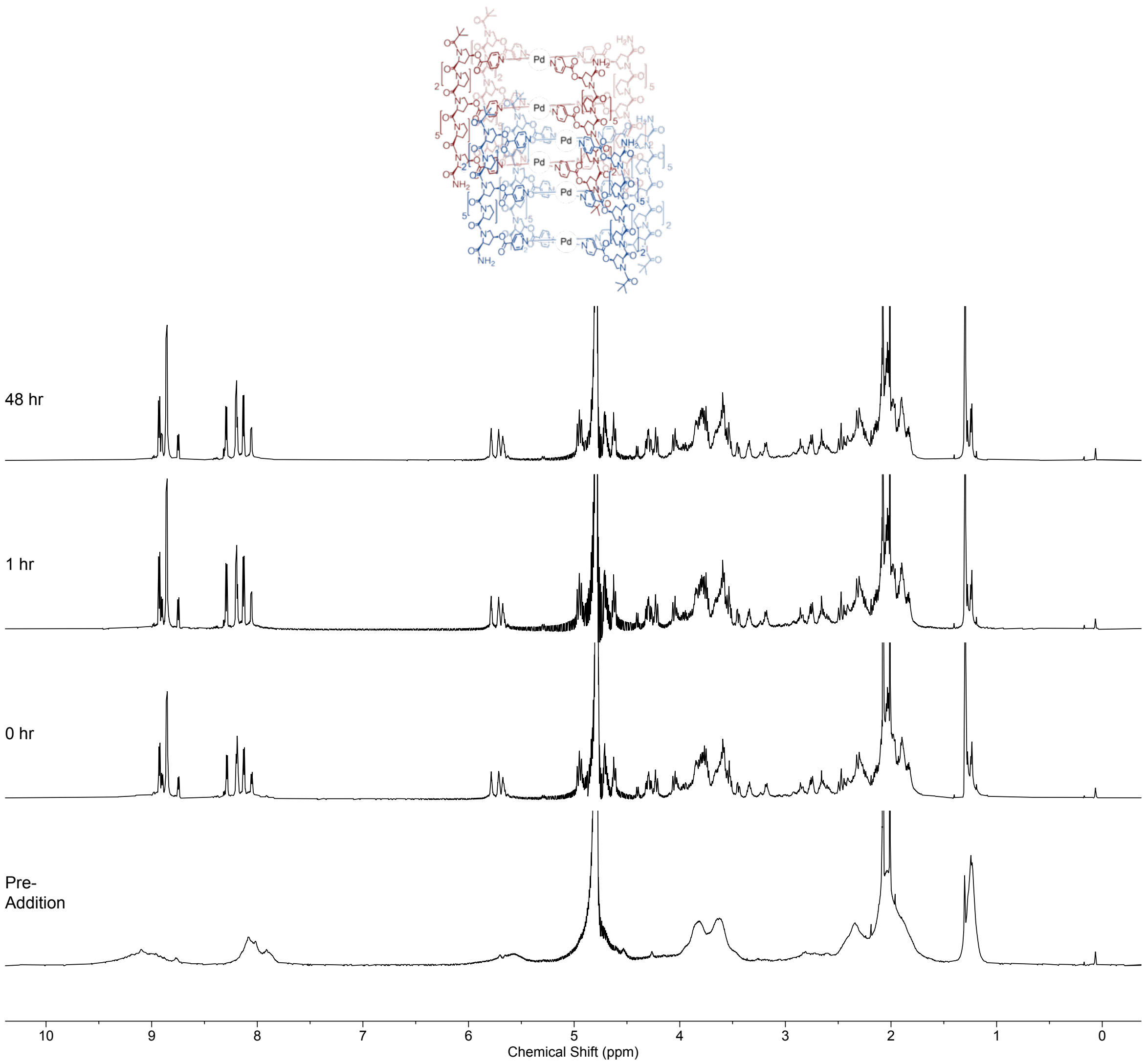

Figure S159. Stacked ^{1}H NMR (D_2O, 600 MHz, 298 K) of cage $[\mathbf{Pd_3L^{7,\mathit{S}}_4}(BF_4)_6]_2$ before (bottom) and after addition of 4 eq glutathione over time at 298 K.

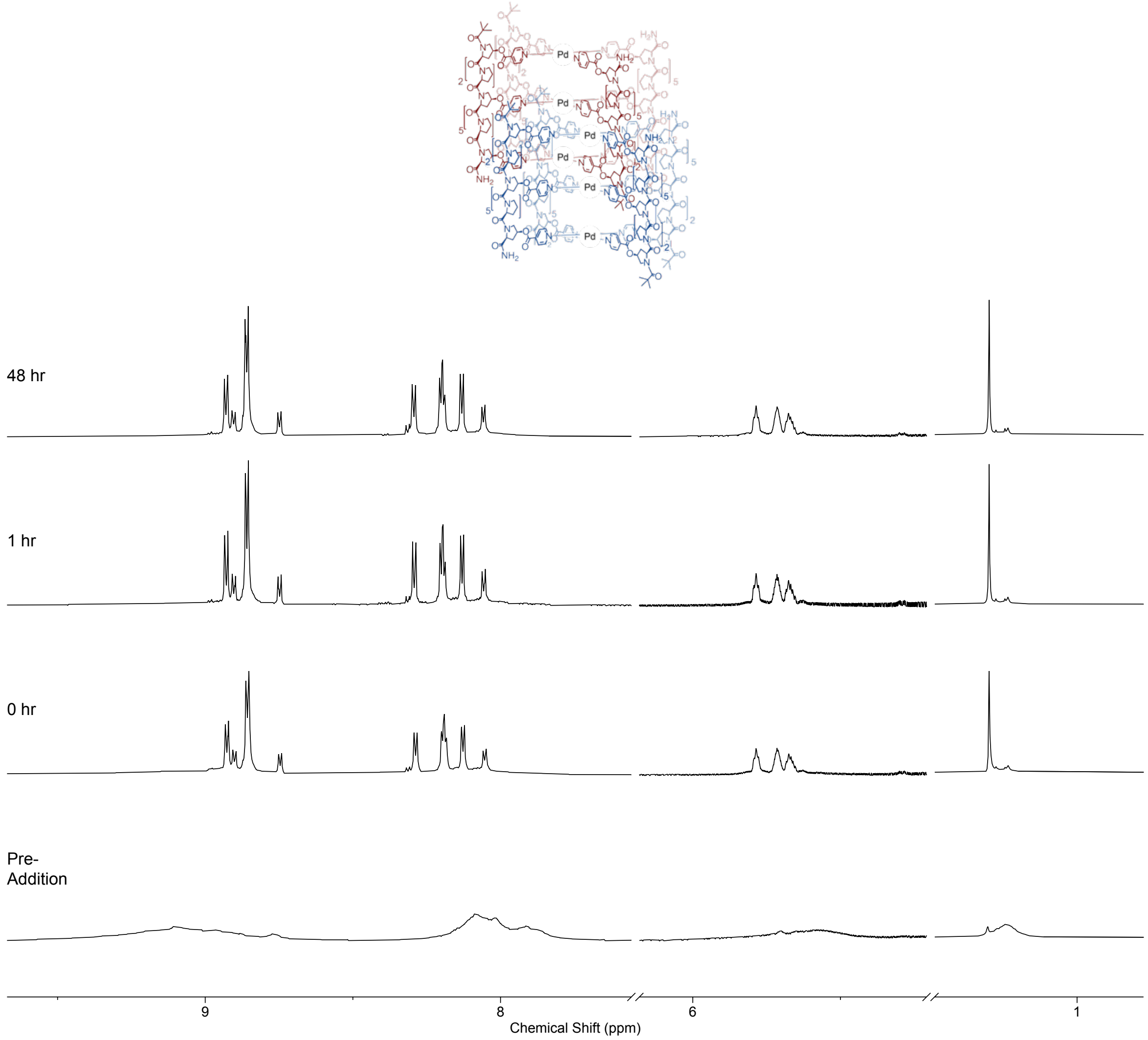

Figure S160. Partial stacked ^{1}H NMR (D_2O, 600 MHz, 298 K) of cage [**Pd$_3$L$^{7,S}_4$**$(BF_4)_6$]$_2$ before (bottom) and after addition of 4 eq glutathione over time at 298 K. Peaks at ~1 ppm have been scaled independently to rest of spectra due to relative intensity.

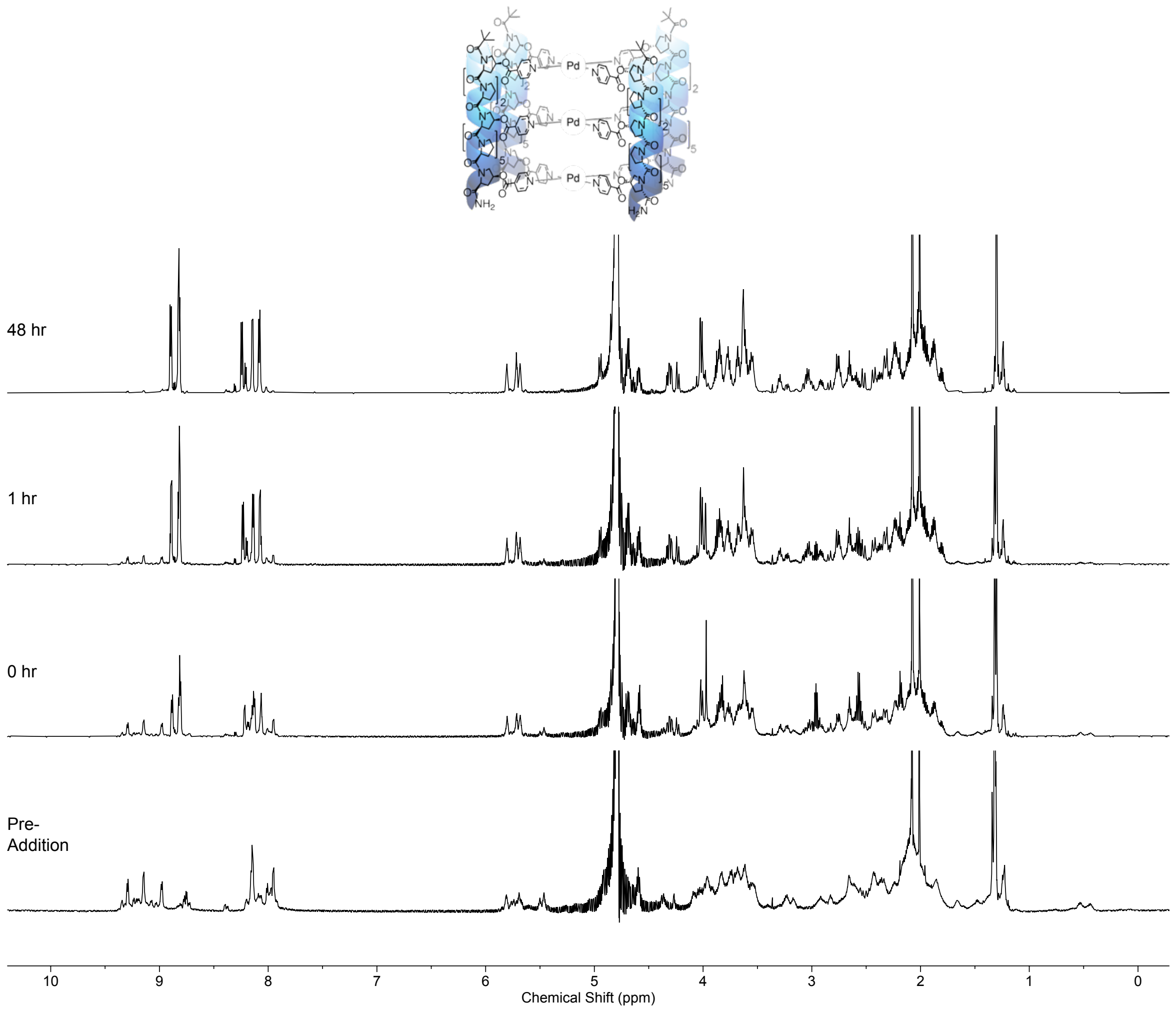

Figure S161. Stacked ^{1}H NMR (D_2O, 600 MHz, 298 K) of cage $\mathbf{Pd_3L^{4,S}_4}(BF_4)_6$ before (bottom) and after addition of 4 eq glutathione over time at 298 K.

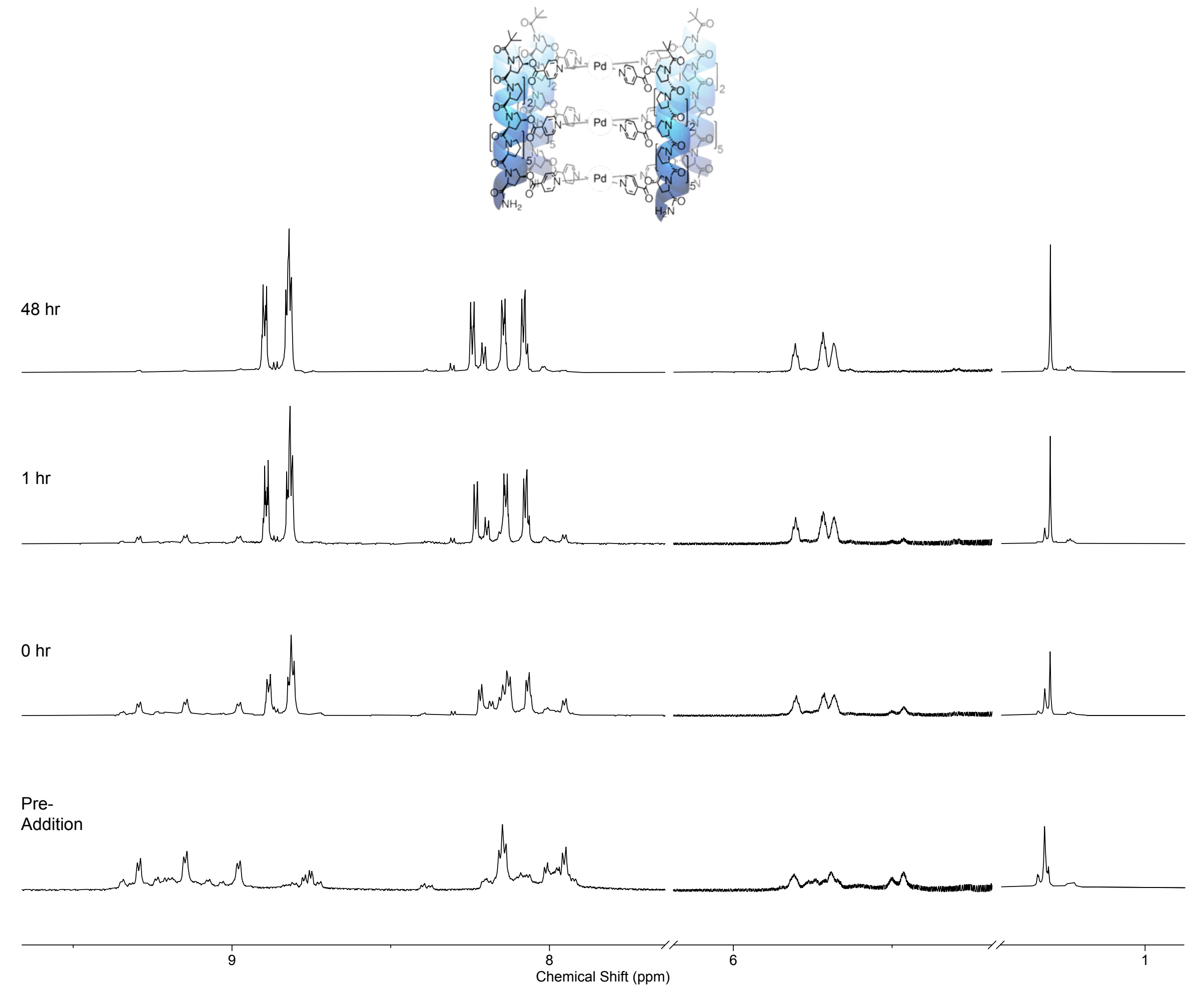

Figure S162. Partial stacked ^{1}H NMR (D_2O, 600 MHz, 298 K) of cage $\mathbf{Pd_3L^{4,\mathit{S}}_4}(BF_4)_6$ before (bottom) and after addition of 4 eq glutathione over time at 298 K. Peaks at ~1 ppm have been scaled independently to rest of spectra due to relative intensity.

8. Ion-Mobility Mass Spectrometry

IM-MS mobilograms for each sample display collision cross-sections consistent with expected sizes from molecular modelling and DOSY NMR. IM-MS mobilograms consisted of single broad-peaks with indistinguishable fine structure. We attribute this to the adoption of additional gas-phase conformations of each cage resulting from partial PPII/PPI conversion and/or *cis-trans* isomerisation of the ^{t}Bu capping group upon desolvation. The observation of differing conformations for oligoprolines in the gas-phase relative to solution has been extensively reported,[S5-S7] as *in vacuo* conditions remove interactions between water molecules and carbonyl groups of the peptide backbone which stabilise the all-*trans* PPII helix.

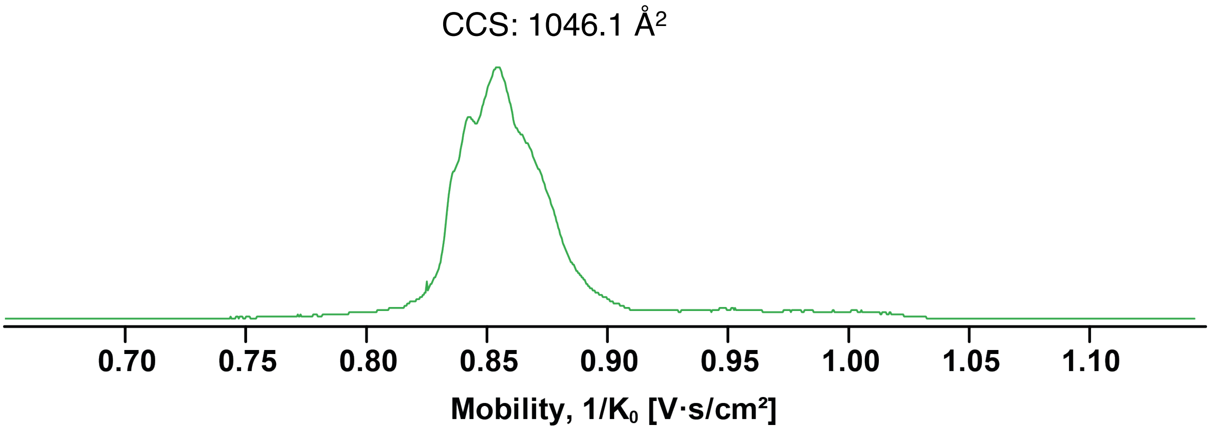

Figure S163. IM-MS of **$Pd_3L^{7,R}{}_4{}^{6+}$**.

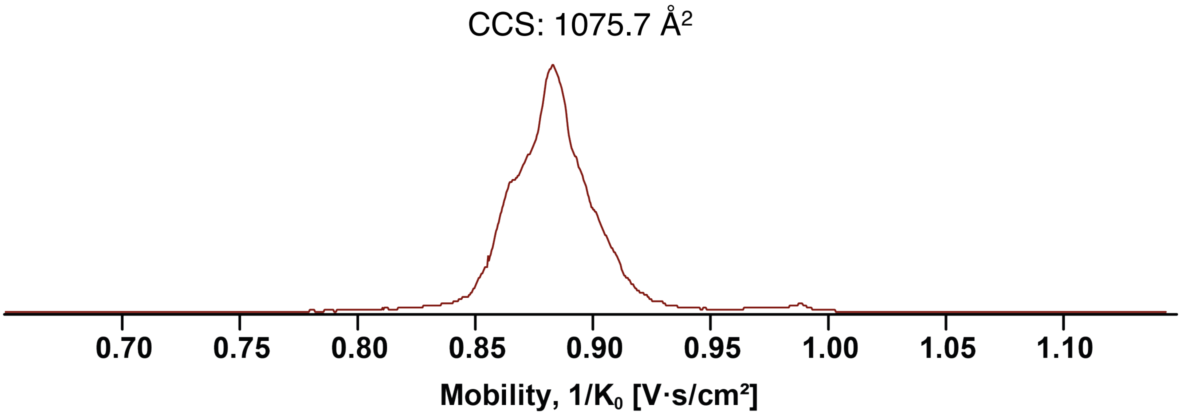

Figure S164. IM-MS of **$Pd_3L^{4,R}{}_4{}^{6+}$**.

Due to fragmentation under IM-MS conditions leading to low intensity peaks, we were unable to obtain a CCS of interpenetrated cage (**$Pd_3L^{7,S}{}_4$**)$_2{}^{12+}$, instead resolving the isolated **$Pd_3L^{7,S}{}_4{}^{6+}$** cage.

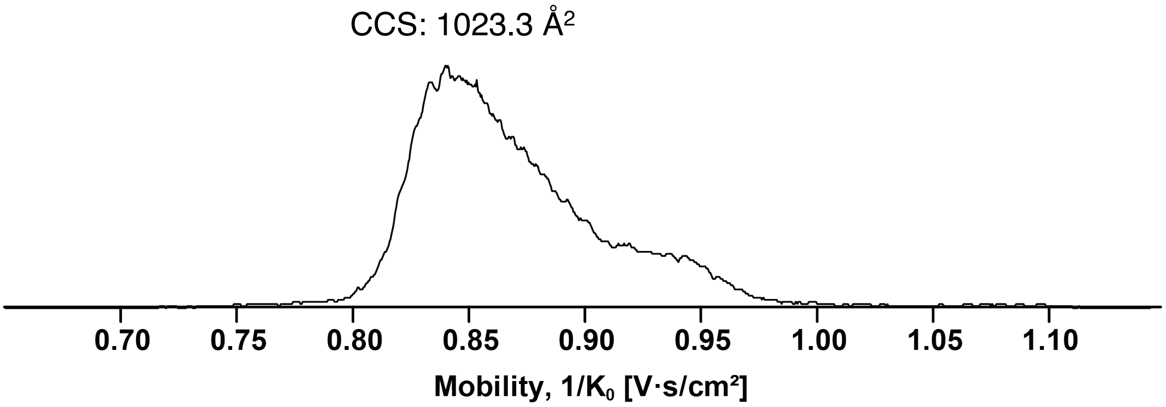

Figure S165. IM-MS of **$Pd_3L^{7,S}{}_4{}^{6+}$**.

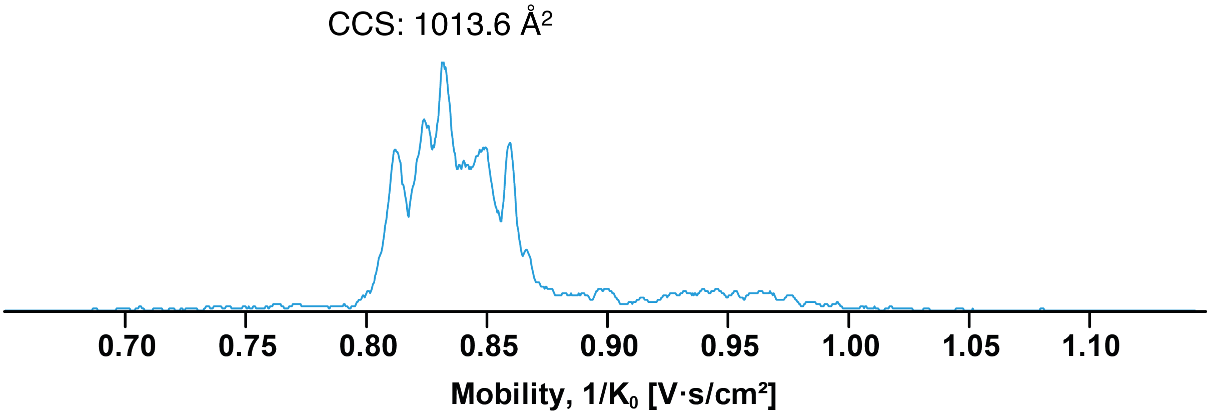

Figure S166. IM-MS of **$Pd_3L^{4,S}{}_4{}^{6+}$**.

9. CD Spectra Comparison

Herein are various comparisons of relative CD spectra to provide context and allow for easy comparison. Wherever ligand and cage have been compared, they are scaled relative to the concentration of ligand, not the molarity, to account for stoichiometry.

$Pd_3L^{7,R}{}_4$ $(BF_4)_6$ (Figure S167) and **$Pd_3L^{4,R}{}_4$**$(BF_4)_6$ (Figure S168) showed minimal changes upon complexation, supporting the retention of PPII character.

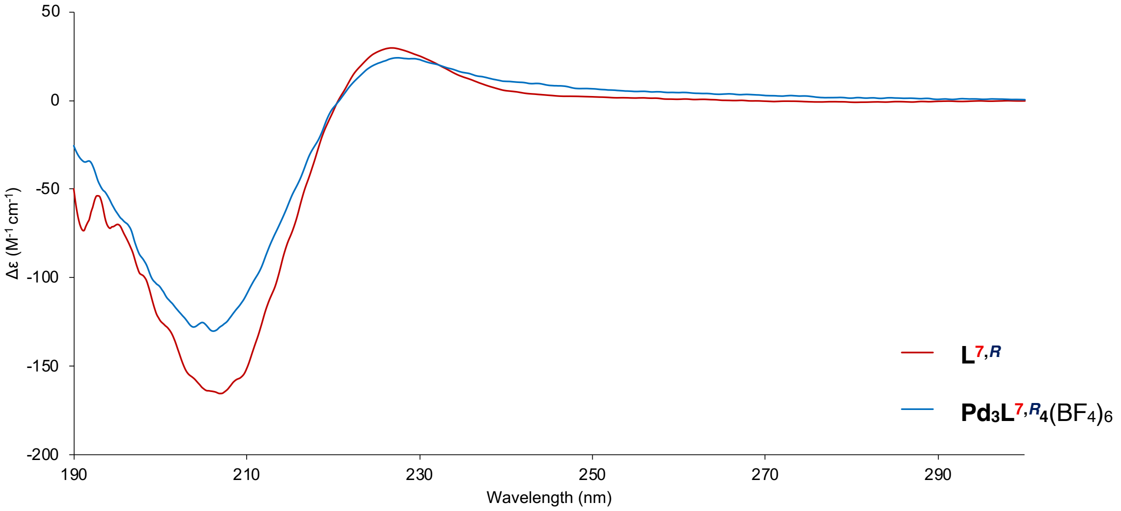

Figure S167. Comparison of CD spectra of **$L^{7,R}$** and **$Pd_3L^{7,R}{}_4$**$(BF_4)_6$ in H_2O (293 K).

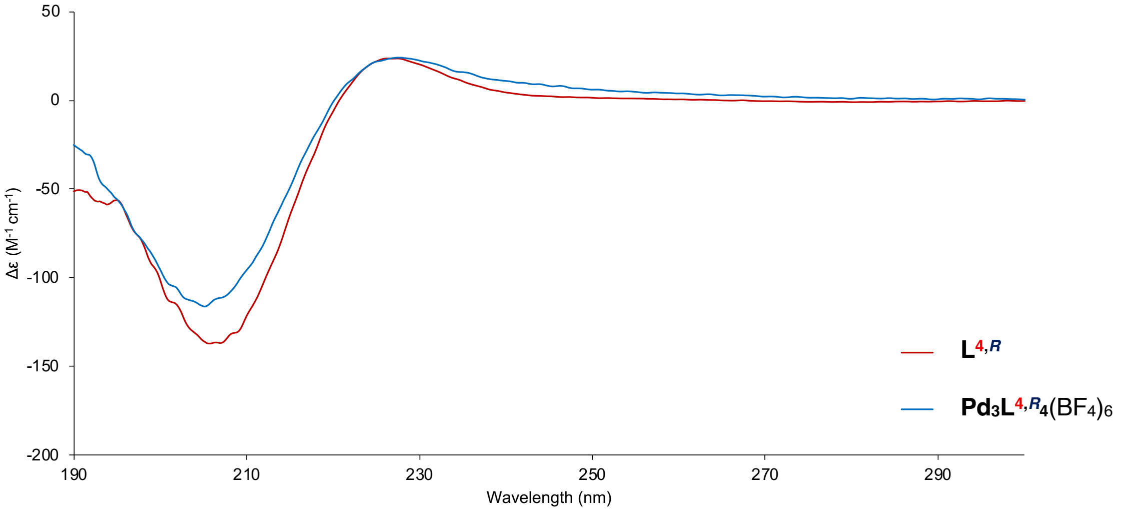

Figure S168. Comparison of CD spectra of **$L^{4,R}$** and **$Pd_3L^{4,R}{}_4$**$(BF_4)_6$ in H_2O (293 K).

There is a more significant reduction in the CD of [**Pd₃L^{7,S}₄**(BF₄)₆]₂ compared with ligand **L^{7,S}**, (Figure S169) suggesting reduction of PPII character upon complexation. This suggests that this cage undergoes a level of distortion on assembly.

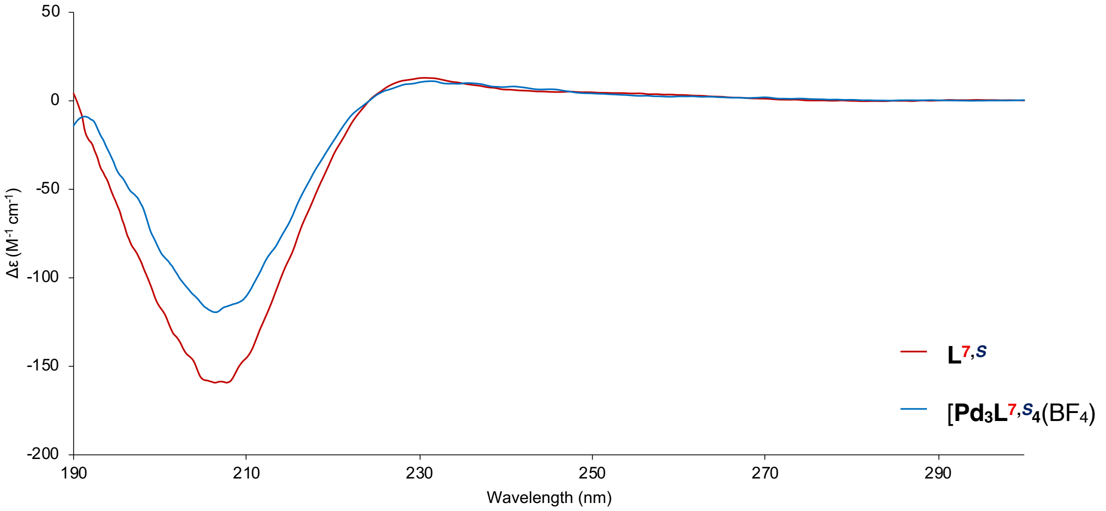

Figure S168. Comparison of CD spectra of **L^{7,S}** and [**Pd$_3$L^{7,S}$_4$**(BF$_4$)$_6$]$_2$ in H_2O (293 K).

The CD of **10**(BF$_4$)$_6$ compared with ligand **5** (Figure S170). The spectra maximum has shifted from 230 nm to 238 nm, a significant deviation to the shape, not typically observed in PPI or PPII. We assign these to a twisting and compression of the smaller cavity of **10**(BF$_4$)$_6$, with correlated interactions between the pyridine and proline residues driving changes in CD absorbance.

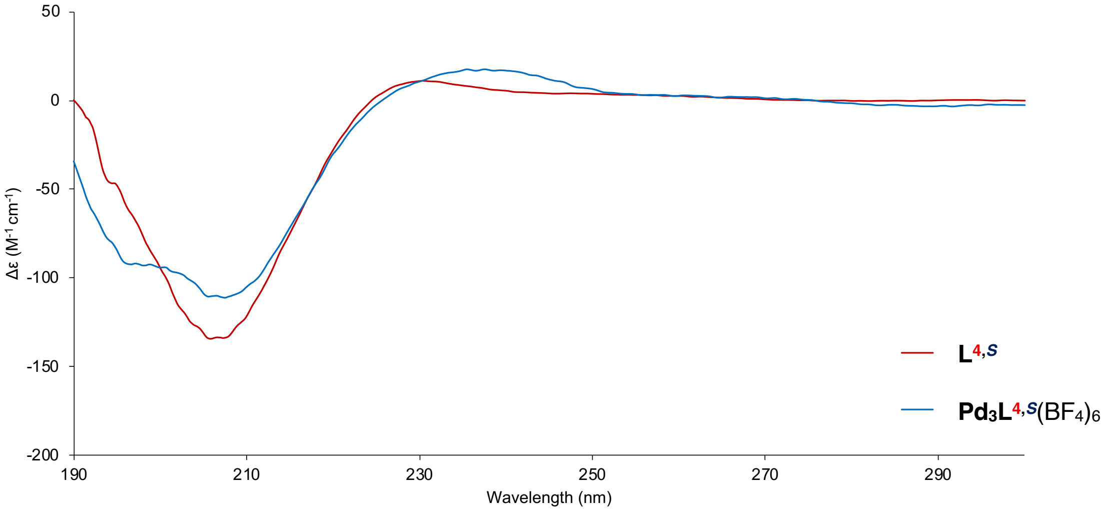

Figure S170. Comparison of CD spectra of **L^{4,S}** and **Pd$_3$L^{4,S}$_4$**(BF$_4$)$_6$ in H_2O (293 K).

Comparison of ligands **L**^{7,R} and **L**^{4,R} with **L**^{7,S} and **L**^{4,S} shows clearly the decreasing PPII character with the modification of the hydroxyproline chirality from 4*R* (PPII enhancing) to 4*S* (PPI enhancing, Figure S171).

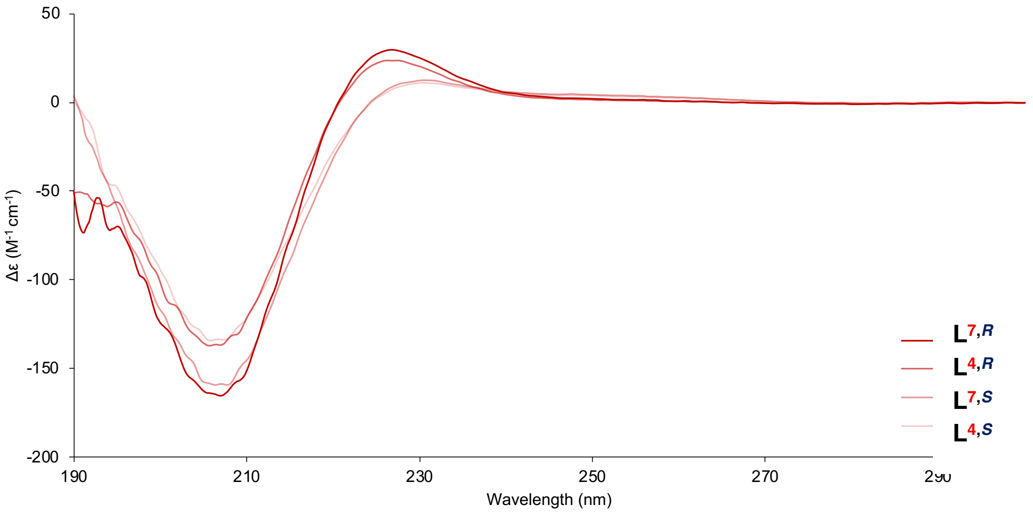

Figure S171. Comparison of CD spectra of all ligands, **L**^{7,R}, **L**^{4,R}, **L**^{7,S} and **L**^{4,S} in H_2O (293 K).

Comparison of cages **Pd$_3$L^{7,R}$_4$**$(BF_4)_6$, **Pd$_3$L^{4,R}$_4$**$(BF_4)_6$, [**Pd$_3$L^{7,S}$_4$**$(BF_4)_6]_2$ and **Pd$_3$L^{4,S}$_4$**$(BF_4)_6$ clearly shows the decreasing PPII character with the modification of the hydroxyproline chirality, and the unusual maximum shift for **Pd$_3$L^{4,S}$_4$**$(BF_4)_6$ (Figure S172).

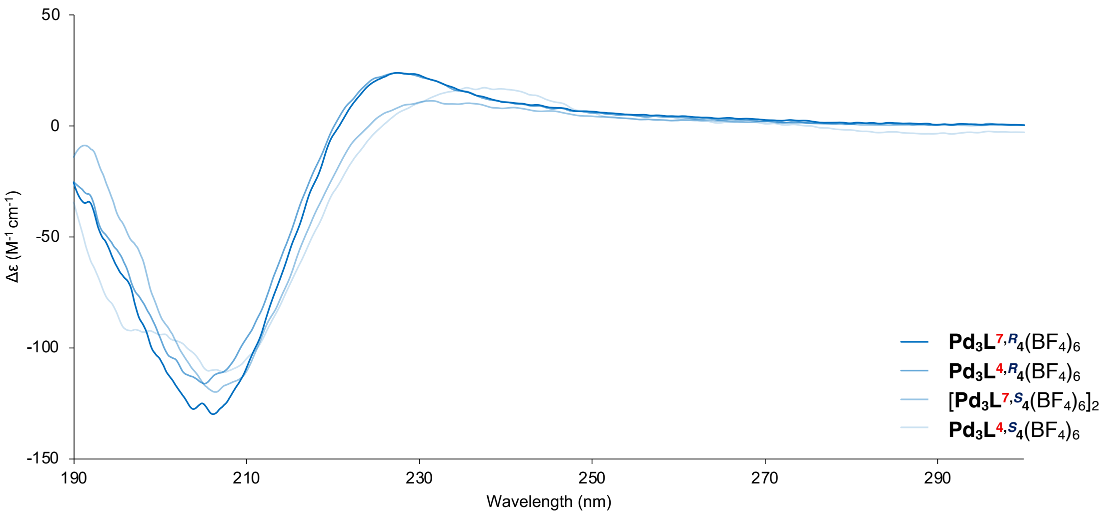

Figure S172. Comparison of CD spectra of all cages, **Pd$_3$L^{7,R}$_4$**$(BF_4)_6$, **Pd$_3$L^{4,R}$_4$**$(BF_4)_6$, [**Pd$_3$L^{7,S}$_4$**$(BF_4)_6]_2$ and **Pd$_3$L^{4,S}$_4$**$(BF_4)_6$ in H_2O (293 K).

Comparisons of ligand **L**^{7,R} with **L**^{7,S} and cage **Pd$_3$L**^{7,R}**$_4$**$(BF_4)_6$ with [**Pd$_3$L**^{7,S}**$_4$**$(BF_4)_6]_2$, clearly showing the decreasing PPII character due to diastereoisomerism (Figures S173 + S174).

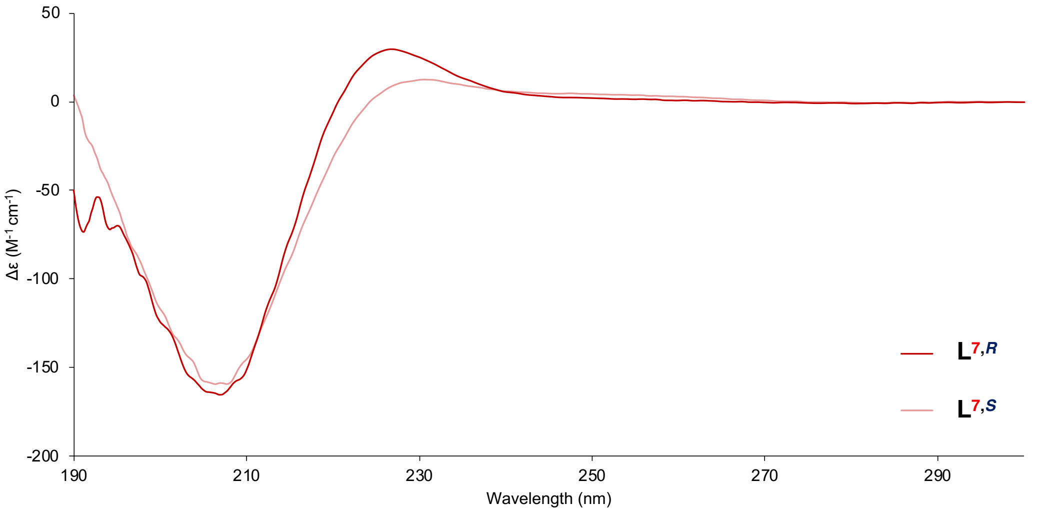

Figure S173. Comparison of CD spectra of ligands **L**^{7,R} and **L**^{7,S} in H_2O (293 K).

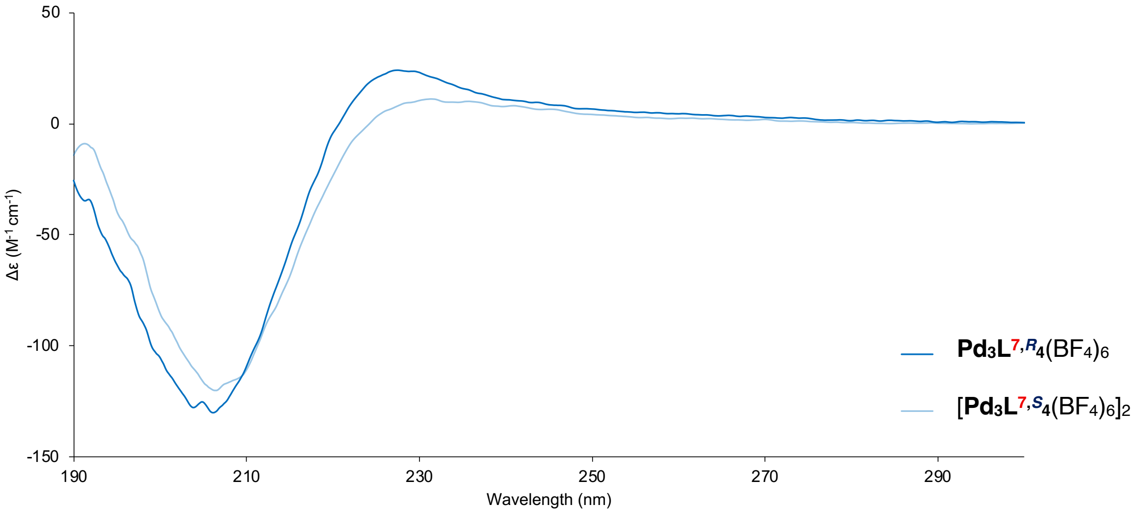

Figure S174. Comparison of CD spectra of **Pd$_3$L**^{7,R}**$_4$**$(BF_4)_6$ and [**Pd$_3$L**^{7,S}**$_4$**$(BF_4)_6]_2$ in H_2O (293 K).

Comparisons of ligand **L**^{4,R} with **L**^{4,S} and cage **Pd$_3$L**^{4,R}**$_4$**$(BF_4)_6$ with **Pd$_3$L**^{4,S}**$_4$**$(BF_4)_6$, clearly showing the decreasing PPII character due to diastereoisomerism (Figures S175 + S176).

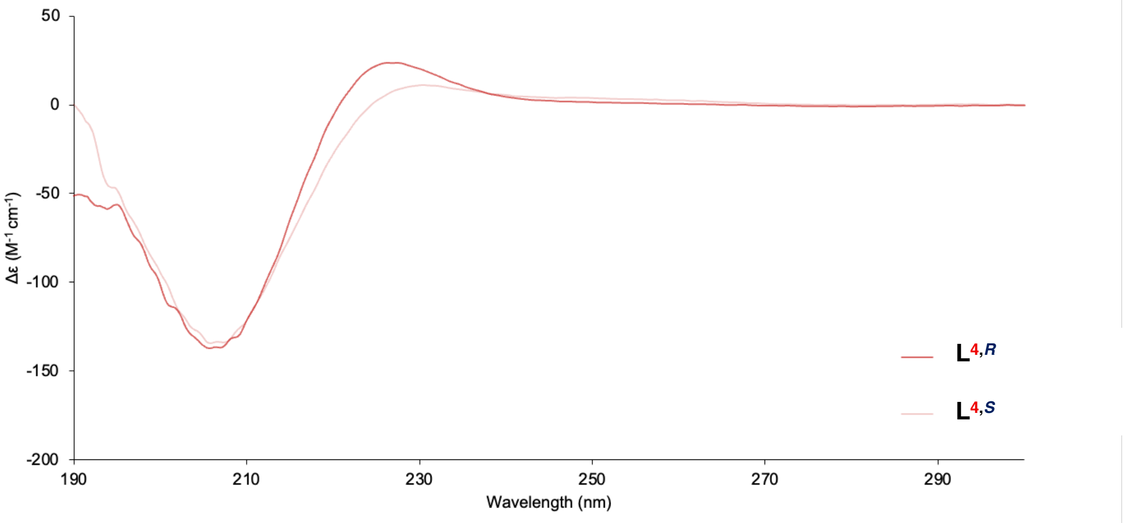

Figure S175. Comparison of CD spectra of ligands **L**^{4,R} and **L**^{4,S} in H_2O (293 K).

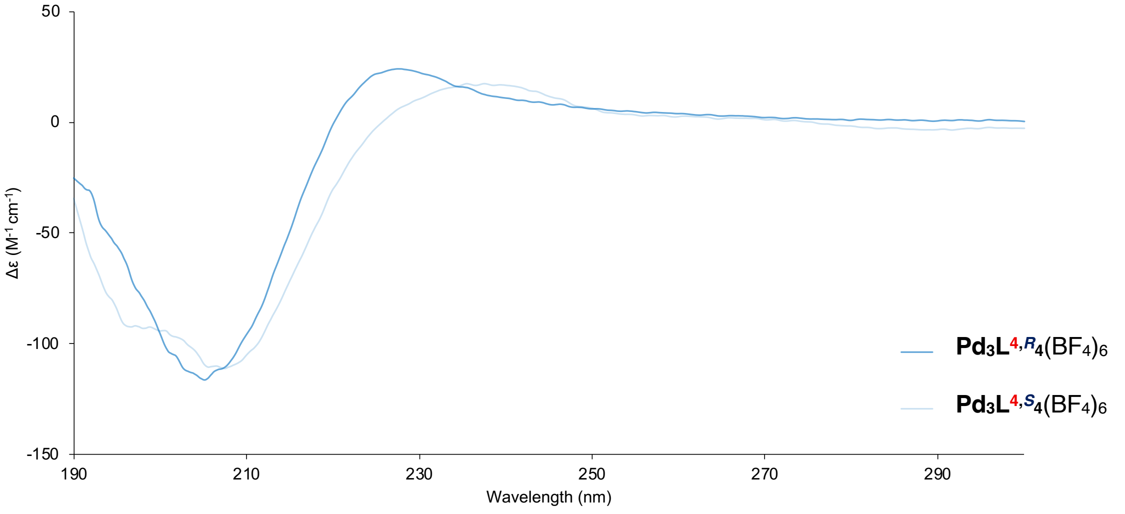

Figure S176. Comparison of CD spectra of **Pd$_3$L**^{4,R}**$_4$**$(BF_4)_6$ and **Pd$_3$L**^{4,S}**$_4$**$(BF_4)_6$ in H_2O (293 K).

Comparisons of ligands **L**^{7,R} with **L**^{4,R} and cage **Pd**$_3$**L**^{7,R}$_4$(BF_4)$_6$ with **Pd**$_3$**L**^{4,R}$_4$(BF_4)$_6$, showing minimal effect on PPII character due to structural isomerism of ligand (i.e. shorter unit at *C*- or *N*-terminus, Figures S177 + S178).

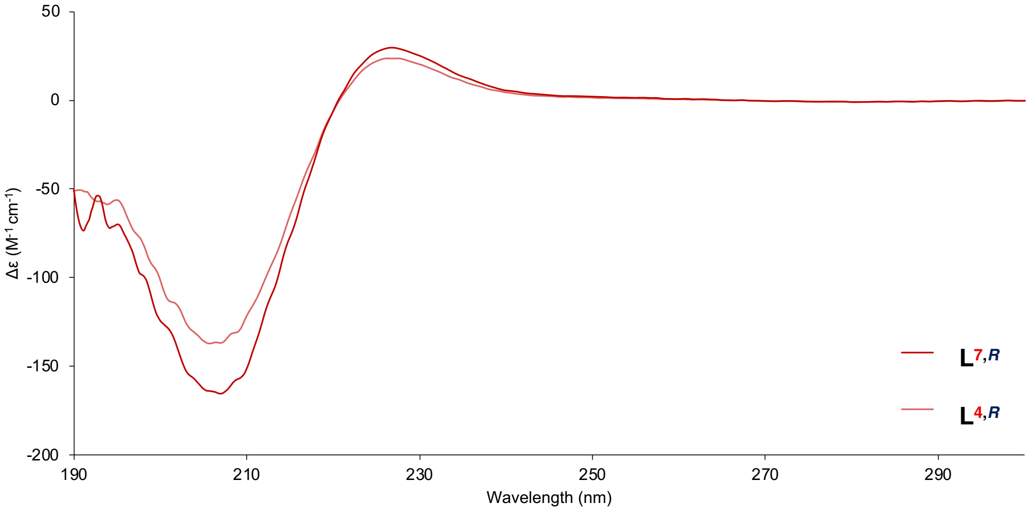

Figure S177. Comparison of CD spectra of ligands **L**^{7,R} and **L**^{4,R} in H_2O (293 K).

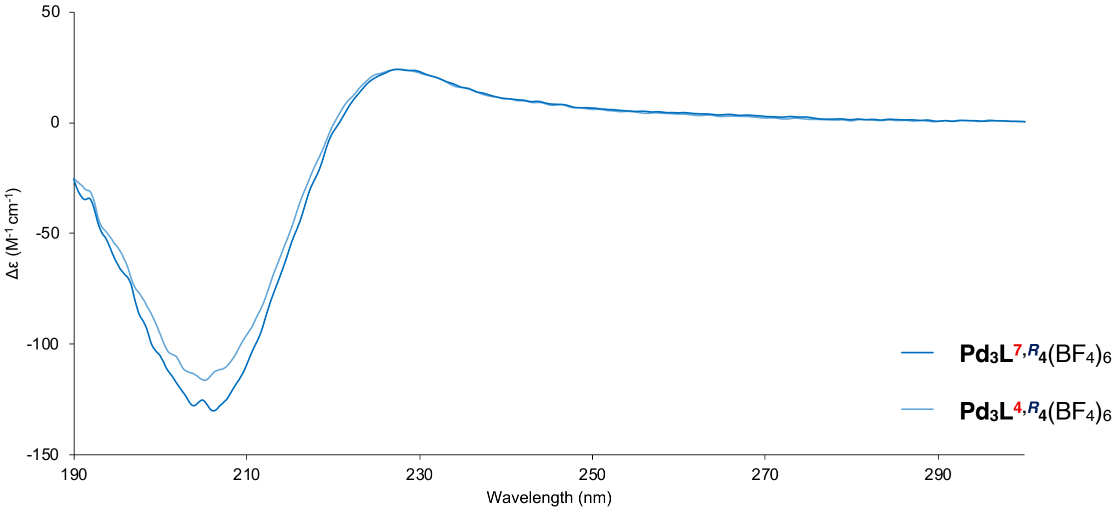

Figure S178. Comparison of CD spectra **Pd**$_3$**L**^{7,R}$_4$(BF_4)$_6$ and **Pd**$_3$**L**^{4,R}$_4$(BF_4)$_6$ in H_2O (293 K).

Comparisons of ligands **L**^{7,S} with **L**^{4,S} showing minimal effect on PPII character due to structural isomerism in the 4*S* series (Figure S179).

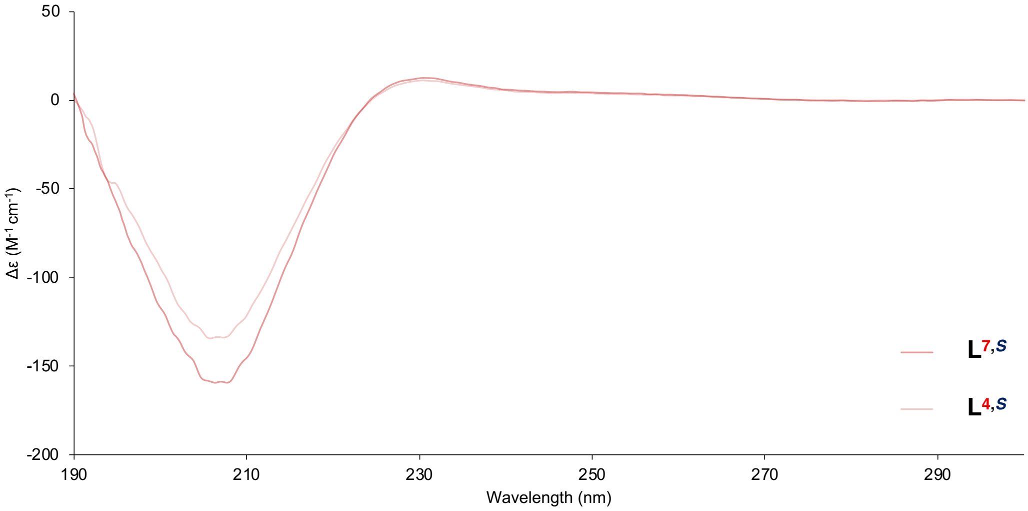

Figure S179. Comparison of CD spectra of **L**^{7,S} and **L**^{4,S} in H_2O (293 K).

Comparisons of cages [**Pd**$_3$**L**^{7,S}$_4$(BF_4)$_6$]$_2$ with **Pd**$_3$**L**^{4,R}$_4$(BF_4)$_6$, shows major deviations in CD spectra upon cage complexation, suggesting significant deviations from standard PPII structures (Figure S180).

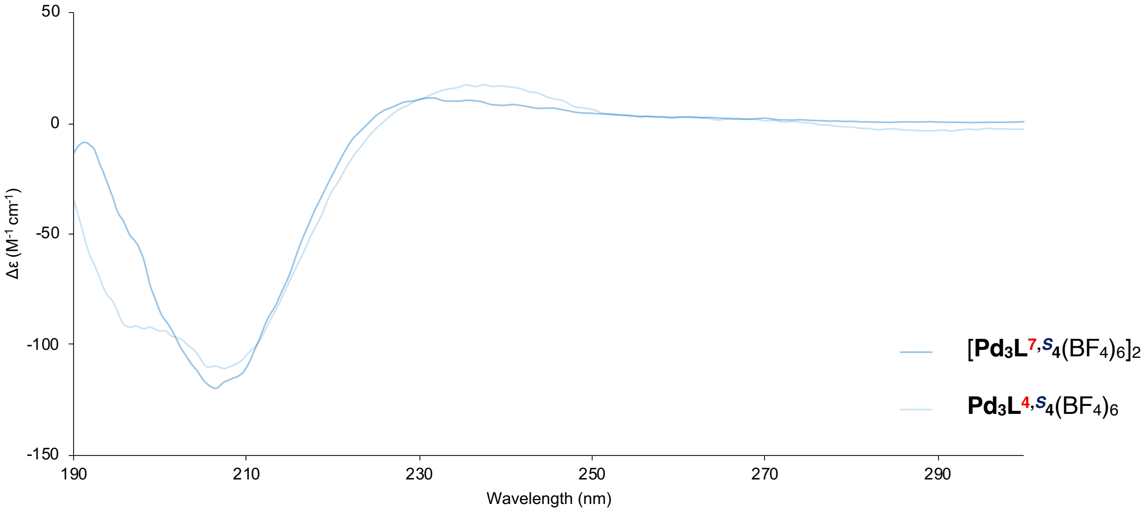

Figure S180. Comparison of CD spectra of **Pd**$_3$**L**^{7,S}$_4$(BF_4)$_6$ and [**Pd**$_3$**L**^{4,S}$_4$(BF_4)$_6$]$_2$ in H_2O (293 K).

10. Isomerisation Discussion

In this section, we will discuss some of the complexities of the isomerism that arises in our metal-peptidic cages.

Our helical peptides are intrinsically directional – as the *N*-terminus is distinct from the *C*-terminus. As such, when our ligands are assembled into lantern type M_XL_4 cages, there are four different isomers that can occur, 'All Up' *CCCC*, 'Three Up' *CCCN*, *cis CCNN*, and *trans CNCN*.

This orientation is a consequence of the removal of the C_{2v} axis present in lantern cages with non-directional ligands, and is an achiral feature. In achiral systems, the only arrangement that results in a desymmetrisation of the ligand environments is the 'Three Up' *CCCN.*

In our system, the inherent chirality results in a facial desymmetrisation of the ligand, giving each ligand a defined 'left' and 'right' face. This ligand desymmetrisation breaks some of the symmetry elements that are present in achiral systems. For example, in the *cis CCNN* cage, the central mirror plane is removed, giving rise to two distinct ligand environments rather than one.

The introduction of a third, internal, ligand coordinating unit influences the relative ligand orientation and isomer selectivity within the cage system. The *cis CCCN* and *trans CNCN* in particular are difficult to model, and will require some level of deformation either to the palladium coordination plane, or to the ligand linearity.

In our previous publication,[S3] we discussed in detail how it is possible to prove the isomerism present in Pd_2L_4 systems by considering the symmetry elements present in each structural isomer, and relating this to the number of ligand environments and the NOE effects that are observed. We summarise this below; however, we direct readers to our previous paper ESI Section 10,[S3] for a more detailed discussion (Figure S181).

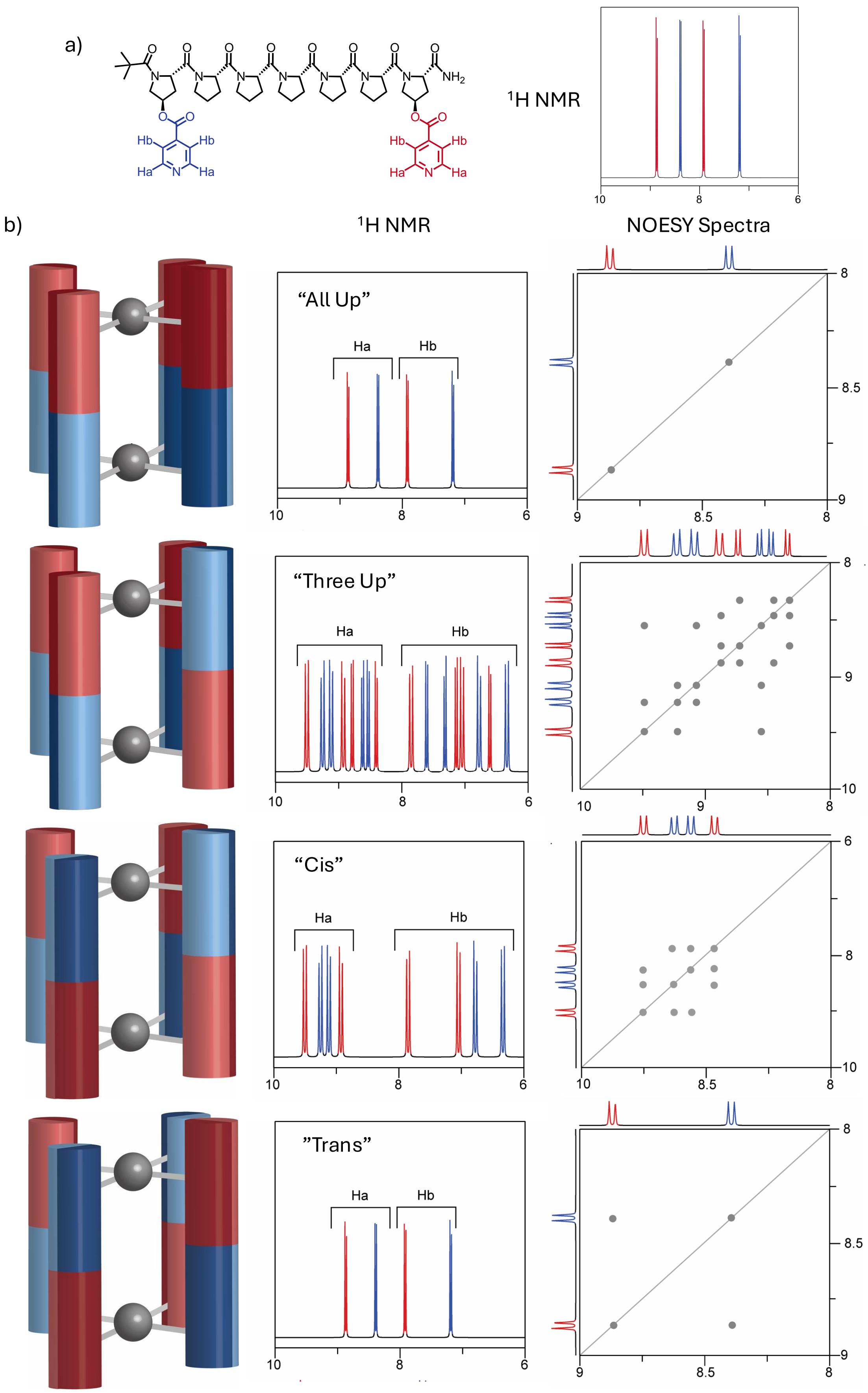

Figure S181. Summary of Pd_2L_4 cage isomerism a) showing the ligand and a hypothetical, simplified ^{1}H NMR spectrum, and b) showing each possible cage isomer, and their simplified, hypothetical partial ^{1}H NMR and NOESY spectra, illustrating how each structural isomer can be identified.

It is important to consider, however, that the above representations are idealised and purely hypothetical and, therefore, do not account for any increase in complexity arising from trapped rotations or increased/decreased proximity and so NOE effects due to unexpected steric constraints.

In an idealised representation, the introduction of a third coordination unit, to generate Pd_3L_4 systems, does not change the number of ligand environments expected to arise for each structural isomer. As it is not possible to account for all deformations and models that could theoretically arise, we begin by considering each system based on symmetry operations and the resulting number of ligand environments, before further analysing the 2D data.

As both NMR and CD spectra indicate a reduction in PPII character in ligand $\mathbf{L}^{7,S}$ and $\mathbf{L}^{4,S}$ containing 4*S* centres, compared with ligands $\mathbf{L}^{7,R}$ and $\mathbf{L}^{4,R}$ containing 4*R* centres, it is likely that ligands $\mathbf{L}^{7,S}$ and $\mathbf{L}^{4,S}$ experience a level of deformation away from PPII. This may either impact linearity or the metal binding coordination centre, but is difficult to represent in simplified model systems like the ones above. We therefore use these representations as a best approximation and suggest they should be taken as a guide.

Cage $\mathbf{Pd_3L^{7,R}_4}(BF_4)_6$

We have assigned cage $\mathbf{Pd_3L^{7,R}_4}(BF_4)_6$ as the *cis CCNN* cage as the major isomer, supported by analysis of the 1D and 2D NMR data discussed below.

In $\mathbf{Pd_3L^{7,R}_4}(BF_4)_6$, we observe two distinct and equal ligand environments that are in separated spin systems. This splitting of the entire ligand system, and not just of the aromatic signals as we would expect from a restricted rotation, is only consistent with formation of a *cis CCNN* cage isomer with diastereotopic splitting. This is analogous to our previously reported Pd_2L_4 *cis CCNN* systems, where the *cis CCNN* preference arises from a ‘tilt’ in the cage.[S3]

We would expect the NOESY correlations of the aromatic signals in this cage to be similar to those observed for our previous Pd_2L_4 systems, whereby each of the four separate pyridine environments (left and right *N*-terminus, and left and right *C*-terminus) show correlations with two other, distinct, pyridine environments. The two aromatics next to each pyridine are close contacts, and the pyridines ‘trans’ to one another are less likely to interact, though there have been previous reports of contacts being possible between *trans* pyridines.[S8]

Additionally, we must also consider the NOESY correlations that would arise from the internal coordinating motifs. These two additional pyridine environments, corresponding to the left and right ligands, would be isolated from the ‘terminal’ coordination motifs and therefore would see correlations between one another but would be unlikely to see the other peaks (Figure S182).

In summary, the ‘outside’ palladiums are coordinated to four different pyridines, and are related to one another *via* a rotational axis of symmetry. The internal palladium is

only coordinated to the internal pyridines, resulting in a system where each pyridine sees it's magnetic equivalent through one side, (therefore no NOE), and it's diastereotopic partner through the other side (therefore one NOE). This results in 2 signals being in one 'group' of NOESY effects, and 4 signals being in the other.

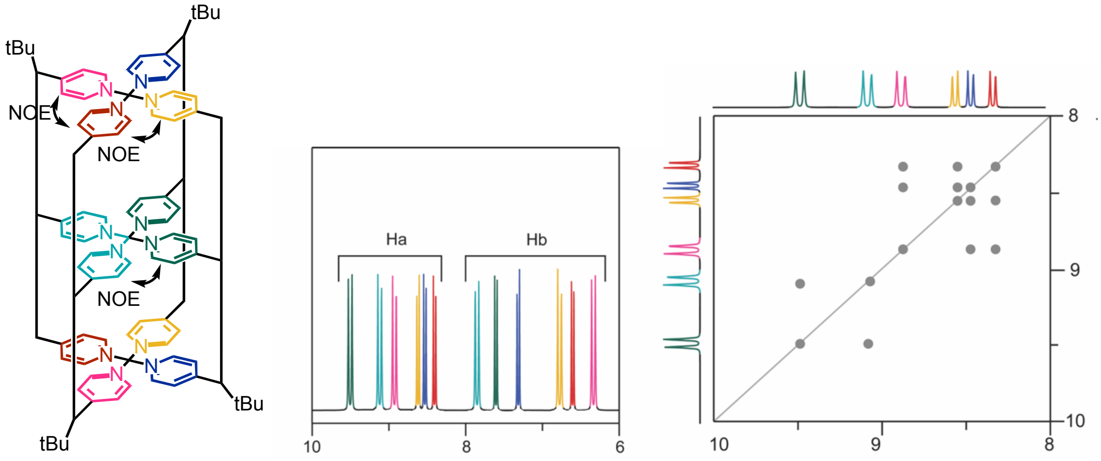

Figure S182. Representation of *cis CCNN* Pd_3L_4 cage with differing aromatic environments highlighted and colour coordinated, showing the desymmetrisation between the left and right side, and an illustrated example of the expected NOE relations.

In the NOESY spectrum of **$Pd_3L^{7,R}_4$**$(BF_4)_6$, we observe an isolated square NOE pattern between two different pyridine environments, with no COSY correlation between the signals (Figure S43), which we can therefore attribute to internal residues coordinated to the same metal centre (Figure S183), and the presence of the *cis CCNN* isomer of cage.

Looking at the top and bottom spin systems, it is harder to discern specific correlations, as the peaks are close together, and so we are at the limit of the peak resolution. However, it is obvious that they are interacting, and are all in close proximity to one another, supporting our assignment of the *cis CCNN* isomer of cage.

As each ligand has three pyridine environments, the observed doubling to six pyridine environments upon cage formation (of which four are in one environment, and two are in another) allows us to confidently assign **$Pd_3L^{7,R}_4$**$(BF_4)_6$, as a *cis CCNN* cage, with a horizontal C_2 axis of rotation symmetry.

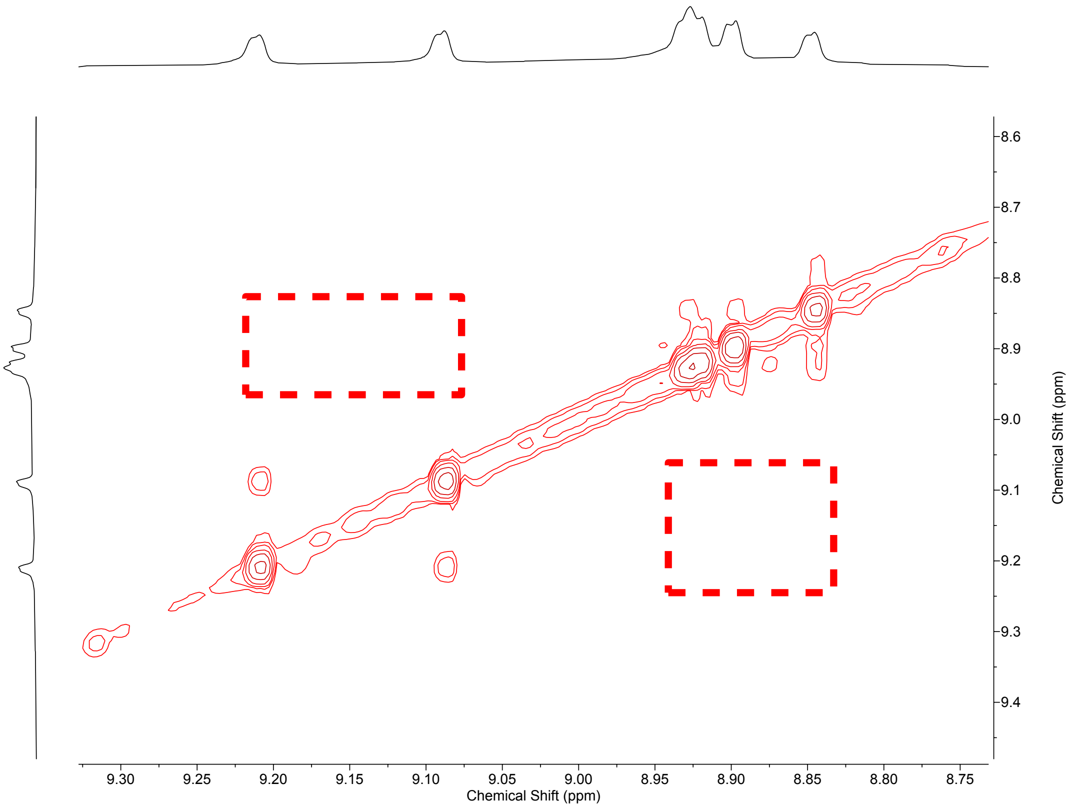

Figure S183. Partial 950 MHz NOESY spectrum of **$Pd_3L^{7,R}_4$**$(BF_4)_6$, showing correlations between internal pyridine signals (9.20 ppm and 9.08 ppm), but no interaction to the termini, with 'gaps' highlighted in red.

Cage $Pd_3L^{4,R}_4(BF_4)_6$

We have assigned cage **$Pd_3L^{4,R}_4$**$(BF_4)_6$ as an inextricable mixture Pd_3L_4 isomers, supported by analysis of the 1D ^{1}H NMR and HRMS data discussed below.

The ^{1}H NMR spectrum of **$Pd_3L^{4,R}_4$**$(BF_4)_6$, is significantly more complex than that of its structural isomer **$Pd_3L^{7,R}_4$**$(BF_4)_6$. Despite this, the HRMS shows clean formation of Pd_3L_4 cage species (Figure S60) and is remarkably similar to that obtained for **7**$(BF_4)_6$, (Figure S49). This suggests that the complexity we observe in the NMR spectrum of **$Pd_3L^{4,R}_4$**$(BF_4)_6$ is due to cage structural isomerism, rather than partial complex formation.

The simplest way to identify the isomerism within a system is to consider the number of ligand environments present. As with our previous systems, the *tert*-butyl protons provide a good handle for this, as there is only one *tert*-butyl group per ligand environment. Therefore, each signal corresponds to a different ligand environment. For **$Pd_3L^{4,R}_4$**$(BF_4)_6$, we clearly resolve eight major *tert*-butyl signals, indicative of eight different ligand environments (Figure S184).

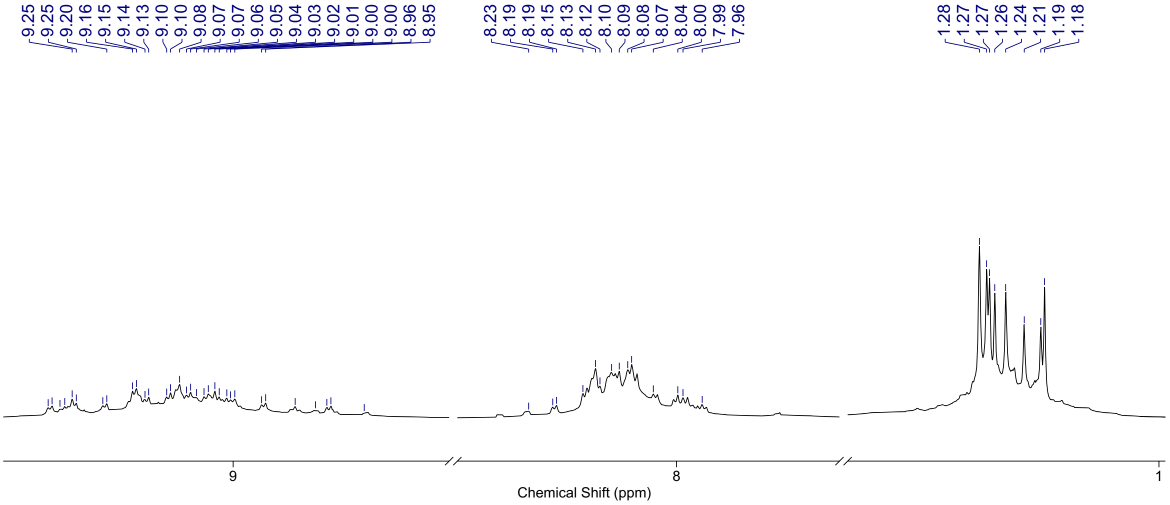

Figure S184. Partial 950 MHz ^{1}H NMR spectrum of $\mathbf{Pd_3L^{4,\mathit{R}}_4}(BF_4)_6$, showing closer detail on the eight major *tert*-butyl peaks, and the complexity of the aromatics.

To observe this number of ligand environments, all four cage isomers must be simultaneously present (one environment for the 'All Up' *CCCC*, one for the *trans CNCN*, two for the *cis CCNN* and four for the 'Three Up' *CCCN*). As such, we assign the complexity of the ^{1}H NMR to an inextricable mixture of all cage isomer peaks, and thus a total lack of isomer selectivity.

In both of the following examples, the stereocentres directly attached to the metal coordinating units have been inverted relative to the above examples. We believed that this inversion from 4*R* to 4*S* substitution would alter the direction of the metal coordinating vectors and thus influence the isomer selectivity. However, the introduction of unnatural 4*S*-Hyp residues into ligands $\mathbf{L^{7,\mathit{S}}}$ and $\mathbf{L^{4,\mathit{S}}}$ is also expected to have a destabilising effect on the PPII structure, which was observed in both the ^{1}H NMR (Figures S185 + S186) and CD (Figure S168) spectra of these ligands. As such, we expected that both factors would influence the self-assembly of these ligands and the observed isomer selectivity.

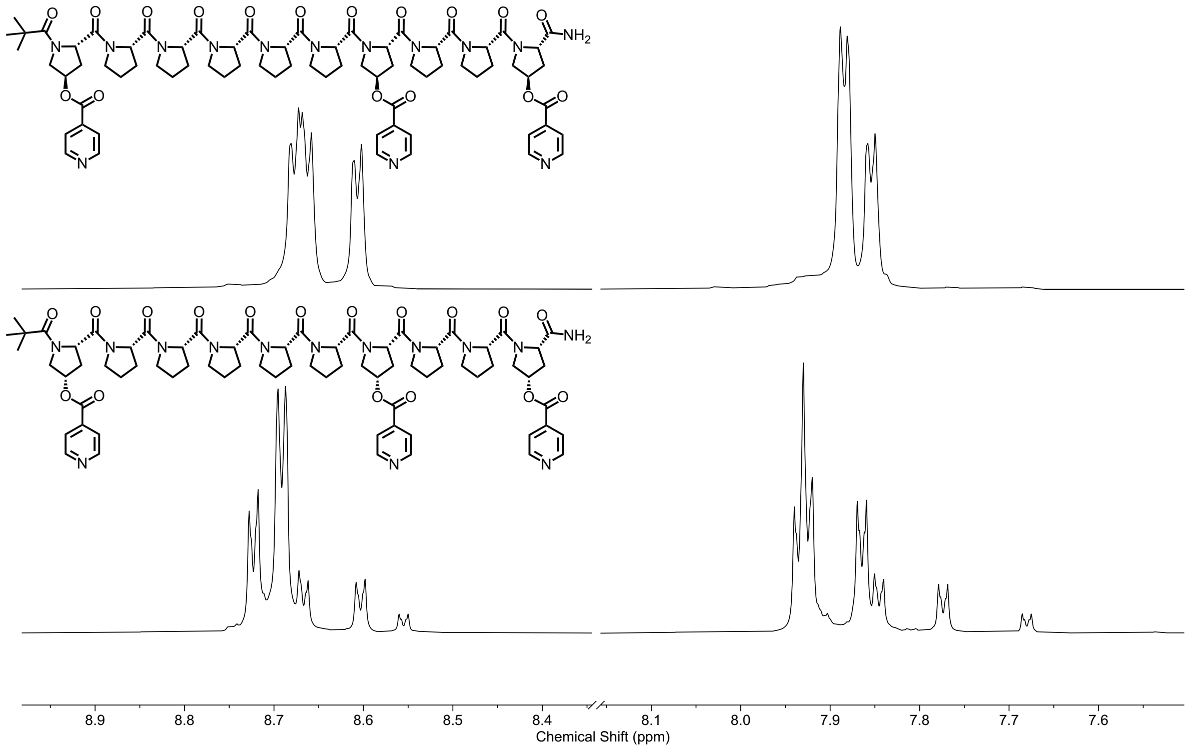

Figure S185. Partial stacked 600 MHz ^{1}H NMR spectrum of diastereoisomers $\mathbf{L}^{7,R}$ (top), and $\mathbf{L}^{7,S}$ (bottom), showing minor species due to increased PPI character in $\mathbf{L}^{7,S}$.

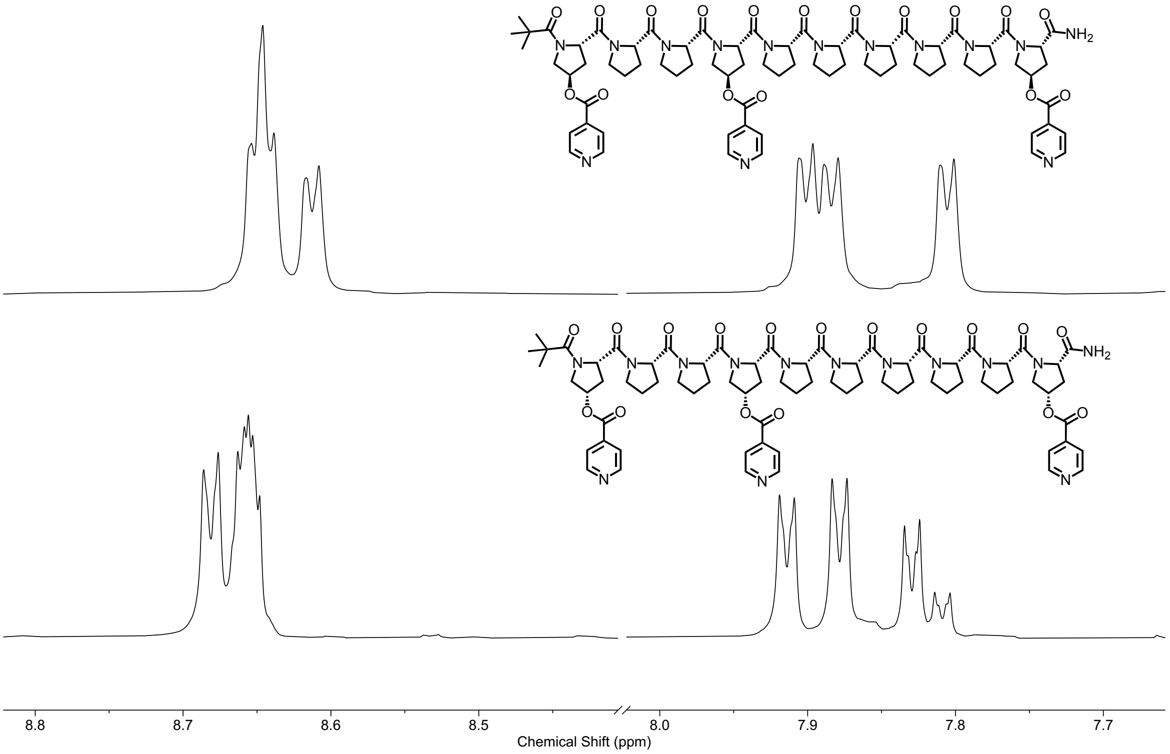

Figure S186. Partial stacked 600 MHz ^{1}H NMR spectrum of diastereoisomers $\mathbf{L}^{4,R}$ (top), and $\mathbf{L}^{4,S}$ (bottom), showing minor species due to increased PPI character in $\mathbf{L}^{4,S}$.

Cage [$Pd_3L^{7,S}_4(BF_4)_6$]$_2$

We have assigned cage [$\mathbf{Pd_3L^{7,S}_4}(BF_4)_6$]$_2$ as a Pd_6L_8 interpenetrated complex, supported by analysis of the 1D and 2D NMR, and HRMS data discussed below (Figure S187).

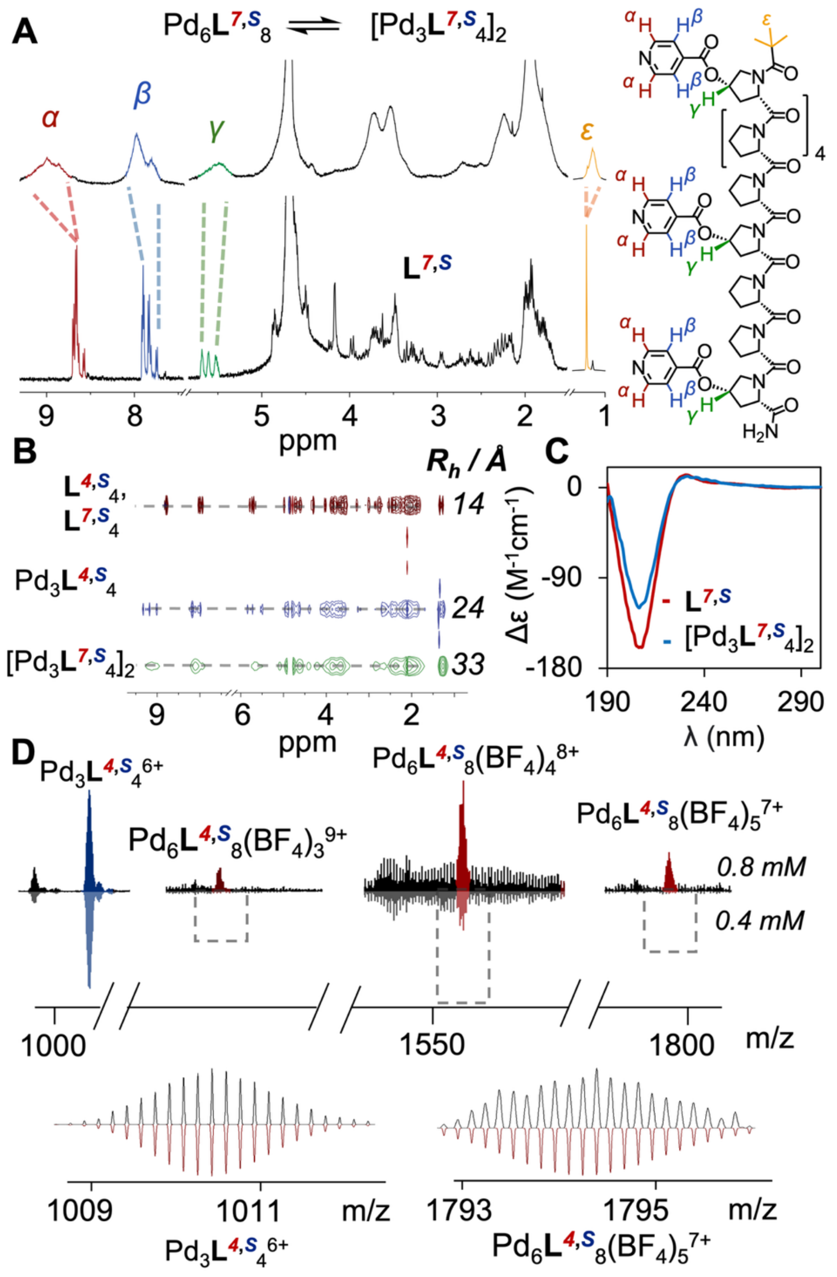

Figure S187. Summary of data supporting assignment of ($\mathbf{Pd_3L^{7,S}_4}$)$_2$ cage **A**. ^{1}H NMR (600 MHz, D_2O, 298 K) of interlocked cage ($\mathbf{Pd_3L^{7,S}_4}$)$_2$ (top) and ligand $\mathbf{L^{7,S}_4}$ (bottom). **B**. ^{1}H DOSY NMR (600 MHz, D_2O, 298 K) of ligands $\mathbf{L^{7,S}_4}$, $\mathbf{L^{4,S}_4}$ (red) and cages ($\mathbf{Pd_3L^{7,S}_4}$)$_2$ (green) and $\mathbf{Pd_3L^{4,S}_4}$ (blue), with hydrodynamic radii shown. **C**. Circular dichroism of ligand $\mathbf{L^{7,S}_4}$ (red) and cage ($\mathbf{Pd_3L^{7,S}_4}$)$_2$ (blue) showing significant loss of PPII structure on assembly. **D**. ESI-HRMS data of ($\mathbf{Pd_3L^{7,S}_4}$)$_2$, showing disassembly of interlocked Pd_6L_8 species on dilution, whilst Pd_3L_4 cage $\mathbf{Pd_3L^{7,S}_4}$ remains upon dilution. Isotopic distributions of peaks in ESI-HRMS (recorded top, simulated bottom) for both $\mathbf{Pd_3L^{7,S}_4}$ and ($\mathbf{Pd_3L^{7,S}_4}$)$_2$.

As the ^{1}H NMR spectrum of [$\mathbf{Pd_3L^{7,S}_4}$(BF_4)$_6$]$_2$ is very broad, this provides us with little information regarding the isomerism present in this system; however, DOSY NMR indicates the formation of a single species with a larger hydrodynamic radius than the other cage species discussed within this work (Figure S67). Additionally, HRMS confirms the presence of a Pd_6L_8 species (Figures S72 – S77). We thus hypothesise that [$\mathbf{Pd_3L^{7,S}_4}$(BF_4)$_6$]$_2$ is an interpenetrated structure formed by the interlocking of two Pd_3L_4 cages. This is supported by DOSY and dilution studies, in which an increase in the proportion of Pd_3L_4 species in the equilibrium is observed upon dilution (Figures S121 + S187D). While Pd_3L_4 cage isomerism is limited to the four major orientations discussed above, there are numerous ways in which interpenetrated species can form, giving rise to a very large number of possible isomers. For example, each single isomer has 4 possible 'partner' cages (*CCCC*/*CCCC*, *CCCC*/*CCCN*, *CCCC*/*CCNN*, *CCCC*/*CNCN*, etc…), giving 10 possible combinations. Further complexity is added as our Pd_3L_4 systems can form both hetero and homoleptic cavities, thus increasing the possible number of combinations. We must also account for the manner of interlocking i.e. entirely interlocked on all four ligands positions, semi-interlocked etc.

As such, it is impossible to discern the exact manner of interlocking of [$\mathbf{Pd_3L^{7,S}_4}$(BF_4)$_6$]$_2$ from the available data; however, we were able to probe the self-assembly of this species further by monitoring its formation with ^{1}H NMR and HRMS. In the ^{1}H NMR of the intermediate species, $\mathbf{Pd_2L^{7,S}_4}$(BF_4)$_4$ we observe four aromatic H_a pyridyl peaks, i.e. two ligand environments and so a two-fold desymmetrisation, shifted to a higher ppm (Figure S188). This is consistent with the formation of a *cis CCNN* Pd_2L_4 cage species.

We observe four rather than six H_α signals as the central moieties are not coordinated, and so are overlapped with the starting material. Intermediate HRMS data confirms the presence of Pd_2L_4 (Figures S99 + S100).

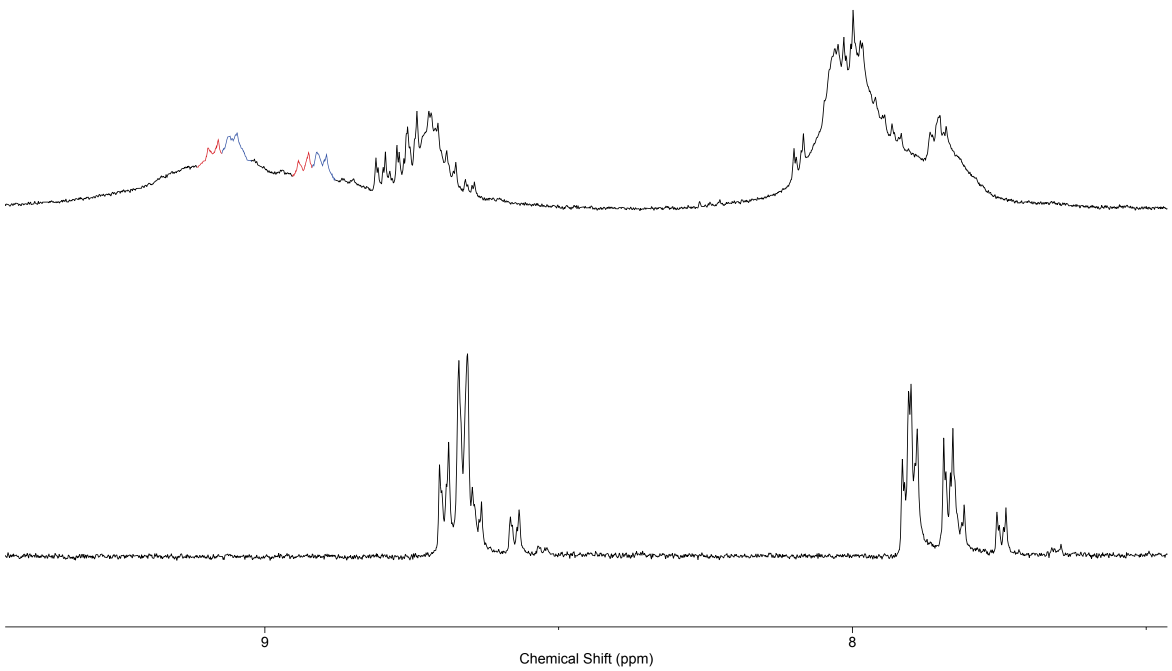

Figure S188. Partial ^{1}H NMR spectra of ligand $\mathbf{L}^{7,S}$ (bottom) and partial cage assembly (top). Intermediate peaks of $\mathbf{Pd_2L^{7,S}_4}(BF_4)_4$ highlighted in red and blue correspond to partial Pd_2L_4, *cis* cage formation.

On occupying the final binding site, so creating a Pd_3L_4 stoichiometry, we hypothesise that deformation is required to align the central binding moieties, favouring the formation of an interpenetrated complex. This is in contrast to ligand $\mathbf{L}^{7,R}$, which forms the Pd_3L_4 cage even at low Pd stoichiometries (Figure S189).

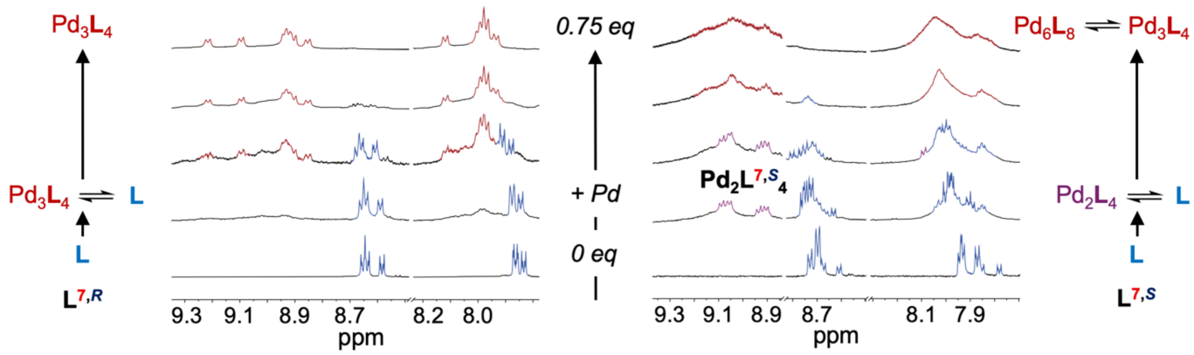

Figure S189. Partial ^{1}H NMR titration (600 MHz, D_2O, 298 K) of $\mathbf{L}^{7,R}$ (left) and $\mathbf{L}^{7,S}$ (right) with $Pd(CH_3CN)_4(BF_4)_2$, showing direct assembly of final Pd_3L_4 species $\mathbf{Pd_3L^{7,R}_4}(BF_4)_6$ for $\mathbf{L}^{7,R}$, but initial formation of a Pd_2L_4 intermediate species $\mathbf{Pd_2L^{7,S}_4}(BF_4)_4$ (highlighted in purple) for $\mathbf{L}^{7,S}$. The intensity of peaks of $\mathbf{Pd_2L^{7,S}_4}(BF_4)_4$ at ~9.0 ppm are scaled x3 relative to the rest of the spectrum for clarity.

A likely driving force for the formation of interpenetrated cage complexes is to free frustrated solvent molecules from within cage cavities. PPI helices have been reported to have weaker interactions than PPII helices with polar solvents such as water due to the orientation of carbonyl groups along the helix.[S9] Therefore, we hypothesise that the displacement of weak water:ligand interactions with Van der Waals and π-stacking interactions on cage interpenetration leads to the unexpected catenation in this ligand system.

Cage $\textbf{Pd}_3\textbf{L}^{4,S}{}_4(BF_4)_6$

Cage $\textbf{Pd}_3\textbf{L}^{4,S}{}_4(BF_4)_6$ is an 'All Up' *CCCC major isomer*, supported by analysis of the 1D and 2D NMR data discussed below.

This ligand, $\textbf{L}^{4,S}$, is the 'opposite' of our first ligand $\textbf{L}^{4,R}$, both in stereoisomerism and structural isomerism. Akin to cage, $\textbf{Pd}_3\textbf{L}^{7,R}{}_4(BF_4)_6$, $\textbf{Pd}_3\textbf{L}^{4,S}{}_4(BF_4)_6$ also exists as one major, discrete isomer (with slightly less preference/selectivity than $\textbf{Pd}_3\textbf{L}^{7,R}{}_4(BF_4)_6$, as revealed by the increased number of sub-peaks) but forms only one ligand environment, as indicated by the presence of one major *tert*-butyl signal and three H_a signals.

The absence of desymmetrisation upon cage assembly is immediately indicative of either the *trans CNCN* or 'All Up' *CCCC* isomer. We believe that the geometric constraints imposed by the design of the ligand would result in *CCCC* 'All-Up' being the more favourable isomer. To evidence this, we provide the below NMR analysis.

In $\textbf{Pd}_3\textbf{L}^{4,S}{}_4(BF_4)_6$ we observe significant distortions to the ligand signals that we have not been observed previously. We see highly shielded proline aliphatic peaks, and additional NOE interactions between pyridines that are absent in other species.

In general, the NOESY spectrum of $\textbf{Pd}_3\textbf{L}^{4,S}{}_4(BF_4)_6$ shows significant interactions between the proline backbone and the aromatics, particularly with the α-pyridyl signals which usually have very low levels of interaction (Figure S188).

Previously we have shown, by CD and ^{1}H NMR analysis of a specifically designed ^{19}F tagged cage, that we see little evidence of cage collapse in *cis CCNN* cages;[S3] however, the increased number of correlations that we observe in this case are very suggestive of a level of collapse to some of the internal cavity.

CD spectroscopy (Figure S170) suggests that this collapse occurs due to a significant deviation from an idealised PPII structure upon cage formation, with some form of chiral secondary structure arising.

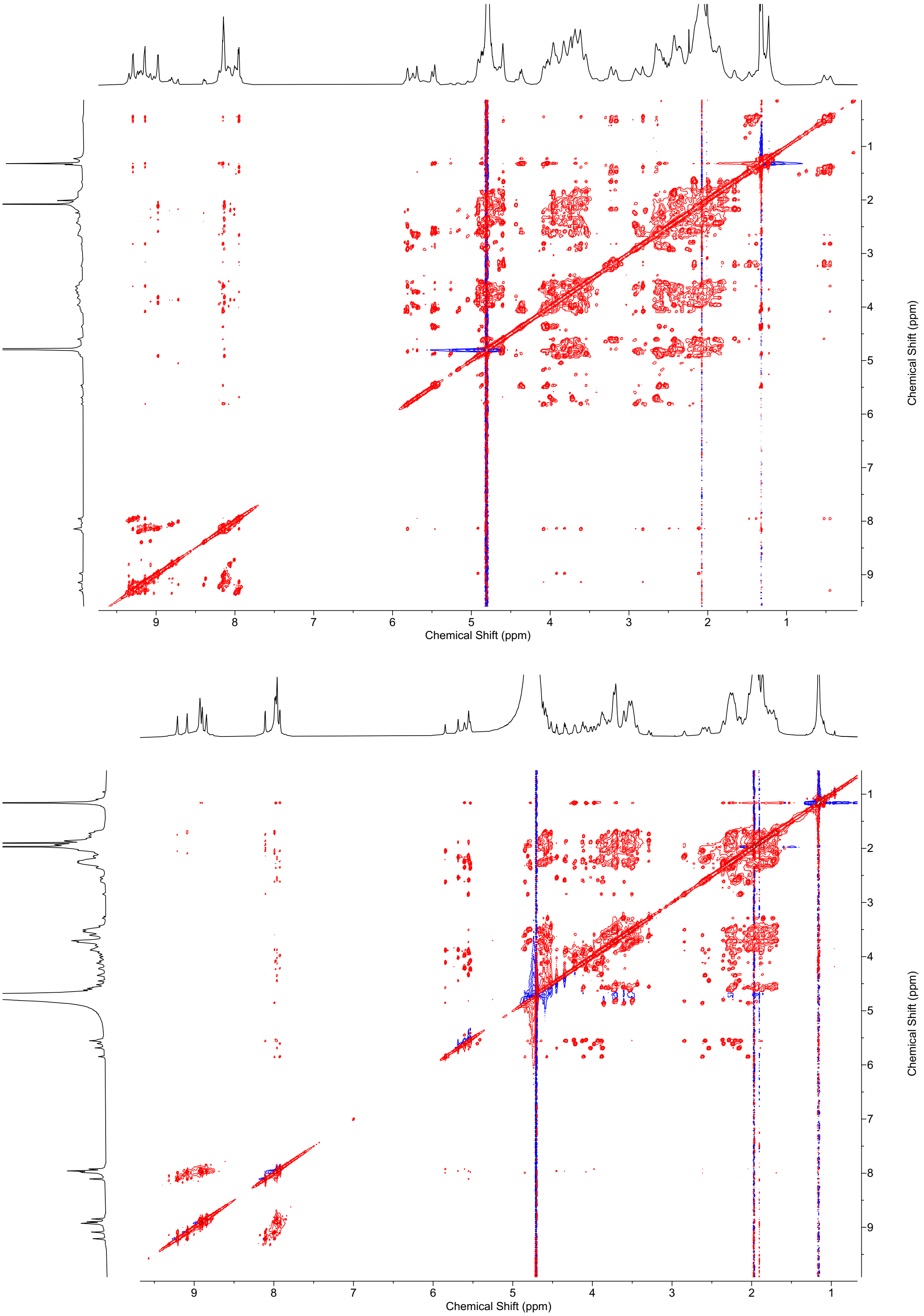

Figure S190. 950 MHz NOESY of **$Pd_3L^{4,S}_4$**$(BF_4)_6$ (top), and NOESY of **$Pd_3L^{7,R}_4$**$(BF_4)_6$ (bottom), showing differences in relative intensity of correlations between aromatics and backbone proline CHs, which are much higher in **$Pd_3L^{4,S}_4$**$(BF_4)_6$.

We hypothesise that cage $\mathbf{Pd_3L^{4,S}_4}(BF_4)_6$ forms an 'All Up' *CCCC* isomer in which the smaller internal cavity is "collapsed" as a result of this deformation. This is supported by a clear and significant upfield shift (up to ~1.5 ppm) in a select few proline backbone signals, indicating a significant shielding, most likely arising from interactions between these peaks and the aromatic pyridine moieties (Figure S191). These peaks also have NOEs to two of the three aromatic signals (Figure S192).

These likely belong to one of the two prolines between the *N*-terminus and the central binding moiety, which are positioned below the aromatics in a collapsed cage cavity brought about by this cage deformation. The smaller Hyp-Pro-Pro-Hyp section is more likely to collapse as it contains a greater local concentration of 4*S*-Hyp which destabilises PPII.

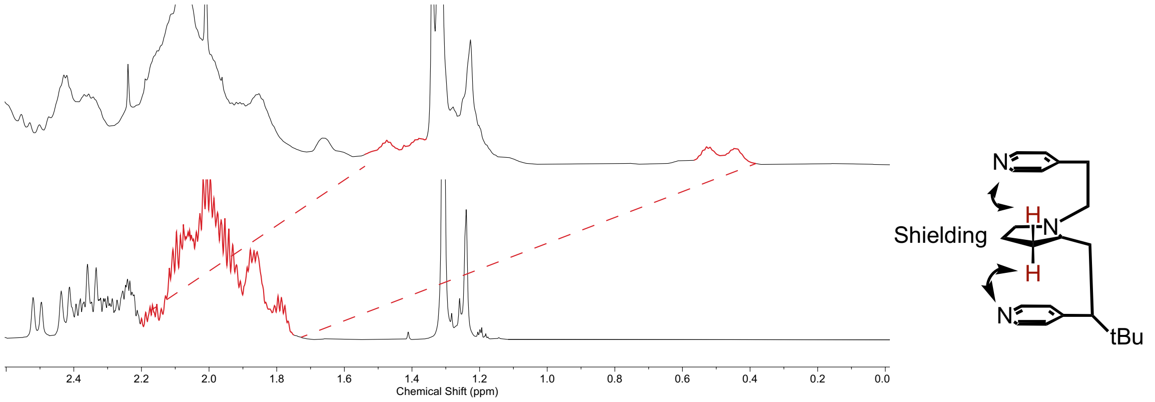

Figure S191. Partial stacked ^{1}H NMR spectra of cage $\mathbf{Pd_3L^{4,S}_4}(BF_4)_6$ (top), and ligand $\mathbf{L^{4,S}}$ (bottom) showing shift of central proline residues, along with representation of shielding stack.

The NOESY spectra show that all the proline peaks that are in close contact with both of these aromatic environments are significantly shielded (Figure S188).

Additionally, there are NOESY interactions between two of three aromatic environments, indicating close proximity. Whilst this could occur in the *trans CNCN* cage, the spectra also show these two aromatic environments have significant interactions to a shared set of peaks belonging to the proline backbone, where the third is an isolated set of signals that interact with its own proline family, but has no other overlap.

This supports the 'All Up' *CCCC* cage where the collapse at the bottom results in some NOE between aromatic signals, but the *C*-terminus remains isolated. If the *trans CNCN* cage had formed, we would expect to see overlap between NOE effects from all three aromatic spin systems, as the *C*- and *N*-termini would be in close proximity. In addition, peaks would result from the *N*-termini being in close contact with the central binding moiety (as it is only 3 residues removed). As we only see two in contact, and one peak with no shared NOE overlap, we can assign this isolated spin system to the *C*-terminus, and the other two to the *N*-terminus and central binding site, confirming that we have formed the 'All Up' *CCCC* isomer.

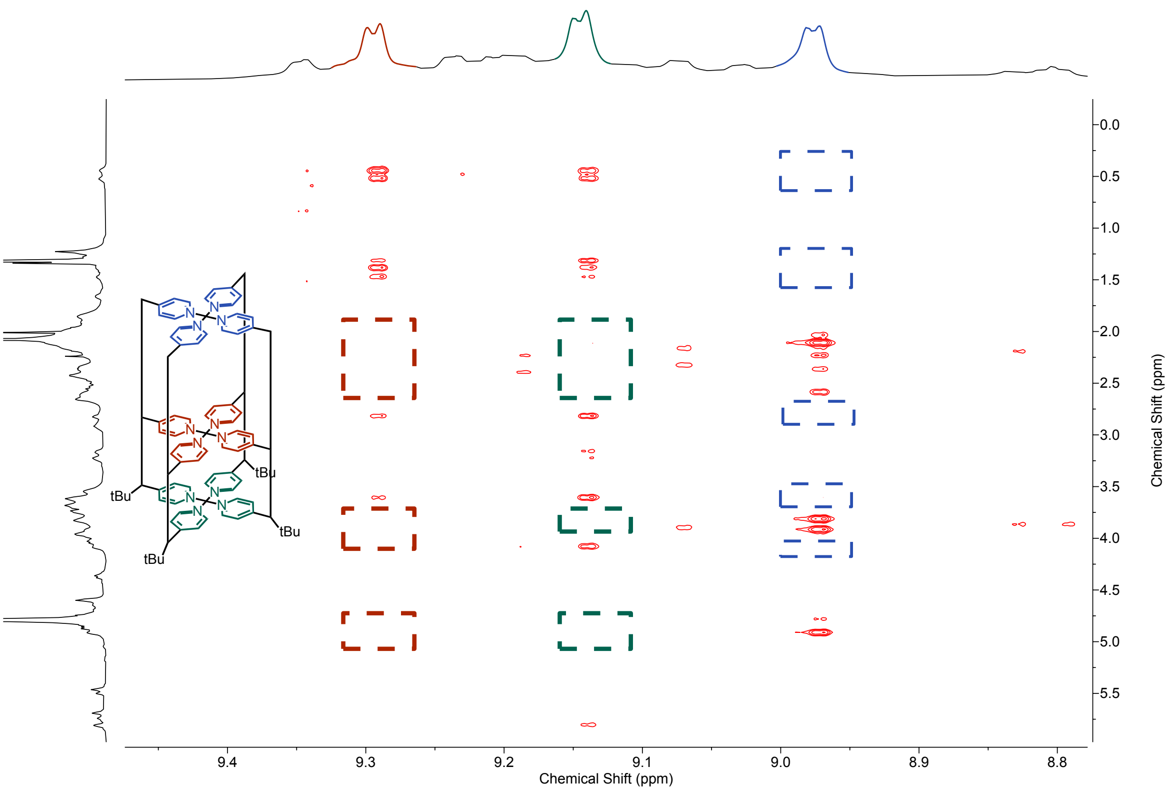

Figure S192. NOE correlations between the middle and *N*-terminal spin systems vs the *C*-terminal spin systems, with missing overlaps highlighted to show that the *C*-terminus is isolated.

Assembly in d_3-MeCN

We have previously reported isomeric control is dependent on solvent. We attribute this dependence to a variation in PPII stability on solvent. As cages **$Pd_3L^{4,R}_4$** and **$Pd_3L^{7,S}_4$** show a tolerance to partial deformation from PPII to PPI structures, we sought to investigate the solvent tolerance of the self-assembly process.

We carried out a cage assembly of **$L^{7,R}$** in d_3-MeCN, a common solvent in metal-organic cage self-assembly and one which we previously observed an increase in PPI structure by CD spectroscopy.[S3]

We observe minor species due to increased PPI character in ^{1}H NMR of **$L^{7,R}$** in d_3-MeCN (Figures S193 and S194) and, upon addition of 0.75 eq. $Pd(CH_3CN)_4(BF4)_2$, a broad ^{1}H NMR spectrum for which no significant formation of discrete cage species were observed by ESI-HRMS (Figures S195 and S196). This suggests that the stability of the PPII structure in H_2O is required for cage-assembly and resultant isomer control.

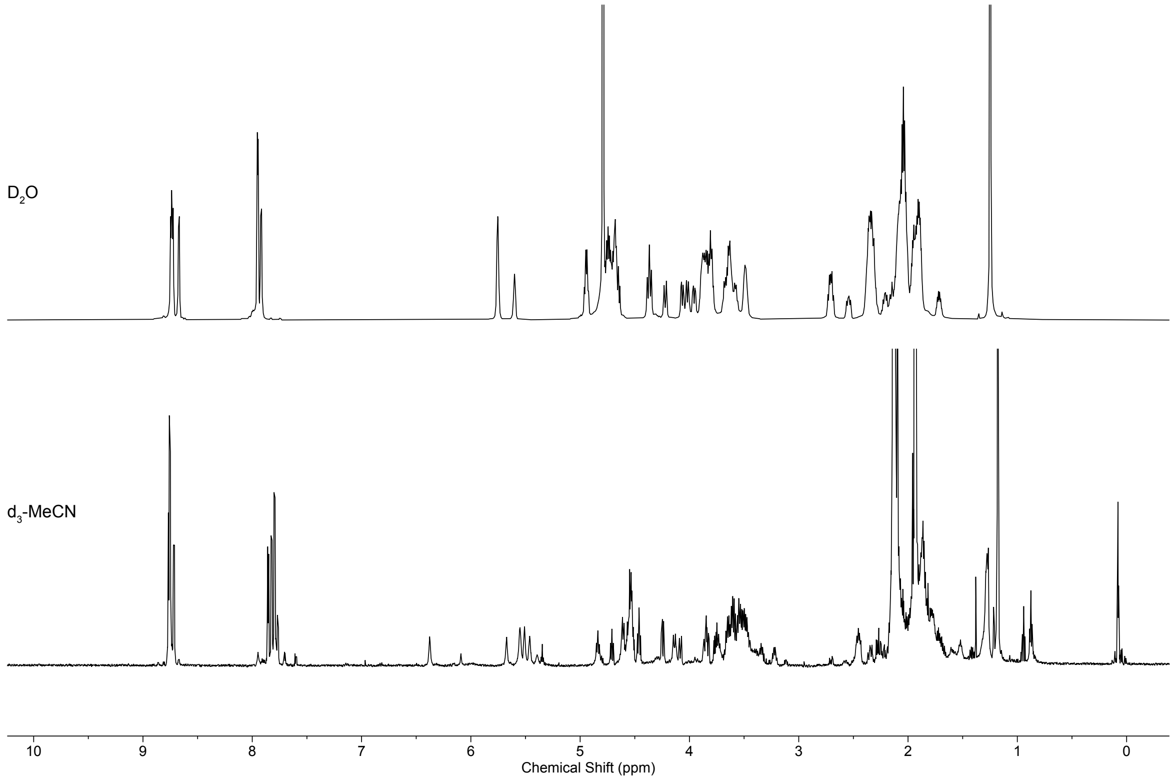

Figure S193. Stacked ^{1}H NMR (600 MHz, 298 K) spectrum of **$L^{7,R}$** in D_2O (top) and d_3-MeCN (bottom), showing minor species due to increased PPI character in d_3-MeCN.

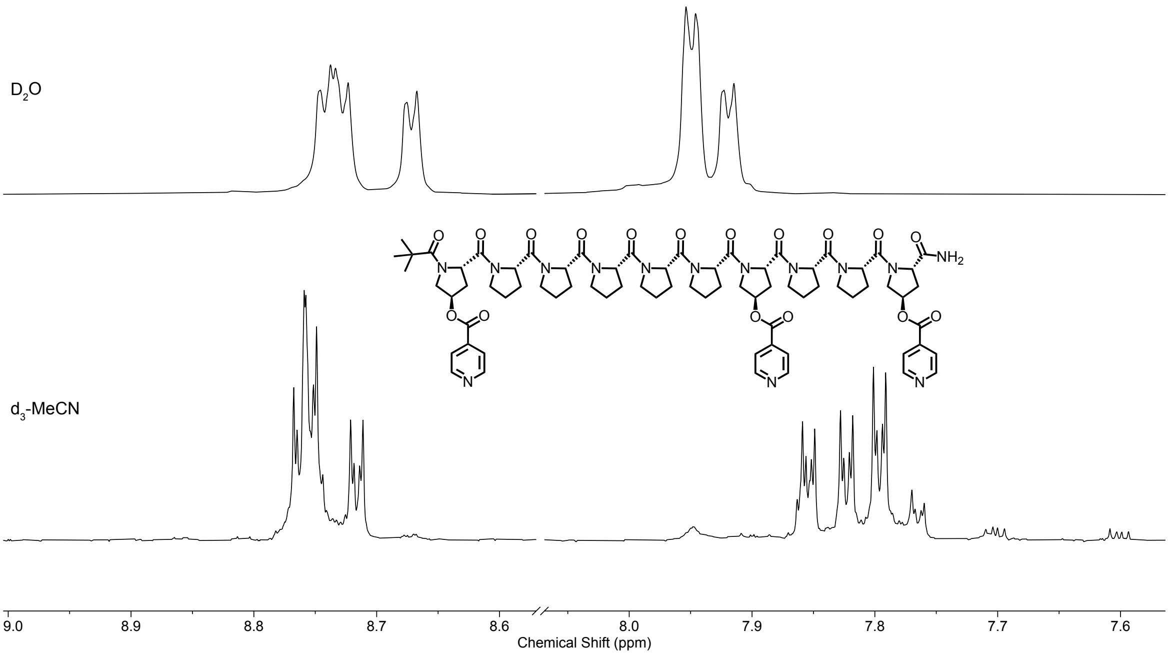

Figure S194. Partial stacked ^{1}H NMR (600 MHz, 298 K) spectrum of isomer **L**^{7,R} in D_2O (top) and d_3-MeCN (bottom), showing minor species due to increased PPI character in d_3-MeCN.

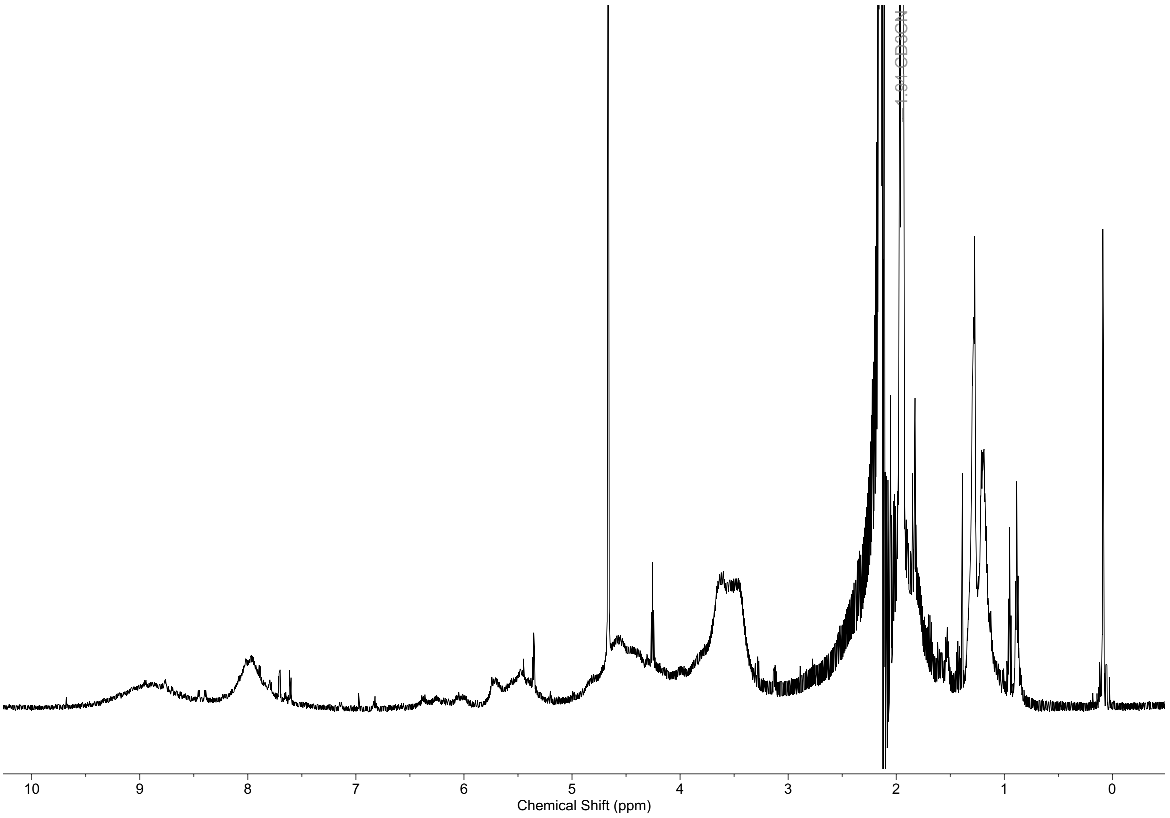

Figure S195. ^{1}H NMR (d_3-MeCN, 600 MHz, 298 K) spectrum of **L**^{7,R} with 0.75 eq. $Pd(CH_3CN)_4(BF_4)_2$.

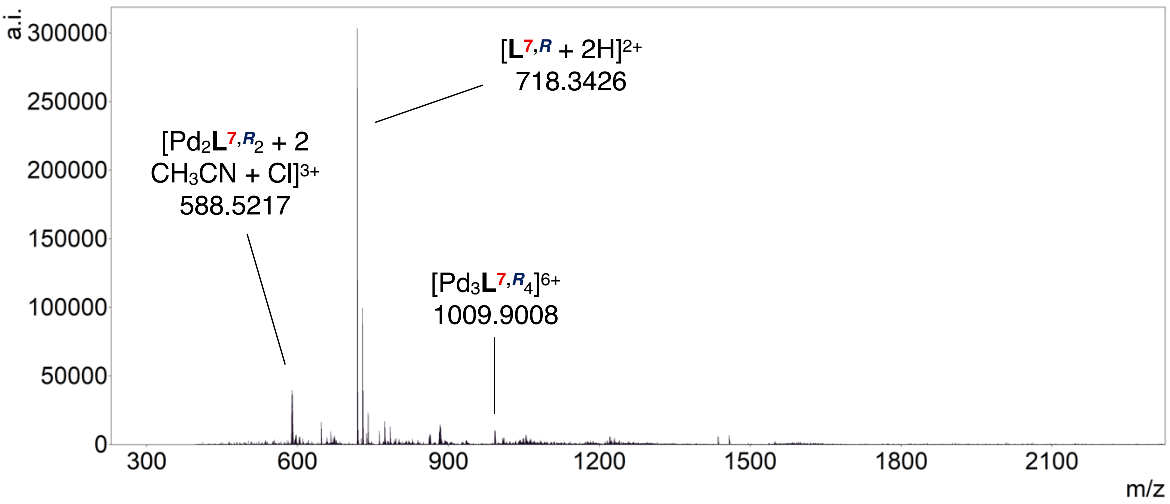

Figure S196. ESI-MS (+ve ion, d_3-MeCN/H_2O) of **2** with 0.75 eq. $Pd(CH_3CN)_4(BF_4)_2$.

Hypothesis for difference in isomer control between $\mathbf{Pd_3L^{7,R}_4}(BF_4)_6$ *and* $\mathbf{Pd_3L^{4,R}_4}(BF_4)_6$

Our previous studies of Pd_2L_4 systems indicated that the *cis CCNN* cage isomer adopted a tilt, where the helical axis of the oligoproline rods was not perpendicular to the pyridine co-ordination vector due to projection of the 4*R* stereocentre from the cyclic proline sidechain (Figure S197). This significantly decreased the energy of the *cis CCNN* relative to the other isomers.

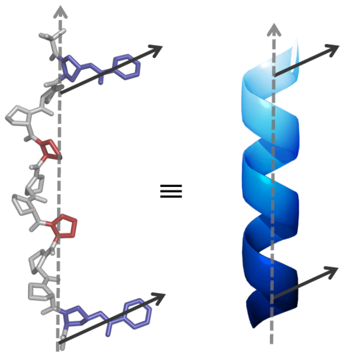

Figure S197. Side view of **1** molecular model (left) and stylised representation (right) showing origin of tilt adopted by ligands within Pd_2L_4 cages. Sites for installation of internal metal co-ordination vectors highlighted in red. Pyridine co-ordination vector represented by black arrows, direction of helical axis represented by grey, dashed arrow.

As the internal metal coordination site is located asymmetrically along the oligoproline backbone, its coordination vector can no longer be parallel to those at the *N*- and *C*-termini in the *cis CCNN* isomer. This prevents the central $PdPyr_4$ plane from lying parallel to the terminal planes in the Pd_3L_4 structure and requires a level of distortion of the internal metal co-ordination unit.

In $\mathbf{L}^{7,R}$, the distortion required is smaller, and in the same direction of the helical axis as already required for the terminal coordination vectors (Figure S198A). In contrast, the distortion in $\mathbf{L}^{4,R}$ is larger and in the opposite direction to the direction of the helical axis and the terminal coordination vectors, which would require significant backbone or metal binding site distortion (Figure S198B).

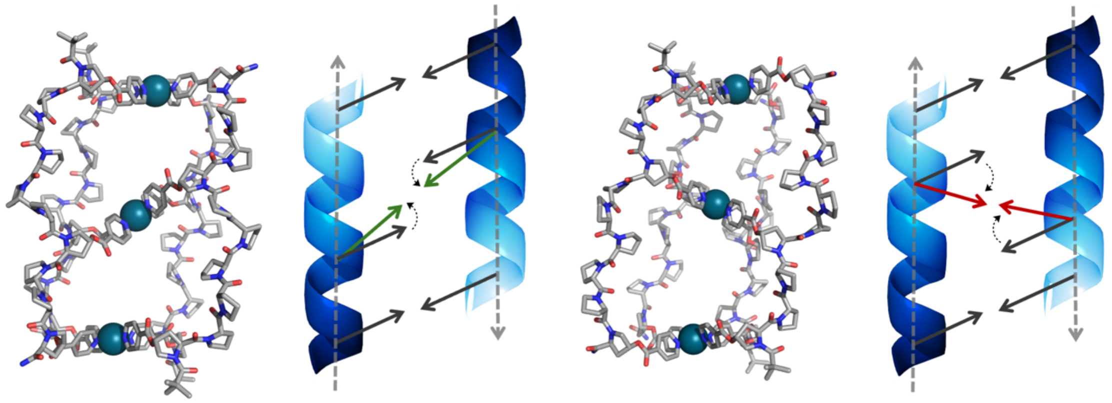

$Pd_3L^{7,R}{}_4$ $(BF_4)_6$ - *CCNN* favored
(Distortion towards direction of Helical Axis)

$Pd_3L^{4,R}{}_4$ $(BF_4)_6$ - *CCNN* disfavored
(Distortion away from direction of Helical Axis)

Figure S198. **A**. Side view of **$Pd_3L^{7,R}{}_4$**$(BF_4)_6$ *cis CCNN* isomer molecular model (left) and stylised representation showing smaller distortions of central co-ordination vector in same direction as terminal co-ordination vectors highlighted in green (right). **B**. Side view of **$Pd_3L^{4,R}{}_4$**$(BF_4)_6$ *cis CCNN* isomer molecular model (left) and stylised representation showing larger distortions of central co-ordination in opposite direction to terminal co-ordination vectors highlighted in red (right).

11. Host-Guest Studies

A stock solution of $\mathbf{Pd_3L^{7,\mathit{R}}_4}(BF_4)_6$ or $\mathbf{Pd_3L^{4,\mathit{S}}_4}(BF_4)_6$ was prepared in D_2O or 10% d_6-DMSO at 0.250 mM. The stock solution contained a small volume of dioxane to act as an internal reference standard. 25 mM - 72 mM stock solutions of guests of interest were prepared in separate vials in D_2O. In each case, 450 µL of cage solution in an NMR tube was titrated with aliquots of 1.5 – 20 µL of guest solution to a typical total of 10 eq. of guest. Where cage precipitation occurred, titrations were stopped. Irinotecan.HCl lacked sufficient solubility in D_2O, and so titrations were carried out with 10% d_6-DMSO in D_2O.

To determine association constants, curve fitting of the experimentally obtained titration isotherms (equivalents of guest vs chemical shift of cage and/or guest proton) was carried using a nonlinear least-squares curve fitting procedure using the online software https://supramolecular.org/, with 1:1 global fitting models (Nelder-Mead method) found to provide the best fit.[S10]

$Pd_3L^{7,R}_4(BF_4)_6$ + Sodium Benzenesulfonate

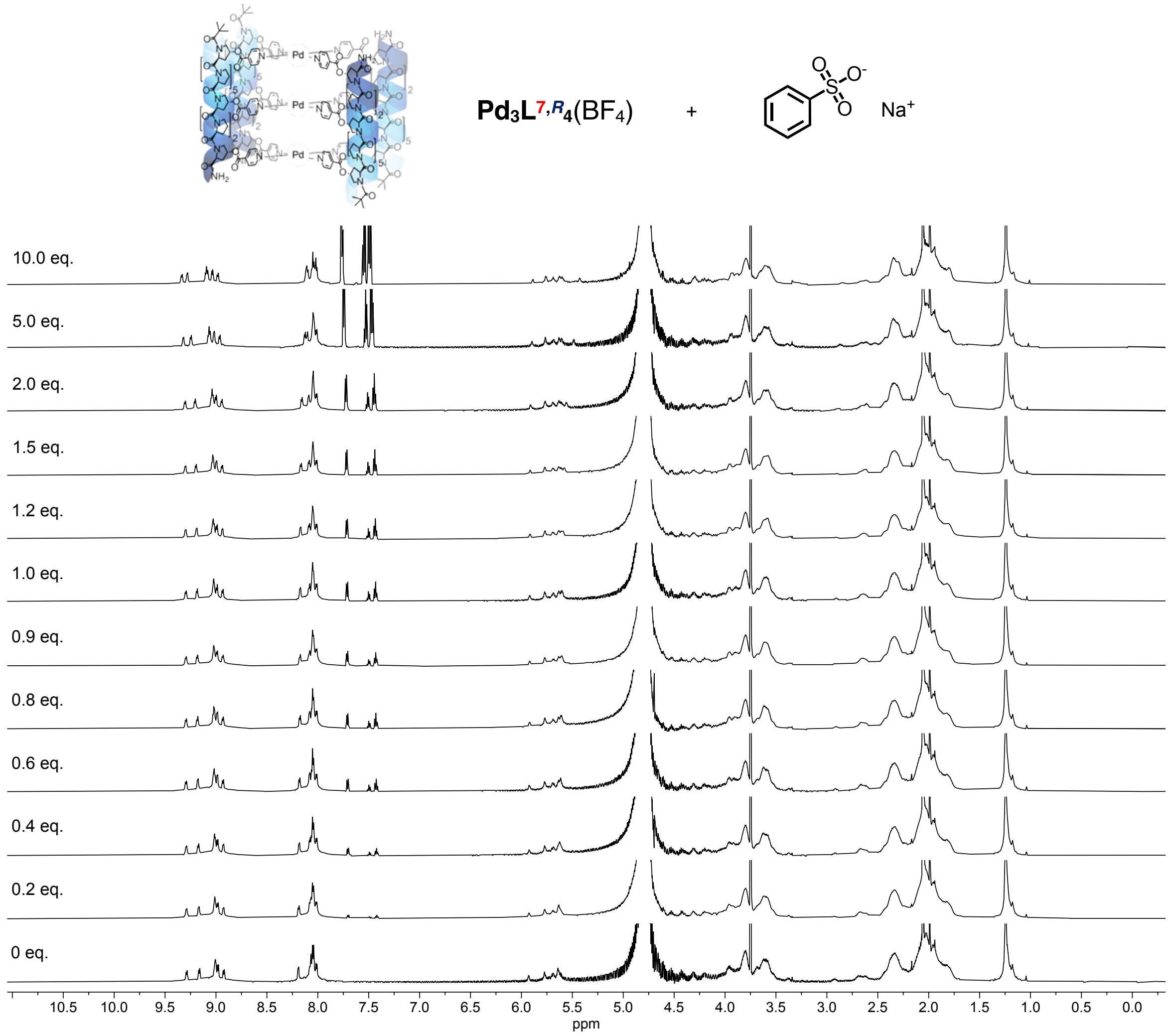

Figure S199. ^1H NMR titration (D_2O, 600 MHz, 298 K) of $\mathbf{Pd_3L^{7,\mathit{R}}_4}(BF_4)_6$ (0.25 mM) and varying equivalents of sodium benzenesulfonate.

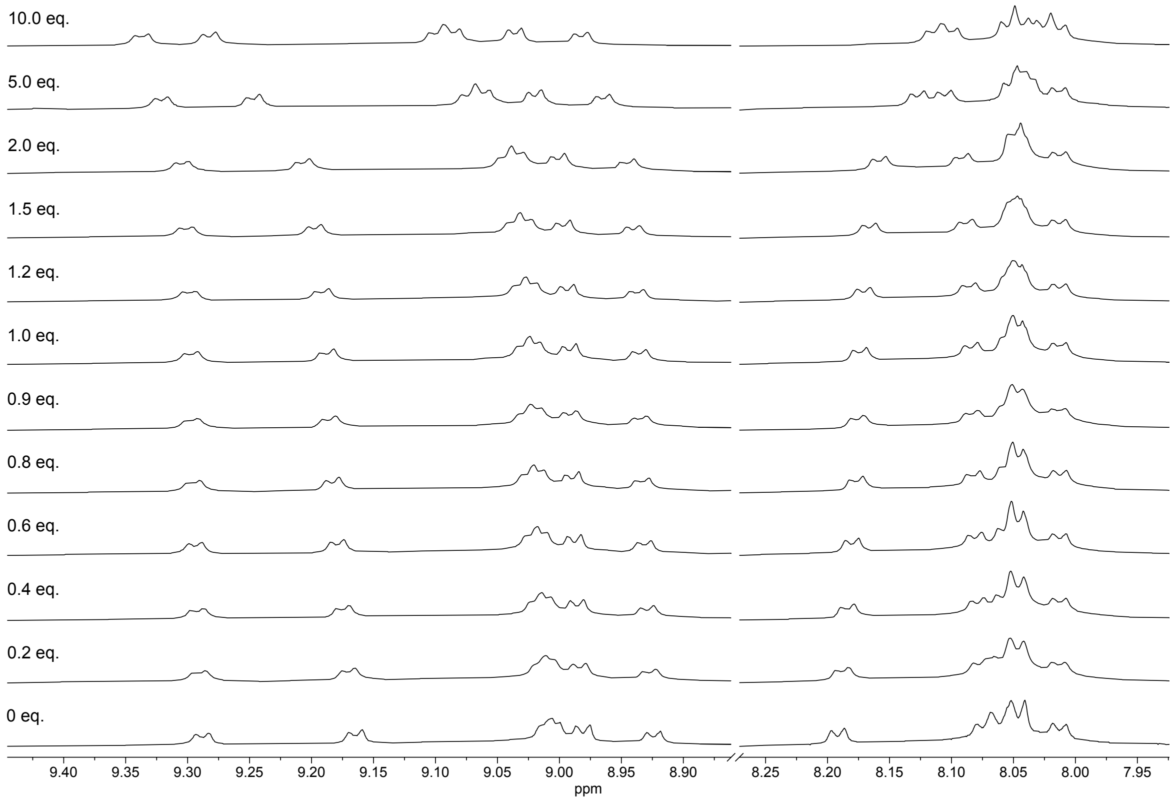

Figure S200. Partial ^1H NMR titration (D_2O, 600 MHz, 298 K) of **$Pd_3L^{7,R}{}_4$**$(BF_4)_6$ (0.25 mM) and varying equivalents of sodium benzenesulfonate.

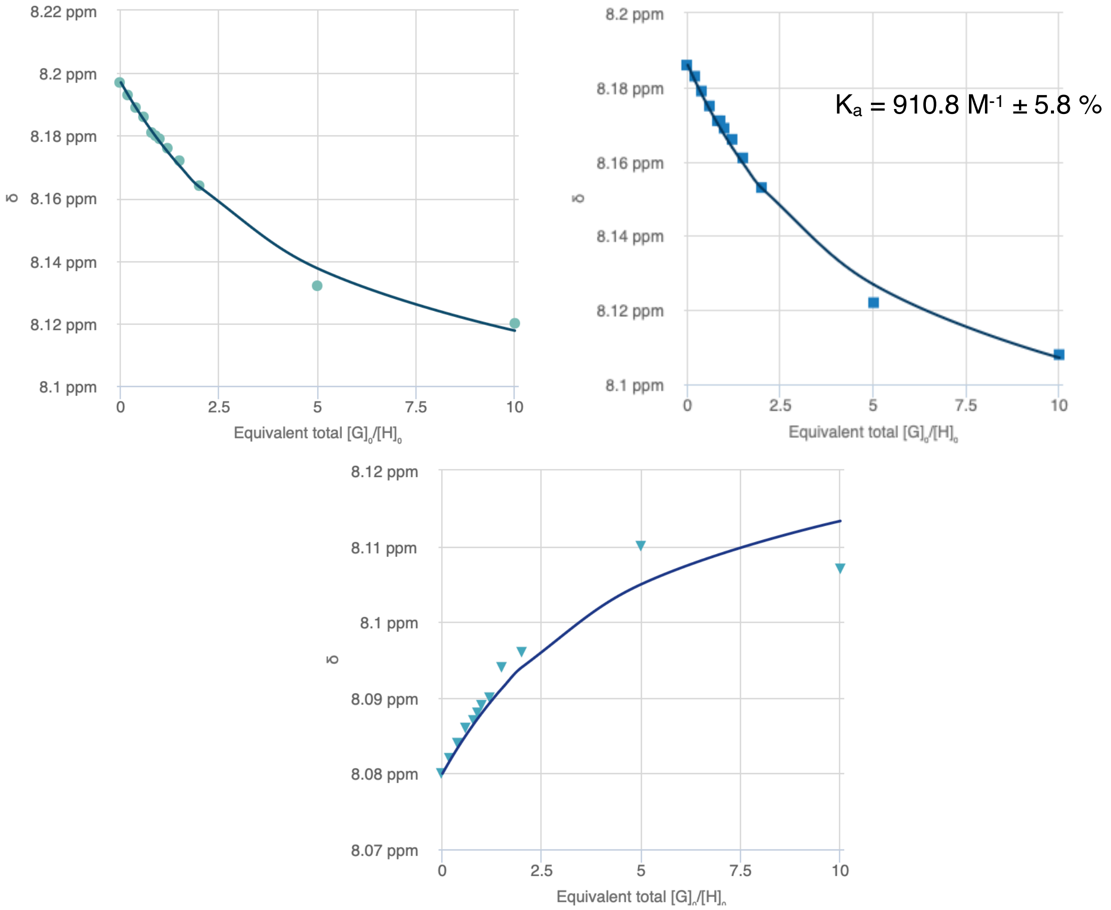

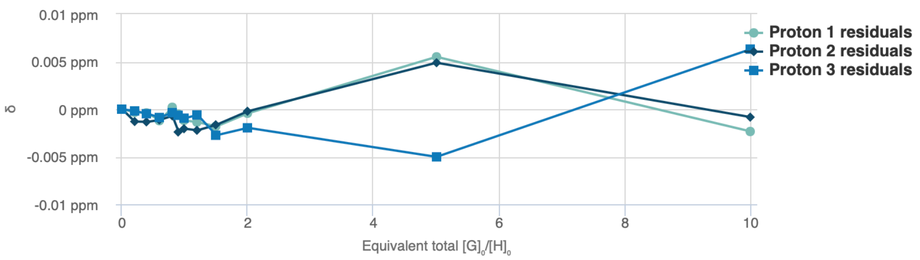

Figure S201. Binding plots for **$Pd_3L^{7,R}_4$**$(BF_4)_6$ and sodium benzenesulfonate. K_a = 910.8 M^{-1} ± 5.8 %.

$Pd_3L^{4,S}_4(BF_4)_6$ + Sodium Benzenesulfonate

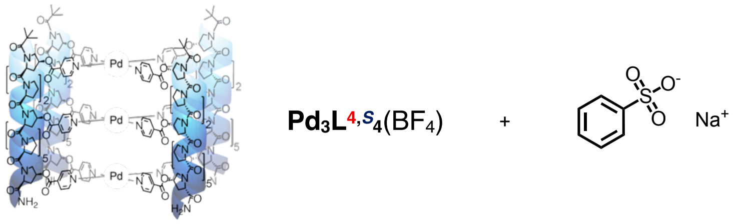

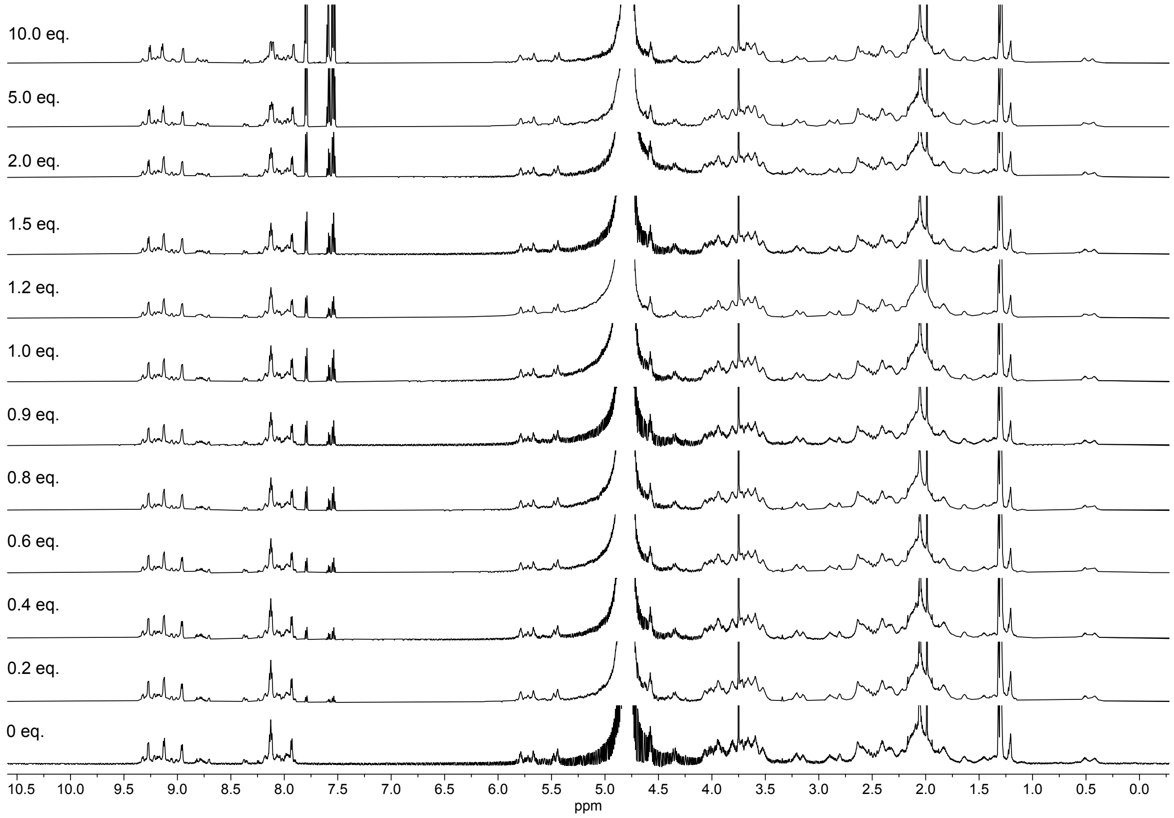

Figure S202. ^{1}H NMR titration (D_2O, 600 MHz, 298 K) of **$Pd_3L^{4,S}_4$**$(BF_4)_6$ (0.25 mM) and varying equivalents of sodium benzenesulfonate.

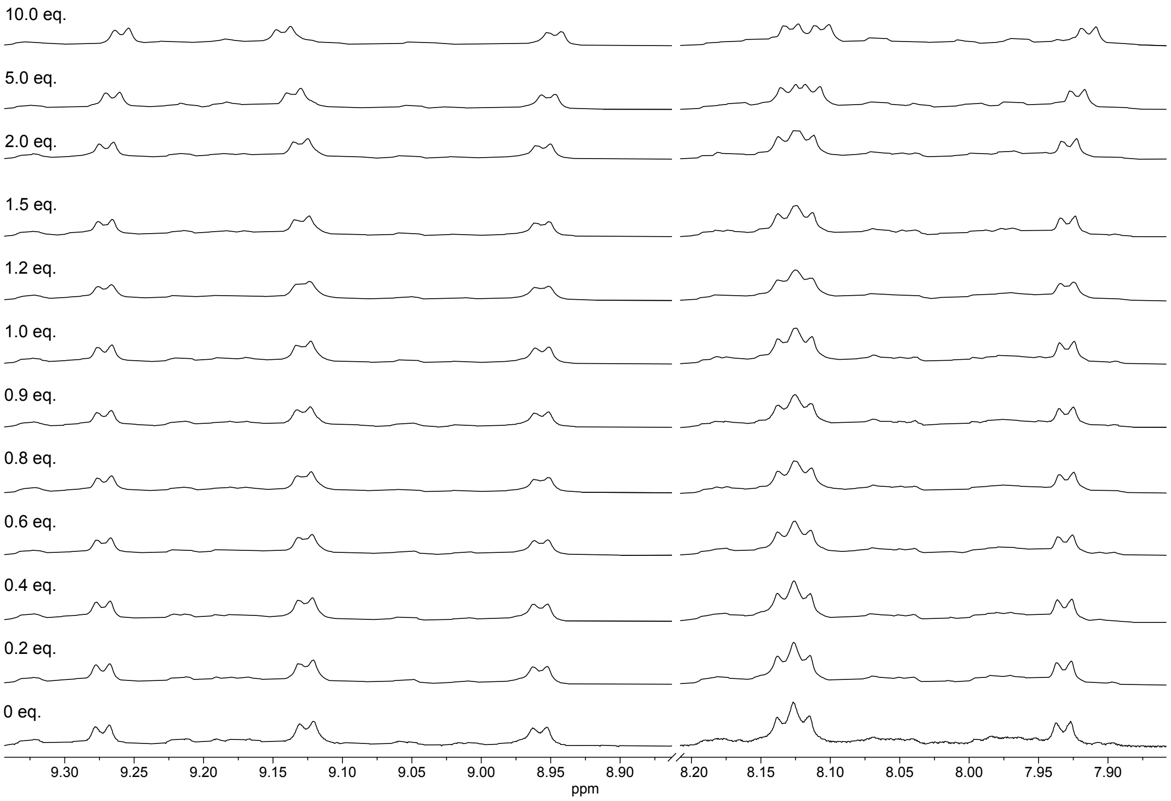

Figure S203. Partial ^{1}H NMR titration (D_2O, 600 MHz, 298 K) of $\mathbf{Pd_3L^{4,\mathit{S}}_4}(BF_4)_6$ (0.25 mM) and varying equivalents of sodium benzenesulfonate.

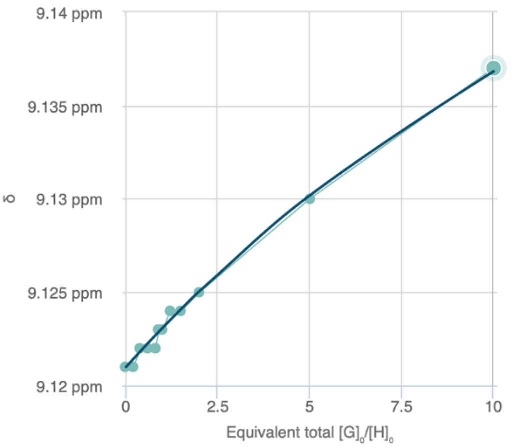

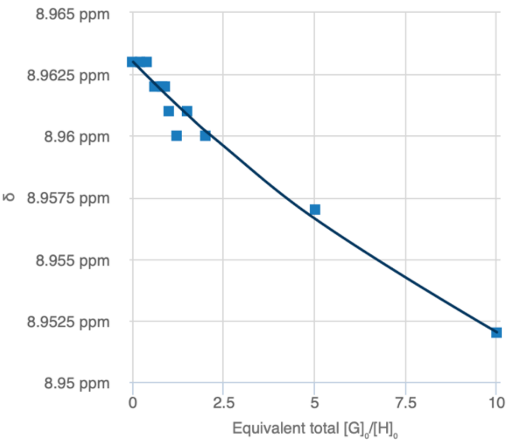

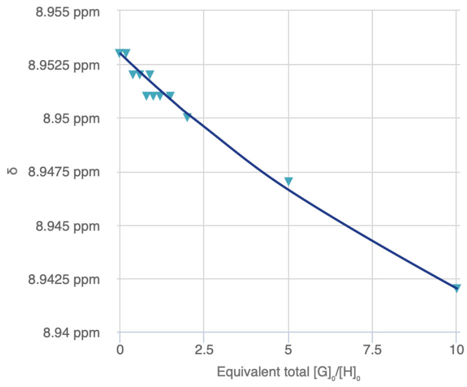

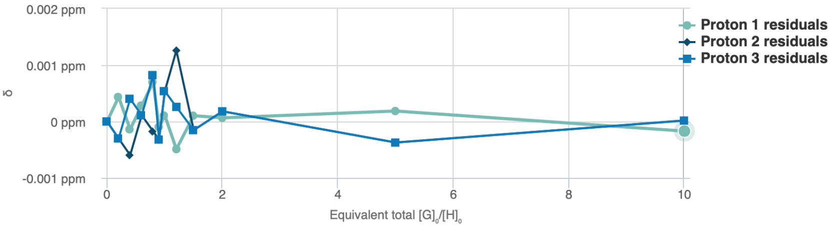

Figure S204. Binding plots for $\mathbf{Pd_3L^{4,S}_4}(BF_4)_6$ and sodium benzenesulfonate. K_a = 119.6 M^{-1} ± 3.8%.

$Pd_3L^{7,R}_4(BF_4)_6$ + Sodium Pyrene-1-sulfonate

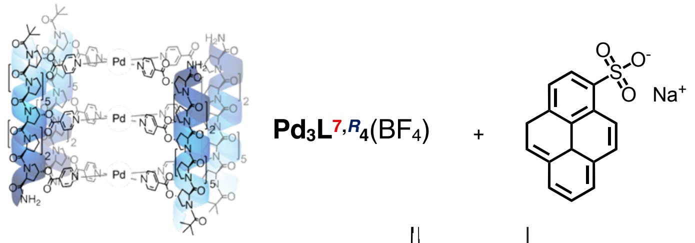

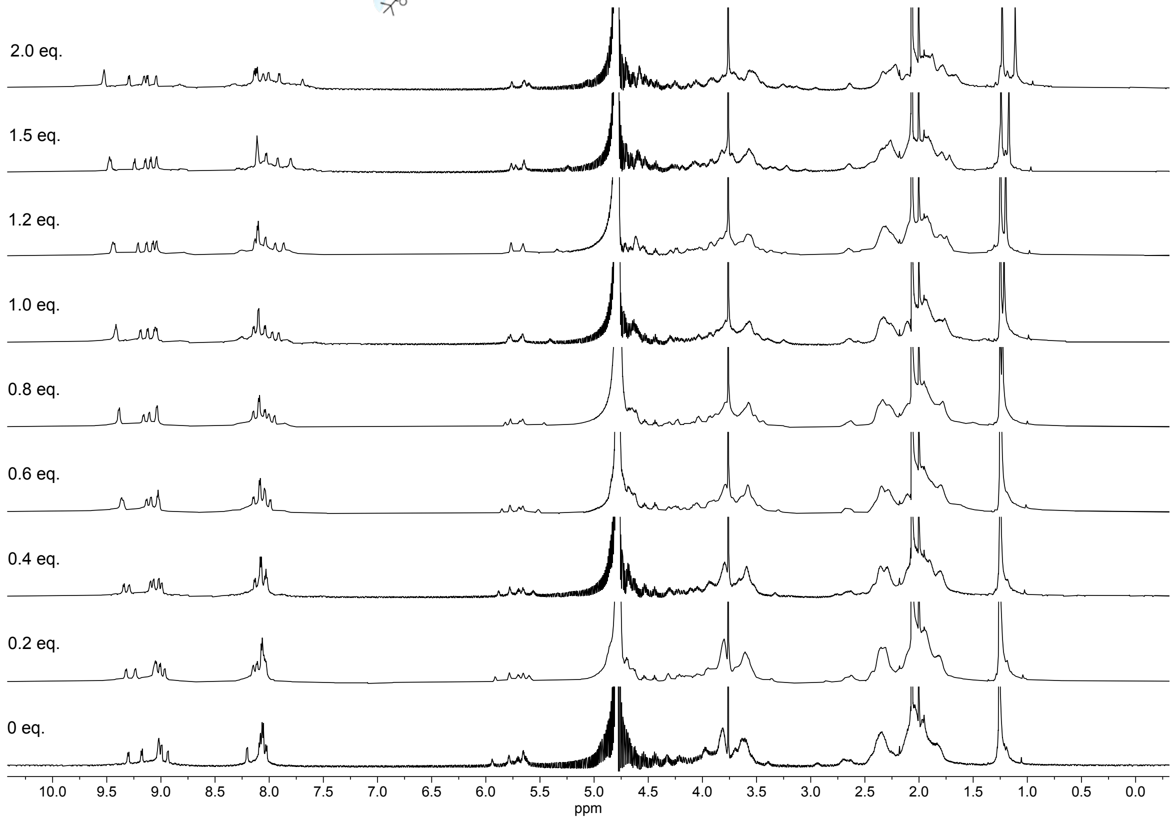

Figure S205. ^1H NMR titration (D_2O, 600 MHz, 298 K) of $\mathbf{Pd_3L^{7,R}_4}(BF_4)_6$ (0.25 mM) and varying equivalents of sodium pyrene-1-sulfonate.

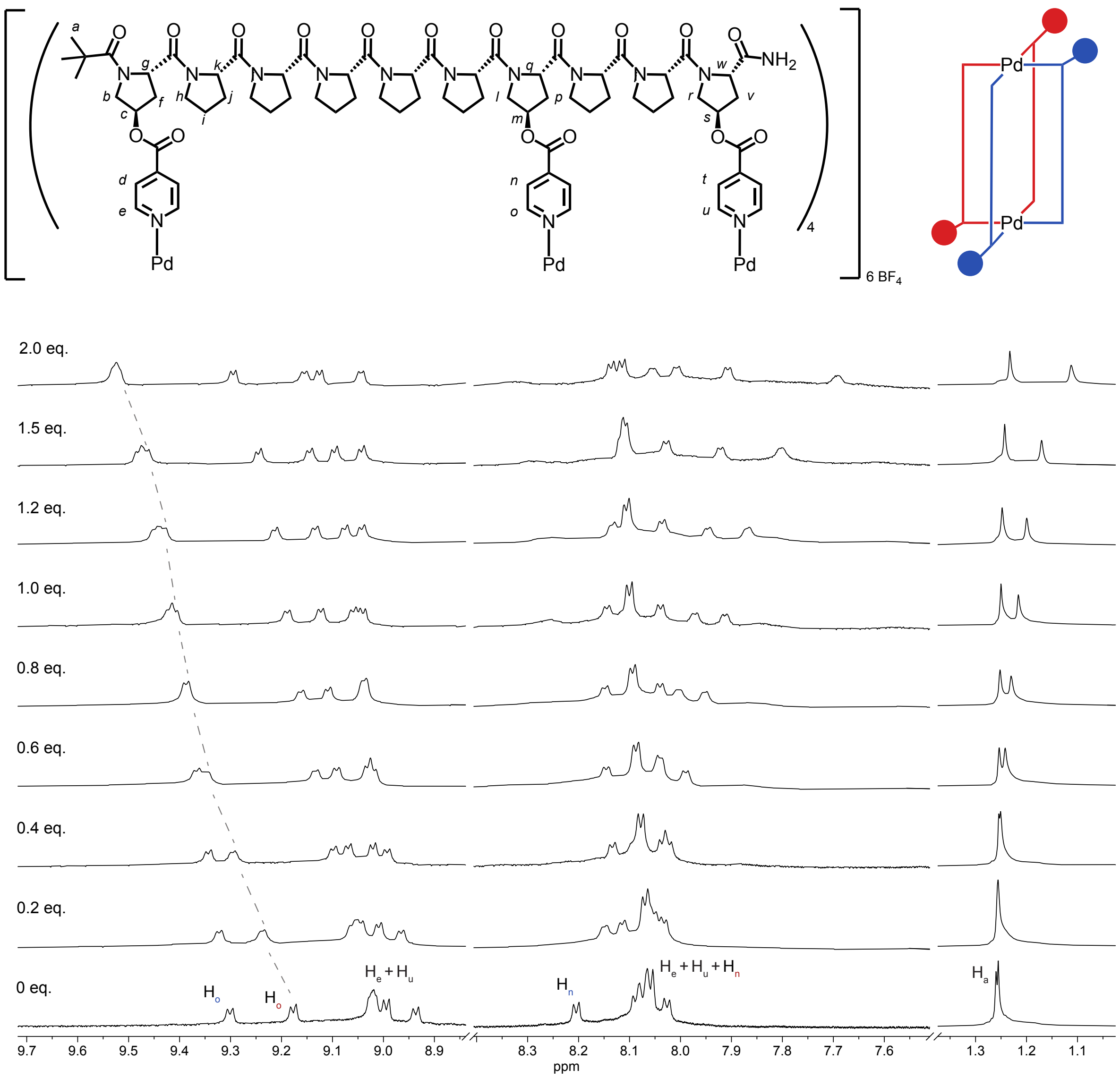

Figure S206. Partial ^{1}H NMR titration (D_2O, 600 MHz, 298 K) of $\mathbf{Pd_3L^{7,R}_4}(BF_4)_6$ (0.25 mM) and varying equivalents of pyrene-1-sulfonate. Peaks at ~1.2 ppm have been scaled independently to the rest of the spectra.

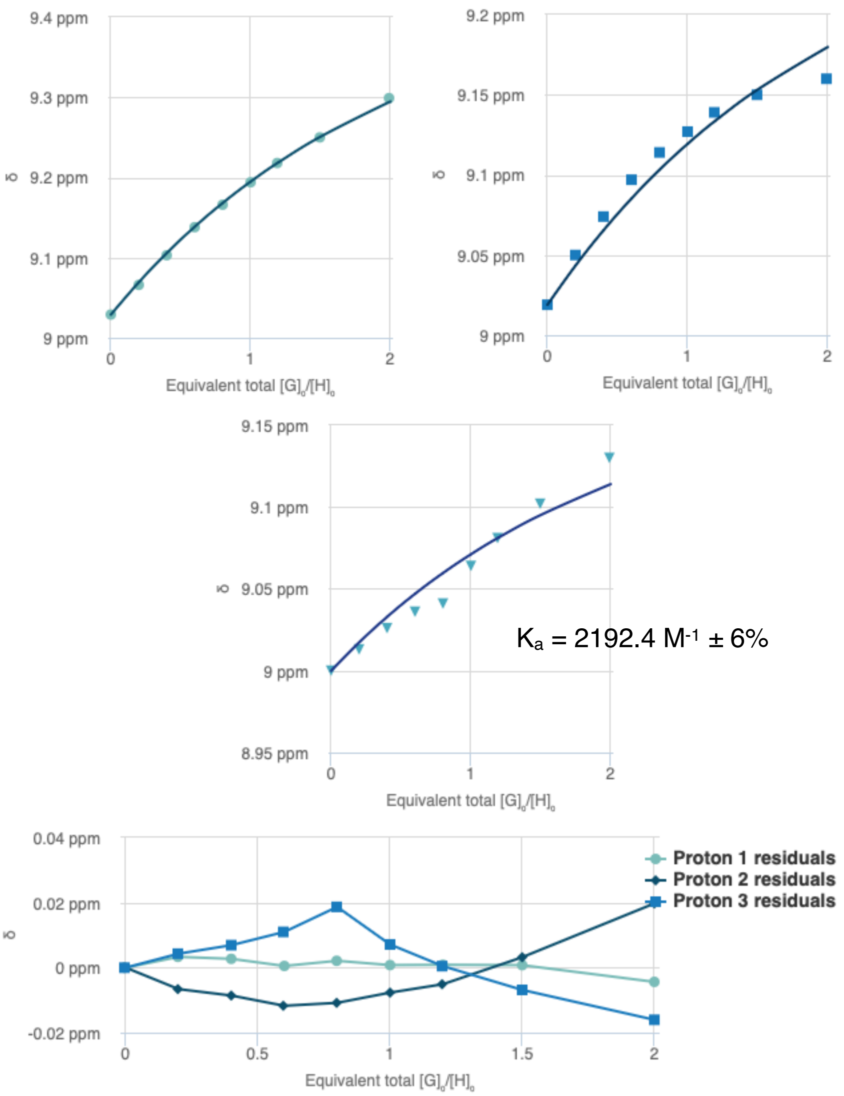

Figure S207. Binding plots for **Pd$_3$L$^{7,R}_4$**(BF$_4$)$_6$ and sodium pyrene-1-sulfonate. K_a = 2192.4 M^{-1} ± 6%.

Pd₃L^{4,S}₄(BF₄)₆ + Sodium Pyrene-1-sulfonate

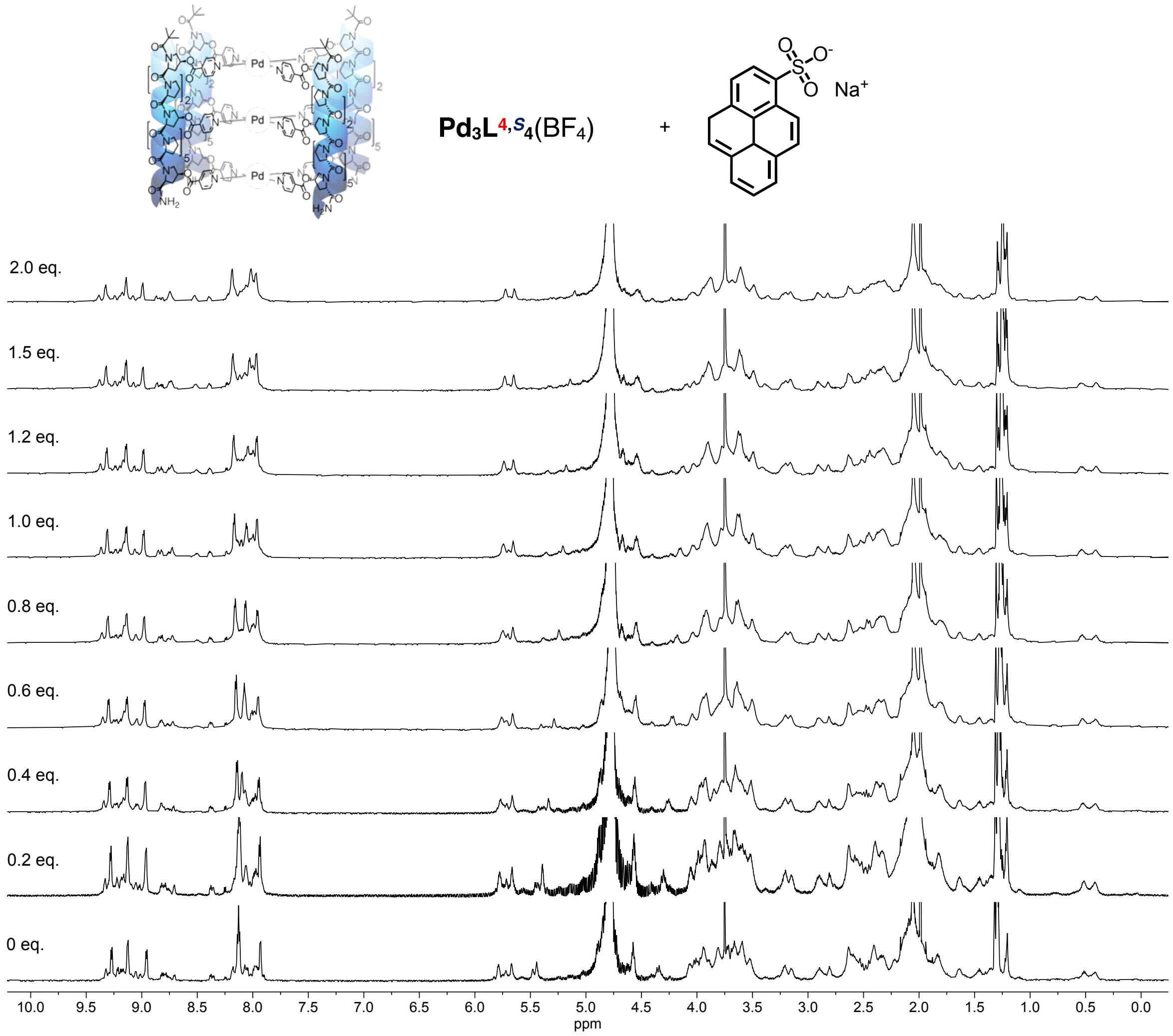

Figure S208. ^{1}H NMR titration (D_2O, 600 MHz, 298 K) of **$Pd_3L^{4,S}_4$**$(BF_4)_6$ (0.25 mM) and varying equivalents of pyrene-1-sulfonate.

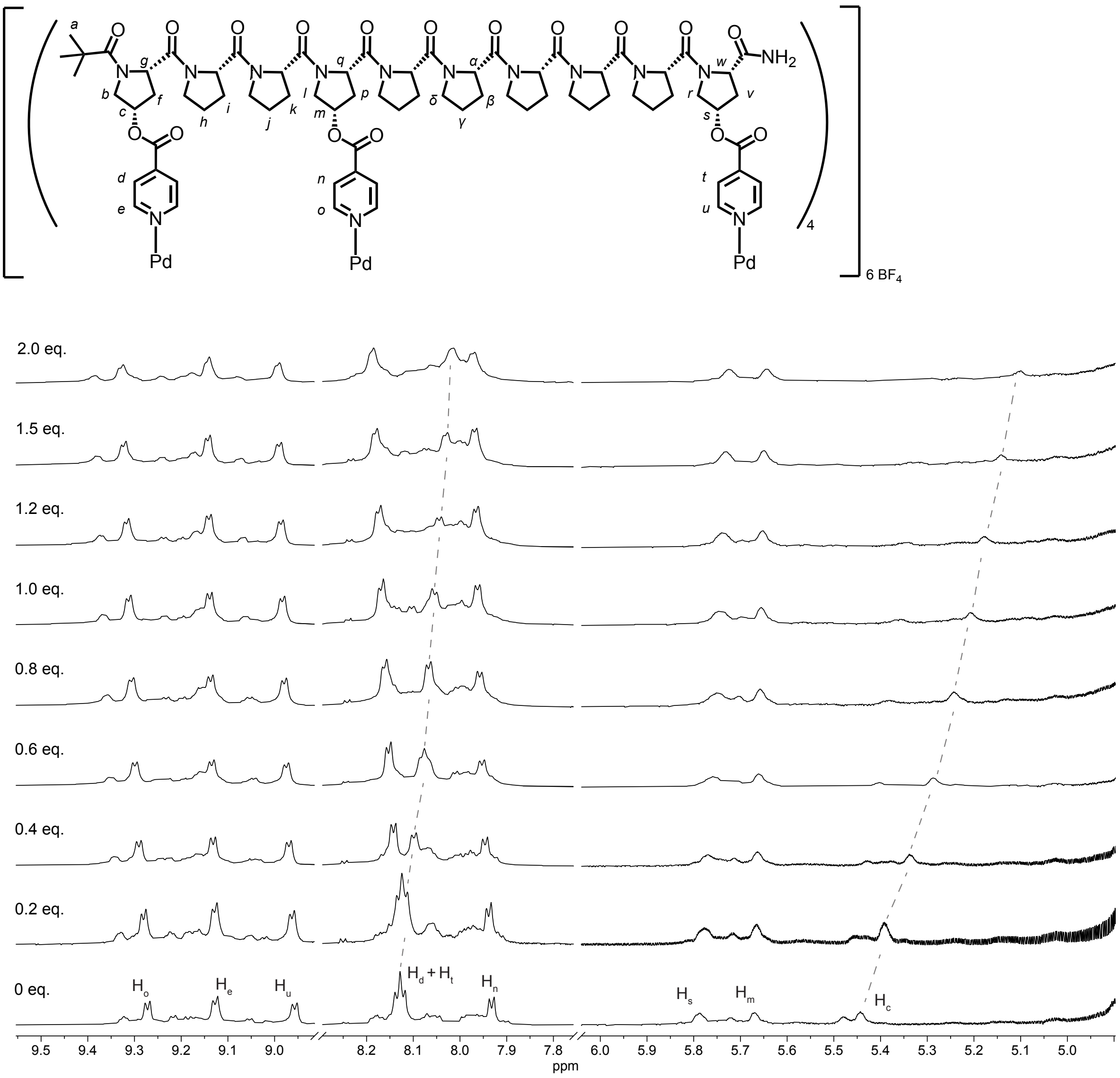

Figure S209. Partial ^{1}H NMR titration (D_2O, 600 MHz, 298 K) of **$Pd_3L^{4,S}_4$**$(BF_4)_6$ (0.25 mM) and varying equivalents of pyrene-1-sulfonate. Larger magnitude shift of external Hyp H_γ and H_β highlighted.

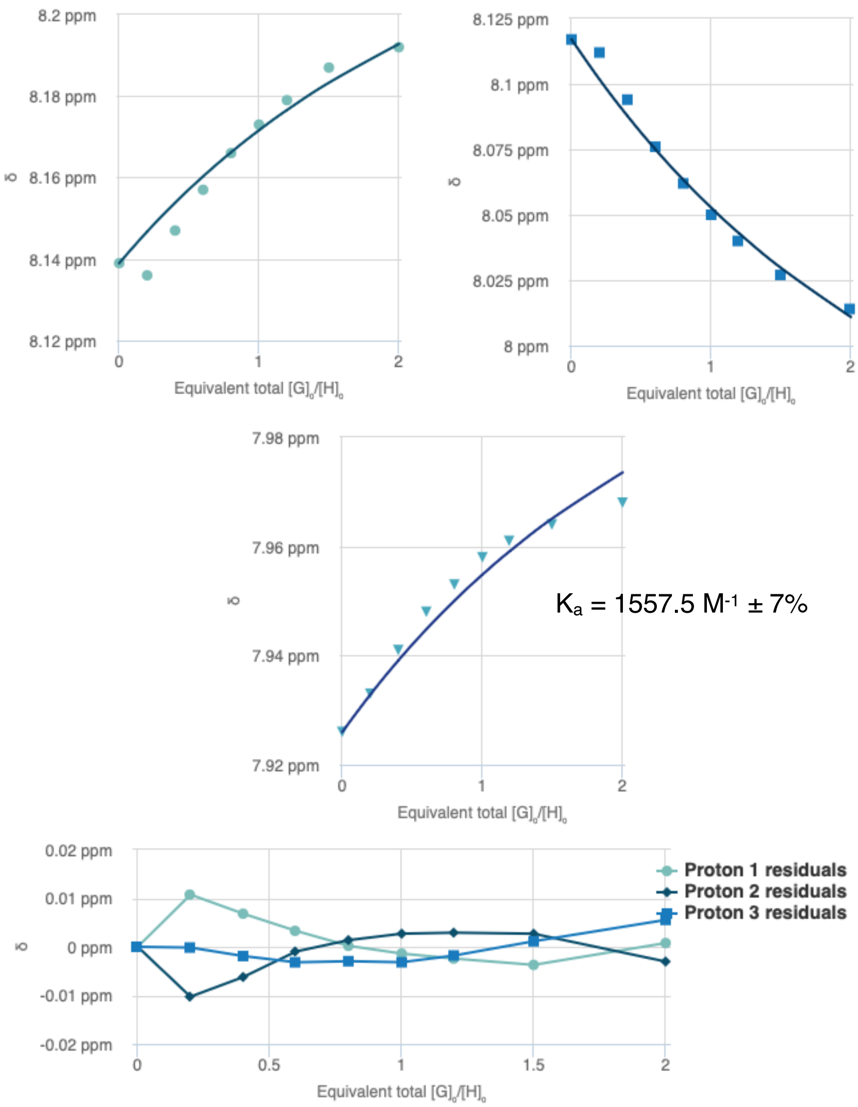

Figure S210. Binding plots for $\mathbf{Pd_3L^{4,S}_4}$(BF$_4$)$_6$ and sodium pyrene-1-sulfonate. K_a = 1557.5 M^{-1} ± 7%.

$Pd_3L^{7,R}{}_4(BF_4)_6$ + Irinotecan.HCl

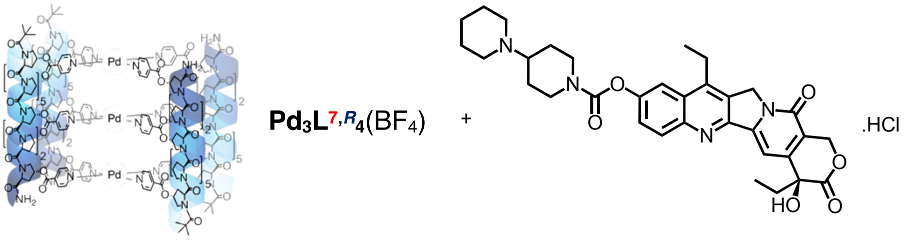

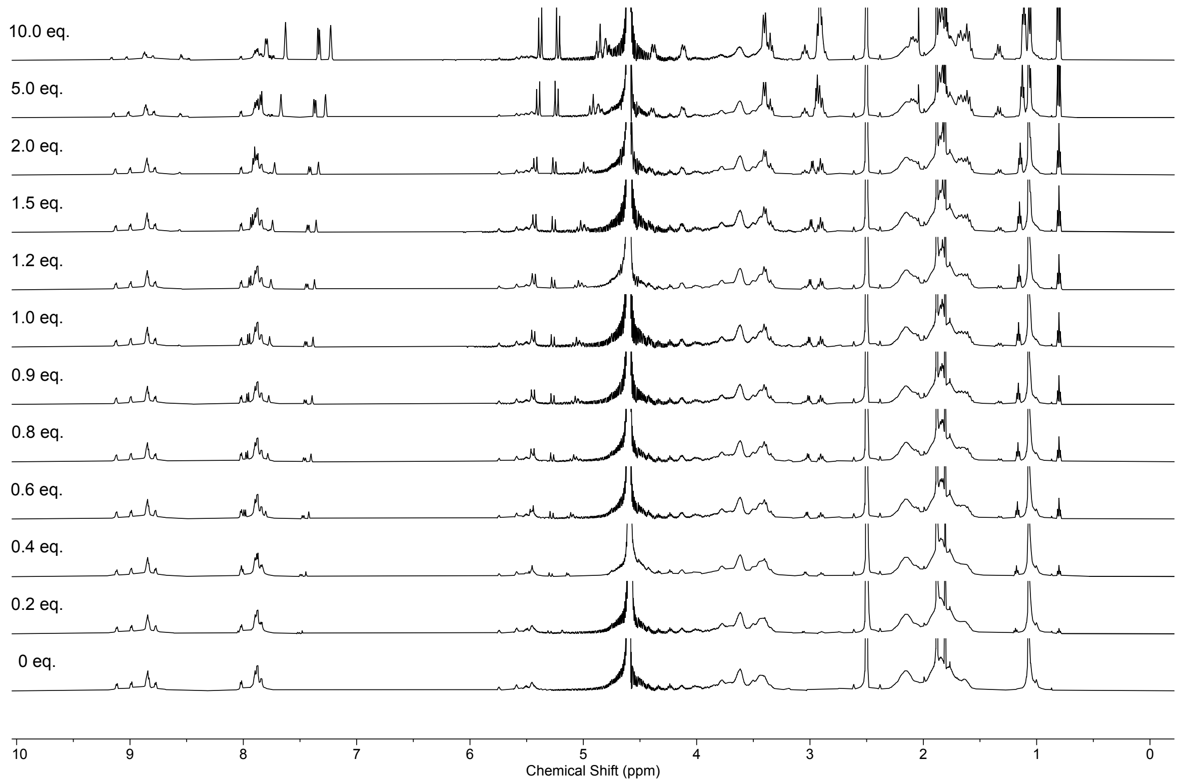

Figure S211. ^{1}H NMR titration (9:1 D_2O:d_6-DMSO, 600 MHz, 298 K) of **$Pd_3L^{7,R}{}_4$**$(BF_4)_6$ (0.25 mM) and varying equivalents of Irinotecan.HCl.

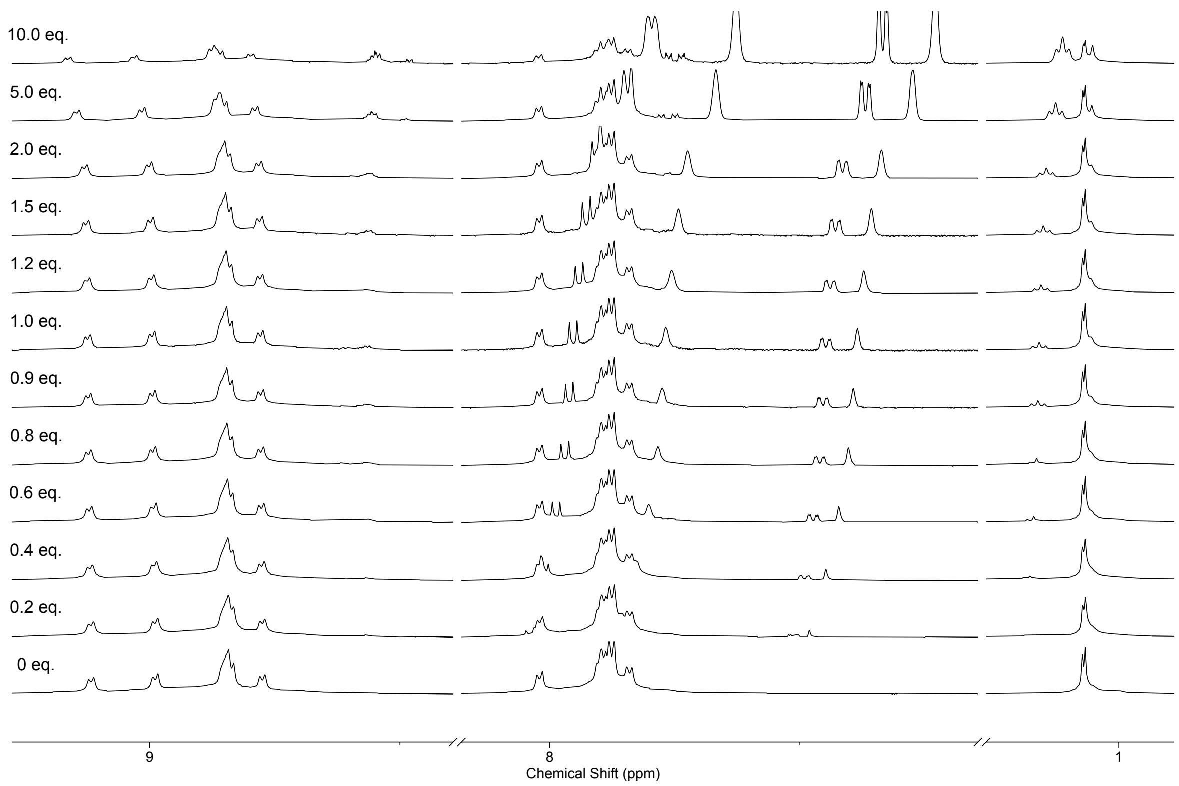

Figure S212. Partial ^{1}H NMR titration (9:1 D_2O:d_6-DMSO, 600 MHz, 298 K) of **$Pd_3L^{7,R}_4$**$(BF_4)_6$ (0.25 mM) and varying equivalents of Irinotecan.HCl.

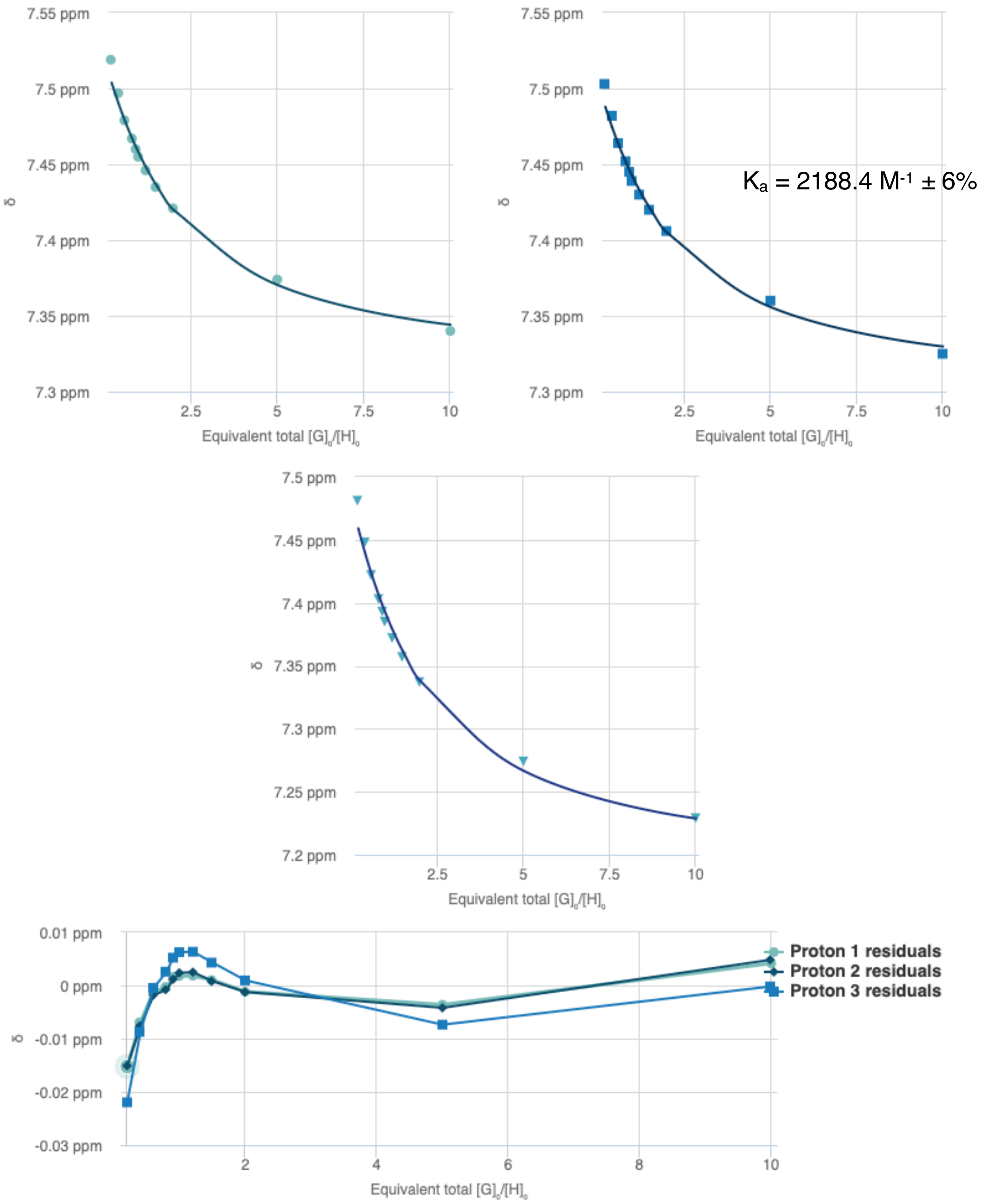

Figure S213. Binding plots for $\mathbf{Pd_3L^{7,R}_4}(BF_4)_6$ and Irinotecan.HCl. K_a = 2188.4 M^{-1} ± 6%.

$Pd_3L^{4,S}_4(BF_4)_6$ + Irinotecan.HCl

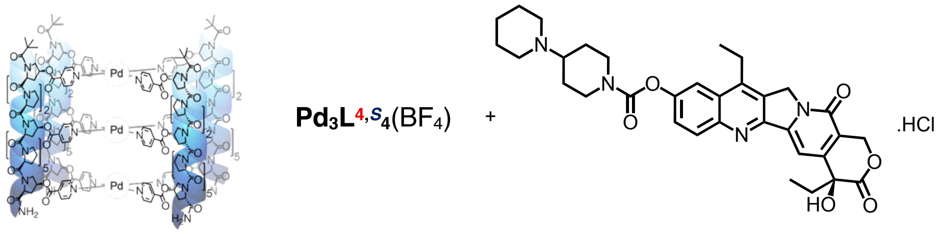

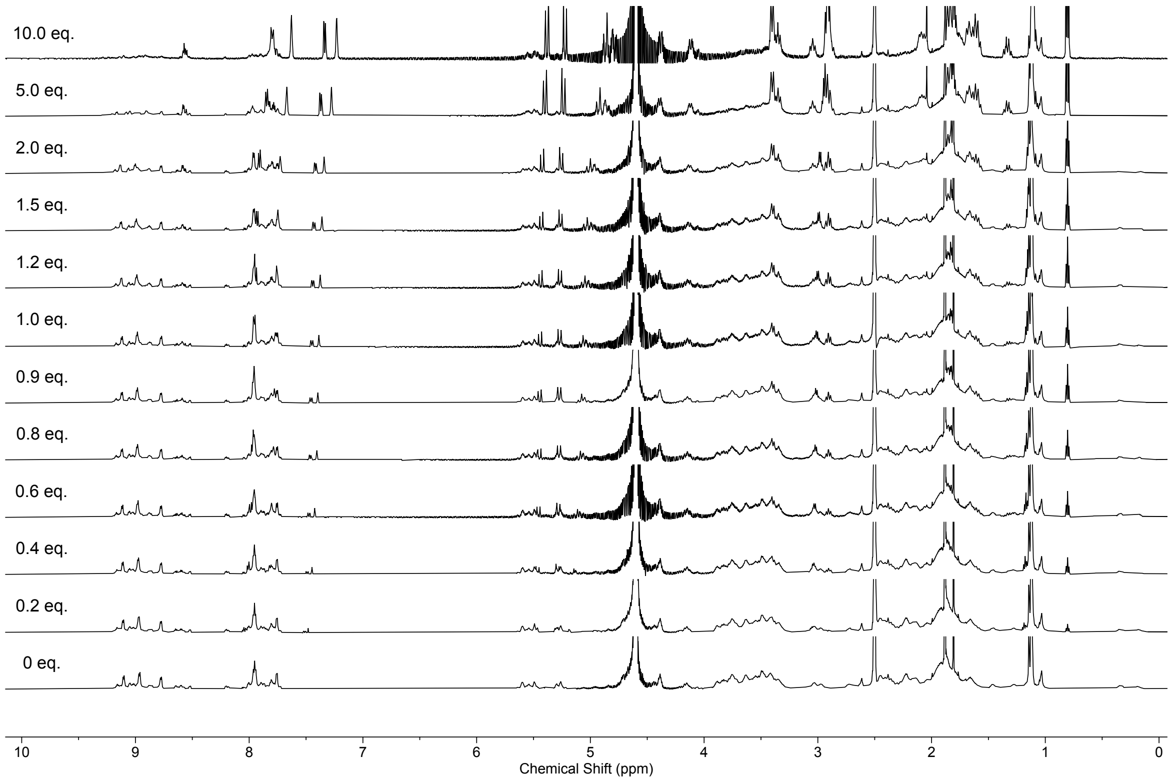

Figure S214. ¹H NMR titration (9:1 D_2O:d_6-DMSO, 600 MHz, 298 K) of **$Pd_3L^{4,S}_4(BF_4)_6$** (0.25 mM) and varying equivalents of Irinotecan.HCl.

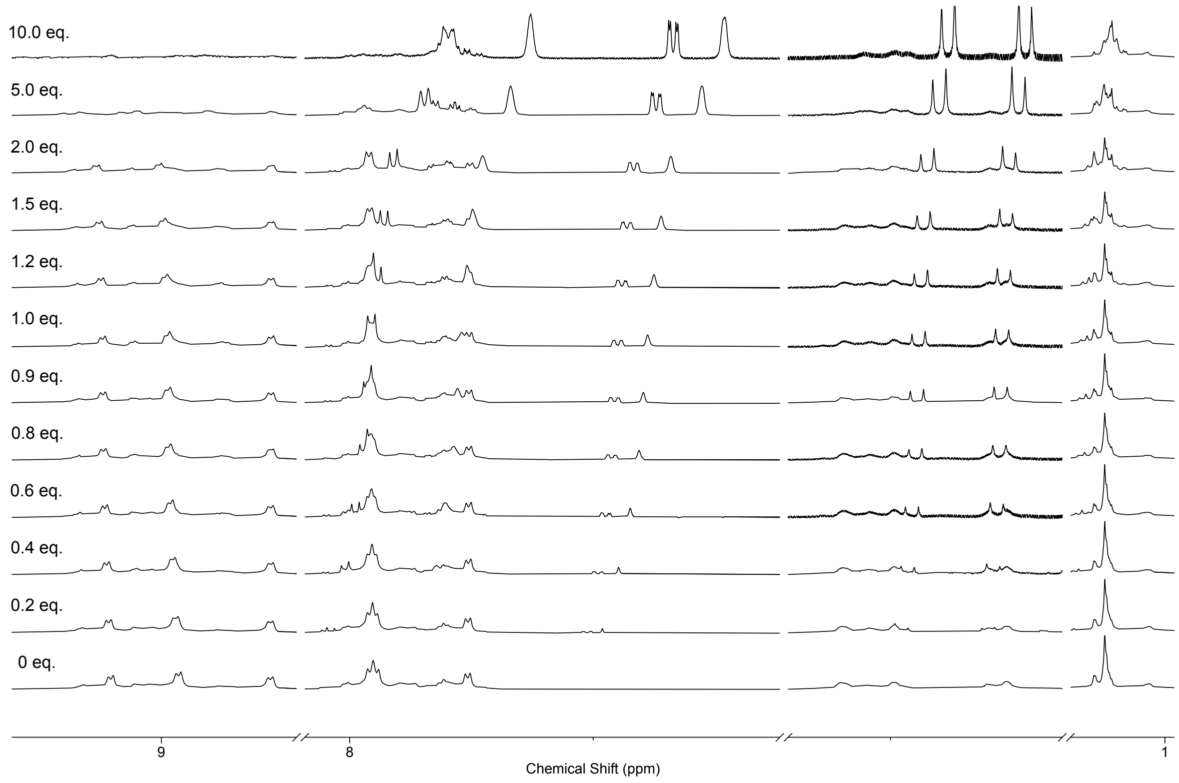

Figure S215. Partial ^{1}H NMR titration (9:1 D_2O:d_6-DMSO, 600 MHz, 298 K) of $\mathbf{Pd_3L^{4,S}_4}(BF_4)_6$ (0.25 mM) and varying equivalents of Irinotecan.HCl. Peaks at ~1 ppm have been scaled independently to the rest of the spectrum.

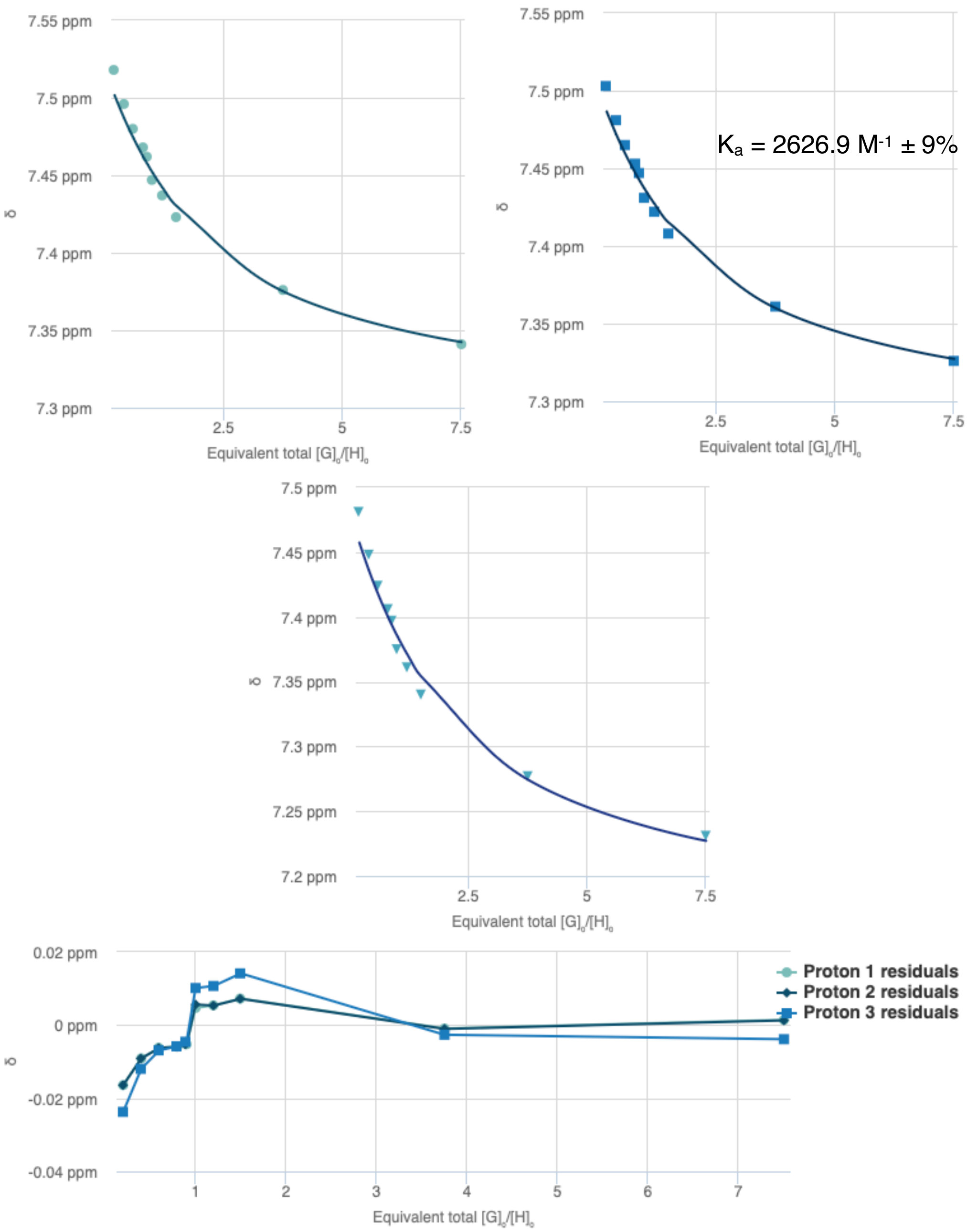

Figure S216. Binding plots for **$Pd_3L^{4,S}{}_4$**$(BF_4)_6$ and Irinotecan.HCl. K_a = 2626.9 M^{-1} ± 9%.

$Pd_3L^{7,R}_4(BF_4)_6$ + Sodium D-10-Camphorsulfonate

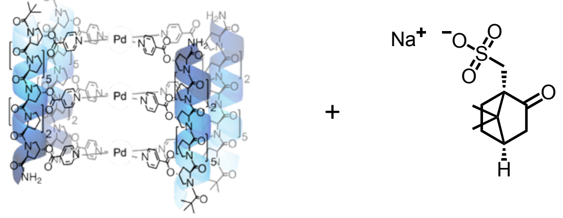

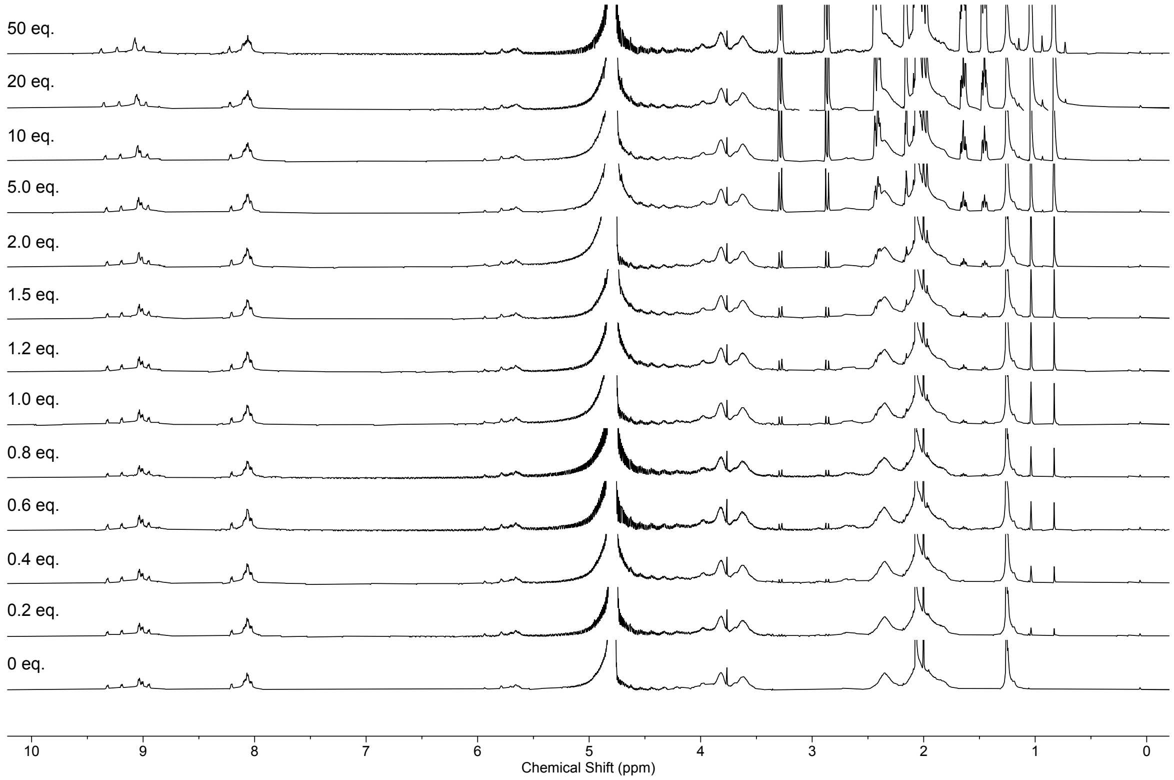

Figure S217. ^{1}H NMR titration (D_2O, 600 MHz, 298 K) of **$Pd_3L^{7,R}_4$**$(BF_4)_6$ (0.25 mM) and varying equivalents of Sodium *D*-10-Camphorsulfonate.

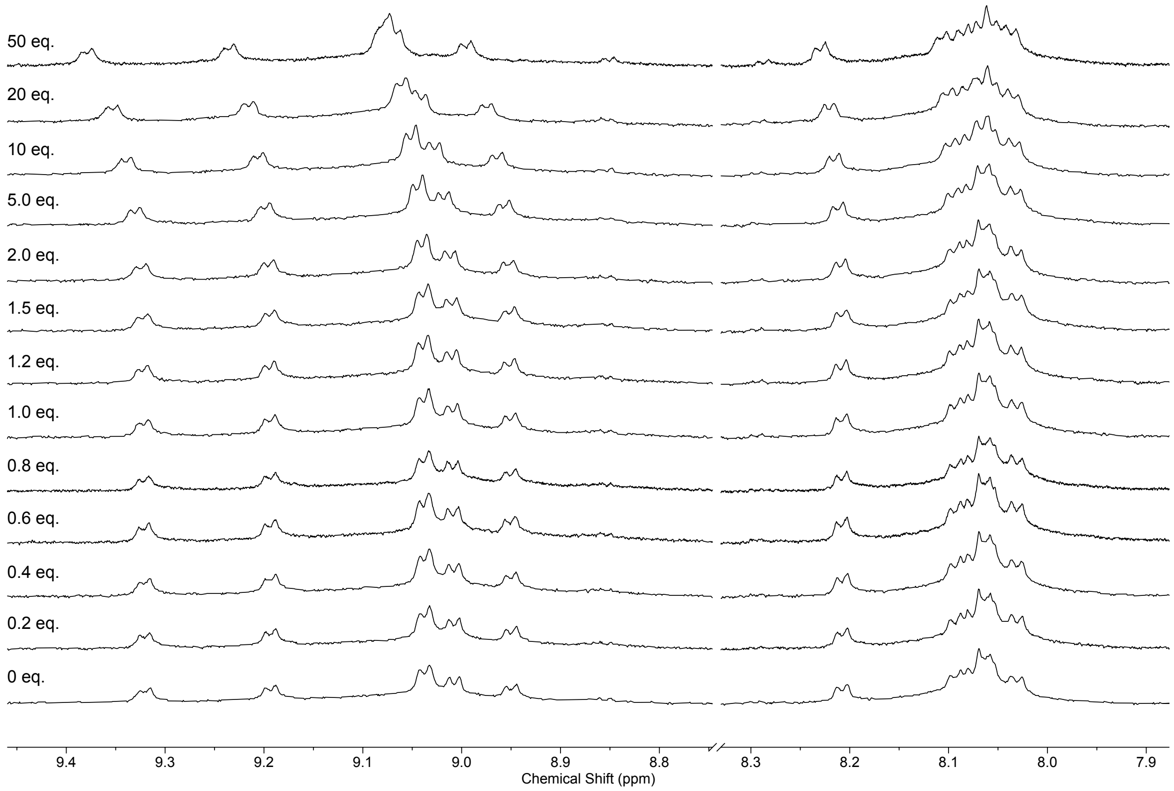

Figure S218. Partial ^{1}H NMR titration (D_2O, 600 MHz, 298 K) of **$Pd_3L^{7,R}{}_4$**$(BF_4)_6$ (0.25 mM) and varying equivalents of Sodium *D*-10-Camphorsulfonate.

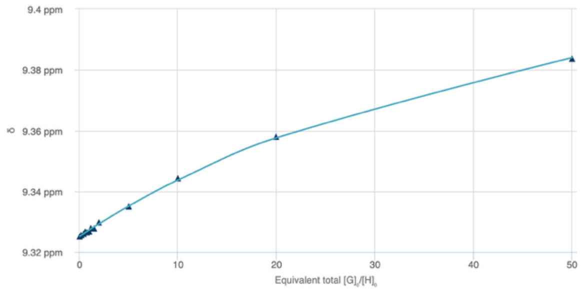

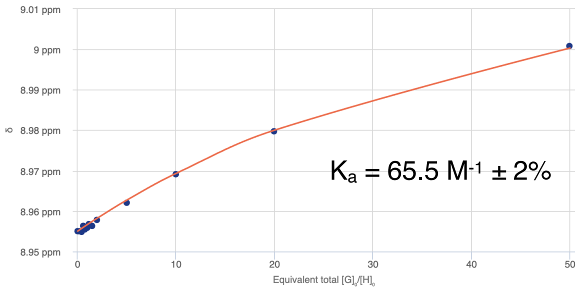

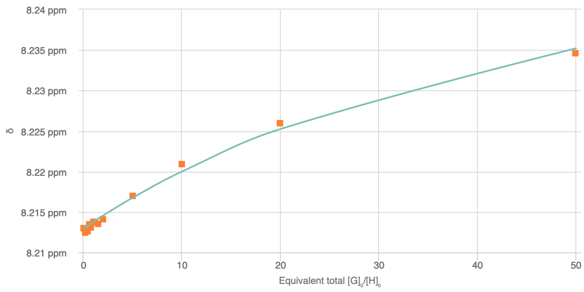

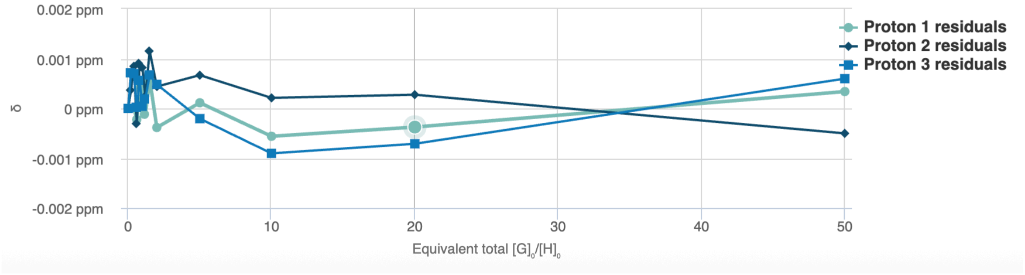

Figure S219. Binding plots for $\mathbf{Pd_3L^{7,\mathit{R}}{}_4}(BF_4)_6$ and Sodium *D*-10-Camphorsulfonate. $K_a = 65.5\ M^{-1} \pm 2\%$.

$Pd_3L^{4,S}{}_4(BF_4)_6$ + Sodium D-10-Camphorsulfonate

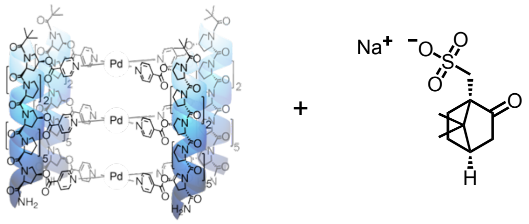

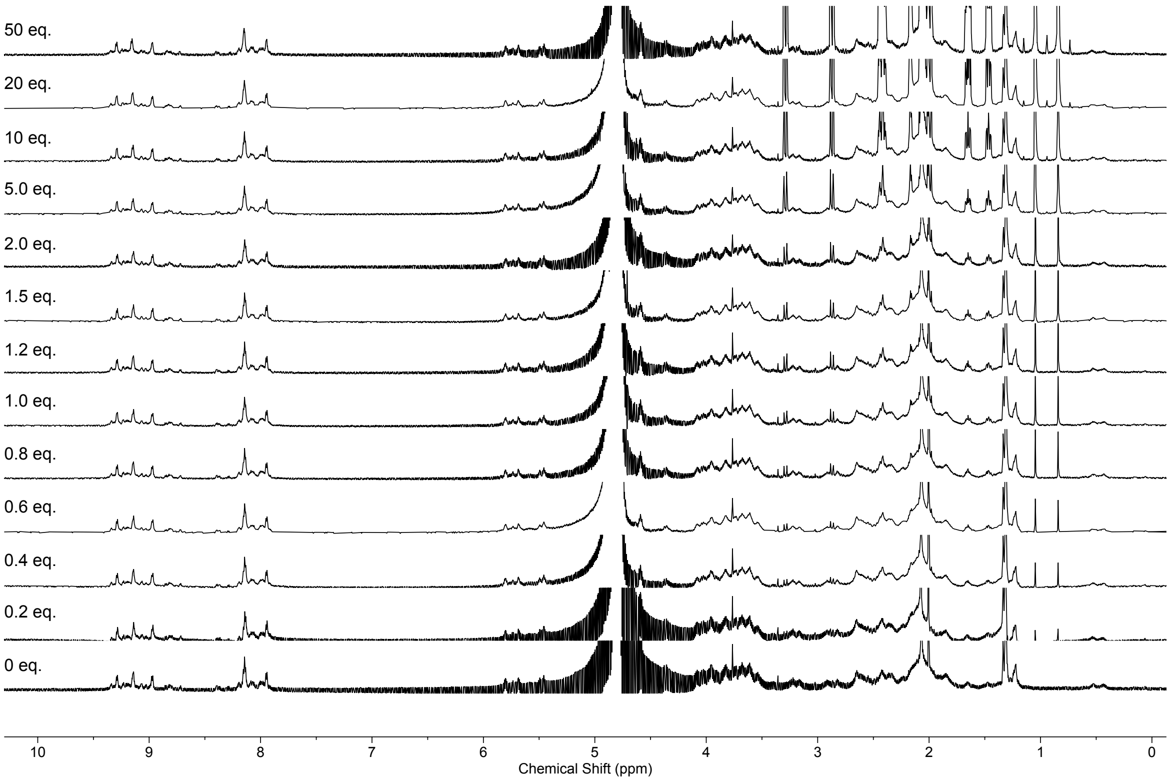

Figure S220. ^1H NMR titration (D_2O, 600 MHz, 298 K) of $\mathbf{Pd_3L^{4,\mathit{S}}{}_4}(BF_4)_6$ (0.25 mM) and varying equivalents of Sodium *D*-10-Camphorsulfonate.

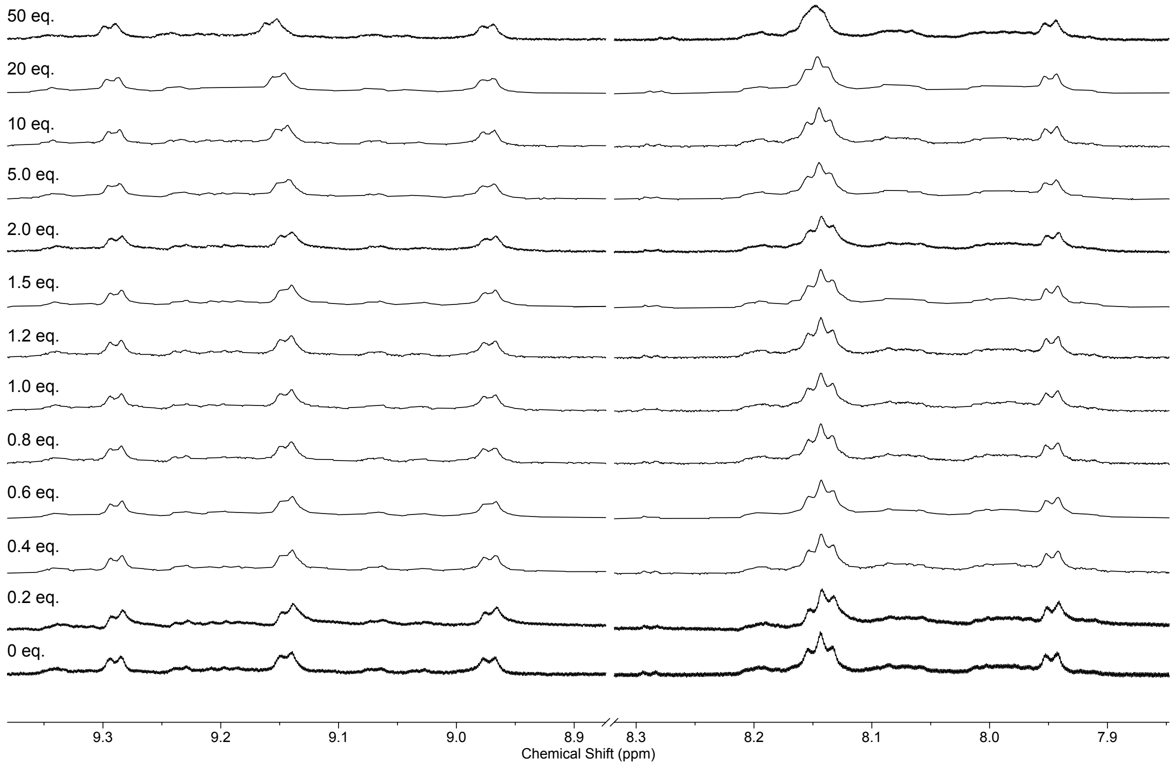

Figure S221. Partial ^{1}H NMR titration (D_2O, 600 MHz, 298 K) of $\mathbf{Pd_3L^{4,S}_4}(BF_4)_6$ (0.25 mM) and varying equivalents of Sodium *D*-10-Camphorsulfonate.

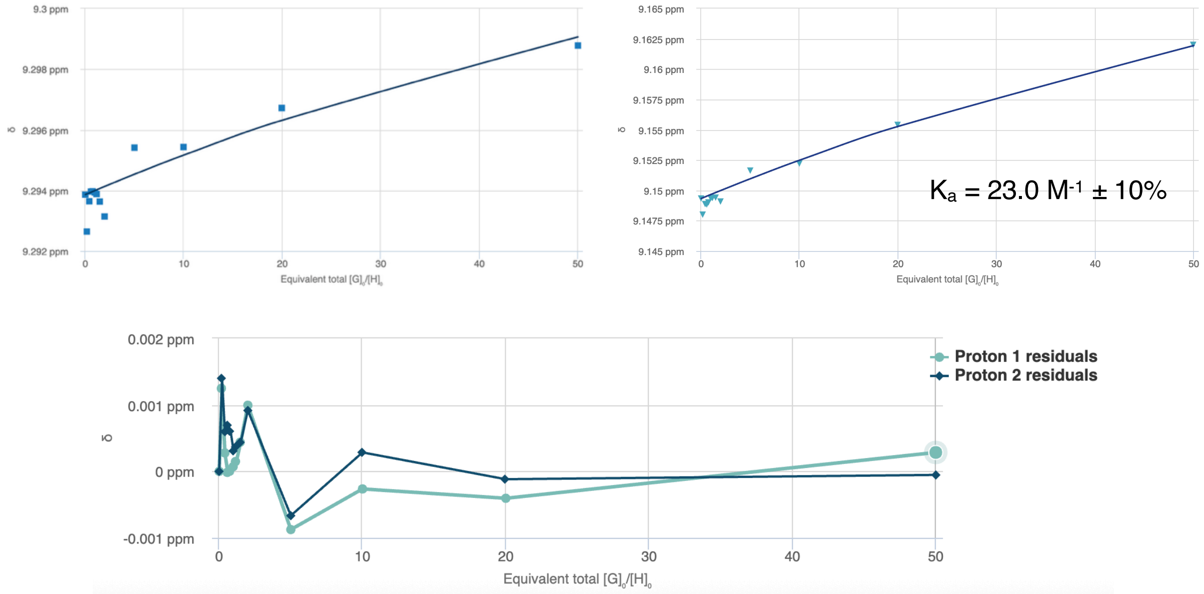

Figure S222. Binding plots for $\mathbf{Pd_3L^{4,S}_4}(BF_4)_6$ and Sodium *D*-10-Camphorsulfonate. K_a = 23.0 M^{-1} ± 10%.

$Pd_3L^{7,R}{}_4(BF_4)_6$ + Sodium (±)-10-Camphorsulfonate

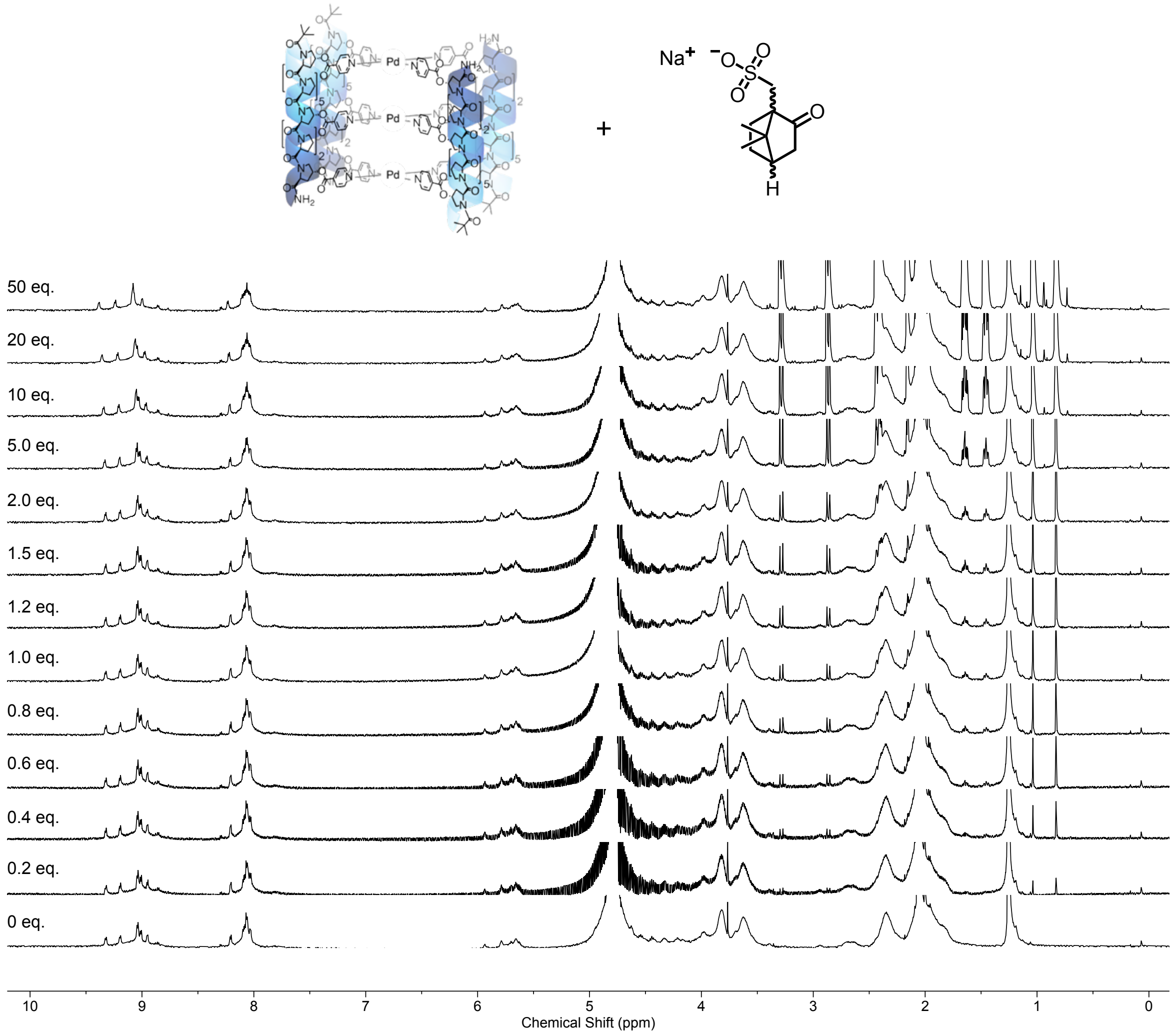

Figure S223. ^{1}H NMR titration (D_2O, 600 MHz, 298 K) of **$Pd_3L^{7,R}{}_4$**$(BF_4)_6$ (0.25 mM) and varying equivalents of Sodium (±)-10-Camphorsulfonate.

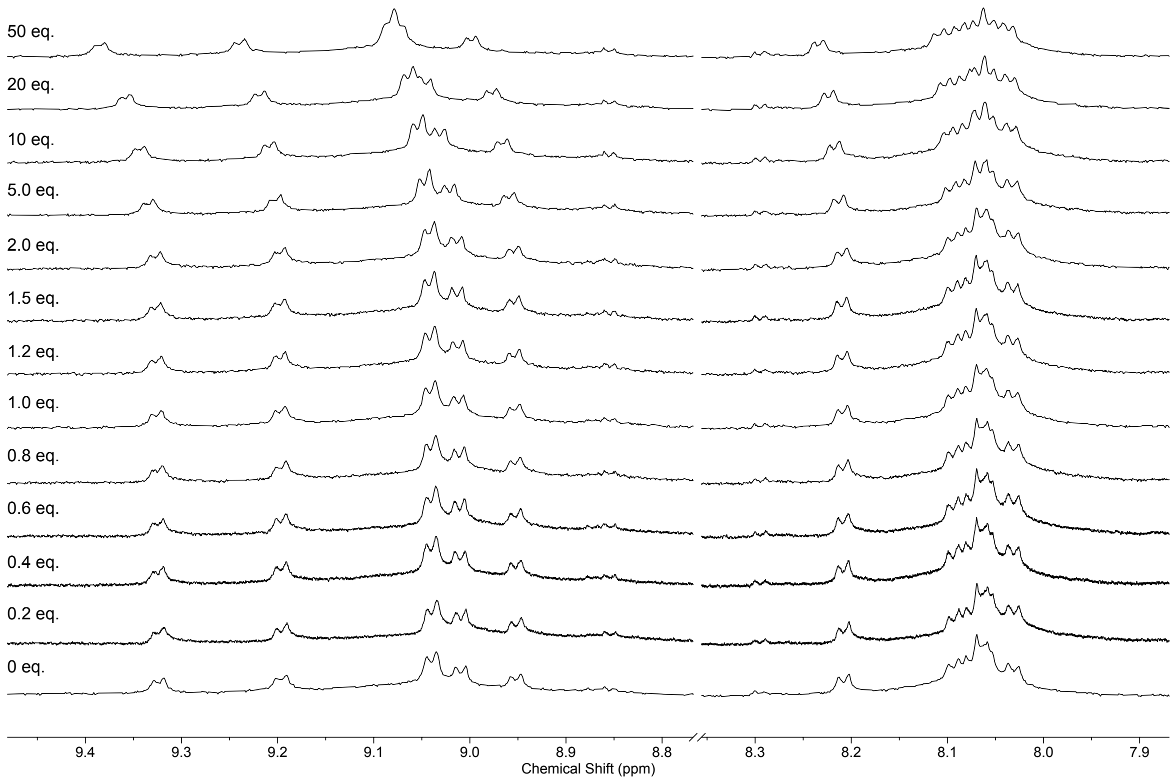

Figure S224. Partial ^{1}H NMR titration (D_2O, 600 MHz, 298 K) of $\mathbf{Pd_3L^{7,R}_4}(BF_4)_6$ (0.25 mM) and varying equivalents of Sodium (±)-10-Camphorsulfonate.

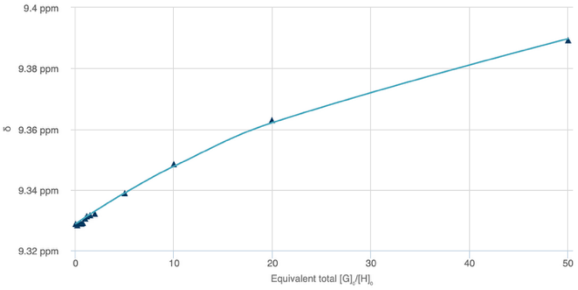

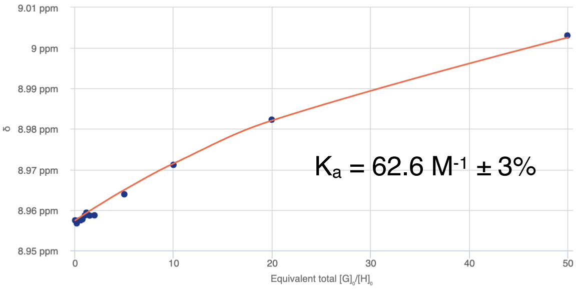

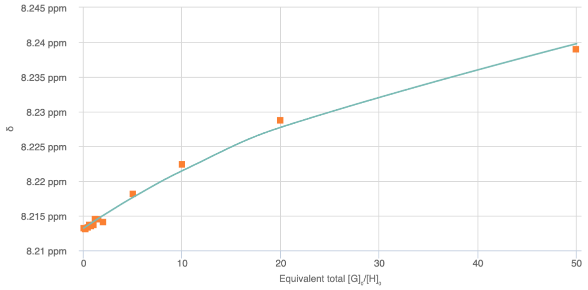

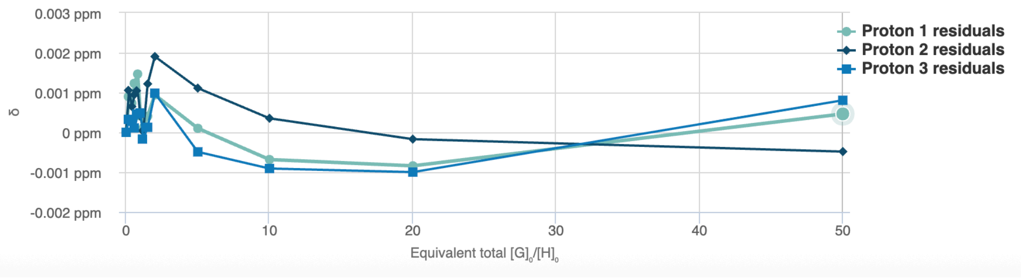

Figure S225. Binding plots for $\mathbf{Pd_3L^{7,R}_4}(BF_4)_6$ and Sodium (±)-10-Camphorsulfonate. K_a = 62.6 M^{-1} ± 3%.

$Pd_3L^{4,S}_4(BF_4)_6$ + Sodium (±)-10-Camphorsulfonate

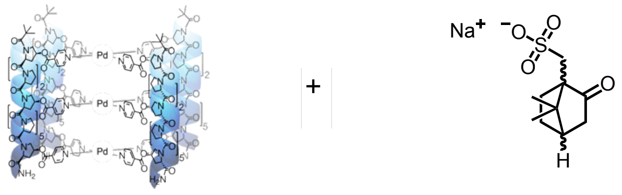

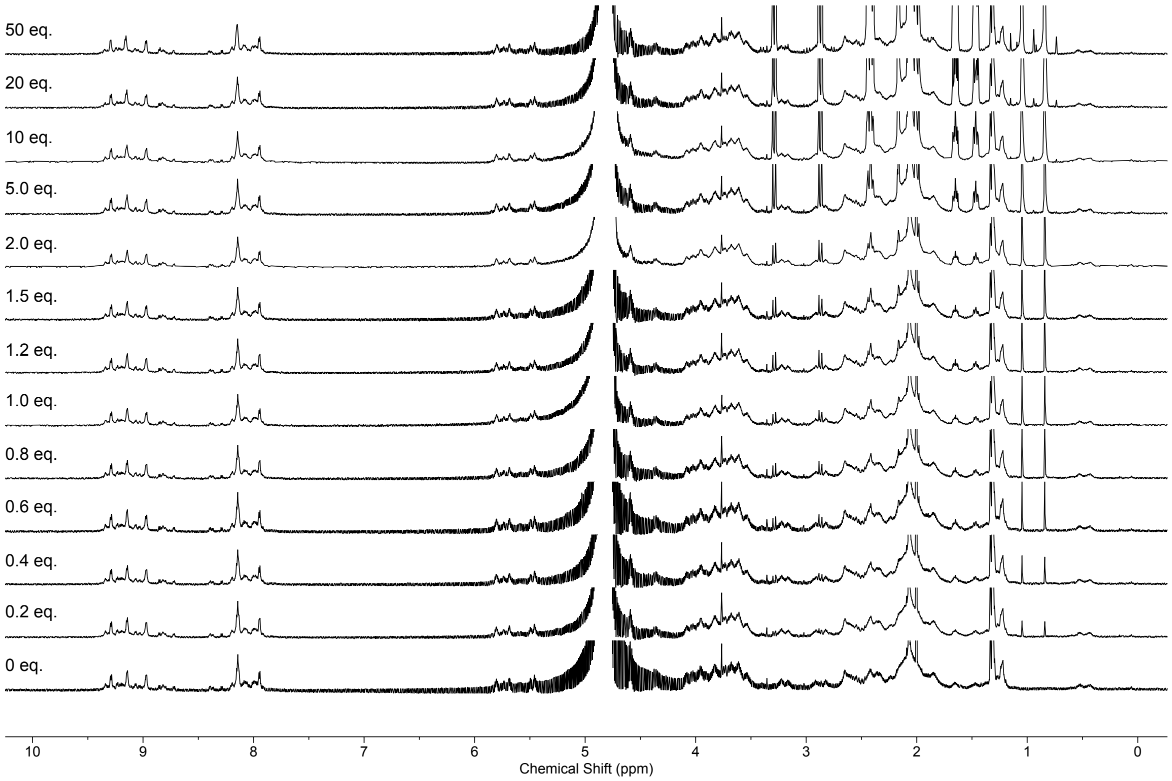

Figure S226. ^1H NMR titration (D_2O, 600 MHz, 298 K) of $\mathbf{Pd_3L^{4,S}_4}(BF_4)_6$ (0.25 mM) and varying equivalents of Sodium (±)-10-Camphorsulfonate.

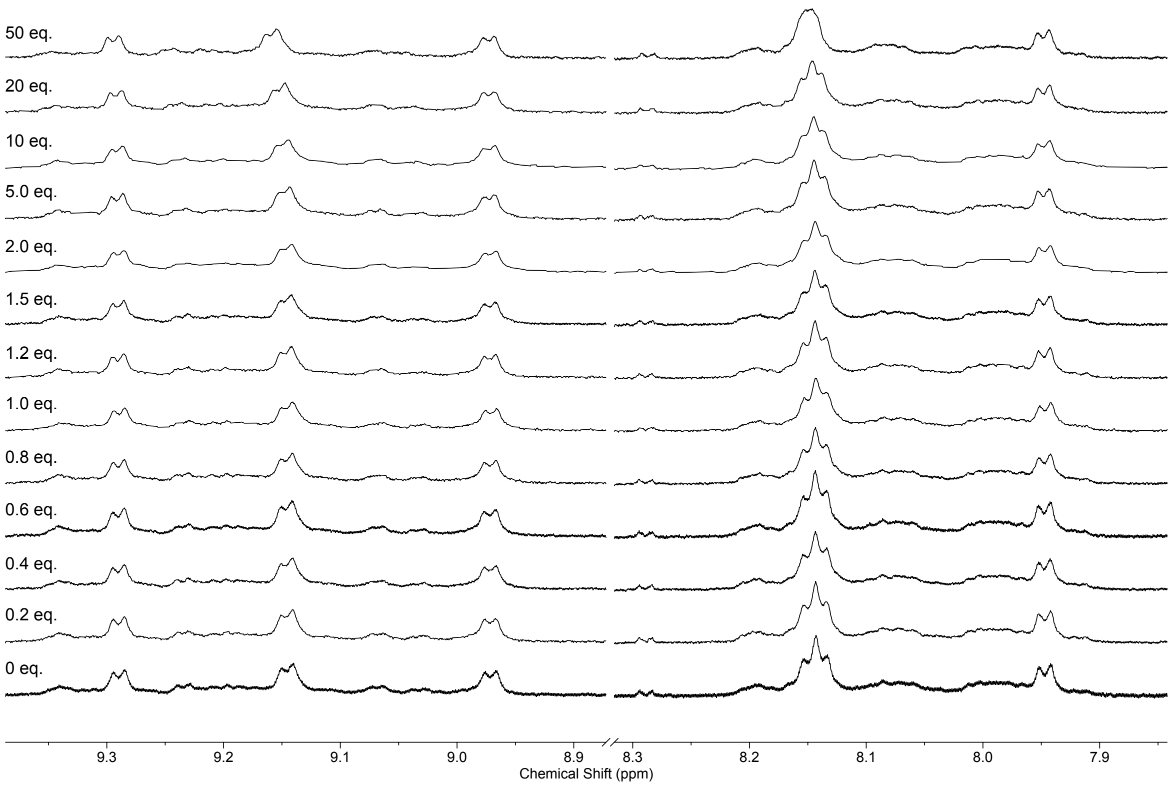

Figure S227. Partial ^{1}H NMR titration (D_2O, 600 MHz, 298 K) of $\mathbf{Pd_3L^{4,\mathit{S}}_4}(BF_4)_6$ (0.25 mM) and varying equivalents of Sodium (±)-10-Camphorsulfonate.

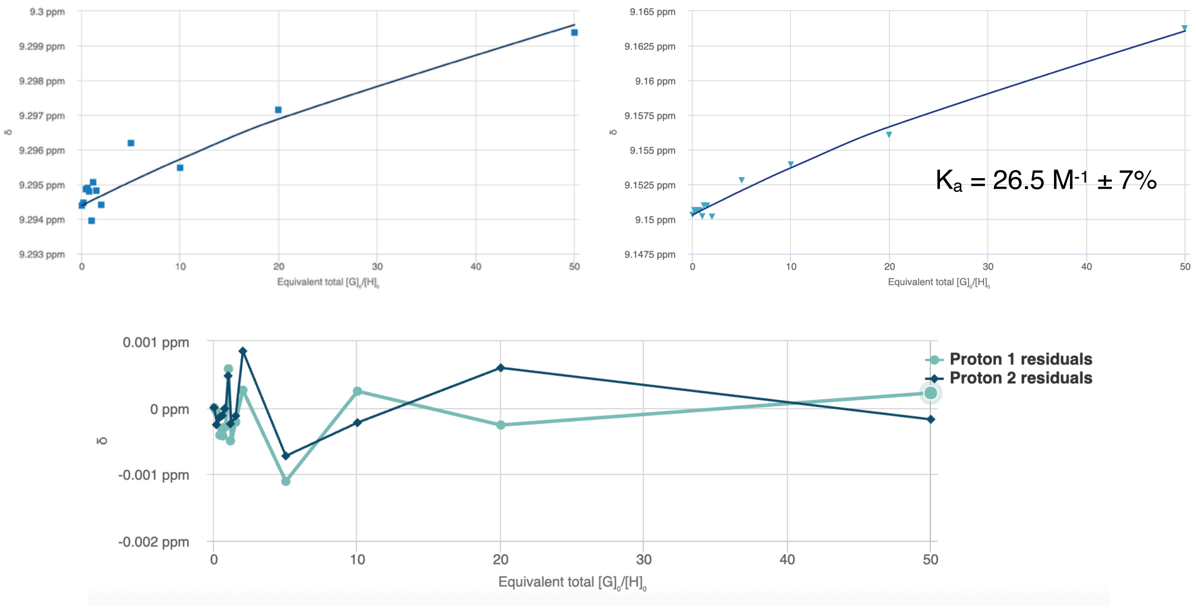

Figure S228. Binding plots for $\mathbf{Pd_3L^{4,\mathit{S}}_4}(BF_4)_6$ and Sodium (±)-10-Camphorsulfonate. K_a = 26.5 M^{-1} ± 7%.

12. Molecular Models

Structures and energies were calculated with SAMSON, using the crystal structure of a hexaproline in the PPII conformation[S11] and our Pd_2L_4 models[S3] to build ligand strands. Structures were manually positioned to minimise apparent strain, before minimisation routines were performed. Once convergence had been achieved, further refinement was undertaken by molecular modelling (Universal Force Field, with Fast Inertial Relaxation Engine, SAMSON)[S12] which was run until iterations converged within 1 kJ/mol. Where appropriate, minimised energies have been included.

All amide bonds were positioned in the *trans* conformation and all 4-substituted proline residues were positioned in an exo ring pucker regardless of the 4*S*/4*R* substitution preference. Whilst we know from experimental ^{1}H NMR and CD data that 4*S*-substituted ligands $\mathbf{L}^{7,S}$ and $\mathbf{L}^{4,S}$ show some level of *cis*-*trans* isomerism and therefore a decrease in PPII character (Figures S21, S29, S31 + S39), we were unable to determine the extent of this isomerism along the proline backbone or the level of deviation this imparts from an ideal PPII structure. As such, we were unable to account for this in our models of cages $\mathbf{Pd_3L^{7,S}_4}$ and $\mathbf{Pd_3L^{4,S}_4}$, neither do we account for any possible cage collapse in cage $\mathbf{Pd_3L^{4,S}_4}$. As such, these models provide an indication and visual guide.

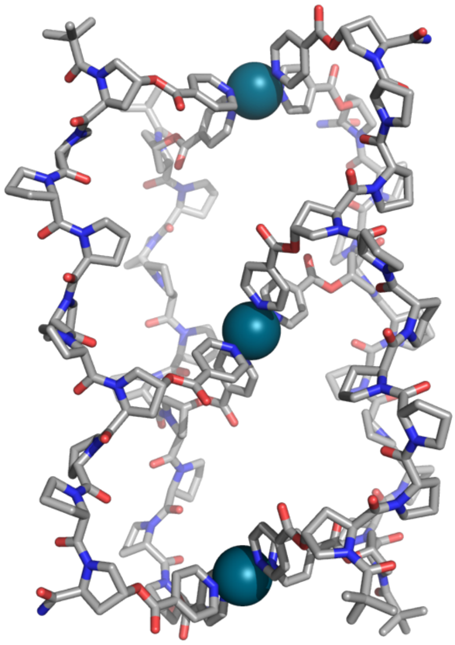

Figure S229. Energy minimised *cis CCNN* cage $\mathbf{Pd_3L^{7,R}_4}(BF_4)_6$, side view. Minimised energy 6991 kJ/mol.

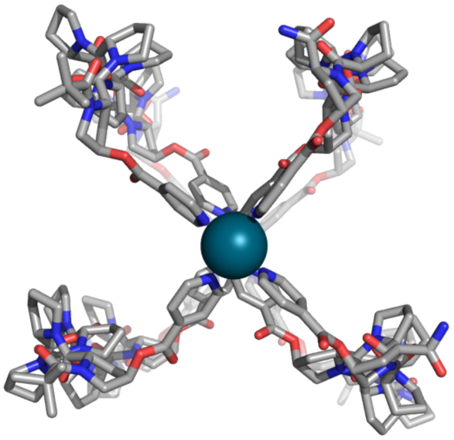

Figure S230. Energy minimised *cis CCNN* cage **$Pd_3L^{7,R}{}_4$**$(BF_4)_6$, top view. Minimised energy 6991 kJ/mol.

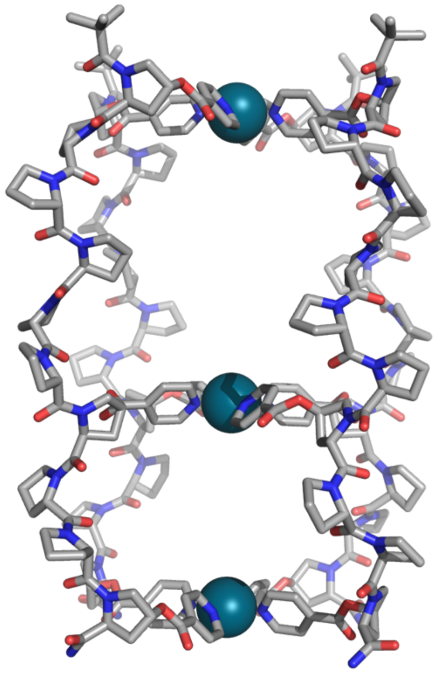

Figure S231. Alternative ‘All Up’ *CCCC* structural isomer of cage **$Pd_3L^{7,R}{}_4$**$(BF_4)_6$, side view. Minimised energy 7078 kJ/mol.

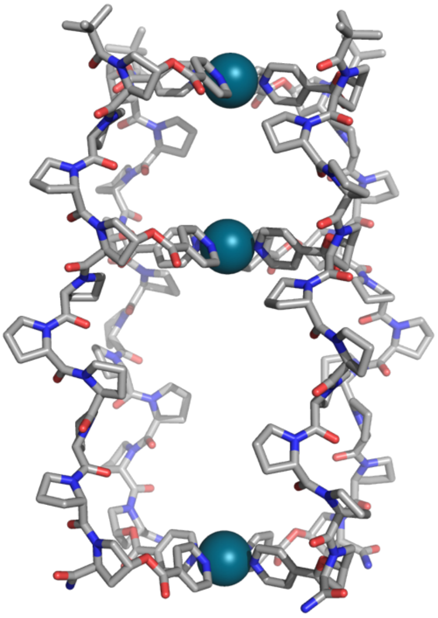

Figure S232. Energy minimised 'All Up' *CCCC* isomer of cage $\mathbf{Pd_3L^{4,\mathit{R}}_4}(BF_4)_6$, side view. Minimised energy 6802 kJ/mol.

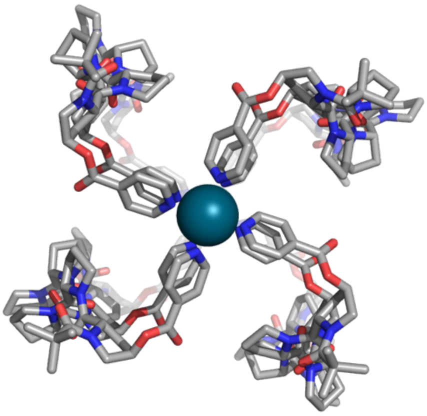

Figure S233. Energy minimised 'All Up' *CCCC* isomer of cage $\mathbf{Pd_3L^{4,\mathit{R}}_4}(BF_4)_6$, top view. Minimised energy 6802 kJ/mol.

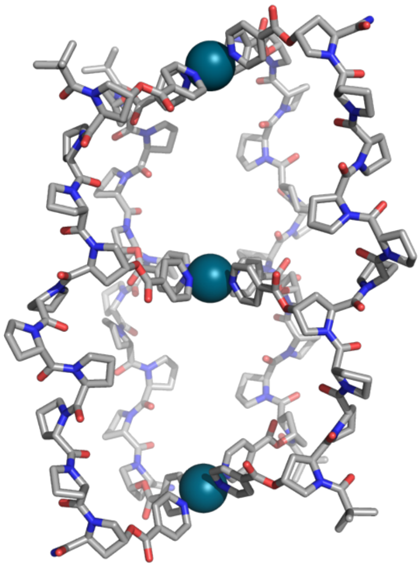

Figure S234. Energy minimised *cis CCNN* isomer of cage $\mathbf{Pd_3L^{4,\mathit{R}}_4}(BF_4)_6$, side view. Minimised energy 6986 kJ/mol.

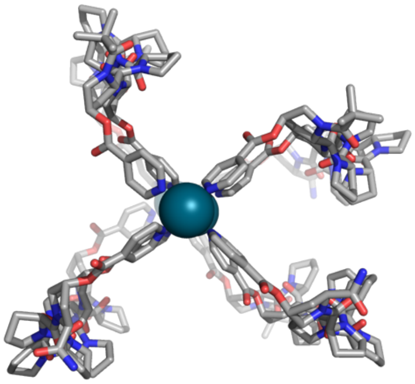

Figure S235. Energy minimised *cis CCNN* isomer of cage $\mathbf{Pd_3L^{4,\mathit{R}}_4}(BF_4)_6$, top view. Minimised energy 6986 kJ/mol.

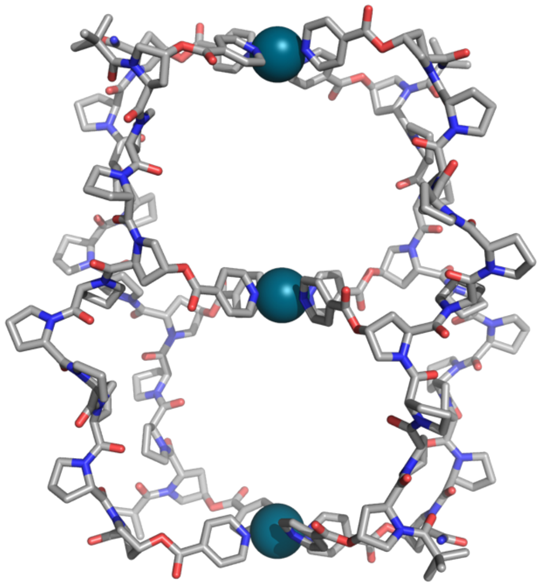

Figure S236. Energy minimised *trans CNCN* isomer of cage **$Pd_3L^{4,R}_4$**$(BF_4)_6$, side view. Minimised energy 6950 kJ/mol.

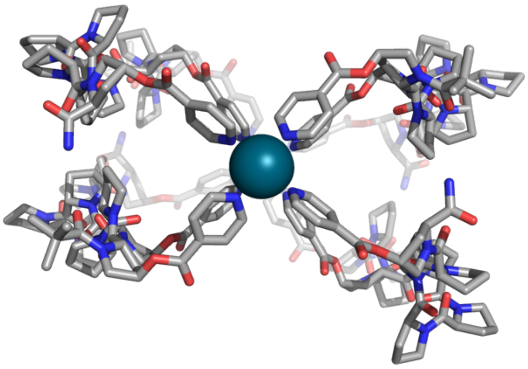

Figure S237. Energy minimised *trans CNCN* isomer of cage **$Pd_3L^{4,R}_4$**$(BF_4)_6$
, top view. Minimised energy 6950 kJ/mol.

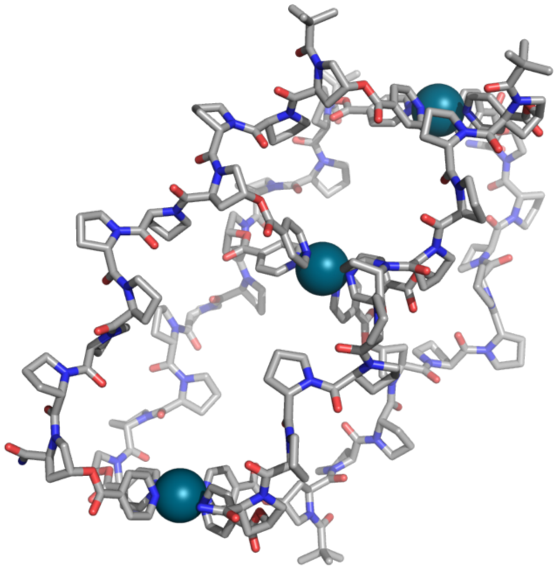

Figure S238. Energy minimised *CCCN* isomer of cage $\mathbf{Pd_3L^{4,\mathit{R}}_4}(BF_4)_6$, side view. Minimised energy 6963 kJ/mol.

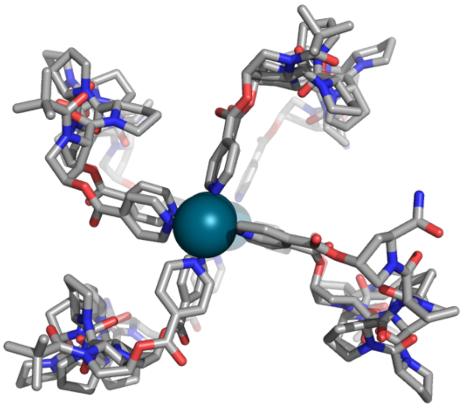

Figure S239. Energy minimised *CCCN* isomer of cage $\mathbf{Pd_3L^{4,\mathit{R}}_4}(BF_4)_6$, top view. Minimised energy 6963 kJ/mol.

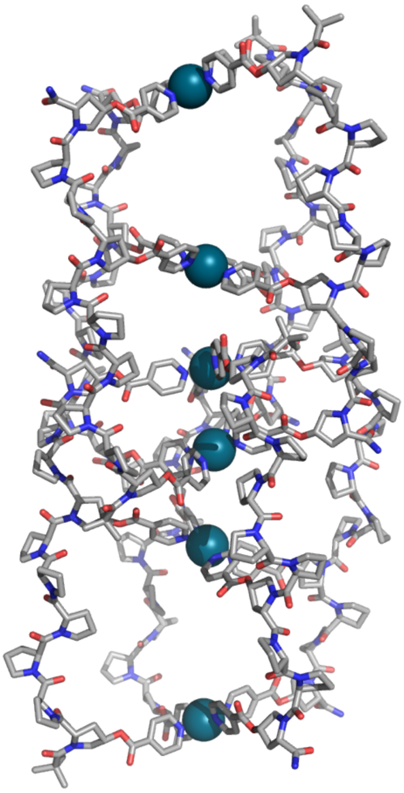

Figure S240. Energy minimised interpenetrated cage $[\mathbf{Pd_3L^{7,S}_4}(BF_4)_6]_2$, side view. Representative structure shows a four-fold interpenetration of two *cis CCNN* cages.

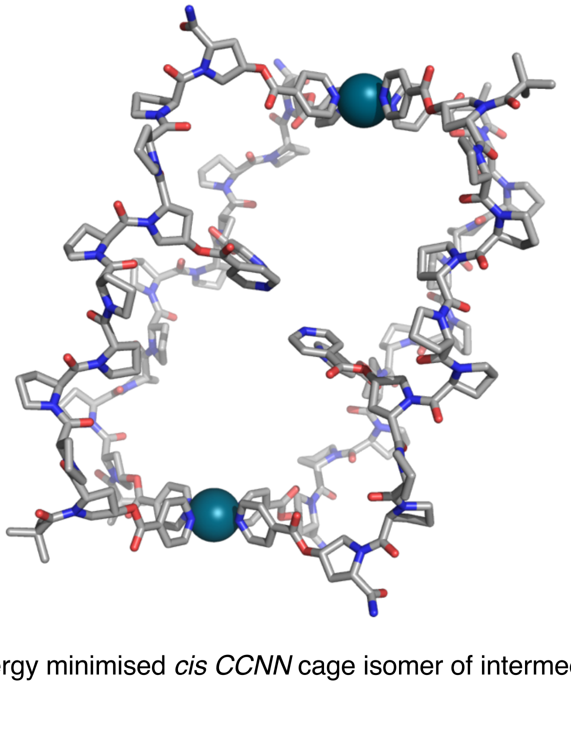

Figure S241. Energy minimised *cis CCNN* cage isomer of intermediate $\mathbf{Pd_2L^{7,S}_4}(BF_4)$, side view.

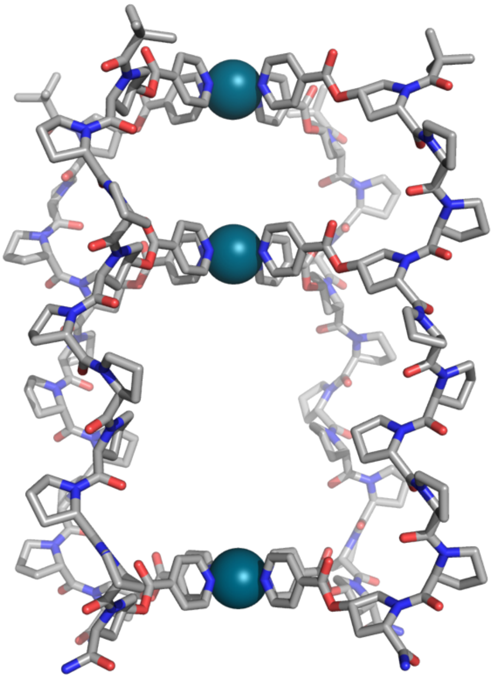

Figure S242. Energy minimised ‘All Up’ *CCCC* cage $\mathbf{Pd_3L^{4,\mathit{S}}_4}(BF_4)_6$, side view. Minimised energy 6672 kJ/mol.

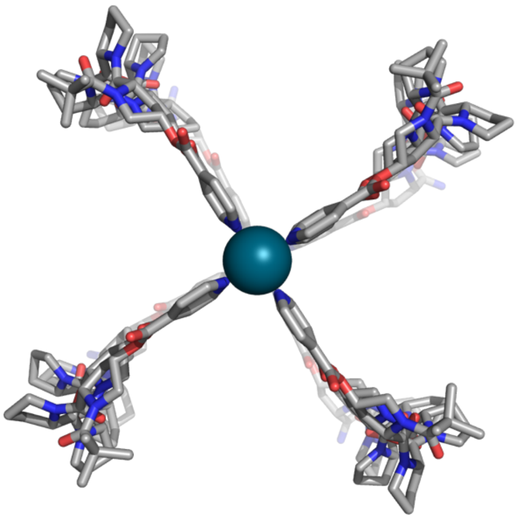

Figure S243. Energy minimised ‘All Up’ *CCCC* cage $\mathbf{Pd_3L^{4,\mathit{S}}_4}(BF_4)_6$, top view. Minimised energy 6672 kJ/mol.

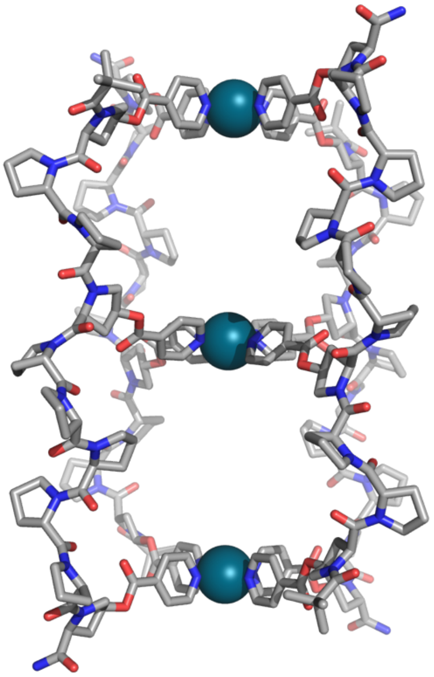

Figure S244. Alternative *trans CNCN* structural isomer of cage **Pd$_3$L^{4,S}$_4$**(BF$_4$)$_6$, side view. Minimised energy 6691 kJ/mol.

13. References

[S1] E. A. Mason and E. W. McDaniel,*Transport Properties of Ions in Gases*, John Wiley & Sons, 1988. DOI: 10.1002/3527602852.

[S2] S. L. Heinz-Kunert, A. Pandya, V. T. Dang, P. N. Tran, S. Ghosh, D. McElheny, B. D. Santarsiero, Z. Ren and A. I. Nguyen, *J. Am. Chem. Soc.*, 2022, **144** (15), 7001–7009. DOI: 10.1021/jacs.2c02146.

[S3] B. E. Barber, E. M. G. Jamieson, L. E. M. White and C. T. McTernan, *Chem,* 2024, **10** (9), 2792–2806. DOI: 10.1016/j.chempr.2024.05.002.

[S4] J. Horng and R. T. Raines, *Protein Sci.*, 2006, **15** (1), 74–83. DOI: 10.1110/ps.051779806.

[S5] T. J. El-Baba, D. R. Fuller, D. A. Hales, D. H. Russell and D. E. Clemmer, *J. Am. Soc. Mass Spectrom.*, 2019, **30** (1), 77–84. DOI: 10.1007/s13361-018-2034-7.

[S6] L. Shi, A. E. Holliday, M. S. Glover, M. A. Ewing, D. H. Russell and D. E. Clemmer, *J. Am. Soc. Mass Spectrom.*, 2016, **27** (1), 22–30. DOI: 10.1007/s13361-015-1255-2.

[S7] L. Shi, A. E. Holliday, B. C. Bohrer, D. Kim, K. A. Servage, D. H. Russell and D. E. Clemmer, *J. Am. Soc. Mass Spectrom.*, 2016, **27** (6), 1037–1047. DOI: 10.1007/s13361-016-1372-6.

[S8] K. Wu, E. Benchimol, A. Baksi and G. H. Clever, *Nat. Chem.*, 2024, **16**, 584–591. DOI: 10.1038/s41557-023-01415-7.

[S9] M. Kuemin, S. Schweizer, C. Ochsenfeld and H. Wennemers, *J. Am. Chem. Soc.*, 2009, **131** (42), 15474–15482. DOI: 10.1021/ja906466q.

[S10] P. Thordarson, *Chem. Soc. Rev.*, 2011, **40**, 1305–1323. DOI: 10.1039/C0C00062K.

[S11] P. Wilhelm, B. Lewandowski, N. Trapp and H. Wennemers, *J. Am. Chem. Soc.*, 2014, **136** (45), 15829–15832. DOI: 10.1021/ja507405j.

[S12] E. Bitzek, P. Koskinen, F. Gähler, M. Moseler and P. Gumbsch, *Phys. Rev. Lett.*, 2006, **97** (17), 170–201. DOI: 10.1103/PhysRevLett.97.170201.
